# A screen for cell envelope stress uncovers an inhibitor of prolipoprotein diacylglyceryl transferase, Lgt, in *Escherichiacoli*

**DOI:** 10.1016/j.isci.2024.110894

**Published:** 2024-09-05

**Authors:** Kenneth Rachwalski, Sean J. Madden, Nicole Ritchie, Shawn French, Timsy Bhando, Adele Girgis-Gabardo, Megan Tu, Rodion Gordzevich, Rowan Ives, Amelia B.Y. Guo, Jarrod W. Johnson, Yiming Xu, Sharookh B. Kapadia, Jakob Magolan, Eric D. Brown

**Affiliations:** 1 Institute of Infectious Disease Research, McMaster University, Hamilton, ON L8S 4L8, Canada; 2 Biochemistry and Biomedical Sciences, McMaster University, Hamilton, ON L8S 4L8, Canada; 3 Department of Biochemical and Cellular Pharmacology, Genentech, South San Francisco, CA, USA; 4 Department of Infectious Diseases, Genentech, South San Francisco, CA, USA

**Keywords:** Microbiology, Biotechnology, Cell biology

## Abstract

The increasing prevalence of antibiotic resistance demands the discovery of antibacterial chemical scaffolds with unique mechanisms of action. Phenotypic screening approaches, such as the use of reporters for bacterial cell stress, offer promise to identify compounds while providing strong hypotheses for follow-on mechanism of action studies. From a collection of ∼1,800 *Escherichia coli* GFP transcriptional reporter strains, we identified a reporter that is highly induced by cell envelope stress—pProm_*rcsA*_-GFP. After characterizing pProm_*rcsA*_-GFP induction, we assessed a collection of bioactive small molecules for reporter induction, identifying 24 compounds of interest. Spontaneous suppressors to one compound in particular, MAC-0452936, mapped to the gene encoding the essential prolipoprotein diacylglyceryl transferase, *lgt*. Lgt inhibition by MAC-0452936 inhibition was confirmed through genetic, phenotypic, and biochemical approaches. The oxime ester, MAC-0452936, represents a useful small molecule inhibitor of Lgt and highlights the potential of using pProm_*rcsA*_-GFP as a phenotypic screening tool.

## Highlights

• Unbiased genetic screening identified pProm_rcsA_-GFP as a probe of envelope stress
• Genetic target list for *rcsA* reporter was annotated with CRISPRi and KO mutants
• Seven hundred ninety-seven bioactive molecules were tested for *rcsA* induction; 24 hits were identified
• The oxime ester MAC-0452936 was identified as an inhibitor of Lgt in *E. coli*

## Graphical abstract

**Figure:**
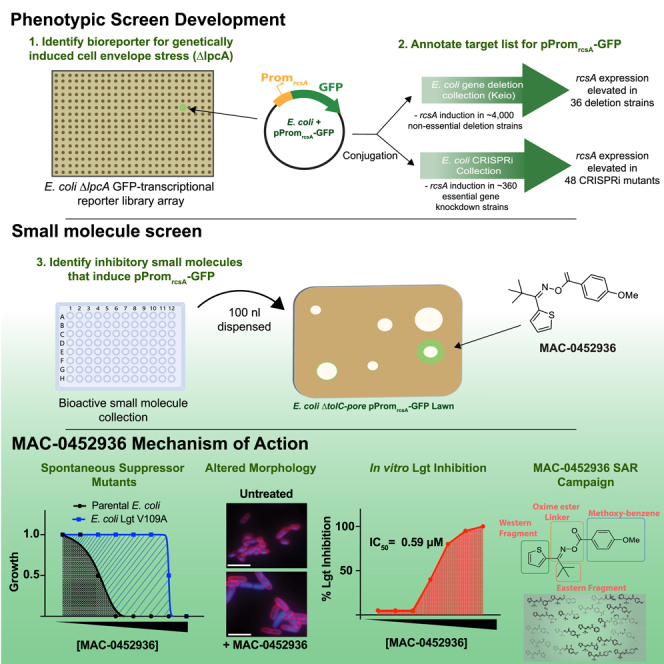

## Introduction

Bacterial cell envelope biogenesis has historically been a successful target for antibiotic discovery, yielding many clinically useful antibiotics.^1^ Of keen interest is the discovery of inhibitors that act on the cell envelope of Gram-negative pathogens, which cause many difficult-to-treat and often multi-drug-resistant infections.^2^ Further, as essential proteins involved in the Gram-negative cell envelope are largely found in the periplasm or as components of bacterial membranes, compounds targeting these processes will not be bound to the same permeability constraints as inhibitors of intracellular proteins. Nevertheless, many critical components of cell envelope biosynthesis and maintenance have unrealized potential as targets for antibiotics, providing an opportunity to discover chemical scaffolds that inhibit components of the cell envelope with unique mechanisms of action (MOAs).^3^

To this end, efforts have shifted to developing screening methodologies that can identify inhibitors of the synthesis and maintenance of the cell envelope.^4^ These methodologies can be broadly characterized as target-based,^5^^,^^6^^,^^7^^,^^8^^,^^9^^,^^10^^,^^11^^,^^12^ where a specific target protein in the cell envelope is chosen, and inhibitors are sought using biochemical screens. Alternatively, phenotypic approaches^13^^,^^14^^,^^15^^,^^16^^,^^17^ have employed assays that reveal cell envelope perturbation, and the compound’s precise MOA is determined later. The phenotypic screens also have value as secondary screening tools, as a follow-up to primary antibacterial screens that have prioritized compounds with whole-cell growth inhibitory activity. In this way, assays that reveal cell envelope perturbation phenotypes allow the discovery of new chemistry with strong prospects for the desired mechanism of action. Ultimately, *bona fide* hit compounds with known molecular targets are strong candidates for lead optimization and pre-clinical development.^18^^,^^19^^,^^20^^,^^21^^,^^22^^,^^23^^,^^24^^,^^25^^,^^26^

In the work described herein, we sought to examine an existing collection of ∼1,800 *Escherichia coli* GFP-transcriptional reporter strains^27^ to identify promoters that were induced in response to genetically imposed cell envelope stress. We identified the *rcsA* promoter construct (pProm_*rcsA*_-GFP) from the Rcs stress response pathway^28^ as an indicator strain to find small molecules acting on the Gram-negative cell envelope. With the goal of a comprehensive exploration of processes linked to pProm_*rcsA*_-GFP induction, we turned to genome-wide genetic perturbation. We constructed a mobile version of the reporter plasmid, which was conjugated into (1) the Keio collection^29^ of ∼4,000 non-essential gene deletion strains and (2) a collection of 356 CRISPRi mutants^30^ targeting the expression of essential genes in *E. coli*. This forward-genetic characterization of *rcsA* induction revealed an extensive array of both essential and non-essential genes, primarily related to the cell envelope, that induce promoter expression when perturbed. The preponderance of essential functions identified led us to use *rcsA* induction as a secondary screen to characterize a previously identified set of bioactive small molecules that inhibit the growth of a hyper-permeable and efflux deficient strain of *E. coli* (*E. coli ΔtolC-pore*), which permits identification of otherwise impermeable molecules. Our screen revealed 24 compounds that induce *rcsA* expression, where suppressor mutants for one compound, MAC-0452936, mapped to the essential gene encoding for prolipoprotein diacylglyceryl transferase, Lgt, involved in lipoprotein processing and one of the essential targets identified in our CRISPRi genetic screen. Herein, we provide both genetic and biochemical evidence supporting the inhibition of Lgt by MAC-0452936. In all, we present an important tool for identifying compounds that act on the Gram-negative cell envelope and report on a previously undiscovered class of Lgt inhibitors, the oxime esters.

## Results

### An unbiased genomics screen uncovers a highly expressed reporter for envelope stress

**Figure 1:**
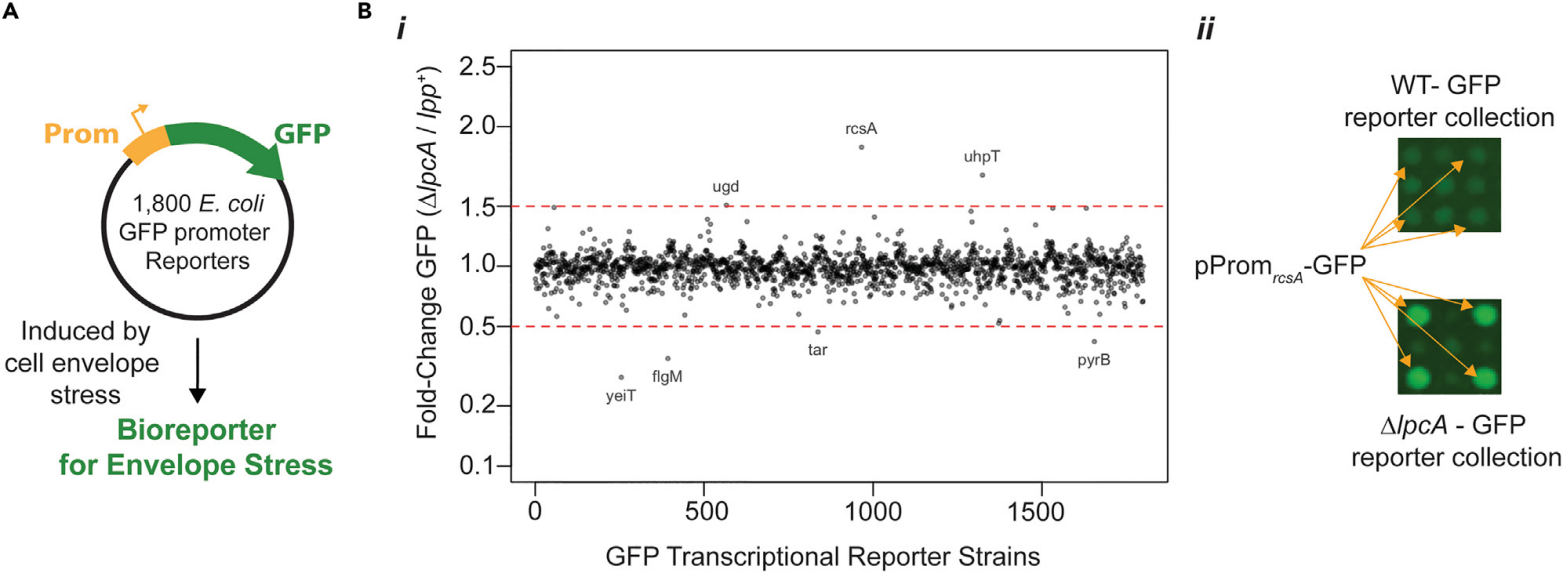
The transcriptional reporter for the gene *rcsA* was highly induced by cell envelope stress (A) A collection of ∼1,800 GFP transcriptional reporter plasmids was interrogated to identify a bioreporter induced by envelope stress. (B) A deletion of *lpcA* was introduced into the transcriptional reporter library to introduce broad cell envelope stress. The *ΔlpcA* and wild-type (*lpcA*^*+*^*)* reporter collections were grown on LB agar at 6,144 colony density—3 to 4 technical replicates of each reporter strain. Data are represented as mean. (i) An index plot showing the fold change of the average biomass normalized GFP expression of the *ΔlpcA* reporter collection relative to the wild-type collection. (ii) A zoomed-in quadrant from the *ΔlpcA* and wild-type reporter collection assay plate showing the four replicates of the most induced promoter, pProm_*rcsA*_-GFP.

To find compounds that act through the inhibition of the Gram-negative cell envelope, we sought to screen a collection of *E. coli* GFP transcriptional reporter strains^27^ to identify a suitable reporter (Figure 1A). We aimed to identify a GFP transcriptional reporter that satisfied two conditions: (1) induced in response to broad perturbations of the cell envelope (i.e., to capture most mechanisms of cell envelope stress) and (2) demonstrated a high magnitude of induction by cell-envelope-acting compounds. To that end, we employed high-throughput Hfr-mediated conjugation^31^^,^^32^ to introduce a deletion of the gene *lpcA*, which codes for the first committed step of lipopolysaccharide (LPS) core biosynthesis,^33^^,^^34^ into a collection of ∼1,800 *E coli* GFP transcriptional reporter strains^27^ to investigate the changes in basal GFP expression of each promoter in the deletion background. A deletion of *lpcA* in *E. coli* manifests as a deep truncation in LPS, resulting in wide-ranging envelope stress and sensitivity to large scaffold antibiotics.^33^^,^^34^^,^^35^ Both *ΔlpcA* and wild-type (*lpcA*^*+*^) transcriptional reporter collections were grown on solid agar in high-density colony arrays and were visualized for both GFP expression and colony biomass; then, GFP expression from each colony was normalized to its respective biomass (Data S1). From this screen, three reporter strains were found to be strongly induced in a *ΔlpcA* background: *rcsA, uhpT*, and *ugd* (Figure 1B). The highest GFP expression was seen in the *rcsA* GFP transcriptional reporter (pProm_*rcsA*_-GFP) strain, making it an excellent candidate tool for our screening platform. RcsA is a transcription factor component of the Rcs envelope stress regulon that responds to stringent negative regulation at both the transcriptional and post-transcriptional level by Hns and the Lon protease,^28^^,^^36^ respectively, likely resulting in the high levels of promoter activity observed compared to other members of the Rcs regulon.

### *rcsA* is induced by both chemically and genetically triggered envelope stress

Before applying *E. coli* pProm_*rcsA*_-GFP in a chemical screen to identify synthetic compounds that act on the cell envelope, we sought to understand the scope of envelope stress that results in the induction of this reporter. Although much is already known about the types of stress that induce the Rcs system in *E. coli*,^28^^,^^36^^,^^37^^,^^38^ many of these studies have not directly measured *rcsA* promoter activity. Hence, we opted to perform a comprehensive chemical and genetic characterization of the stresses that result in pProm_*rcsA*_-GFP induction. This analysis also served as a prospective target list for a chemical screen.

**Figure 2:**
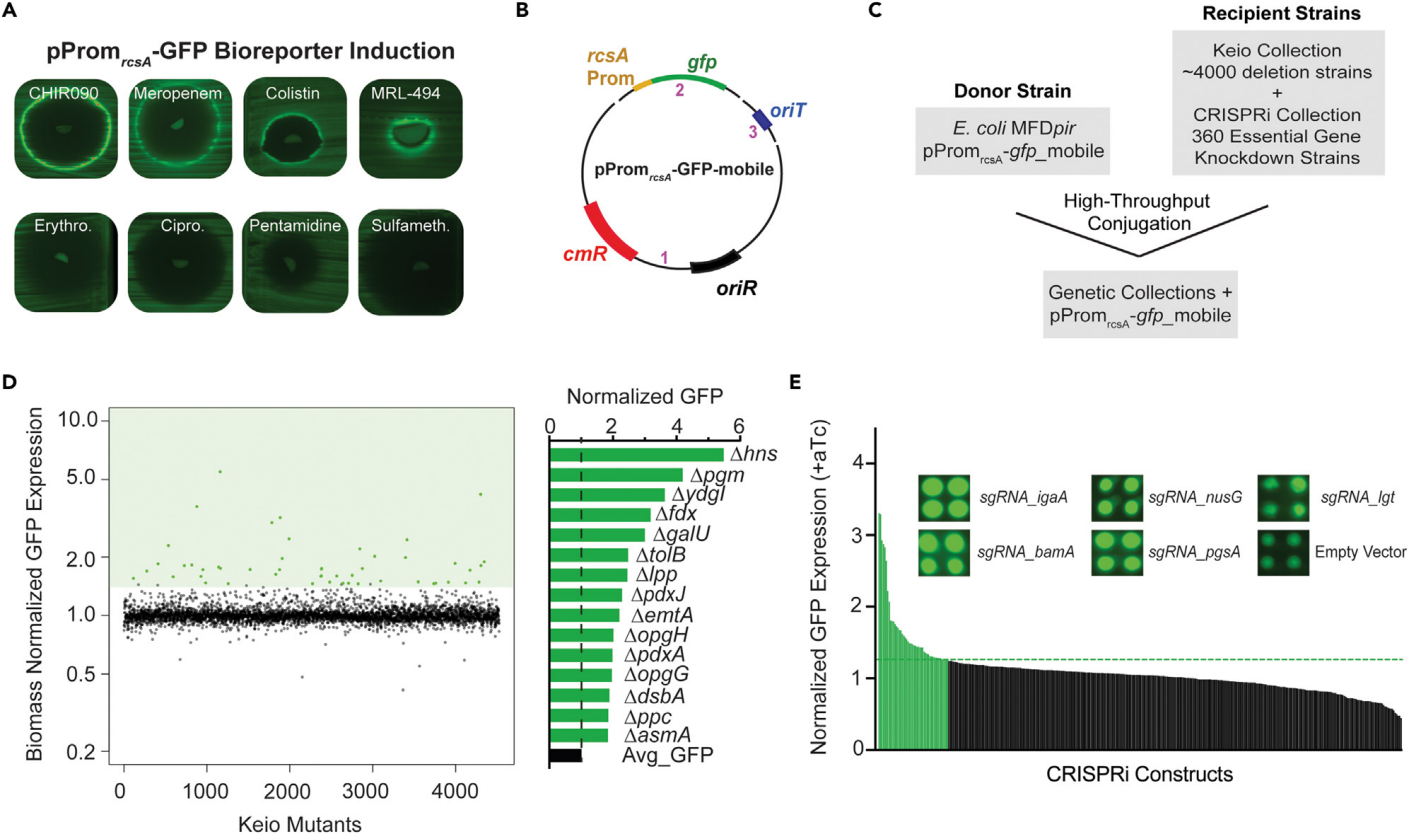
Genetic and chemical characterization in pProm_*rcsA*_-GFP revealed a large target list of cell-envelope-related processes (A) Disk diffusion assay with known antibiotics and chemical probes revealed compounds that induce pProm_*rcsA*_-GFP on LB agar and MOPS minimal agar. A total of 20 antibiotics were assessed for their ability to induce *rcsA*. Shown are eight representative disk diffusion assays on MOPS minimal agar: four compounds were found to induce the bioreporter and four did not. All plate images were taken on the Chemidoc MP (BioRad) using the Cy2 setting with a fixed exposure time (1 s). (B) Scheme showing the construction of pProm_*rcsA*_-GFP-mobile by Gibson assembly. The three PCR amplicons used in the assembly are labeled. (C) Workflow for the genetic screens characterizing induction of the bioreporter. (D) The Keio collection with pProm_*rcsA*_-GFP-mobile was grown on both LB agar and MOPS minimal agar in biological duplicate. Shown is an index plot of the average biomass-normalized GFP expression of the collection grown on MOPS-minimal agar. Strains that induced bioreporter expression were determined as those that deviated by at least three standard deviations from the mean of the dataset. The deletion mutants with the 15 highest measured GFP levels are shown in the adjacent bar plot. (E) The CRISPRi collection targeting essential gene knockdowns with pProm_*rcsA*_-GFP-mobile was grown on LB agar or MOPS minimal agar with and without aTc (100 ng/mL). Biomass-normalized GFP expression was further normalized to the average GFP production of the 17 empty vector controls found on the plate. CRISPRi strains that induce bioreporter expression were determined as those that increase GFP production by >25%. Shown is a rank-ordered bar plot of the average GFP production (*n* = 4) in MOPS-minimal agar. Representative images of five of the strongest hits from the induced plate are shown.

First, we aimed to determine the known antibiotics that induce pProm_*rcsA*_-GFP. We conducted disk diffusion assays with 20 antibiotics with diverse MOAs in a nutrient-deficient (MOPS minimal) medium and investigated which compounds resulted in *rcsA* induction (Figure 2A; Table 1). MOPS minimal medium was chosen due to the reduced background fluorescence observed compared to canonical mediums such as LB. Disk diffusion assays on solid agar enable the examination of promoter activity across a gradient of compound concentrations. Indeed, promoter activity screening in a liquid medium is challenging and limited by the screening concentration. If the compound concentration is too high, a potent growth inhibitory compound might be missed due to lack of growth, and if the concentration is too low, the biological activity will be missed due to a lack of induction. In the approach used here, promoter induction is visualized by a signature ring of GFP produced by bacteria that are directly proximal to the zone of inhibition from the antibiotic disk (Figure 2A). From these screens, we found the *rcsA* reporter was induced exclusively by antibiotic compounds that target the cell envelope of *E. coli* (Table 1), with rifampicin as an exception. Interestingly, the induction of the *rcsA* reporter strain by rifampicin was abolished in resistant isolates with point mutations in RpoB (data not shown), suggesting that rifampicin induces *rcsA* expression as a consequence of on-target activity. While this observation is intriguing, considering rifampicin’s established synergistic interactions with other antibiotics that cause envelope stress, for example, colistin,^39^^,^^40^ we chose not to further investigate this phenotype.

**Table 1:** Compounds that induced pProm_*rcsA*_-GFP in disk diffusion assays

Compound	Target	GFP expression
CHIR090	LpxC	++
MRL494	BamA	++
Triclosan	FAB	++
Rifampicin	RNA polymerase	++
Mecillinam	PBPs	++
Cefmetazole	PBPs	++
Penicillin G	PBPs	+
Meropenem	PBPs	+
Ampicillin	PBPs	+
Fosfomycin	MurA	+
Colistin	LPS	+
Pentamidine	Membrane	–
Vancomycin	PBPs, lipid II	–
Nalidixic acid	Gyrase, topo IV	–
Sulfamethoxazole	Folate biosynthesis	–
Ciprofloxacin	Gyrase, topo IV	–
Erythromycin	Ribosome	–
Tetracycline	Ribosome	–
Furazolidone	DNA	–
Trimethoprim	Folate biosynthesis	–

GFP expression by *E. coli* harboring pProm_*rcsA*_-GFP was assessed qualitatively by examining regions proximal to the zone of inhibition to classify compounds as not inducing GFP (−) or those that induced GFP production to a high (++) or intermediate (+) degree.

To supplement this characterization of *rcsA* induction with chemical probes, we endeavored to determine the genes in *E. coli* that lead to the induction of the bioreporter when disrupted either by genetic knockouts or by CRISPR interference (CRISPRi). Given available chemical probes target only a subset of *E. coli* essential biology, we reasoned that these genetic screens could further probe routes of perturbation that result in the induction of the bioreporter. To enable high-throughput assays with the Keio and the CRISPRi collections, we constructed a mobile version of the reporter plasmid with an origin of transfer (*oriT*) to enable conjugation and a compatible resistance cassette (*cmR*) to facilitate plasmid selection (Figure 2B). This mobile reporter plasmid was then introduced into the Keio collection of ∼4,000 *E. coli* non-essential gene deletion strains^29^ and a CRISPRi collection of 356 *E. coli* essential gene knockdowns^30^ by high-throughput conjugation performed in colony arrays (Figures 2C and S1).

To identify the non-essential genes that induce *rcsA* expression when perturbed, we grew the Keio collection containing the mobile pProm_*rcsA*_-GFP in colony arrays on a minimal medium, then imaged the array for colony biomass and GFP expression (Figure S2A, Data S2). It was evident that some colonies in the array expressed much higher levels of GFP than neighboring colonies with high reproducibility (Figure S2B). In sum, we found that 36 deletion strains exhibited elevated levels of *rcsA* promoter expression (Figure S2C). The highest level of GFP expression from our bioreporter was observed in a *Δhns* background, a known transcriptional repressor of *rcsA* (Figure 2D). Many of the other deletion mutant strains that resulted in bioreporter induction with both media conditions are known to cause gross perturbation of the cell envelope or result in envelope stress, including in *ΔrodZ*^41^*, ΔgalU*^42^*, Δlpp*^43^*, ΔwaaP*^35^*, ΔrfaH*,^35^ *Δpgm*,^35^ and *ΔasmA*.^44^

As with the Keio *rcsA* induction screen, the CRISPRi collection containing the mobile pProm_*rcsA*_-GFP was grown in colony arrays on MOPS minimal agar supplemented with the dCas9 inducer, anhydrotetracycline (aTc) (Figure 2E and Data S2). In the absence of CRISPRi induction, GFP fluorescence was uniform across the colony array; however, colonies with increased GFP production were clearly visible with the inducer supplemented into the agar (Figure S3A). In sum, we found that the bioreporter was induced in 48 CRISPRi mutants (Figure S3B). The highest levels of GFP production were seen in the CRISPRi strain silencing *igaA* expression, a known essential negative regulator of the Rcs pathway.^45^ An important consideration when interpreting CRISPRi data is the potential for constructs to silence the expression of downstream genes in the same operon. Other CRISPRi constructs that induced bioreporter expression included those targeting either genes upstream of, or involved in lipoprotein pre-processing (*lgt*, *lnt*), cardiolipin biosynthesis (*pgsA*), transcription and transcriptional termination (*nusA*, *nusG*, *rho*, *rpoC*), iron cluster assembly (*fdx*, *hscA*, *iscU*), and other processes that are more challenging to interpret (*racR*, *csrA*). Importantly, the CRISPRi results aligned with our chemical characterization of the *rcsA* reporter, where we noted induction with chemical inhibitors of LpxC and BamA. Further, we observed *rcsA* induction upon depletion of essential genes involved in transcriptional termination and multiple σ factors that supported our previous results showing induction of the bioreporter with an antibiotic targeting transcription, rifampicin.

In all, the genetic characterization of *rcsA* promoter induction revealed a compelling target list of essential and non-essential genes that are largely involved in cell envelope biogenesis. Interestingly, we also identified many genes without a clear link to the cell envelope that induced expression of our bioreporter when perturbed. We postulate that these processes could have a yet-to-be-characterized link to the cell envelope or play an enigmatic role in regulating expression from the *rcsA* promoter.

### A screen using the *rcsA*-bioreporter-identified cell-envelope-active small molecules

**Figure 3:**
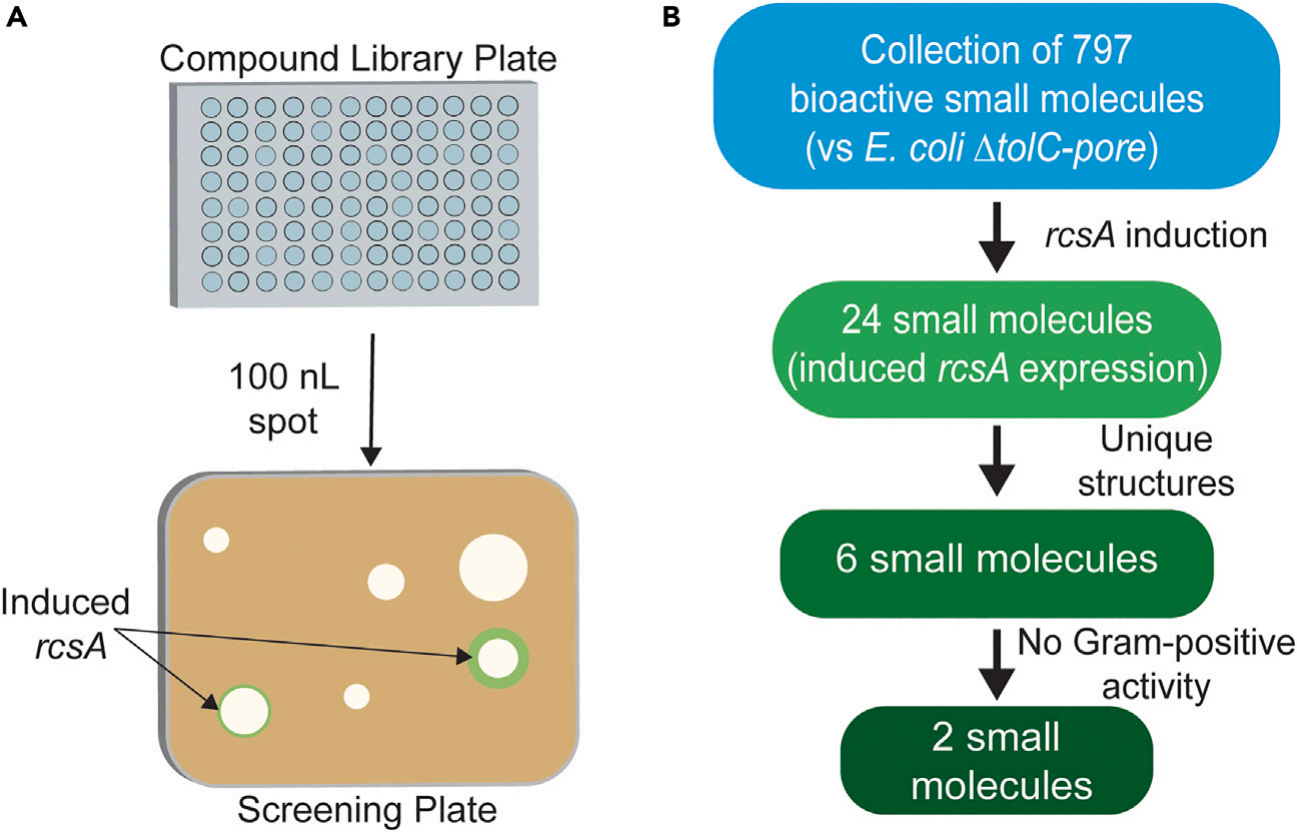
A screen for *rcsA* bioreporter induction identifies candidate cell-envelope-acting small molecules (A) Secondary screen of growth inhibitory compounds for bioreporter induction was conducted in medium throughput on MOPS-minimal agar to capture sub-inhibitory concentrations of molecules; 100 nL droplets of compounds from library stock plates (10 mM stock concentration) were spotted onto discrete positions of an uniwell plate inoculated with *E. coli ΔtolC*-pore pProm_*rcsA*_-mobile using acoustic dispensing. (B) The screening workflow undertaken in this study.

To identify small molecules that act on the Gram-negative cell envelope, a set of 797 bioactive compounds that were previously identified to be antibacterial against efflux-deficient hyper-permeable *E. coli* (*E. coli ΔtolC*-pore) was screened to determine those that can induce the *rcsA* bioreporter on MOPS minimal medium. *E. coli ΔtolC-pore* is deficient in TolC-mediated drug efflux and expresses an outer-membrane pore encoded by *fhuA*,^46^^,^^47^ thereby allowing the capture of additional antibacterial chemical scaffolds that would normally be subject to efflux or are unable to traverse the *E. coli* outer membrane. As the compounds in the bioactive compound collection were all antibacterial at varying inhibitory concentrations, we conducted this secondary screen using a modified solid agar diffusion assay. This enabled testing of these antibacterial small molecules at a range of concentrations, which is often required to assess bioreporter induction. To that end, 100 nL aliquots of compounds from screening library plates were deposited onto discrete positions on MOPS agar impregnated with *E. coli ΔtolC*-pore harboring the reporter (Figure 3A). Compounds that induced reporter expression were determined through visual analysis of the screening plates following overnight growth by looking for signature green “halos” proximal to the zones of inhibition (Figure S4).

From the small molecules assessed for induction of the *rcsA* bioreporter, 24 of the 797 (3% hit rate) were found to induce GFP production (Figure 3B and Data S3). Of these 24 compounds, six were prioritized for further follow-up based on structural novelty relative to known antibacterial scaffolds and were tested in dose for growth inhibition of *E. coli* and the Gram-positive organisms *Staphylococcus aureus* and *Bacillus subtilis* (Figure S5). We hypothesized that compounds active against *E. coli*, but not the Gram-positive organisms, might target processes involved in the Gram-negative outer-membrane synthesis, many of which were identified as possible targets in our genetic screen. Importantly, these processes are either absent or not essential for growth in Gram-positive organisms, offering promise for narrow-spectrum applications.

### MAC-0452936 mechanism of action elucidation

**Figure 4:**
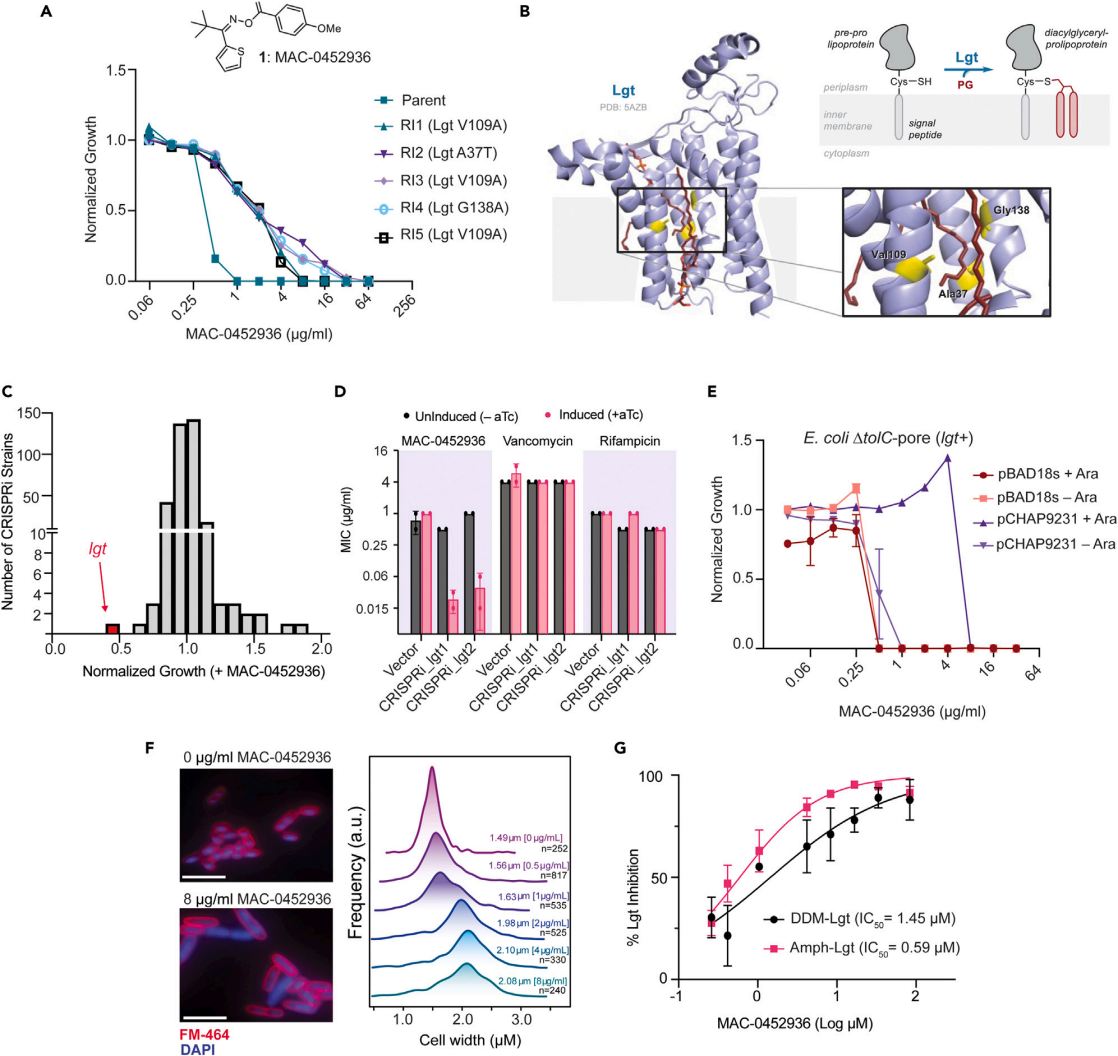
MAC-0452936 inhibited prolipoprotein diacylglyceryl transferase, Lgt (A) Growth inhibition of *E. coli ΔtolC-*pore, three independent MAC-0452936 resistance mutants with a V109A substitution in Lgt (2,959,071A→G), a mutant with an A37T substitution (2,959,288C→T), and a mutant with a G138A substitution (2,958,984C→G). The experiment was conducted in biological triplicate. Data are represented as mean ± SEM. (B) Structure of Lgt from *E. coli* with two phosphatidyl-glycerol (PG) moieties bound (PDB: 5AZB).^49^ Mutated residues from spontaneous resistance mutants are highlighted in blue on proximal α-helices, with the blue sphere highlighting the space in between the mutated residues where both PG moieties reside. (C) An unbiased fitness screen of 356 CRISPRi constructs in *E. coli ΔtolC-pore* identifies Lgt as the only essential gene that enhances MAC-0452926 activity upon knockdown. The CRISPRi array was pinned onto LB agar with or without 1 μg/mL of MAC-0452936 with basal levels of essential gene knockdown. The experiment was conducted in technical quadruplicate. Data are represented as mean. (D) MAC-0452936 MICs of *E. coli ΔtolC*-pore harboring CRISPRi plasmids targeting *lgt* with two different gRNAs or the vector control. dCas9 expression was induced by supplementing anhydrotetracycline (100 ng/mL) into the assay media. Vancomycin and rifampicin MICs were also conducted to control for enhanced envelope permeation. Two independent biological replicates are shown. Bars are represented as mean ± SEM. (E) Ectopic expression of *lgt* suppresses the MIC of MAC-0452936. MAC-0452936 dose responses in LB media were conducted with *E. coli ΔtolC-*pore harboring the *lgt* expression plasmid pCHAP9231 or the empty vector pBAD19s with or without 0.4% arabinose. Experiment was conducted in biological duplicate; data are represented as mean ± SEM. (F) Fluorescent micrographs of *E. coli ΔtolC-pore* treated with MAC-0452936. Cells were treated with increasing concentrations of MAC-0452936 for 1 h, then stained with both DAPI (DNA stain) and FM-646 (membrane stain). Scale, 5 μm. Cells were assayed at each concentration of MAC-0452936 in biological triplicate; a representative micrograph is shown. The width of at least 240 cells was measured at each MAC-0452936 concentration, with specific *n* values for each concentration of MAC-0452936 listed in the figure. (G) *In vitro* Lgt biochemical assay assessing dose response of MAC-0452936 enzyme inhibition of the enzyme. Two different Lgt preparations were used (DDM and amphipol preparations) and offered similar results. The assay was conducted in duplicate at each MAC-0452936 concentration. Data are represented as mean ± SEM.

Of the six compounds selected for follow-up, two were found to specifically inhibit *E. coli*, i.e., MAC-0447613 and MAC-0452936. Both compounds were active against *E. coli ΔtolC* and *E. coli ΔtolC*-pore strains but were inactive against wild-type (WT) *E. coli*, suggesting that they are both substrates for TolC-mediated efflux. Resistant mutants were selected for both MAC-0447613 and MAC-0452936 by plating *E. coli ΔtolC-*pore onto LB agar containing varying concentrations of compound (4×, 8×, and 16× MIC). No resistant isolates were identified for MAC-0447613; however, five independent resistant isolates were generated for MAC-0452936 at a frequency of resistance of 2 x 10^−8^. The isolated mutants displayed 8- to 16-fold resistance to MAC-0452936 (Figure 4A) and did not exhibit increased basal levels of Rcs stress response activation that might explain the observed resistance (Figure S6A), and whole-genome sequencing revealed that all isolates harbored point mutations in the gene coding for prolipoprotein diacylglyceryl transferase, Lgt. The point mutations in Lgt were the only mutations identified in all five isolates (the Illumina sequencing and BreSeq alignment files have been made available in an online data repository, https://zenodo.org/records/11068394). Lgt catalyzes the essential transfer of diacylglycerol from phosphatidyl-glycerol (PG) to the terminal cysteine of pre-prolipoproteins^48^ and was also identified as a possible target in our CRISPRi screen for *rcsA* bioreporter induction (Figures 2E and S3). When mapped to a recently solved crystal structure of *E. coli* Lgt,^49^ the three distinct variants identified in our resistant clones (A37T, V109A, and G138A) were found on three proximal α-helices in the substrate binding pocket of the enzyme (Figure 4B). Interestingly, complementation assays with Lgt variants have previously identified that Lgt remains active with a G138A substitution, but the enzyme does not tolerate substitutions with slightly larger amino acids (G138I and G138V).^49^ This suggests that residue 138 is important for enzyme activity and that substitution with larger amino acids may lead to steric inhibition of substrate binding.

We next turned to genetic approaches to confirm the mechanism of action of MAC-0452936. Recent work that characterized a macrocyclic-peptide inhibitor of Lgt demonstrated sensitization of *E. coli* to the inhibitor upon depletion of Lgt levels using CRISPRi.^12^ To that end, we screened the *E. coli* CRISPRi collection in the *ΔtolC*-pore genetic background with a sub-inhibitory concentration of MAC-0452936 and measured the fitness of all 356 CRISPRi mutants (Figure 4C and Data S4). Across the entire collection, the *lgt* CRISPRi mutant was the only mutant demonstrating a considerable growth defect in the presence of MAC-0452936 and did not demonstrate a growth defect when grown without compound or with sub-inhibitory concentrations of vancomycin, LolCDE-in-1, or CHIR090 (Figure S6B, Data S4). Interestingly, CRISPRi strains for the molecular targets of LolCDE-in-1 and CHIR090—the LolCDE complex and LpxC, respectively—showed the strongest growth defect in both respective screens, demonstrating the power of this approach to solve the MOA of antibacterial compounds. To confirm our large-scale screen, we then assessed the potency of MAC-0452936 and that of two negative control antibiotics, rifampicin and vancomycin, against *E. coli* harboring two CRISPRi constructs targeting *lgt* with different guide RNAs (Figure 4D). As expected, depletion of Lgt levels with either of the CRISPRi constructs sensitized *E. coli* to MAC-0452936 by 8- to 16-fold but not to either of the negative control antibiotics. Importantly, no sensitization to the compound was observed in the empty vector strain or strains harboring the *lgt* targeting CRISPRi constructs in the absence of CRISPRi induction. Depletion of Lgt by CRISPRi provided supporting evidence that MAC-0452936 targeted Lgt in *E. coli*; however, we sought to confirm this observation using a parallel genetic approach. We hypothesized that MAC-0452936 potency would also be affected by high-copy expression of *lgt*. To that end, we introduced either an arabinose inducible copy of *lgt* supplied on the pCHAP9231 plasmid^48^ or the pBAD19s plasmid as a control into *E. coli ΔtolC-*pore and tested the potency of MAC-0452936 (Figure 4E). Induction of *lgt* expression with arabinose suppressed the activity of MAC-0452936 by about 8-fold. We hypothesized that MAC-0452936 potency would exhibit a dose-response to arabinose concentrations using the pCHAP9231 *lgt* expression plasmid. To test this hypothesis, we constructed *E. coli ΔlgtΔtolC*-pore with pCHAP9231^48^ so that *lgt* expression was driven solely by arabinose from the plasmid. Like *E. coli Δlgt* pCHAP9231, *E. coli ΔlgtΔtolC-pore* pCHAP9231 requires arabinose for growth; however, we noted a longer lag phase and increased minimal requirement for arabinose in the double deletion strain compared to *E. coli Δlgt* (Figures S7A and S7B). As expected, a dose-dependent increase in the MIC of MAC-0452936 was observed with increasing arabinose concentrations, confirming that compound potency was correlated with Lgt levels in the cell (Figure S7C). A well-described pitfall of targeting lipoprotein transport is the high frequency of resistance arising from mutations in the gene encoding for murein lipoprotein, *lpp*.^13^^,^^50^^,^^51^^,^^52^ However, recent work has shown that rather than causing resistance, deletions of *lpp* sensitized *E. coli* to the Lgt inhibitor G2824 by about 2-fold.^12^ To test this observation with MAC-0452936, we introduced an *lpp* deletion into our screening strain *E. coli ΔtolC-*pore and assessed the activity of our Lgt inhibitor in this mutant background. As with G2824, we observed that deletion of *lpp* resulted in a similarly modest 2-fold sensitization to MAC-0452936 (Figure S8A).

With compelling genetic evidence for MAC-0452936 targeting Lgt in *E. coli*, we next investigated whether cells challenged with our inhibitor display phenotypes characteristic of Lgt inhibition. Perturbations in Lgt manifest as defects in bacterial cell morphology, including an increase in cell size (particularly in width), DNA leakage, and outer-membrane blebbing.^12^^,^^53^ Indeed, *E. coli ΔtolC-*pore treated with MAC-0452936 demonstrated an evident increase in cell size in addition to defects in DNA localization (Figure 4F). We found that treatment with MAC-0452936 resulted in a dose-dependent increase in cell width, consistent with previously described inhibitors of lipoprotein transport^12^^,^^13^^,^^17^^,^^54^ and genetic depletion of *lgt* expression.^12^^,^^53^

With both genetic and phenotypic evidence supporting MAC-0452936 inhibiting Lgt in *E. coli*, we next investigated whether MAC-0452936 could inhibit Lgt activity *in vitro*. Recently, a biochemical assay for Lgt activity was developed using phosphatidylglycerol and Pal-IAAC peptide as substrates.^12^ Using this assay, we determined that MAC-0452936 inhibited Lgt activity with an IC_50_ of 0.59 μM and 1.45 μM for Lgt purified using amphipol and DDM lipid matrices, respectively (Figure 4G). As similar results were obtained using two different Lgt enzyme preparations, the inhibition observed by MAC-0452936 was not dependent on the preparation of the lipid matrix. Together, we present genetic, phenotypic, and biochemical evidence that MAC-0452936 inhibits the growth of *E. coli* through inhibition of Lgt. MAC-0452936 represents a previously undiscovered small molecule inhibitor of bacterial Lgt, and the oxime ester represents a promising chemical scaffold for antibiotic discovery.

### MAC-0452936 is potentiated by SPR741 against wild-type *E. coli*

We next sought to determine whether we could achieve growth inhibition of wild-type *E. coli* using MAC-0452936 and an antibiotic adjuvant. Specifically, the outer-membrane binding adjuvant SPR741, a derivative of polymyxin B nonapeptide,^55^ has been shown to potentiate the activity of antibiotics that are inactive against *E. coli* due to either a lack of permeability or drug efflux.^39^^,^^56^^,^^57^ To that end, we determined the MIC of MAC-0452936 against wild-type *E. coli* in the presence and absence of a sub-MIC concentration of SPR741 (Figure S8B). Indeed, in the presence of 8 μg/mL SPR741, the MIC of MAC-0452936 was comparable to that of the *E. coli ΔtolC-*pore strain (1 μg/mL vs. 0.5 μg/mL). We leveraged this combination to characterize the spectrum of activity of MAC-0452936 against a panel of Gram-negative pathogens (Table 2). In the presence of 8 μg/mL SPR741, we observed MAC-0452936 activity against the clinical *E. coli* isolates C0244 and CFT073 at 2 μg/mL as well as against *Salmonella* Typhimurium SL1344 at 4 μg/mL. We did not observe MAC-0452936 activity with SPR741 against other Gram-negative pathogens tested, including *Pseudomonas aeruginosa*, *Klebsiella pneumoniae*, *Klebsiella aerogenes*, and *Acinetobacter baumannii*, or against the Gram-positive pathogen *S. aureus*. Interestingly, *S. aureus* has a functional homolog of Lgt but it is not essential for growth.^58^^,^^59^

**Table 2:** MAC-0452936 MICs against different pathogens in the presence and absence of outer-membrane permeabilizer SPR741

strain	MAC-0452936 + 0 μg/mL SPR741	MAC-0452936 + 8 μg/mL SPR741
*E. coli* BW25113	>64	1
*E. coli* CO244	>64	2
*E. coli* CFT073	>64	2
*S.* Typhimurium SL1344	>64	4
*P. aeruginosa* PA01	>64	>64
*K. aerogenes* CO045	>64	>64
*K. pneumoniae* MKP103	>64	>64
*A. baumannii* ATCC17978	>64	>64
*S. aureus* MRSA JE2	>64	>64

### MAC-0452936 requires growing cells for antibacterial activity

As Lgt is found at the inner membrane of Gram-negative bacteria, we sought to rule out physical perturbation of the membrane as the cause of the antibacterial activity observed for MAC-0452936. Membrane active molecules are known to maintain activity against non-growing bacteria in nutrient-deplete media^60^ and often demonstrate unfavorable hemolytic and cytotoxic characteristics.^47^ To that end, we treated both non-growing *E. coli ΔtolC*-pore in a nutrient-deplete medium (PBS) and actively growing *E. coli ΔtolC*-pore in a nutrient-replete medium (LB) with varying concentrations of MAC-0452936 and enumerated colony-forming units (CFUs) at different time points (Figure S9A). We found that MAC-0452936 could not kill non-growing bacteria but displayed potent bactericidal activity against actively growing bacteria. Indeed, MAC-0452936 reduced the bacterial counts of *E. coli ΔtolC-pore* to the limit of detection after 6 h at compound concentrations greater than 2 μg/mL. We then tested MAC-0452936 for hemolytic activity against human-derived red blood cells and cytotoxicity against HEK-293 cells. We did not observe hemolysis of human red blood cells challenged with MAC-0452936 (Figure S9B) or cytotoxicity to HEK-293 cells at MIC concentrations of MAC-0452936 (Figure S9C). However, we did observe a modest decrease in the viability of HEK-293 cells when treated with concentrations of MAC-0452936 greater than 8 μg/mL. Together, these data and our biochemical data on Lgt *in vitro* suggest that the antibacterial activity of MAC-0452936 is not due to the physical perturbation of the inner membrane but rather due to the inhibition of Lgt.

### Activity of MAC-0452936 analogs

The activity of MAC-0452936 with SPR741 against strains of *E. coli* and *S.* Tm encouraged us to synthesize a series of analogs to investigate structure activity relationships (SARs) (Data S5). We explored modifications to the “eastern” methoxyphenyl ring, the central oxime “linker” region, the “western” *tert*-butyl fragment, and the “southern” thiophene heterocycle (Table S1). The analogs were evaluated against a panel of bacterial strains, including strains of *E. coli* with improved permeability and/or impaired efflux (Δ*tolC*, pore, and Δ*tolC*-pore) and wild-type strains of *E. coli* and *S.* Tm in the presence and absence of SPR741. Although none of the analogs showed activity against wild-type strains, the MIC values against *E. coli* with SPR741 indicate that modifications to the “eastern” and “southern” rings can be tolerated. For example, each of the 2-fluoro, 3-pyridyl, and *O*-benzoyl “eastern” analogs, **2**, **3**, and **11**, respectively, retained activity (MIC = 0.5 μg/mL), but other subtle changes led to ≥4-fold reductions in potency. Similarly, replacement of the “southern” thiophene ring of **1** with a benzene delivered equal potency (**21**, MIC = 0.5 μg/mL), whereas the thiazolyl and pyridyl analogs **20** and **22** were ≥32-fold less active. In the “linker” region, extension by a single methylene diminished potency 8-fold (**16**, MIC = 4 μg/mL), and the *O*-sulfamoyl oxime analog **17**, which lacks the “eastern” aromatic ring, was inactive. Exploring “western” fragment modifications showed the *tert*-butyl group of MAC-0452936 to be superior to the methyl and ethyl groups of **18** and **19**. Interestingly, although the *E*- and *Z*-oxime isomers of **18** could be isolated and assayed separately, the *Z*-oxime stereochemistry is strongly favored for all *t*-Bu analogs. The bulky *t*-Bu substituent also forces the neighboring thiophene ring out of planarity with the oxime (Data S6), and it is possible that this conformation is more favorable for binding to Lgt.

Generally, the oxime analogs showed similar potencies (within 2-fold) against *E. coli* WT with SPR741 as they did against *E. coli* Δ*tolC*-pore and *E. coli* Δ*tolC* but were all inactive against *E. coli* pore. These results indicate that the oximes are TolC substrates and effluxed efficiently but also demonstrate that efflux can be overcome with the addition of outer-membrane permeabilizer SPR741, even more than overexpression of outer-membrane pores.

**Figure 5:**
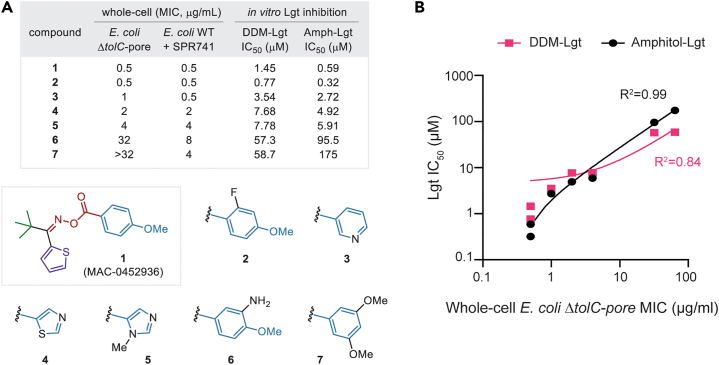
Whole-cell activity of MAC-0452936 analogs correlated with *in vitro* Lgt activity (A) Antibacterial activity (MICs) of MAC-0452936 (1) and synthetic analogs 2–7 against *E. coli ΔtolC-*pore and *E. coli* BW25113 with SPR741 (8 μg/mL) compared with their inhibitory activity (IC_50_s) *in vitro* against Lgt purified using either DDM- or amphipol lipid matrices. (B) Correlation of whole-cell MICs to *in vitro* Lgt IC_50_ values for seven analogs visualized in a scatterplot. For both DDM-Lgt and Amphipol-Lgt, each point in the scatterplot represents a different analog. A simple linear regression was used to determine the R^2^-correlation for both DDM-Lgt and Amphipol-Lgt.

We then aimed to correlate whole-cell activity with *in vitro* Lgt activity by assaying a selection of oxime analogs (**1**–**7**) that showed differential potencies against strains of *E. coli* (Figure 5A). *In vitro* IC_50_ values were determined for oximes against Lgt protein that was purified using either DDM- or amphipol lipid matrices. The resulting *in vitro* IC_50_ values for MAC-0452936 (**1**) and the six oxime analogs (**2**–**7**) against Lgt correlated remarkably well with their whole-cell MICs against *E. coli* with SPR741 and reasonably well with MICs against *E. coli ΔtolC-*pore (Figure 5B). This provided further evidence that the antimicrobial activity observed for MAC-0452936 was the direct result of Lgt inhibition in whole cells.

## Discussion

The Gram-negative cell envelope is an attractive target for antibiotic drug discovery, often selected by nature,^61^ that offers the potential to discover novel antibiotics with narrow spectrum activity.^54^ Here, we used a phenotypic screening approach, employing a reporter strain harboring pProm_*rcsA*_-GFP, to identify compounds that perturbed the Gram-negative cell envelope. The effort succeeded in identifying an oxime-ester-based small molecule inhibitor of Lgt, an essential enzyme involved in lipoprotein processing. The only previously identified inhibitor of Lgt in bacteria, G2824,^12^ is a macrocyclic synthetic peptide. Other synthetic small molecule inhibitors of lipoprotein processing and transport in *E. coli* have been identified, specifically a pyrazole compound,^17^ a 7-deazaxanthine compound,^13^ and a pyridine imidazole compound,^50^ which were all found to inhibit the LolCDE complex. Likewise, the natural products globomycin^51^^,^^62^ and myxovirescin^63^ are well-characterized natural product inhibitors of LspA. Inhibition of Lgt offers a distinct advantage to inhibition of downstream targets in lipoprotein transport including the Lol system and LspA, due to the insensitivity to mutations in *lpp*, a common mechanism for resistance to inhibitors of lipoprotein transport.^12^^,^^51^^,^^53^ Interestingly, two of the three small molecule inhibitors of *E. coli* LolCDE were identified using cell-stress-based phenotypic screening approaches measuring induction of the σ^E^ pathway^13^ and the promoter for the β-lactamase gene *ampC*,^17^ respectively.

In our study, we describe a genetic pipeline and screening methodology to identify GFP transcriptional reporters in *E. coli* responsive to cell envelope stress. Using Hfr-mediated conjugation,^31^^,^^32^ we introduced a deletion of *lpcA* into a collection of transcriptional reporter strains in *E. coli*^27^ to identify highly induced bioreporters for cell envelope stress. That effort revealed the pProm_*rcsA*_-GFP reporter that provided the phenotypic assay for our discovery effort. Similarly, the transcriptional reporter collection could also be screened with other gene deletions or with chemical inhibitors^64^ to probe different types of bacterial cell stress and identify bioreporters for the inhibition of other cellular processes. As such, these bioreporters for different cellular processes could likewise be screened to provide mechanistic hypotheses for compounds of interest in *E. coli.*

After identifying pProm_*rcsA*_-GFP as a reporter strain of interest, we then characterized the chemical and genetic basis for reporter induction. This was done by conducting disk diffusion assays with known antibacterial compounds and by constructing a mobile version of the bioreporter that could then be introduced into arrayed genomic collections by high-throughput conjugation. This comprehensive characterization of perturbations in both essential and non-essential genes provided a compelling target list as proof of principle for our small molecule screen but also uncovered insights into the genetic basis of *rcsA* induction in *E. coli*. Importantly, we identified that disruption of either of the known negative regulators of *rcsA* expression,^28^ *hns* and *igaA,* resulted in high levels of bioreporter induction, thus validating our genetic screening approach. Indeed, we believe that the approach described here could be a powerful phenotypic tool to rapidly characterize the regulation of promoters in bacteria. We believe that the majority of the other genetic processes that we identified to induce *rcsA* promoter expression likely result in some degree of envelope stress when disrupted. This includes Keio collection deletions in the genes *rodZ*,^41^ *lpp*,^43^ *pgm*,^35^ and *asmA*^44^ and genes involved in lipopolysaccharide biosynthesis (*rfaH*, *waaP*, *lpcA*),^35^ which all have characterized defects in the cell envelope. Likewise, from the CRISPRi screen, we identified that silencing expression in several genes involved in cell envelope biosynthesis and maintenance induced our bioreporter, including those involved in lipid-A biosynthesis (*lpxCD*), lipoprotein processing (*lgt*, *lspA*), *bamA*, *uppS*, and *glmU*. Chemical inhibitors of LpxC and BamA have also been shown^15^^,^^65^ to induce *rcsA*, providing validation to our CRISPRi screen. Interestingly, many of the genetic perturbations that we identified do not have a clear link to the cell envelope. Among these were CRISPRi knockdowns of genes involved in transcription, including those encoding RNA polymerase subunits (*rpoAC*), the σ factor *rpoD*, and the transcriptional termination factor *rho*. Additionally, we observed induction of pProm_*rcsA*_-GFP with rifampicin, an antibiotic targeting transcription, which we believe to be a result of on-target activity of the antibiotic. Indeed, rifampicin has been observed to demonstrate synergy with compounds targeting the cell envelope,^39^ and studies have linked mutations in RNA polymerase subunits to changes in the levels of peptidoglycan precursors and sensitivity to β-lactam antibiotics.^66^ The link between envelope stress and inhibition of transcription merits further investigation.

To search for antibacterial chemical matter targeting Gram-negative cell envelope, we used pProm_*rcsA*_-GFP as a secondary screen to characterize a collection of synthetic compounds with growth inhibitory activity against *E. coli*. Assays for the induction of a bioreporter are often conducted in broth,^13^^,^^15^^,^^17^^,^^65^ which could miss the critical sub-inhibitory concentrations at which bioreporter activation is observed with an antibiotic. Compound diffusion assays offer remarkable sensitivity to sub-inhibitory concentrations of a chemical inhibitor, including those that are nearly growth inhibitory. Indeed, we found that the majority of compounds that were found to induce pProm_*rcsA*_-GFP did so at concentrations directly adjacent to their respective zone of inhibition. This is particularly important in the context of screening compound libraries for bioreporter induction, where bioactive compounds typically display a range of antibacterial potencies. Conventionally, such compounds would need to be screened at different concentrations to assure sub-inhibitory levels, making higher throughput screening in liquid broth exceptionally challenging. Using this diffusion assay, we identified 24 small molecules that induced pProm_*rcsA*_-GFP including MAC-0452936, an oxime ester that we determined was a selective inhibitor of *E. coli* Lgt. Of note, Lgt was one of the gene disruptions found to induce our bioreporter in the CRISPRi genetic screen, providing further confidence in our genetically determined target list.

Just as for the Lgt inhibitor G2824,^12^ we observed a modest 2-fold increase in MAC-0452936 sensitivity in an *Δlpp* background. Interestingly, these data conflict with genetic studies that have shown that a deletion of *Δlpp* greatly increases the viability of mutants with reduced functional *lgt*.^53^ Indeed, inhibiting an enzyme is sometimes unlike limiting its copy through genetic perturbation, and thus, observed phenotypes could differ. For MAC-0452936, we observed a bactericidal mechanism of killing that requires actively growing cells (Figure S9A). Should a deletion of *Δlpp* promote the viability and growth of cells, then it may also sensitize those cells to the bactericidal action of the inhibitor, perhaps explaining the modest sensitization to MAC-0452936 observed. Rather than being sensitive to the presence or lack of *lpp*, we show that MAC-0452936 activity is sensitive to the copy number of Lgt in the cell. We demonstrate that high-copy expression of *lgt* from an inducible vector in a wild-type background suppresses the activity of MAC-0452936 (Figure 4E), and low-levels of expression of *lgt* from an inducible vector in an *lgt-*null background enhances MAC-0452936 activity relative to wild-type (Figure S7C). The high-copy suppression and low-copy enhancement phenotypes observed are consistent with that of other small molecule inhibitors targeting such processes and have been demonstrated with inhibitors of both soluble periplasmic and envelope proteins.^67^^,^^68^

Although inactive against wild-type *E. coli* due to compound efflux, we demonstrated potent activity of MAC-0452936 against wild-type isolates of *E. coli and S.* Tm when combined with the antibiotic adjuvant SPR741. We did not observe antimicrobial activity against the Gram-negative pathogens *A. baumannii*, *K. pneumoniae*, *K. aerogenes*, or *P. aeruginosa* regardless of SPR741. As SPR741 had been shown to work in each of these bacteria,^55^ we speculate that the inactivity in these backgrounds is due to differences in Lgt. Nevertheless, MAC-0452936 offers promise as a scaffold for future medicinal chemistry efforts to synthesize analogs or conjugate molecules with activity independent of SPR741. Our preliminary medicinal chemistry efforts determined a high tolerance for modifications to the thiophene and methoxybenzene groups of MAC-0452936, offering a wide chemical space for molecular substitutions. Together, our work showcases a pipeline for identifying and characterizing bioreporters for chemical screening that resulted in the identification of a previously undiscovered structural class of antibacterial compounds, the oxime esters.

### Limitations of study

Our study describes a comprehensive pipeline for antibiotic discovery through phenotypic screening of promoter-GFP reporter strains in *E. coli* that identified MAC-0452936 as an inhibitor of Lgt. However, some limitations in our study must be addressed. First, the GFP transcriptional reporter collection^27^ used in our genetic screen that identified pProm_rcsA_-GFP does not contain all of the promoters found in *E. coli*. In particular, this collection lacks intragenic promoters and promoters for non-coding genetic elements (sRNAs) that might be better probes than the promoters found in the collection, for example, for cell envelope stress. Secondly, this study described a small molecule screen of bioactive small molecules with activity against an efflux-deficient hyperpermeable strain of *E. coli* (*E. coli ΔtolC-*pore). Although this strain provided advantages to our study, such as allowing the screening of small molecules that are normally effluxed or impermeable to *E. coli*, follow-on chemical analoging to overcome the poor permeability characteristics of MAC-0452936 (medicinal chemistry/SAR efforts) was ultimately unsuccessful. We hope that the data presented in our study will prompt future efforts to overcome the permeability problems encountered by MAC-0452936. Finally, we used a combination of genetic, phenotypic, and chemical approaches to provide compelling evidence for the MOA of MAC-0452936—inhibition of Lgt in *E. coli*. Further experiments, that were not performed in our study, could be conducted to provide additional proof for the MOA of MAC-0452936. These experiments include tracking lipoprotein processing in cells treated with MAC-0452936 by western blotting, introducing the point mutations identified in the suppressor mutants into the Lgt expression plasmid, and expressing the mutant Lgt *in trans*, purifying recombinant Lgt with the identified point mutations for biochemical assays to determine the IC_50_ of MAC-0452936 against mutant Lgt, and isolating and solving the structure of an MAC-0452936 Lgt complex. These experiments could provide additional insight into the activity of MAC-0452936 and could be the focus of future studies investigating MAC-0452936 activity.

## Resource availability

### Lead contact

Further inquiries or requests can be directed to Eric Brown (ebrown@mcmaster.ca).

### Materials availability

All reagents will be available upon request to the lead contact.

### Data and code availability

• All data reported in this study are available in both the Supplemental Tables included in this publication or has been deposited onto Zenodo, to the NIH Sequenced Read Archive (SRA) as a bioproject, or to the Cambridge Crystallographic Data Centre (CCDC). Deposited data include the Illumina sequencing files of the suppressor mutants, the corresponding BreSeq alignment, and X-ray crystallography data for solving the structures for MAC-0452936 and analog *Z-*(18). For accession information please refer to the key resources table
• No original code was used in this publication. For the analysis pipeline used to process colony array images with ImageJ, and the R code that was used to normalize growth data, please refer to Rachwalski et al.^30^
• Any additional data or information required to reproduce the results reported in this study are available from the lead contact upon request.

## Acknowledgments

We thank Dr. Nienke Buddelmeijer (Institut Pasteur) for kindly providing the *lgt* expression vector pCHAP9231 and Dr. David Bikard (Institut Pasteur) for supplying the CRISPRi plasmid pFD152 (Addgene plasmid # 125546; http://n2t.net/addgene:125546; RRID:Addgene_125546). We also thank Dr. Michael Ellis for help and discussion in designing pProm_*rcsA*_-GFP-mobile, Dr. Maya Farha for reviewing the manuscript and helpful discussions throughput the project, Dr. James Britten for running crystallographic experiments, and Susan McCusker and the Center for Microbial Chemical Biology at McMaster University for their help with the secondary screen. We gratefully acknowledge Spero Therapeutics and Northern Antibiotics for providing samples of SPR741. This research was supported by a Discovery Grant for the 10.13039/501100000038Natural Sciences and Engineering Research Council of Canada (RGPIN-2019-07090) and by infrastructure funding from Canada Foundation for Innovation and the 10.13039/100012171Ontario Research Fund (ORF-RE09-047). E.D.B. was supported by a Tier I Canada research chair award, K.R. was supported by an Ontario graduate scholarship, M.M.T. was supported by Canada Graduate Scholarship, T.B. was supported by CIHR Postdoctoral fellowship.

## Author contributions

Conceptualization, K.R., N.R., A.BY.G., J.W.J., Y.X., S.B.K., J.K., and E.D.B.; methodology, K.R., N.R., J.W.J., Y.X., S.B.K., J.K., and E.D.B.; investigation, K.R., S.J.M., N.R., S.F., T.B., A.G., M.M.T., R.G., R.I., and Y.X.; formal analysis, K.R.; writing—original draft, K.R., J.W.J., and E.D.B.; writing—review & editing, M.M.T., R.G., J.W.J., Y.X., S.B.K., and E.D.B.; funding acquisition, E.D.B.; resources, J.K. and E.D.B; supervision, J.K. and E.D.B.

## Declaration of interests

The authors declare no conflicts of interest.

## STAR★Methods

### Key resources table

**Table:** 

REAGENT or RESOURCE	SOURCE	IDENTIFIER
**Bacterial and virus strains**		

Refer to Data S7	This Work	N/A

**Chemicals, peptides, and recombinant proteins**		

Anhydrotetracycline	Sigma-Aldrich	Cat#37919
Spectinomycin	Sigma-Aldrich	Cat#S4014
Kanamycin	Sigma-Aldrich	Cat#60615
LOLCDE-in-1	MedChem Express	Cat#HY-130839
Chloramphenicol	Sigma-Aldrich	Cat#C0378
CHIR-090	Sigma-Aldrich	Cat#SML3092
SPR741	Spero Therapeutics and Northern Antibiotics	N/A
Diaminopimelic acid (DAP)	Fisher Scientific	Cat#AAB2239103
MOPS-minimal media	TekNova	Cat#M2101
LB Broth, Miller	Fisher Scientific	Cat#DF0446
Agar	Fisher Scientific	Cat#BP1423
All other antibiotics used were purchased from Sigma-Aldrich unless otherwise stated	Sigma-Aldrich	Variable
Hexafluoroisopropylsulfamate (HFIPS)	Enamine	Cas# [637772-38-4]
Dess-Martin periodinane	AK Scientific	Cas# [87413-09-0]
3-quinolinecarboxylic acid	AK Scientific	Cas# [6480-68-8]
2-picolinic acid	AK Scientific	Cas# [98-98-6]
4-(methylamino)benzoic acid	AK Scientific	Cas# [10541-83-0]
3,5-dimethoxybenzoic acid	AK Scientific	Cas# [1132-21-4]
2-thiophenecarboxaldehyde	AK Scientific	Cas# [98-03-3]
2-fluoro-4-methoxybenzoic acid	AK Scientific	Cas# [394-42-3]
3-amino-4-methoxybenzoic acid	AK Scientific	Cas# [2840-26-8]
4-(trifluoromethyl)benzoic acid	AK Scientific	Cas# [455-24-3]
EDC·HCl	AK Scientific	Cas# [25952-53-8]
DMAP	AK Scientific	Cas# [1122-58-3]
Acetylthiophene	Oakwood Chemical	Cas# [88-15-3]
hexafluoroisopropanol	Oakwood Chemical	Cas# [920-66-1]
2-Propionylthiophene	AmBeed	Cas# [13679-75-9]
4-chlorobenzoic acid	AmBeed	Cas# [74-11-3]
1-methyl-1*H*-imidazole-5-carboxylic acid	AmBeed	Cas# [41806-40-0]
2-pivaloylthiophene	AmBeed	Cas# [20409-48-7]
4-methoxybenzoic acid	AmBeed	Cas# [100-09-4]
Benzoic acid	Fisher Scientific	Cas# [65-85-0]
*t*-BuMgCl (1.0 M in THF)	Sigma-Aldrich	Cas# [677-22-5]
2,2,2-Trimethylacetophenone	Aaron Chemicals	Cas# [938-16-9]
2-(4-methoxyphenyl)acetic acid	Aaron Chemicals	Cas# [104-01-8]
isoxazole-5-carboxylic acid	Aaron Chemicals	Cas# [21169-71-1]
6-methoxynicotinic acid	Aaron Chemicals	Cas# [66572-55-2]
Thiazole-5-carboxylic acid	Combi-Blocks	Cas# [14527-41-4]
nicotinic acid	Combi-Blocks	Cas# [59-67-6]
NaOAc	BioShop Canada	Cas# [127-09-3]

**Experimental models: Cell lines**		

HEK293t Cells	ATCC	Cat# CRL-11268

**Deposited data**		

*E. coli Δ*tolC-pore parental and MAC0452936 resistant isolate whole genome sequencing raw fastq files	This Work	NCBI Sequenced Red Archive (SRA) Accession number SRA: PRJNA1152998
MAC0452936 spontaneous suppressor BreSeq alignment	This work	https://doi.org/10.5281/zenodo.11068394
Crystallographic data for MAC-0452936	This work	CCDC: 2309307
Crystallographic data for analog (Z)-18	This work	CCDC: 2313967

**Oligonucleotides**		

Refer to Data S7	This work	N/A

**Software and algorithms**		

GraphPad PRISM 9	GraphPad	https://www.graphpad.com/
CRISPRbact sgRNA design tool	Cui et al.^69^	https://gitlab.pasteur.fr/dbikard/crisprbact
ImageJ	ImageJ	https://imagej.net/ij/ij/
ImageJ colony biomass quantification script	French et al.^70^; Rachwalski et al.^30^	N/A

**Other**		

Epson Perfection V750 high-resolution scanner	Epson	NA
Singer ROTOR+	Singer Instruments	https://www.singerinstruments.com/solution/rotor/
Singer RePads (384 Long and 1536 short)	Singer Instruments	Cat#REP-003; Cat#REP-005
Singer PlusPlates	Singer Instruments	Cat#PLU-003
Biotek Synergy *Neo*2 Plate Reader	BioTek	N/A

### Experimental model and study participants

All bacterial strains and genomic collections used in this study are listed in Data S7. Bacteria were cultured in LB at 37°C (10 g/L NaCl, 10 g/L tryptone, 5 g/L yeast extract), supplemented with antibiotics where appropriate (kanamycin, 50 μg/mL; chloramphenicol, 25 μg/mL; apramycin, 100 μg/mL; and spectinomycin, 150 μg/mL) and with 15 g/L agar for experiments on solid medium. MOPS minimal medium supplemented with 0.4% glucose (Teknova) was prepared according to the manufacturer’s instructions: components were filter sterilized after preparing liquid growth medium or added to sterile water and agar (1.5% w/v) for solid growth medium. Media was supplemented with 0.4 mM diaminopimelic acid (DAP) when required. Antibiotics were obtained from Sigma.

To determine the haemolytic activity and cytotoxicity of MAC-0452936, pooled human blood and HEK293t cells were used, respectively. Fresh pooled human blood for haemolytic assay was acquired from STEMCELL Technologies, whilst, HEK293t cells were originally acquired from ATCC (Cat# CRL-3216) and were regularly grown in Eagle’s minimal essential medium (DMEM) with 10% fetal bovine serum (FBS), L-glutamine and incubated at 37°C. Cell lines are regularly tested for mycoplasma contamination.

### Method details

#### Genetic manipulation, cloning and plasmids used in study

CRISPRi strains targeting *lgt* expression were constructed by modifying of the CRISPRi plasmid pFD152 by Golden Gate assembly as previously described,^71^ before transforming into the assay strain – *E. coli ΔtolC-pore*. Deletion of *lgt* in *E. coli ΔtolC*-pore pCHAP9231 was accomplished by standard λ-RED recombination methodology^72^ in the presence of arabinose supplementation (0.4%). The mobile *rcsA* plasmid, pProm_*rcsA*_-mobile, was constructed by Gibson assembly using the NEBuilder HiFi Assembly Kit (NEB) with three PCR amplified fragments; the promoter-reporter fragment from the corresponding plasmid in the transcriptional reporter collection,^27^ the chloramphenicol resistance cassette, *oriR* from pACYC184, and *oriT* from a synthesized gene fragment (BioBasic). All primers used in this study to generate strains and plasmids are listed in Data S7.

#### Antimicrobial-susceptibility testing and disk diffusion assays

For MIC assays, cultures of bacteria were grown in LB broth. Overnight cultures were diluted (1:100) into fresh LB media and grown to the mid-log phase of growth (OD_600_ ∼0.5). Subcultures were then normalized to an OD_600_ of 0.1, and diluted (1:5000) into assay media. Assay plates were then incubated for 18 h at 37°C, then OD_600_ was measured using a Biotek *Neo* plate reader.

For disk diffusion assays, overnight cultures of *E. coli* pProm_rcsA_-GFP were grown in LB broth, and then were spread onto LB or MOPS agar using sterile cotton swabs. Antibiotic disks were placed onto inoculated agar plates using sterile tweezers, and plates were incubated for 18 h at 37°C. Disk diffusion plates were then imaged for GFP production using the Chemidoc MP (BioRad).

#### High-throughput conjugation for genomic screens

For generating the *ΔlpcA* transcriptional reporter collection, a strain harboring an apramycin-marked *lpcA* deletion (*E. coli ΔlpcA::apraR*^73^) was used as the donor strain for conjugation into a collection of ∼1,800 transcriptional reporter strains (*kanR*)^27^ using synthetic genetic array methodology previously described by Côté et al.^74^ and Klobucar et al.^73^ In short, *E. coli ΔlpcA::apraR* was first made competent for conjugation through mating with pseudo-F+ *E. coli* strains harboring a chromosomal integrative plasmid containing the machinery required for conjugation,^31^ as previously described.^73^ The *ΔlpcA* Hfr^+^ conjugative donor strain and the GFP transcriptional reporter collection were then arrayed on LB agar at 1,536 colony density using the Singer ROTOR, then co-pinned onto LB agar without antibiotic selection and incubated at 30°C for 8 h for mating. Exconjugants were then selected by pinning the conjugation mixture onto LB-agar with both apramycin and kanamycin.

For generating the pProm_*rcsA*_-GFP-mobile Keio collection and the pProm_*rcsA*_-GFP-mobile CRISPRi collection, pProm_*rcsA*_*-*GFP-mobile was first transformed into the conjugative donor strain *E. coli* MFD*pir*,^75^ which is auxotrophic for DAP. *E. coli* MFD*pir* pProm_*rcsA*_-GFP-mobile was then pinned onto LB agar with chloramphenicol and DAP at 1,536 colony density. The Keio and CRISPRi collections were pinned at 1,536 colony density onto LB agar supplemented with kanamycin or spectinomycin, respectively. The conjugative donor strain and the genomic collections were then co-pinned onto LB agar supplemented with DAP and incubated at 37°C for 2 h for mating. Exconjugants were selected by pinning conjugation mixtures on LB plates supplemented with either kanamycin and chloramphenicol or spectinomycin and chloramphenicol for the Keio collection or CRISPRi collection, respectively.

#### Genomic screening, imaging and analysis

Following conjugation, genomic collections harboring pProm_*rcsA*_*-*GFP-mobile were arrayed on either LB or MOPS minimal agar with appropriate selection and grown for 18 h at 37°C. Colonies were then imaged for GFP expression using the Chemidoc MP (Biorad), and for colony biomass using high-resolution scanners (Epson Perfection v750). Colony biomass and GFP production were quantified using previously described approaches,^70^^,^^76^ and the calculated GFP production of each colony was normalized to calculated colony biomass. For the genomic screen with the CRISPRi collection, the genomic collection was also grown on LB agar and MOPS minimal agar supplemented with 100 ng/mL anhydrotetracycline (aTc) to induce dCas9 expression.

#### Secondary screen for GFP expression

For the small molecule secondary screen, an overnight culture of *E. coli ΔtolC-*pore harboring pProm_*rcsA*_*-*GFP-mobile was prepared, then used to impregnate molten MOPS-minimal agar at an OD_600_ of ∼0.1. The inoculated molten agar was then poured into uniwell PlusPlates (Singer Instruments), was left to solidify at room temperature, then was dried briefly in a laminar flow cabinet. The Echo liquid handler (Beckman-Coulter) was then used to spot 100 nL aliquots of bioactive small molecules directly from library plates onto discrete, evenly spaced positions on the uniwell agar plate such that 24 small molecules were screened on each place. Screening plates were then incubated for 18 h at 37°C and visualized for GFP production using the Chemidoc MP (BioRad). Compounds that induced GFP production were identified by visual identification.

#### Selection and characterization of spontaneous resistance mutants

A culture of *E. coli ΔtolC-pore* was grown overnight in LB broth at 37°C with shaking, then, ∼6 × 10^8^ CFU in 100 μL of LB broth was plated on LB agar plates containing 4, 8, or 16 μg/mL of MAC-0452936. Plates were then incubated at 37°C, and examined every 24 h for the emergence of resistant colonies. Colonies began to emerge after 48 h of incubation and were purified by restreaking on LB agar and LB agar containing the same concentrations of MAC-0452936 from which the colonies originally appeared. Five independent MAC-0452936 resistant colonies were selected for whole genome sequencing. Chromosomal DNA from the resistant mutants and the parent *E. coli ΔtolC-pore* was isolated using the DNeasy Blood and Tissue Kit (Qiagen). DNA samples were sequenced on the Illumina MiSeq platform (SeqCenter LLC) and reads were aligned to the *E. coli* BW25113 reference genome using Breseq^77^ (GenBank: CP009273.1). Raw Illumina sequencing data is on the NCBI Sequenced Read Archive (SRA: PRJNA1152998) and the BreSeq output is available on Zenodo (https://doi.org/10.5281/zenodo.11068394).

#### CRISPRi screens and CRISPRi *lgt* knockdown

For screens of the CRISPRi collection with MAC-0452936, the mobile CRISPRi collection was first conjugated into *E. coli ΔtolC-pore* as previously described.^30^ The CRISPRi collection was then arrayed on solid agar at 1536 density using the Singer-ROTOR, such that each CRISPRi strain is arrayed in technical quadruplicate. The CRISPRi collection was then pinned onto LB agar plates containing ¼ MIC concentrations of MAC-0452936 (1 μg/mL), vancomycin (250 ng/mL), LolCDE-in-1 (6 ng/mL), and CHIR090 (7 ng/mL), then grown for 18 h at 37°C. The screens were conducted without CRISPRi induction, using just the 5–10% basal knockdown from the pFD152 CRISPRi plasmid. Following overnight growth, plates were imaged using high-resolution scanners (Epson Perfection v750), and colony biomass for each mutant was determined.

For CRISPRi Lgt low-copy enhancement assays, two independent 20 base pair gRNAs targeting *lgt* expression were designed using predictive software^69^^,^^78^ to optimize on-target activity and minimize off-target binding in *E. coli* BW25113. Guide RNAs were then cloned into the CRISPRi plasmid pFD152,^71^ and guide RNA sequences were verified by Sanger sequencing. CRISPRi constructs targeting *lgt*, or the empty vector harboring a spacer sequence *in lieu* of a specific guide RNA, were transformed into *E. coli ΔtolC-*pore for MIC assays with MAC-0452936. MIC assays were conducted in the presence of 100 ng/mL aTc to induce dCas9 expression.

#### Live cell fluorescence microscopy and image quantification

An overnight culture of *E. coli* ΔtolC-pore was prepared, then diluted (1:100) into fresh LB media and grown to the mid-exponential phase of growth (OD_600_ ∼0.5). Cultures were then challenged with different concentrations of MAC-0542936 (4 μg/mL) for 1 h at 37°C with shaking, Cell suspensions were transferred to a poly-lysine coated 0.17 mm glass-bottom imaging plate (Brooks Scientific) and exposed to FM4–64 (1 μg/mL final concentration; Invitrogen) and DAPI (0.2 μg/mL final concentration; Invitrogen) probes for 10 min in the dark. Samples were imaged using a Nikon Eclipse Ti inverted microscope at 100× magnification, with a minimum of 200 cells counted per image. Images were quantified with ImageJ, using the following macro. First, channels were split to use only the FM4-64 channel, and image backgrounds were subtracted with a 40-pixel rolling ball radius with smoothing disabled. The Otsu method was used as a thresholding algorithm to convert to a binary image, and holes were filled resulting in solid cells. A watershed algorithm was used to separate adjoined cells, then measurements were taken, compiled, and loaded into the R statistical programming language to visualize. Density plots were generated from cell width measurements, then were overlaid and offset in the y-dimension for ease of comparison.

#### Lgt biochemical assay

Expression, purification, and *in vitro* Lgt biochemical assay were conducted as described by Diao et al.^12^

#### Time-kill assays

*E. coli ΔtolC-*pore was grown overnight in LB broth. For killing in nutrient-deplete media, overnight culture was pelleted by centrifugation at 5,000*g* for 30 min, and resuspended in sterile PBS buffer. Cells in PBS were added to a microwell plate containing different concentrations of MAC-0452936 (100 μL final volume), and incubated for the specified time at 37°C with shaking. At the specified time points, cultures were pelleted by centrifugation 5,000*g* for 30 min at 4°C, then 10-fold serially diluted in PBS before being plated on LB agar for CFU enumeration. For killing of actively growing cells in nutrient replete media, overnight culture of *E. coli ΔtolC-*pore was diluted 1/1000 into fresh LB broth and grown at 37°C with shaking to the early-log phase of growth (∼2.5 h, OD_600_ ∼0.2). Cells in LB broth were then added to a microwell plate containing different concentrations of MAC-0452936 (100 μL final volume), and incubated for the specified time at 37°C with shaking. At the specified time points, cultures were pelleted by centrifugation 5,000*g* for 30 min at 4°C, then 10-fold serially diluted in PBS before being plated on LB agar for CFU enumeration.

#### Haemolysis assay

Haemolytic activity of MAC-0452936 was assessed as described previously. Fresh human blood (10 mL, Stem Cell Technologies, USA) was centrifuged at 4,000 rpm for 10 min. The supernatant was discarded, and the pellet was rinsed three times with 1× PBS. Subsequently, the pellet was diluted 1:5 (v/v) in 1× PBS and RBC suspensions (0.5 mL each) were incubated with varying concentrations of test compounds at 37°C for 1 h. Following this, the cell suspensions were centrifuged at 5,000*g* for 10 min and the supernatant was meticulously collected without disturbing the pellet. Hemolysis was assessed by measuring the absorbance of the released hemoglobin in the supernatant at 540 nm using a spectrophotometer. Triton X-100 (0.1%) was used as the positive control causing 100 per cent hemolysis. All experiments were performed in triplicates.

#### *In vitro* cytotoxicity

The cytotoxicity of MAC-0452936 on HEK293t cells was determined by performing a cell viability assay using PrestoBlue Cell Viability Reagent (Invitrogen). 3 × 10^4^ cells/mL were seeded in tissue culture-treated 96-well plates (Nunc; white opaque with clear bottom) in Eagle’s minimal essential medium (DMEM) with 10% fetal bovine serum (FBS), L-glutamine and incubated at 37°C for 24 h in 5% CO_2_. The cells were then treated with varying concentrations of MAC-0452936 and similar percentage of DMSO as in drug (solvent control) for 24 h at 37°C. PrestoBlue reagent (20 μL) was added to the wells and incubated for 1 h in the CO_2_ incubator before reading the plates at excitation/emission wavelength of 560/590 nm on the Biotek Synergy *Neo*2 plate reader. Fluorescence values were expressed as percentage and inhibition with respect to solvent control was calculated.

#### NMR of MAC-0452936 and analogs

Chemical shifts in ^1^H NMR and ^13^C NMR spectra are reported in parts per million (ppm) relative to tetramethylsilane (TMS), with calibration to TMS (δ_H_, δ_C_ 0.0) or the residual solvent peaks according to values reported by Gottlieb et al.^79^ (chloroform: δ_H_ 7.26, δ_C_ 77.16; dichloromethane: δ_H_ 5.32, δ_C_ 54.00; acetone: δ_H_ 2.05, δ_C_ 29.84, 206.26; methanol: δ_H_ 3.31, δ_C_ 49.00; DMSO: δ_H_ 2.50, δ_C_ 39.52). When peak multiplicities are given, the following abbreviations are used: s, singlet; d, doublet; t, triplet; q, quartet; sept., septet; dd, doublet of doublets; m, multiplet; br, broad; app., apparent; *gem*, geminal. ^1^H NMR spectra were acquired at 400 or 700 MHz with a default digital resolution (Brüker parameter: FIDRES) of 0.22 and 0.15 Hz/point, respectively. Coupling constants reported herein therefore have uncertainties of ±0.4 Hz and ±0.3 Hz, respectively. All assignments of protons and carbons relied on data from 2-dimensional NMR experiments including COSY, HMQC, and HMBC. The ^13^C NMR spectra provided herein (^13^C{^1^H} DEPTQ-135; Brüker pulse program deptqgpsp) show CH and CH_3_ carbon signals above the baseline and C and CH_2_ carbons below the baseline. Melting points (mp) are uncorrected. Reactions were carried out at room temperature (rt) if temperature is not specified. Compounds purified by normal-phase flash chromatography used Teledyne Combi*Flash* Rf+ and NextGen 300+ purification systems (www.teledyneisco.com) with pre-packed silica cartridges (either 40–63 μM or 20–40 μM particle size). High-resolution mass spectrometry (HRMS) data was obtained using a Brüker micrOTOF II system with electrospray ionization (ESI) and paired with an Agilent HPLC and UV detector.

#### MAC-0452936 and (*Z*)-18 X-Ray crystallography

Crystals of MAC-0452936 (1) were obtained by vapor diffusion over a period of 5 days using EtOAc and hexanes. A suitable crystal was mounted on a Brüker Venture Photon III diffractometer. The crystal was kept at 296 K during data collection. Using Olex2,^80^ the structure was solved with the XT^81^ structure solution program using Intrinsic Phasing and refined with the XL^82^ refinement package using Least Squares minimization. Crystallographic data for MAC-0452936 (1) has been deposited in the Cambridge Crystallographic Data Center database (CCDC: 2309307).

Single crystals of (*Z*)-**18** were obtained by the vapor diffusion method over a period of 5 days using EtOAc and hexanes. Crystallographic data has been deposited in the Cambridge Crystallographic Data Center database (CCDC: 2313967).

#### Synthesis of MAC-0542936 and analogues

##### General procedures

**Figure:**
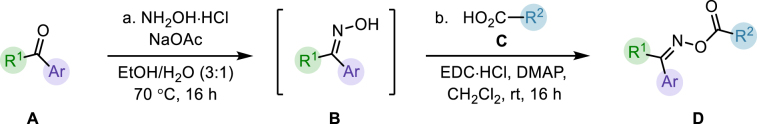

###### General procedure A

As a modification of a known procedure,^83^ NH_2_OH·HCl (6 equiv) and NaOAc (1 equiv) were combined with ketone **A** (1 equiv) in EtOH and H_2_O (3:1) was heated to 70°C and stirred overnight. After 16 h, the reaction mixture was cooled to rt, diluted with water, and extracted with EtOAc (3×). The combined organic extracts were washed with H_2_O (3×) and satd NaCl, dried over Na_2_SO_4_, and concentrated under reduced pressure. Some crude oximes **B** were purified by normal-phase chromatography (EtOAc/Hex) on silica gel (S1–**S3**), but others were used in the following step without further purification (**S6**, **S7**, S10). For details, see the procedures below.

###### General procedure B

Oxime **B** (1 equiv) was added at rt to a solution of carboxylic acid **C** (1 equiv), EDC·HCl (2.5 equiv), and DMAP (0.1 equiv) in CH_2_Cl_2_ (0.1 M) and the reaction was stirred under argon at room temperature for 16 h. The reaction mixture was diluted with CH_2_Cl_2_, washed with satd Na_2_CO_3_ and satd NaCl, dried over Na_2_SO_4_, and concentrated under reduced pressure. The *O*-acyloxime product **D** was purified by normal-phase flash chromatography on silica gel (EtOAc/Hex).

###### (*Z*)-2-Pivaloylthiophene oxime (S1)

**Figure:**
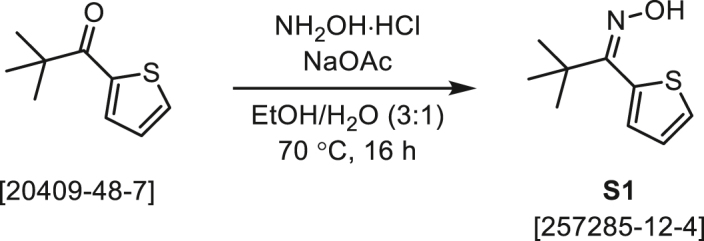

According to General Procedure A, 2-pivaloylthiophene (1.00 g, 5.94 mmol) was combined with NH_2_OH·HCl (2.48 g, 35.7 mmol) and NaOAc (488 mg, 5.94 mmol) in EtOH (45 mL) and H_2_O (15 mL) and heated to 70°C overnight. After stirring for 16 h, the reaction mixture was cooled to rt, diluted with H_2_O (10 mL), and extracted with EtOAc (3 × 35 mL). The combined extracts were washed with H_2_O (25 mL) and satd NaCl (25 mL), and concentrated under reduced pressure. The crude product was subjected to flash chromatography (0→20% EtOAc/Hex) and the oxime S1 was isolated as a white powder (986.7 mg, 5.38 mmol, 91%). *R*_f_ = 0.54 (20% EtOAc/Hex). ^1^H NMR (400 MHz, CDCl_3_) δ 8.30–7.58 (brs, 1H), 7.47 (dd, *J* = 4.7, 1.5 Hz, 1H), 7.12 (dd, *J* = 4.7, 3.6 Hz, 1H), 7.10 (dd, *J* = 3.6, 1.5 Hz, 1H), 1.25 (s, 9H). ^13^C NMR (101 MHz, CDCl_3_) δ 160.2, 130.8, 128.1, 127.0, 126.5, 38.0, 28.6 (3C). LCMS (ESI) *m/z*: 184.0791 calcd for C_9_H_14_NOS^+^ [M + H]^+^; Found 184.0781.

###### (*Z*)-2-Pivaloylthiophene *O*-(4-methoxybenzoyl) oxime (1, MAC-0452936)

**Figure:**
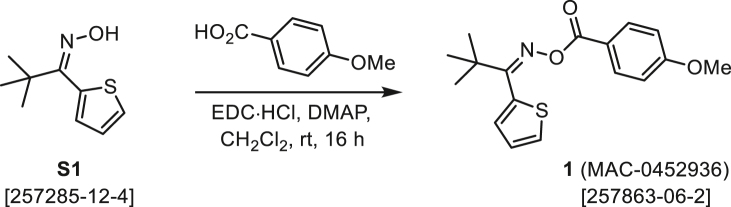

According to General Procedure B, the oxime S1 (400.0 mg, 2.183 mmol) was added at rt to a stirring solution of 4-methoxybenzoic acid (332.0 mg, 2.183 mmol), EDC·HCl (1.046 g, 5.456 mmol), and DMAP (26.7 mg, 0.218 mmol) in CH_2_Cl_2_ (3 mL). After 16 h, the reaction mixture was diluted with CH_2_Cl_2_ (25 mL), washed with satd Na_2_CO_3_ (15 mL) and satd NaCl (15 mL), dried over Na_2_SO_4_, and concentrated under reduced pressure. The crude product was subjected flash chromatography on silica gel (0→15% EtOAc/Hex) to provide MAC-0452936 (1) as a white solid (526.5 mg, 1.659 mmol, 76%). *R*_f_ = 0.50 (20% EtOAc/Hex). ^1^H NMR (400 MHz, CDCl_3_) δ 7.71 (AA′ of AA′XX′, *J* = 8.9 Hz, 2H), 7.49 (dd, *J* = 5.0, 1.2 Hz, 1H), 7.13 (dd, *J* = 5.0, 3.6 Hz, 1H), 7.05 (dd, *J* = 3.6, 1.2 Hz, 1H), 6.83 (XX′ of AA′XX′, *J* = 8.9 Hz, 2H), 3.83 (s, 3H), 1.36 (s, 9H). ^13^C NMR (101 MHz, CDCl_3_) δ 168.7, 163.6, 163.5, 131.8 (2C), 131.0, 127.8, 127.0, 126.5, 121.4, 113.8 (2C), 55.5, 28.4 (3C). HRMS (ESI) *m/z*: 340.0978 calcd for C_17_H_19_NO_3_SNa^+^ [M + Na]^+^; Found 340.0978.

###### (*Z*)-2-Pivaloylthiophene *O*-(2-fluoro-4-methoxybenzoyl) oxime (2)

**Figure:**
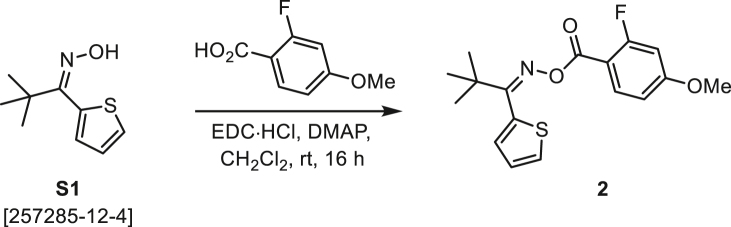

According to General Procedure B, the oxime S1 (50.0 mg, 0.273 mmol) was added at rt to a stirring solution of 2-fluoro-4-methoxybenzoic acid (46.4 mg, 0.273 mmol), EDC·HCl (130.3 mg, 0.682 mmol), and DMAP (3.3 mg, 0.027 mmol) in CH_2_Cl_2_ (3 mL). After 16 h, the reaction mixture was diluted with CH_2_Cl_2_ (25 mL), washed with satd Na_2_CO_3_ (15 mL) and satd NaCl (15 mL), dried over Na_2_SO_4_, and concentrated under reduced pressure. The crude product was subjected flash chromatography on silica gel (0→20% EtOAc/Hex) to provide oxime ester 2 as a white solid (41.8 mg, 0.125 mmol, 46%). *R*_f_ = 0.41 (20% EtOAc/Hex). ^1^H NMR (400 MHz, CDCl_3_) δ 7.63 (t, *J* = 8.6 Hz, ^4^*J*_HF_ = 8.6 Hz, 1H), 7.45 (dd, *J* = 5.0, 1.2 Hz, 1H), 7.10 (dd, *J* = 5.0, 3.6 Hz, 1H), 7.04 (dd, *J* = 3.6, 1.2 Hz, 1H), 6.63 (dd, *J* = 8.8, 2.5 Hz, 1H), 6.55 (dd, *J* = 2.4 Hz, ^3^*J*_HF_ = 12.4 Hz, 1H), 3.81 (s, 3H), 1.35 (s, 9H). ^13^C NMR (101 MHz, CDCl_3_) δ 169.3, 164.9, 163.6 (d, ^1^*J*_CF_ = 261.5 Hz), 161.5 (d, ^3^*J*_CF_ = 4.5 Hz), 133.4 (d, ^3^*J*_CF_ = 2.7 Hz), 130.8, 128.0, 126.9, 126.5, 110.3 (d, ^4^*J*_CF_ = 3.0 Hz), 102.5 (d, ^2^*J*_CF_ = 25.6 Hz), 55.9, 39.1, 28.4. A quaternary carbon expected between 105 and 115 ppm with ^2^*J*_CF_ ∼10–25 Hz was not detected in the ^13^C NMR spectrum. ^19^F NMR (377 MHz, CDCl_3_) δ −105.2. HRMS(ESI) *m/z:* 358.0884 calcd for C_17_H_18_FNO_3_SNa^+^ [M + Na]^+^; Found 358.0881.

###### (*Z*)-2-Pivaloylthiophene *O*-(pyridine-3-oyl) oxime (3)

**Figure:**
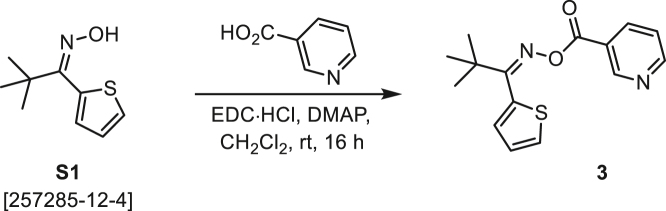

According to General Procedure B, the oxime S1 (50 mg, 0.273 mmol) was added at rt to a stirring solution of nicotinic acid (33.6 mg, 0.273 mmol), EDC·HCl (130 mg, 0.680 mmol), and DMAP (3.3 mg, 0.027 mmol) in CH_2_Cl_2_ (3 mL). After 16 h, the reaction mixture was diluted with CH_2_Cl_2_ (25 mL), washed with satd Na_2_CO_3_ (15 mL) and satd NaCl (15 mL), dried over Na_2_SO_4_, and concentrated under reduced pressure. The crude product was subjected flash chromatography on silica gel (0→40% EtOAc/Hex) to provide the pyridyl oxime ester **3** as a white solid (41.8 mg, 0.145 mmol, 53%). *R*_f_ = 0.15 (20% EtOAc/Hex). ^1^H NMR (400 MHz, CDCl_3_) δ 8.86 (brd, *J* = 1.4 Hz, 1H), 8.72 (dd, *J* = 4.9, 1.8 Hz, 1H), 8.07 (dt, *J* = 8.0, 1.9 Hz, 1H), 7.50 (dd, *J* = 5.1, 1.2 Hz, 1H), 7.32 (ddd, *J* = 8.0, 4.9, 1.0 Hz, 1H), 7.13 (dd, *J* = 5.1, 3.6 Hz, 1H), 7.05 (dd, *J* = 3.5, 1.2 Hz, 1H), 1.36 (s, 9H). ^13^C NMR (101 MHz, CDCl_3_) δ 170.1, 162.5, 153.7, 150.9, 137.3, 130.4, 127.9, 127.3, 126.7, 125.2, 123.5, 39.2, 28.3 (3C). HRMS(ESI) *m/z:* 311.0825 calcd for C_15_H_16_N_2_O_2_SNa^+^ [M + Na]^+^; Found 311.0827.

###### (*Z*)-2-Pivaloylthiophene *O*-(thiazol-5-ylcarbonyl) oxime (4)

**Figure:**
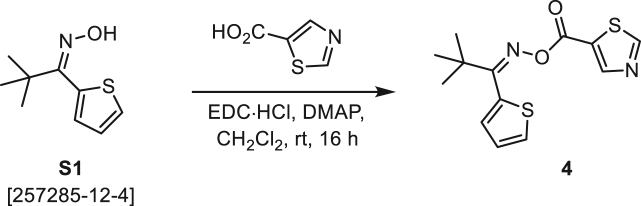

According to General Procedure B, the oxime S1 (50 mg, 0.273 mmol) was added at rt to a stirring solution of thiazole-5-carboxylic acid (35.2 mg, 0.273 mmol), EDC·HCl (130.3 mg, 0.682 mmol), and DMAP (3.3 mg, 0.027 mmol) in CH_2_Cl_2_ (3 mL). After 16 h, the reaction mixture was diluted with CH_2_Cl_2_ (25 mL), washed with satd Na_2_CO_3_ (15 mL) and satd NaCl (15 mL), dried over Na_2_SO_4_, and concentrated under reduced pressure. The crude product was subjected flash chromatography on silica gel (0→20% EtOAc/Hex) to provide oxime ester product **4** as a white solid (54.5 mg, 0.185 mmol, 68%). *R*_f_ = 0.33 (20% EtOAc/Hex). ^1^H NMR (400 MHz, CDCl_3_) δ 8.90 (s, 1H), 8.28 (s, 1H), 7.51 (dd, *J* = 5.0, 1.1 Hz, 1H), 7.14 (dd, *J* = 5.0, 3.6 Hz, 1H), 7.06 (dd, *J* = 3.6, 1.1 Hz, 1H), 1.36 (s, 9H). ^13^C NMR (101 MHz, CDCl_3_) δ 169.9, 158.6, 158.4, 149.4, 130.3, 128.1, 127.3, 126.7, 124.8 (br), 39.3, 28.3 (3C). HRMS(ESI) *m/z:* 317.0389 calcd for C_13_H_14_N_2_O_2_S_2_Na^+^ [M + Na]^+^; Found 317.0389.

###### (*Z*)-2-Pivaloylthiophene *O*-(1-methyl-1*H*-imidazol-5-oyl) oxime (5)

**Figure:**
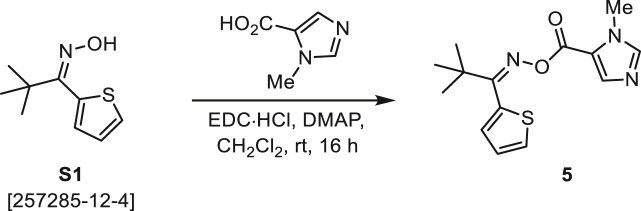

According to General Procedure B, the oxime S1 (50 mg, 0.273 mmol) was added at rt to a stirring solution of 1-methyl-1*H*-imidazole-5-carboxylic acid (34.4 mg, 0.273 mmol), EDC·HCl (130.3 mg, 0.682 mmol), and DMAP (3.3 mg, 0.027 mmol) in CH_2_Cl_2_ (3 mL). After 16 h, the reaction mixture was diluted with CH_2_Cl_2_ (25 mL), washed with satd Na_2_CO_3_ (15 mL) and satd NaCl (15 mL), dried over Na_2_SO_4_, and concentrated under reduced pressure. The crude product was subjected flash chromatography on silica gel (0→100% EtOAc/Hex) to provide the thiazolyl oxime ester **5** as a white solid (65.8 mg, 0.226 mmol, 83%). *R*_f_ = 0.32 (100% EtOAc). ^1^H NMR (400 MHz, CDCl_3_) δ 7.69 (s, 1H), 7.50 (dd, *J* = 5.1, 1.2 Hz, 1H), 7.34 (s, 1H), 7.12 (dd, *J* = 5.1, 3.6 Hz, 1H), 7.05 (dd, *J* = 3.6, 1.2 Hz, 1H), 3.87 (s, 3H), 1.35 (s, 9H). ^13^C NMR (101 MHz, CDCl_3_) δ 169.0, 157.7, 142.8, 137.5, 130.5, 128.0, 127.3, 126.6, 121.5 (br), 39.1, 34.2, 28.3 (3C). HRMS(ESI) *m/z:* 314.0934 calcd for C_14_H_17_N_3_O_2_SNa^+^ [M + Na]^+^; Found 314.0935.

###### (*Z*)-2-Pivaloylthiophene *O*-(3-amino-4-methoxybenzoyl) oxime (6)

**Figure:**
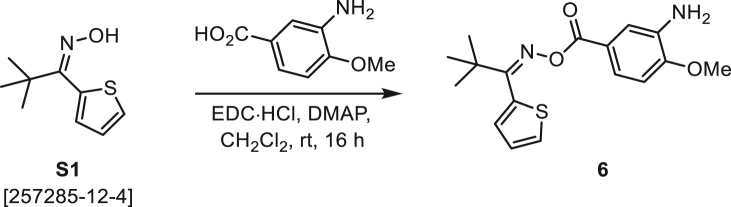

According to a modified General Procedure B, the oxime S1 (150 mg, 0.818 mmol) was added at rt to a stirring solution of 3-amino-4-methoxybenzoic acid (45.6 mg, 0.273 mmol), EDC·HCl (130.3 mg, 0.682 mmol), and DMAP (3.3 mg, 0.027 mmol) in CH_2_Cl_2_ (3 mL). After 16 h, the reaction mixture was diluted with CH_2_Cl_2_ (25 mL), washed with satd Na_2_CO_3_ (15 mL) and satd NaCl (15 mL), dried over Na_2_SO_4_, and concentrated under reduced pressure. The crude product was subjected flash chromatography on silica gel (0→40% EtOAc/Hex) to provide oxime ester **6** as a yellow solid (49.8 mg, 0.150 mmol, 55%). *R*_f_ = 0.16 (20% EtOAc/Hex). ^1^H NMR (400 MHz, CDCl_3_) δ 7.48 (dd, *J* = 5.0, 1.2 Hz, 1H), 7.17 (dd, *J* = 8.4, 2.1 Hz, 1H), 7.13 (d, *J* = 2.0 Hz, 1H), 7.12 (dd, *J* = 5.0, 3.5 Hz, 1H), 7.05 (dd, *J* = 3.5, 1.2 Hz, 1H), 6.70 (d, *J* = 8.4 Hz, 1H), 3.86 (s, 3H), 4.02–3.84 (brs, 2H), 1.36 (s, 9H). ^13^C NMR (101 MHz, CDCl_3_) δ 168.5, 163.9, 151.3, 135.9, 131.1, 127.9, 126.9, 126.5, 121.6, 121.4, 115.9, 109.6, 55.7, 39.0, 28.4 (3C). HRMS(ESI) *m/z:* 355.1087 calcd for C_17_H_20_N_2_O_3_SNa^+^ [M + Na]^+^; Found 355.1087.

###### (*Z*)-2-Pivaloylthiophene *O*-(3,5-dimethoxybenzoyl) oxime (7)

**Figure:**
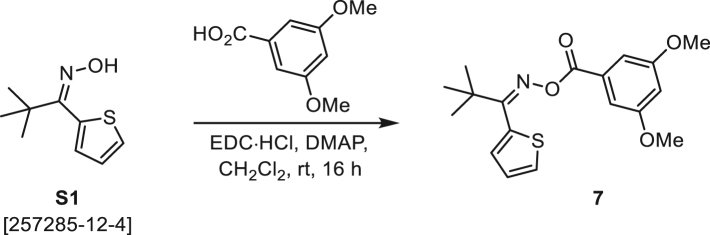

According to General Procedure B, the oxime S1 (50 mg, 0.273 mmol) was added at rt to a stirring solution of 3,5-dimethoxybenzoic acid (49.7 mg, 0.273 mmol), EDC·HCl (130.3 mg, 0.682 mmol), and DMAP (3.3 mg, 0.027 mmol) in CH_2_Cl_2_ (3 mL). After 16 h, the reaction mixture was diluted with CH_2_Cl_2_ (25 mL), washed with satd Na_2_CO_3_ (15 mL) and satd NaCl (15 mL), dried over Na_2_SO_4_, and concentrated under reduced pressure. The crude product was subjected flash chromatography on silica gel (0→20% EtOAc/Hex) to provide oxime ester **7** as a white solid (54.2 mg, 0.156 mmol, 57%). *R*_f_ = 0.41 (20% EtOAc/Hex). ^1^H NMR (400 MHz, CDCl_3_) δ 7.48 (dd, *J* = 5.0, 1.2 Hz, 1H), 7.12 (dd, *J* = 5.0, 3.6 Hz, 1H), 7.05 (dd, *J* = 3.6, 1.2 Hz, 1H), 6.89 (d, *J* = 2.4 Hz, 2H), 6.59 (t, *J* = 2.4 Hz, 1H), 3.72 (s, 6H), 1.37 (s, 9H). ^13^C NMR (101 MHz, CDCl_3_) δ 169.4, 163.3, 160.7 (2C), 131.1, 130.9, 127.8, 126.8, 126.6, 107.1 (2C), 106.5, 55.6 (2C), 39.1, 28.3 (3C). HRMS(ESI) *m/z:* 370.1084 calcd for C_18_H_21_NO_4_SNa^+^ [M + Na]^+^; Found 370.1098.

###### (*Z*)-2-Pivaloylthiophene *O*-(4-chlorobenzoyl) oxime (8)

**Figure:**
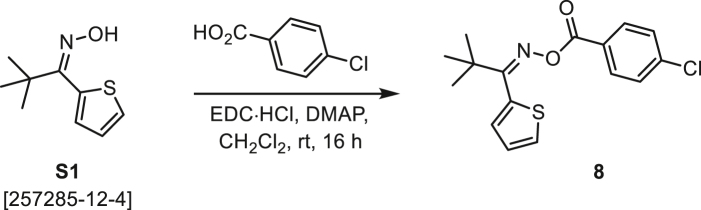

According to General Procedure B, the oxime S1 (50 mg, 0.273 mmol) was added at rt to a stirring solution of 4-chlorobenzoic acid (42.7 mg, 0.273 mmol), EDC·HCl (130.3 mg, 0.682 mmol), and DMAP (3.3 mg, 0.027 mmol) in CH_2_Cl_2_ (3 mL). After 16 h, the reaction mixture was diluted with CH_2_Cl_2_ (25 mL), washed with satd Na_2_CO_3_ (15 mL) and satd NaCl (15 mL), dried over Na_2_SO_4_, and concentrated under reduced pressure. The crude product was subjected flash chromatography on silica gel (0→15% EtOAc/Hex) to provide oxime ester **8** as a white solid (55.4 mg, 0.172 mmol, 63%). *R*_f_ = 0.61 (20% EtOAc/Hex). ^1^H NMR (400 MHz, CDCl_3_) δ 7.68 (appd, *J* = 8.6 Hz, 2H), 7.50 (dd, *J* = 5.0, 1.2 Hz, 1H), 7.33 (appd, *J* = 8.6 Hz, 2H), 7.13 (dd, *J* = 5.0, 3.6 Hz, 1H), 7.03 (dd, *J* = 3.6, 1.2 Hz, 1H), 1.36 (s, 9H). ^13^C NMR (101 MHz, CDCl_3_) δ 170.0, 162.9, 139.7, 131.1 (2C), 130.6, 128.9 (2C), 127.8, 127.5, 127.1, 126.6, 39.1, 28.3 (3C). HRMS(ESI) *m/z:* 344.0483 calcd for C_16_H_16_ClNO_2_SNa^+^ [M + Na]^+^; Found 344.0481.

###### (*Z*)-2-Pivaloylthiophene *O*-(4-trifluoromethylbenzoyl) oxime (9)

**Figure:**
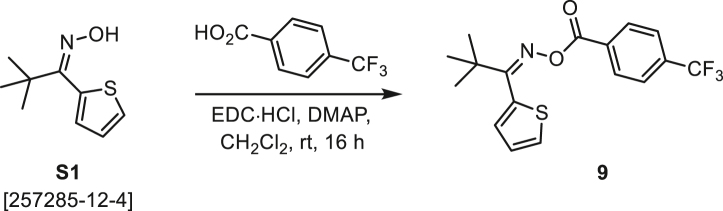

According to General Procedure B, the oxime S1 (50 mg, 0.273 mmol) was added at rt to a stirring solution of 4-(trifluoromethyl)benzoic acid (51.9 mg, 0.273 mmol), EDC·HCl (130.3 mg, 0.682 mmol), and DMAP (3.3 mg, 0.027 mmol) in CH_2_Cl_2_ (3 mL). After 16 h, the reaction mixture was diluted with CH_2_Cl_2_ (25 mL), washed with satd Na_2_CO_3_ (15 mL) and satd NaCl (15 mL), dried over Na_2_SO_4_, and concentrated under reduced pressure. The crude product was subjected flash chromatography on silica gel (0→10% EtOAc/Hex) to provide oxime ester **9** as a white solid (71.0 mg, 0.200 mmol, 73%). *R*_f_ = 0.68 (20% EtOAc/Hex). ^1^H NMR (400 MHz, CDCl_3_) δ 7.85 (d, *J* = 8.1 Hz, 2H), 7.63 (d, *J* = 8.1 Hz, 2H), 7.51 (dd, *J* = 5.0, 1.2 Hz, 1H), 7.14 (dd, *J* = 5.0, 3.6 Hz, 1H), 7.05 (dd, *J* = 3.6, 1.2 Hz, 1H), 1.37 (s, 9H). ^13^C NMR (101 MHz, CDCl_3_) δ 170.1, 162.5, 134.6 (q, ^2^*J*_CF_ = 32.7 Hz, 2C), 132.4 (q, ^5^*J*_CF_ = 1.5 Hz), 130.5, 130.1 (2C), 127.9, 127.2, 126.7, 125.6 (q, ^3^*J*_CF_ = 3.7 Hz, 2C), 123.7 (q, ^1^*J*_CF_ = 272.8 Hz), 39.2, 28.3 (3C). ^19^F NMR (377 MHz, CDCl_3_) δ −63.74. HRMS (ESI) *m/z:* 378.0746 calcd for C_17_H_16_F_3_NO_2_SNa^+^ [M + Na]^+^; Found 378.0745.

###### (*Z*)-2-Pivaloylthiophene *O*-(4-methylaminobenzoyl) oxime (10)

**Figure:**
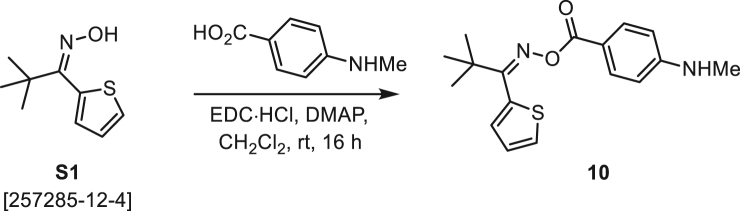

According to a modified General Procedure B, the oxime S1 (150 mg, 0.818 mmol) was added at rt to a stirring solution of 4-(methylamino)benzoic acid (41.2 mg, 0.273 mmol), EDC·HCl (130.3 mg, 0.682 mmol), and DMAP (3.3 mg, 0.027 mmol) in CH_2_Cl_2_ (3 mL). After 16 h, the reaction mixture was diluted with CH_2_Cl_2_ (25 mL), washed with satd Na_2_CO_3_ (15 mL) and satd NaCl (15 mL), dried over Na_2_SO_4_, and concentrated under reduced pressure. The crude product was subjected flash chromatography on silica gel (0→30% EtOAc/Hex) to provide oxime ester 10 as a yellow solid (33.9 mg, 0.107 mmol, 39%). *R*_f_ = 0.23 (20% EtOAc/Hex). ^1^H NMR (400 MHz, CDCl_3_) δ 7.60 (appd, *J* = 8.8 Hz, 2H), 7.47 (dd, *J* = 5.1, 1.2 Hz, 1H), 7.11 (dd, *J* = 5.1, 3.6 Hz, 1H), 7.05 (dd, *J* = 3.6, 1.2 Hz, 1H), 6.46 (appd, *J* = 8.8 Hz, 2H), 4.15 (brs, 1H), 2.86 (s, 3H), 1.36 (s, 9H). ^13^C NMR (101 MHz, CDCl_3_) δ 167.9, 164.1, 153.1, 131.8 (2C), 131.2, 127.8, 126.8, 126.5, 116.7, 111.2 (2C), 39.0, 30.2, 28.4 (3C). HRMS (ESI) *m/z:* 339.1138 calcd for C_17_H_20_N_2_O_2_SNa^+^ [M + Na]^+^; Found 339.1138.

###### (*Z*)-2-Pivaloylthiophene *O*-benzoyl oxime (11)

**Figure:**
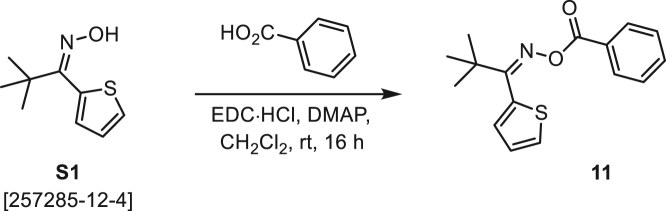

According to General Procedure B, the oxime S1 (50 mg, 0.273 mmol) was added at rt to a stirring solution of benzoic acid (33.3 mg, 0.273 mmol), EDC·HCl (130.3 mg, 0.682 mmol), and DMAP (3.3 mg, 0.027 mmol) in CH_2_Cl_2_ (3 mL). After 16 h, the reaction mixture was diluted with CH_2_Cl_2_ (20 mL), washed with satd Na_2_CO_3_ (20 mL) and satd NaCl (20 mL), dried over Na_2_SO_4_, and concentrated under reduced pressure. The crude product was subjected flash chromatography on silica gel (0→10% EtOAc/Hex) to provide oxime ester 11 as a white solid (41.8 mg, 0.145 mmol, 53%). *R*_f_ = 0.34 (10% EtOAc/Hex). ^1^H NMR (400 MHz, CDCl_3_) δ 7.76 (dd, *J* = 8.4, 1.4 Hz, 2H), 7.51 (app tt, *J* = 7.8, 1.4 Hz, 1H), 7.50 (dd, *J* = 5.0, 1.2 Hz, 1H), 7.36 (appt, *J* = 7.8 Hz, 2H), 7.13 (dd, *J* = 5.1, 3.6 Hz, 1H), 7.06 (dd, *J* = 3.6, 1.2 Hz, 1H), 1.37 (s, 9H).^13^C NMR (101 MHz, CDCl_3_) δ 169.2, 163.8, 133.2, 130.8, 129.7 (2C), 129.1, 128.5 (2C), 127.9, 127.0, 126.6, 39.1, 28.3 (3C). HRMS (ESI) *m/z:* 310.0872 calcd for C_16_H_17_NO_2_SNa^+^ [M + Na]^+^; Found 310.0872.

###### (*Z*)-2-Pivaloylthiophene *O*-picolinoyl oxime (12)

**Figure:**
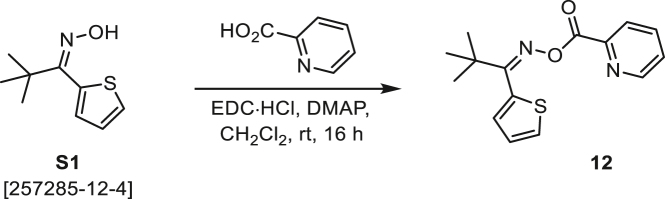

According to General Procedure B, the oxime S1 (50 mg, 0.273 mmol) was added at rt to a stirring solution of picolinic acid (33.6 mg, 0.273 mmol), EDC·HCl (130.3 mg, 0.682 mmol), and DMAP (3.3 mg, 0.027 mmol) in CH_2_Cl_2_ (3 mL). After 16 h, the reaction mixture was diluted with CH_2_Cl_2_ (25 mL), washed with satd Na_2_CO_3_ (15 mL) and satd NaCl (15 mL), dried over Na_2_SO_4_, and concentrated under reduced pressure. The crude product was subjected flash chromatography on silica gel (0→40% EtOAc/Hex) to provide oxime ester **12** as a white solid (19.1 mg, 0.0662 mmol, 24%). *R*_f_ = 0.13 (20% EtOAc/Hex). ^1^H NMR (400 MHz, CDCl_3_) δ 8.73 (ddd, *J* = 4.7, 1.7, 1.0 Hz, 1H), 7.72 (td, *J* = 7.6, 1.7 Hz, 1H), 7.67 (ddd, *J* = 7.7, 1.5, 1.0 Hz, 1H), 7.47 (dd, *J* = 4.9, 1.3 Hz, 1H), 7.41 (ddd, *J* = 7.3, 4.7, 1.5 Hz, 1H), 7.14 (dd, *J* = 3.6, 1.3 Hz, 1H), 7.11 (dd, *J* = 4.9, 3.6 Hz, 1H), 1.38 (s, 9H). ^13^C NMR (101 MHz, CDCl_3_) δ 170.2, 162.1, 150.3, 147.4, 137.0, 130.6, 128.4, 127.3, 126.9, 126.6, 125.2, 39.2, 28.4 (3C). HRMS (ESI) *m/z*: 311.0825 calcd for C_15_H_16_N_2_O_2_SNa^+^ [M + Na]^+^; Found 311.0825.

###### (*Z*)-2-Pivaloylthiophene *O*-(6-methoxynicotinoyl) oxime (13)

**Figure:**
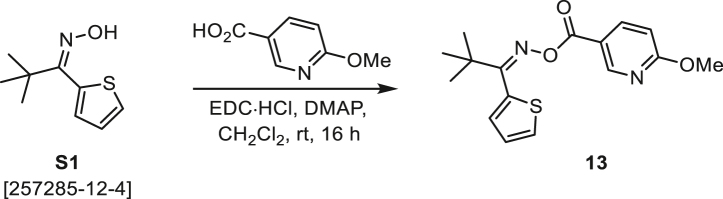

According to General Procedure B, the oxime S1 (50 mg, 0.273 mmol) was added at rt to a stirring solution of 6-methoxynicotinic acid (41.8 mg, 0.273 mmol), EDC·HCl (130.3 mg, 0.682 mmol), and DMAP (3.3 mg, 0.027 mmol) in CH_2_Cl_2_ (3 mL). After 16 h, the reaction mixture was diluted with CH_2_Cl_2_ (25 mL), washed with satd Na_2_CO_3_ (15 mL) and satd NaCl (15 mL), dried over Na_2_SO_4_, and concentrated under reduced pressure. The crude product was subjected flash chromatography on silica gel (0→15% EtOAc/Hex) to provide oxime ester **13** as a white solid (28.8 mg, 0.090 mmol, 33%). *R*_f_ = 0.35 (20% EtOAc/Hex). ^1^H NMR (400 MHz, CDCl_3_) δ 8.51 (d, *J* = 2.4 Hz, 1H), 7.92 (dd, *J* = 8.7, 2.4 Hz, 1H), 7.50 (dd, *J* = 5.0, 1.2 Hz, 1H), 7.13 (dd, *J* = 5.0, 3.6 Hz, 1H), 7.05 (dd, *J* = 3.6, 1.2 Hz, 1H), 6.70 (d, *J* = 8.7 Hz, 1H), 3.96 (s, 3H), 1.36 (s, 9H). ^13^C NMR (101 MHz, CDCl_3_) δ 169.3, 167.0, 162.7, 150.1, 139.7, 130.7, 127.8, 127.2, 126.7, 118.6, 111.0, 54.2, 39.2, 28.4 (3C). HRMS (ESI) *m/z*: 341.0930 calcd for C_16_H_18_N_2_O_3_SNa^+^ [M + Na]^+^; Found 341.0930.

###### (*Z*)-2-Pivaloylthiophene *O*-(quinolin-3-oyl) oxime (14)

**Figure:**
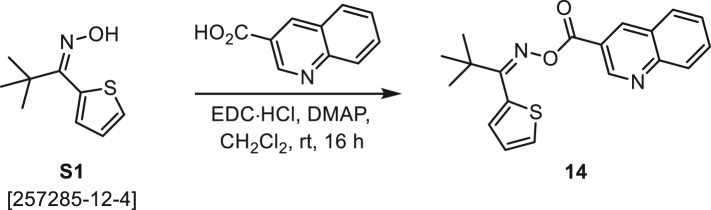

According to General Procedure B, the oxime S1 (50 mg, 0.273 mmol) was added at rt to a stirring solution of quinoline-3-carboxylic acid (41.8 mg, 0.273 mmol), EDC·HCl (130.3 mg, 0.682 mmol), and DMAP (3.3 mg, 0.027 mmol) in CH_2_Cl_2_ (3 mL). After 16 h, the reaction mixture was diluted with CH_2_Cl_2_ (25 mL), washed with satd Na_2_CO_3_ (15 mL) and satd NaCl (15 mL), dried over Na_2_SO_4_, and concentrated under reduced pressure. The crude product was subjected flash chromatography on silica gel (0→20% EtOAc/Hex) to provide oxime ester **14** as a white solid (53.0 mg, 0.156 mmol, 57%). *R*_f_ = 0.33 (20% EtOAc/Hex). ^1^H NMR (400 MHz, CDCl_3_) δ 9.07 (d, *J* = 2.1 Hz, 1H), 8.64 (d, *J* = 2.1 Hz, 1H), 8.13 (d, *J* = 8.5 Hz, 1H), 7.87 (d, *J* = 8.3 Hz, 1H), 7.83 (appt, *J* = 7.8 Hz, 1H), 7.61 (appt, *J* = 7.5 Hz, 1H), 7.54 (dd, *J* = 5.1, 1.2 Hz, 1H), 7.17 (dd, *J* = 5.1, 3.6 Hz, 1H), 7.10 (dd, *J* = 3.6, 1.2 Hz, 1H), 1.40 (s, 9H). ^13^C NMR (101 MHz, CDCl_3_) δ 170.2, 162.5, 149.9, 149.7, 139.4, 132.2, 130.6, 129.6, 129.3, 127.9, 127.6, 127.3, 127.0, 126.8, 122.2, 39.3, 28.3 (3C). HRMS (ESI) *m/z:* 361.0981 calcd for C_19_H_18_N_2_O_2_SNa^+^ [M + Na]^+^; Found 361.0982.

###### (*Z*)-2-Pivaloylthiophene *O*-(isoxazol-5-ylcarbonyl) oxime (15)

**Figure:**
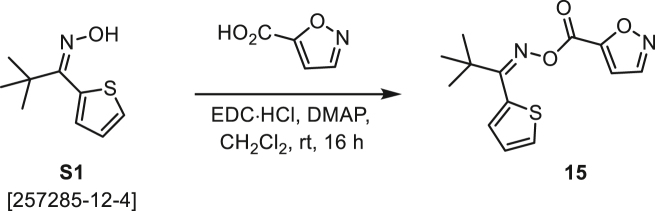

According to General Procedure B, the oxime S1 (50 mg, 0.273mmol) was added at rt to a stirring solution of isoxazole-5-carboxylic acid (30.9 mg, 0.273 mmol), EDC·HCl (130.3 mg, 0.682 mmol), and DMAP (3.3 mg, 0.027 mmol) in CH_2_Cl_2_ (3 mL). After 16 h, the reaction mixture was diluted with CH_2_Cl_2_ (25 mL), washed with satd Na_2_CO_3_ (15 mL) and satd NaCl (15 mL), dried over Na_2_SO_4_, and concentrated under reduced pressure. The crude product was subjected flash chromatography on silica gel (0→20% EtOAc/Hex) to provide oxime ester **15** as a white solid (57.5 mg, 0.207 mmol, 76%). *R*_f_ = 0.38 (20% EtOAc/Hex). ^1^H NMR (400 MHz, CDCl_3_) δ 8.30 (d, *J* = 1.8 Hz, 1H), 7.51 (dd, *J* = 4.7, 1.5 Hz, 1H), 7.13 (dd, *J* = 4.7, 3.6 Hz, 1H), 7.11 (dd, *J* = 3.6, 1.5 Hz, 1H), 6.69 (d, *J* = 1.8 Hz, 1H), 1.37 (s, 9H). ^13^C NMR (101 MHz, CDCl_3_) δ 170.8, 158.7, 154.0, 150.7, 129.9, 128.6, 127.7, 126.7, 109.2, 39.5, 28.4 (3C). HRMS (ESI) *m/z*: 301.0617 calcd for C_13_H_14_N_2_O_3_SNa^+^ [M + Na]^+^; Found 301.0619.

###### (*Z*)-2-Pivaloylthiophene *O*-(2-(4-methoxyphenyl)acetyl) oxime (16)

**Figure:**
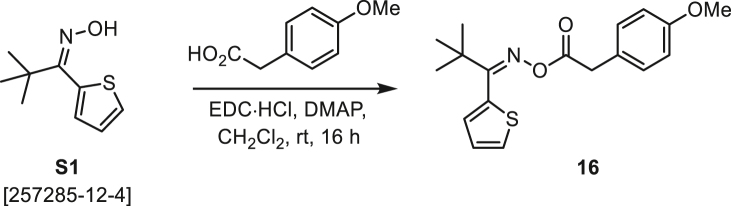

According to General Procedure B, the oxime S1 (50 mg, 0.273mmol) was added at rt to a stirring solution of 2-(4-methoxyphenyl)acetic acid (45.3 mg, 0.273 mmol), EDC·HCl (130.3 mg, 0.682 mmol), and DMAP (3.3 mg, 0.027 mmol) in CH_2_Cl_2_ (3 mL). After 16 h, the reaction mixture was diluted with CH_2_Cl_2_ (20 mL), washed with satd Na_2_CO_3_ (20 mL) and satd NaCl (20 mL), dried over Na_2_SO_4_, and concentrated under reduced pressure. The crude product was subjected flash chromatography on silica gel (0→10% EtOAc/Hex) to provide oxime ester **16** as a yellow oil (72.3 mg, 0.218 mmol, 80%). *R*_f_ = 0.19 (10% EtOAc/Hex). ^1^H NMR (400 MHz, CDCl_3_) δ 7.43 (dd, *J* = 5.1, 1.1 Hz, 1H), 7.05 (dd, *J* = 5.1, 3.6 Hz, 1H), 7.02 (appd, *J* = 8.6 Hz, 2H), 6.91 (dd, *J* = 3.6, 1.1 Hz, 1H), 6.77 (appd, *J* = 8.6 Hz, 2H), 3.79 (s, 3H), 3.52 (s, 2H), 1.29 (s, 9H). ^13^C NMR (101 MHz, CDCl_3_) δ 169.0, 168.8, 158.8, 130.5 (2C), 130.4, 128.0, 127.0, 126.4, 125.4, 114.0 (2C), 55.4, 39.6, 39.0, 28.3 (3C). HRMS (ESI) *m/z:* 354.1134 calcd for C_18_H_21_NO_3_SNa^+^ [M + Na]^+^; Found 354.1134.

###### 2-Pivaloylthiophene *O*-(sulfamoyl) oxime (17)

**Figure:**
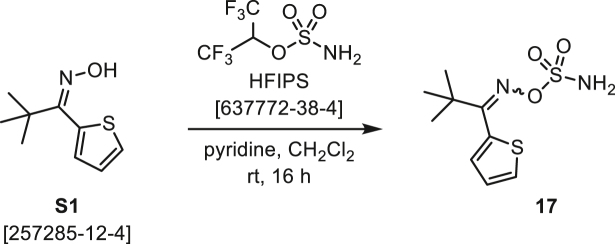

According to a procedure described by Sguazzin et al.,^84^ hexafluoroisopropyl sulfamate (HFIPS, 162 mg, 0.655 mmol) was added to a solution of oxime S1 (100 mg, 0.546 mmol) in CH_2_Cl_2_ (3.8 mL) and pyridine (1.6 mL) and stirred at rt for 16 h. The solvent was then removed by rotary evaporation and the removal of residual pyridine was aided by co-evaporation with PhMe (15 mL). The crude product was subjected flash chromatography on silica gel (0→50% EtOAc/Hex) to provide sulfamate **17** as a white powder (9.3 mg, 7%). Note: The low isolated yield for **17** is attributed to poor stability of the oxime sulfamate on silica gel. *R*_f_ = 0.17 (20% EtOAc/Hex). ^1^H NMR (400 MHz, CDCl_3_) δ 7.63 (s, 1H), 7.28 (dd, *J* = 5.1, 1.2, 1H), 7.25 (dd, *J* = 3.7, 1.2, 1H), 7.03 (dd, *J* = 5.1, 3.7 Hz, 1H), 2.31 (s, 3H). ^13^C NMR (101 MHz, CDCl_3_) δ 152.0, 140.3, 127.3, 127.0, 126.6, 12.4. HRMS (ESI) *m*/*z*: 184.0791 calcd for C_9_H_14_NOS^+^ [M – SO_2_NH + H] ^+^; Found 184.0786.

###### 2-Acetylthiophene *O*-(4-methoxybenzoyl) oximes (*E*)-18 and (*Z*)-18

**Figure:**
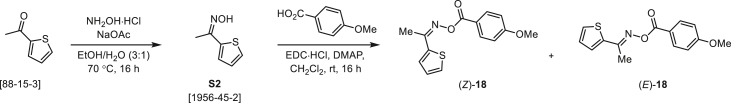

According to General Procedure A, 2-acetylthiophene (126.2 mg, 1.00 mmol) was combined with NH_2_OH·HCl (417 g, 6.00 mmol) and NaOAc (82.0 mg, 1.00 mmol) in EtOH (10 mL) and H_2_O (3 mL) and heated to 70°C overnight. After stirring for 16 h, the reaction mixture was cooled to rt, diluted with H_2_O (20 mL), and extracted with EtOAc (3 × 30 mL). The combined extracts were washed with H_2_O (50 mL) and satd NaCl (50 mL), and concentrated under reduced pressure. The crude product was subjected to flash chromatography (0→20% EtOAc/Hex) and 2-acetylthiophene oxime (**S2**) was isolated as a white solid (131.9 mg, 0.934 mmol, 93%) in a 62:38 mixture of undefined oxime stereoisomers. A small sample of purified **S2a** was isolated after additional chromatography, but the majority of the oxime was used as an *E*/*Z* mixture in the following step. HRMS (ESI) *m*/*z*: 142.0321 calcd for C_6_H_7_NOSNa^+^ [M + Na] ^+^; Found 142.0326. **Isomer S2a**. *R*_f_ = 0.25 (20% EtOAc/Hex). ^1^H NMR (400 MHz, CDCl_3_) δ 7.63 (s, 1H), 7.28 (dd, *J* = 5.1, 1.2, 1H), 7.25 (dd, *J* = 3.7, 1.2, 1H), 7.03 (dd, *J* = 5.1, 3.7 Hz, 1H), 2.31 (s, 3H). ^13^C NMR (101 MHz, CDCl_3_) δ 152.0, 140.3, 127.3, 127.0, 126.6, 12.4. **Isomer S2b**. *R*_f_ = 0.25 (20% EtOAc/Hex). ^1^H NMR (400 MHz, CDCl_3_) δ 9.55 (brs, 1H), 7.58 (dd, *J* = 5.1, 1.2, 1H), 7.53 (dd, *J* = 3.8, 1.2, 1H), 7.12 (dd, *J* = 5.1, 3.8 Hz, 1H), 2.41 (s, 3H). ^13^C NMR (101 MHz, CDCl_3_) δ 147.2, 132.2, 131.2, 130.0, 125.7, 12.6.

According to General Procedure B, a 62:38 mixture of oxime isomers **S2a** and **S2b** (118.0 mg, 0.831 mmol) was added at rt to a stirring solution of 4-methoxybenzoic acid (126.5 mg, 0.831 mmol), EDC·HCl (398.6 mg, 2.079 mmol), and DMAP (10.2 mg, 0.083 mmol) in CH_2_Cl_2_ (5 mL). After 16 h, the reaction mixture was diluted with CH_2_Cl_2_ (20 mL), washed with satd Na_2_CO_3_ (20 mL) and satd NaCl (20 mL), dried over Na_2_SO_4_, and concentrated under reduced pressure. The crude product contained a mixture of oxime isomers (*E*:*Z* 66:34), as indicated by ^1^H NMR. Flash chromatography on silica gel (0→20% EtOAc/Hex) provided oxime ester (*Z*)-**18** as a white solid (28.7 mg, 0.104 mmol, 13%) along with additional fractions of (*E*)-**18** mixed with (*Z*)-**18**. Stereochemistry of the oxime was assigned based on a single-crystal X-ray diffraction experiment with the more polar oxime isomer, (*Z*)-**18**. HRMS (ESI) *m/z:* 298.0508 calcd for C_14_H_13_NO_3_SNa^+^ [M + Na]^+^; Found 298.0509. **(*E*)-18**. *R*_f_ = 0.44 (40% EtOAc/Hex). ^1^H NMR (700 MHz, CDCl_3_) δ 8.10 (d, *J* = 8.9 Hz, 2H), 7.48 (dd, *J* = 3.7, 1.1 Hz, 1H), 7.44 (dd, *J* = 5.1, 1.1 Hz, 1H), 7.11 (dd, *J* = 5.1, 3.7 Hz, 1H), 6.99 (d, *J* = 8.9 Hz, 2H), 3.91 (s, 3H), 2.54 (s, 3H). ^13^C NMR (176 MHz, CDCl_3_) δ 163.8, 163.3, 158.7, 138.3, 132.5 (2C), 129.5, 129.2, 127.4, 121.4, 114.0 (2C), 55.6, 14.8. **(*Z*)-18**. *R*_f_ = 0.34 (40% EtOAc/Hex). ^1^H NMR (700 MHz, CDCl_3_) δ 8.21 (d, *J* = 8.7 Hz, 2H), 7.66 (dd, *J* = 5.1, 1.2 Hz, 1H), 7.59 (dd, *J* = 3.8, 1.2 Hz, 1H), 7.16 (dd, *J* = 5.1, 3.8 Hz, 1H), 7.00 (d, *J* = 8.7 Hz, 2H), 3.89 (s, 3H), 2.58 (s, 3H). ^13^C NMR (176 MHz, CDCl_3_) δ 164.1, 163.9, 154.9, 132.7, 132.5 (2C), 132.3, 131.8, 126.5, 121.2, 114.0 (2C), 55.6, 20.6.

###### 2-(Propan-1-oyl)thiophene *O*-(4-methoxybenzoyl) oximes (*E*)-19 and (*Z*)-19

**Figure:**
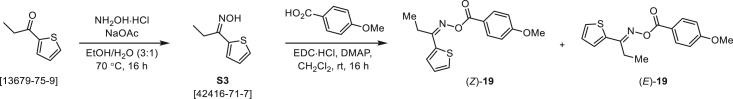

According to General Procedure A, 2-(propan-1-oyl)thiophene (140.2 mg, 1.00 mmol) was combined with NH_2_OH·HCl (417 mg, 6.00 mmol) and NaOAc (82.0 mg, 1.00 mmol) in EtOH (10 mL) and H_2_O (3 mL) and heated to 70°C overnight. After stirring for 16 h, the reaction mixture was cooled to rt, diluted with H_2_O (20 mL), and extracted with EtOAc (3 × 30 mL). The combined extracts were washed with H_2_O (50 mL) and satd NaCl (50 mL), and concentrated under reduced pressure. The crude reaction mixture contained a 57:43 mixture of isomeric oximes **S3a** and **S3b**, respectively, as indicated by ^1^H NMR. Flash chromatography (0→20% EtOAc/Hex) provided a mixture of **S3a** and **S3b** (115.4 mg, 0.743 mmol, 74%), which was used in the following reaction without further purification. A small amount of purified **S3b** was obtained following further chromatography. HRMS(ESI) *m/z:* 178.0297 calcd for C_7_H_9_NOSNa^+^ [M + Na]^+^; Found 178.0295. **Isomer S3a**. *R*_f_ = 0.55 (20% EtOAc/Hex). ^1^H NMR (400 MHz, CDCl_3_) δ 8.96, (brs, 1H), 7.29 (dd, *J* = 5.1, 1.0 Hz, 1H), 7.27 (dd, *J* = 3.9, 1.0 Hz, 1H), 7.04 (dd, *J* = 5.1, 3.9 Hz, 1H), 2.82 (q, *J* = 7.4 Hz, 2H), 1.31 (t, *J* = 7.4 Hz, 3H). **Isomer S3b**. *R*_f_ = 0.38 (20% EtOAc/Hex). ^1^H NMR (400 MHz, CD_2_Cl_2_) δ 7.60 (dd, *J* = 5.1, 1.2 Hz, 1H), 7.56 (dd, *J* = 3.9, 1.2 Hz, 1H), 7.14 (dd, *J* = 5.1, 3.9 Hz, 1H), 2.79 (q, *J* = 7.5 Hz, 2H), 1.27 (t, *J* = 7.5 Hz, 3H). The signal for the oxime O*H* was not detected. ^13^C NMR (101 MHz, CDCl_3_) δ 151.4, 131.7, 130.8, 129.6, 125.8, 27.3, 12.5.

According to General Procedure B, the oxime **S3** (50.0 mg, 0.322 mmol) was added at rt to a stirring solution of 4-methoxybenzoic acid (49.0 mg, 0.322 mmol), EDC·HCl (154.4 mg, 0.805 mmol), and DMAP (3.9 mg, 0.032 mmol) in CH_2_Cl_2_ (4 mL). After 16 h, the reaction mixture was diluted with CH_2_Cl_2_ (20 mL), washed with satd Na_2_CO_3_ (20 mL) and satd NaCl (20 mL), dried over Na_2_SO_4_, and concentrated under reduced pressure. The crude reaction mixture contained a mixture of isomeric oximes (*E*:*Z* ∼57:43), as indicated by ^1^H NMR. The stereochemistry for oxime isomers of **19** were assigned by relation of the chemical shifts to the *E*- and *Z*-isomers of **18**. For NMR assignments, see Data S5. Flash chromatography on silica gel (0→20% EtOAc/Hex) provided a stereoisomeric mixture of **19** (*E*:*Z* ∼67:33) as a white solid (22 mg, 0.076 mmol, 24%), and the mixture was used for biological assays. HRMS (ESI) *m/z:* 312.0665 calcd for C_15_H_15_NO_3_SNa^+^ [M + Na]^+^; Found 312.0669. (*E*)-**19**. *R*_f_ = 0.29 (20% EtOAc/Hex). ^1^H NMR (700 MHz, CDCl_3_) δ 8.06 (d, *J* = 8.8 Hz, 2H), 7.49 (d, *J* = 3.7 Hz, 1H), 7.44 (d, *J* = 5.1 Hz, 1H), 7.09 (dd, *J* = 5.1, 3.7 Hz, 1H), 6.97 (d, *J* = 8.9 Hz, 2H), 3.89 (s, 3H), 2.94 (q, *J* = 7.7 Hz, 2H), 1.34 (t, *J* = 7.7 Hz, 3H). ^13^C NMR (176 MHz, CDCl_3_) δ 163.8, 163.5, 163.4, 137.4, 132.3 (2C), 129.5, 129.0, 127.5, 121.5, 114.0 (2C), 55.6, 22.9, 12.0. (*Z*)-**19**. ^1^H NMR (700 MHz, CDCl_3_) δ 8.20 (d, *J* = 8.9 Hz, 2H), 7.65 (d, *J* = 5.1 Hz, 1H), 7.61 (d, *J* = 3.9 Hz, 1H), 7.17 (dd, *J* = 5.1, 3.9 Hz, 1H), 7.00 (d, *J* = 8.9 Hz, 2H), 3.89 (s, 3H), 2.97 (q, *J* = 7.5 Hz, 2H), 1.38 (t, *J* = 7.5 Hz, 3H). ^13^C NMR (176 MHz, CDCl_3_) δ 164.2, 163.8, 158.4, 132.5 (2C), 131.9, 131.8, 131.1, 126.6, 121.4, 114.1 (2C), 55.6, 28.2, 13.3.

###### (*Z*)-5-Pivaloylthiazole *O*-(4-methoxybenzoyl) oxime (20)

**Figure:**
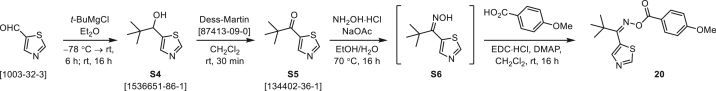

As an adaptation of a reported procedure,^79^ a solution of thiazole-5-carbaldehyde (300 mg, 2.65 mmol) in dry Et_2_O (30 mL) was degassed with argon and then cooled to −78°C. A solution of *t*-BuMgCl (3.18 mL, 1 M in THF, 3.18 mmol) was added dropwise to the stirring aldehyde at −78°C over 10 min. The reaction was allowed to warm to rt gradually over 6 h and left to stir at rt overnight before it was quenched with a slow addition of 5% HCl at 0°C (10 mL). The mixture was then diluted with Et_2_O (20 mL), washed with 1 M HCl (40 mL), satd NaCl (50 mL), dried over Na_2_SO_4_, and concentrated under reduced pressure. Flash chromatography on silica gel (0→50% EtOAc/Hex) provided alcohol **S4** as a yellow oil (62.1 mg, 0.363 mmol, 14%). *R*_f_ = 0.14 (20% EtOAc/Hex). ^1^H NMR (400 MHz, CDCl_3_) δ 8.72 (s, 1H), 7.71 (d, *J* = 0.7 Hz, 1H), 4.75 (d, *J* = 3.3 Hz, 1H), 2.43 (d, *J* = 3.5 Hz, 1H), 0.98 (s, 9H). ^13^C NMR (101 MHz, CDCl_3_) δ 152.69, 141.22, 140.27, 76.87, 35.78, 25.83. HRMS (ESI) *m/z:* 172.0791 calcd for C_8_H_14_NOS^+^ [M + H]^+^; Found 172.0787.

Dess-Martin periodinane (DMP, 247.7 mg, 0.584 mmol) was added to a solution of 2,2-dimethyl-1-(thiazol-5-yl)propan-1-ol (**S4**) (50 mg, 0.292 mmol) in CH_2_Cl_2_ (3 mL) at rt. The reaction was monitored by TLC and was quenched upon completion at 30 min with the addition of satd aq. Na_2_S_2_O_3_ (10 mL) at rt. The mixture was transferred to a separatory funnel, the aqueous layer extracted with CH_2_Cl_2_ (20 mL) and the combined organics were washed with H_2_O (15 mL), satd NaCl (15 mL), dried over Na_2_SO_4_, and concentrated *in vacuo*. Flash chromatography on silica gel (0→20% EtOAc/Hex) provided the ketone product **S5** as a yellow oil (19.0 mg, 0.112 mmol, 38%). *R*_f_ = 0.41 (20% EtOAc/Hex). ^1^H NMR (400 MHz, CDCl_3_) δ 8.92 (s, 1H), 8.50 (s, 1H), 1.39 (s, 9H). ^13^C NMR (101 MHz, CDCl_3_) δ 198.9, 158.0, 146.7, 138.2, 44.5, 27.9 (3C). HRMS (ESI) *m/z:* 170.0634 calcd for C_8_H_12_NOS^+^ [M + H] ^+^ Found 170.0632.

According to General Procedure A, 2-pivaloylthiazole (**S5**) (29.6 mg, 0.175 mmol) was combined with NH_2_OH·HCl (67.7 mg, 1.05 mmol) and NaOAc (14.4 mg, 0.175 mmol) in EtOH (2 mL) and H_2_O (0.5 mL) and heated to 70°C overnight. After stirring for 16 h, the reaction mixture was cooled to rt, diluted with H_2_O (15 mL), and extracted with EtOAc (3 × 15 mL). The combined extracts were washed with H_2_O (10 mL) and satd NaCl (10 mL), and concentrated under reduced pressure. The crude oxime **S6** was used in the next step without further purification.

According to a modified General Procedure B, the crude oxime **S6** was added at rt to a stirring solution of 4-methoxybenzoic acid (80.0 mg, 0.525 mmol), EDC·HCl (100.6 mg, 0.525 mmol), and DMAP (21.4 mg, 0.175 mmol) in CH_2_Cl_2_ (3 mL). After 16 h, the reaction mixture was diluted with CH_2_Cl_2_ (20 mL), washed with satd Na_2_CO_3_ (20 mL) and satd NaCl (20 mL), dried over Na_2_SO_4_, and concentrated under reduced pressure. The crude product was subjected flash chromatography on silica gel (0→30% EtOAc/Hex) to provide product 20 as a white solid (27.0 mg, 0.085 mmol, 49%).^1^H NMR (400 MHz, CDCl_3_) δ 8.98 (d, *J* = 0.7 Hz, 1H), 7.90 (d, *J* = 0.7 Hz, 1H), 7.69 (d, *J* = 9.0 Hz, 2H), 6.84 (d, *J* = 9.0 Hz, 2H), 3.82 (s, 3H), 1.36 (s, 9H). ^13^C NMR (101 MHz, CDCl_3_) δ 165.7, 163.8, 163.2, 154.4, 142.9, 131.8 (2C), 125.5, 120.8, 114.0 (2C), 55.6, 39.1, 28.2 (3C). HRMS (ESI) *m/z:* 341.0930 calcd for C_16_H_18_N_2_O_3_SNa^+^ [M + Na]^+^; Found 341.0930.

###### (*Z*)-Pivaloylbenzene *O*-(4-methoxybenzoyl) oxime (21)

**Figure:**
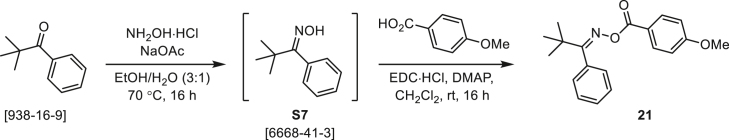

According to General Procedure A, 2,2,2-trimethylacetophenone (500 mg, 3.08 mmol) was combined with NH_2_OH·HCl (1.19 g, 18.5 mmol) and NaOAc (253 mg, 3.08 mmol) in EtOH (30 mL) and H_2_O (10 mL) and heated to 70°C overnight. After stirring for 16 h, the reaction mixture was cooled to rt, diluted with H_2_O (15 mL), and extracted with EtOAc (3 × 20 mL). The combined extracts were washed with H_2_O (10 mL) and satd NaCl (15 mL), and concentrated under reduced pressure. The crude oxime **S7** (232.4 mg) was used in the next step without further purification. *R*_f_ = 0.46 (10% EtOAc/Hex).

According to a modified General Procedure B, the oxime **S7** (18.9 mg, 0.110 mmol) was added at rt to a stirring solution of 4-methoxybenzoic acid (50.1 mg, 0.329 mmol), EDC·HCl (63.1 mg, 0.329 mmol), and DMAP (13.4 mg, 0.110 mmol) in CH_2_Cl_2_ (2 mL). After 16 h, the reaction mixture was diluted with CH_2_Cl_2_ (20 mL), washed with satd Na_2_CO_3_ (20 mL) and satd NaCl (20 mL), dried over Na_2_SO_4_, and concentrated under reduced pressure. The crude product was subjected flash chromatography on silica gel (0→30% EtOAc/Hex) to provide product 21 as a white solid (29.5 mg, 0.095 mmol, 76%). *R*_f_ = 0.51 (30% EtOAc/Hex). ^1^H NMR (400 MHz, CDCl_3_) δ 7.48 (d, *J* = 8.9 Hz, 2H), 7.45–7.37 (m, 3H), 7.13 (dd, *J* = 7.8, 1.7 Hz, 2H), 6.74 (d, *J* = 8.9 Hz, 2H), 3.78 (s, 3H), 1.32 (s, 9H). ^13^C NMR (101 MHz, CDCl_3_) δ 175.3, 163.6, 163.4, 133.8, 131.5 (2C), 128.2, 128.1 (2C), 126.9 (2C), 121.5, 113.7 (2C), 55.5, 38.5, 28.2 (3C). HRMS (ESI) *m/z:* 334.1413 calcd for C_19_H_21_NO_3_Na^+^ [M + Na]^+^; Found 334.1413.

###### (*Z*)-3-Pivaloylpyridine *O*-(4-methoxybenzoyl) oxime (22)

**Figure:**
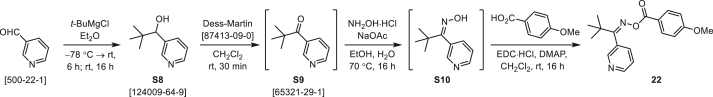

As an adaptation of a reported procedure,^85^ a solution of nicotinaldehyde (300 mg, 2.65 mmol) in dry Et_2_O (30 mL) was degassed with argon and then cooled to −78°C. A solution of *t*-BuMgCl (3.18 mL, 1 M in THF, 3.18 mmol) was added dropwise to the stirring aldehyde at −78°C over 10 min. The reaction was allowed to warm to rt gradually over 6 h and left to stir at rt overnight before it was quenched with a slow addition of 5% HCl at 0°C (10 mL). The mixture was then diluted with Et_2_O (20 mL), washed with 1 M HCl (40 mL), satd NaCl (50 mL), dried over Na_2_SO_4_, and concentrated under reduced pressure. Flash chromatography on silica gel (0→10% MeOH/CH_2_Cl_2_) provided alcohol **S8** as a yellow solid (180.3 mg, 1.09 mmol, 41%). *R*_f_ = 0.27 (10% MeOH/CH_2_Cl_2_). ^1^H NMR (400 MHz, CDCl_3_) δ 8.48 (d, *J* = 2.3 Hz, 1H), 8.46 (dd, *J* = 4.8, 1.7 Hz, 1H), 7.67 (appdt, *J* = 7.9, 1.8 Hz, 1H), 7.24 (d, *J* = 7.9, 4.8 Hz, 1H), 4.42 (s, 1H), 2.52 (brs, 1H), 0.92 (s, 9H). ^13^C NMR (101 MHz, CDCl_3_) δ 149.2, 148.7, 137.7, 135.3, 122.9, 80.2, 35.9, 25.8 (3C). HRMS (ESI) *m/z:* 166.1226 calcd for C_10_H_16_NO^+^ [M + H]^+^; Found 166.1230.

Dess-Martin periodinane (DMP, 294.1 mg, 0.690 mmol) was added to a solution of alcohol **S8** (75.0 mg, 0.460 mmol) in CH_2_Cl_2_ (5 mL) at rt. The reaction was monitored by TLC and was quenched upon completion at 30 min with the addition of satd aq. Na_2_S_2_O_3_ (10 mL) at rt. The mixture was transferred to a separatory funnel, the aqueous layer extracted with CH_2_Cl_2_ (20 mL) and the combined organics were washed with H_2_O (15 mL), satd NaCl (15 mL), dried over Na_2_SO_4_, and concentrated *in vacuo*. Flash chromatography on silica gel (0→10% MeOH/CH_2_Cl_2_) provided the ketone product **S9** as a yellow oil (73.3 mg, 0.450 mmol, 97%). *R*_f_ = 0.67 (MeOH/CH_2_Cl_2_)). ^1^H NMR (400 MHz, CDCl_3_) δ 8.97 (dd, *J* = 2.2, 0.9 Hz, 1H), 8.69 (dd, *J* = 4.9, 1.7 Hz, 1H), 7.99 (ddd, *J* = 8.0, 2.3, 1.7 Hz, 1H), 7.36 (ddd, *J* = 8.0, 4.9, 0.9 Hz, 1H), 1.36 (s, 9H). ^13^C NMR (101 MHz, CDCl_3_) δ 151.7, 149.9, 135.7, 123.3, 44.6, 27.8 (3C). Signals for the carbonyl carbon and the quaternary carbon of the pyridine were not detected. HRMS (ESI) *m/z:* 164.1070 calcd for C_10_H_14_NO^+^ [M + H]^+^; Found 164.1067. Spectral data are consistent with a literature report.^86^

Following general procedure A, ketone **S9** (50 mg, 0.306 mmol) was combined with NH_2_OH·HCl (119 mg, 1.84 mmol) and NaOAc (25.1 mg, 0.306 mmol) in EtOH (3 mL) and H_2_O (1 mL) and heated to 70°C overnight. After stirring for 16 h, the reaction mixture was cooled to rt, diluted with H_2_O (5 mL), and extracted with EtOAc (3 × 10 mL). The combined extracts were washed with H_2_O (10 mL) and satd NaCl (10 mL), concentrated under reduced pressure, and the crude oxime S10 was carried forward without further purification.

According to a modified General Procedure B, the oxime S10 (31.0 mg, 0.174 mmol) was added at rt to a stirring solution of 4-methoxybenzoic acid (79.4 mg, 0.522 mmol), EDC·HCl (100.1 mg, 0.522 mmol), and DMAP (21.3 mg, 0.174 mmol) in CH_2_Cl_2_ (3 mL). After 16 h, the reaction mixture was diluted with CH_2_Cl_2_ (20 mL), washed with satd Na_2_CO_3_ (20 mL) and satd NaCl (20 mL), dried over Na_2_SO_4_, and concentrated under reduced pressure. The crude product was subjected flash chromatography on silica gel (0→40% EtOAc/Hex) to provide product **22** as a white solid (31.2 mg, 0.100 mmol, 33% over 2 steps). *R*_f_ = 0.33 (40% EtOAc/Hex). ^1^H NMR (400 MHz, CDCl_3_) δ 8.67 (dd, *J* = 4.9, 1.7 Hz, 1H), 8.45 (dd, *J* = 2.2, 0.9 Hz, 1H), 7.50 (d, *J* = 8.9 Hz, 2H), 7.49 (appdt, *J* = 7.8, 2.0, 1H), 7.39 (ddd, *J* = 7.8, 4.9, 0.9 Hz, 1H), 6.76 (d, *J* = 8.9 Hz, 2H), 3.79 (s, 3H), 1.32 (s, 9H). ^13^C NMR (101 MHz, CDCl_3_) δ 172.1, 163.7, 163.3, 149.7, 147.5, 134.6, 131.5 (2C), 129.8, 123.1, 121.0, 113.8 (2C), 55.5, 38.6, 28.0 (3C). HRMS (ESI) *m/z:* 313.1547 calcd for C_18_H_21_N_2_O_3_H^+^ [M + H]^+^; Found 313.1546.

### Quantification and statistical details

All experiments described in this study were conducted in at least biological duplicate and results were verified through testing on different days. Graphpad PRISM 9 was used for plotting most data presented in the study, and error was calculated in the software as the SEM of the replicates. *n*-values for each experiment can be found in each corresponding figure legend.

## Supplemental information

Document S1. Figures S1‒S9 and Table S1

Data S1. ΔlpcA Transcriptional Reporter Collection Screening Data

Data S2. pPromrcsA-GFP genetic induction screen with Keio and CRISPRi collections

Data S3. Small molecules from screen that induce pPromrcsA-GFP

Data S4. CRISPRi collection screens with antibiotics

Data S5. MAC-0452936 and analogs NMR data

Data S6. MAC-0452936 and Z-(18) X-ray crystallography

Data S7. Strains and plasmid used in study
