# Supplementary material for: A screen for cell envelope stress uncovers an inhibitor of prolipoprotein diacylglyceryl transferase, Lgt, in Escherichiacoli
Source: iScience. 2024 Sep 5;27(10):110894. doi: 10.1016/j.isci.2024.110894 (PMC11456916; doi:10.1016/j.isci.2024.110894)
Supplement: Document S1. Figures S1‒S9 and Table S1 [file mmc1.pdf]

iScience, Volume *27*

Supplemental information

A screen for cell envelope stress uncovers an inhibitor of prolipoprotein diacylglyceryl transferase, Lgt, in *Escherichia coli*

Kenneth Rachwalski, Sean J. Madden, Nicole Ritchie, Shawn French, Timsy Bhando, Adele Girgis-Gabardo, Megan Tu, Rodion Gordzevich, Rowan Ives, Amelia B.Y. Guo, Jarrod W. Johnson, Yiming Xu, Sharookh B. Kapadia, Jakob Magolan, and Eric D. Brown

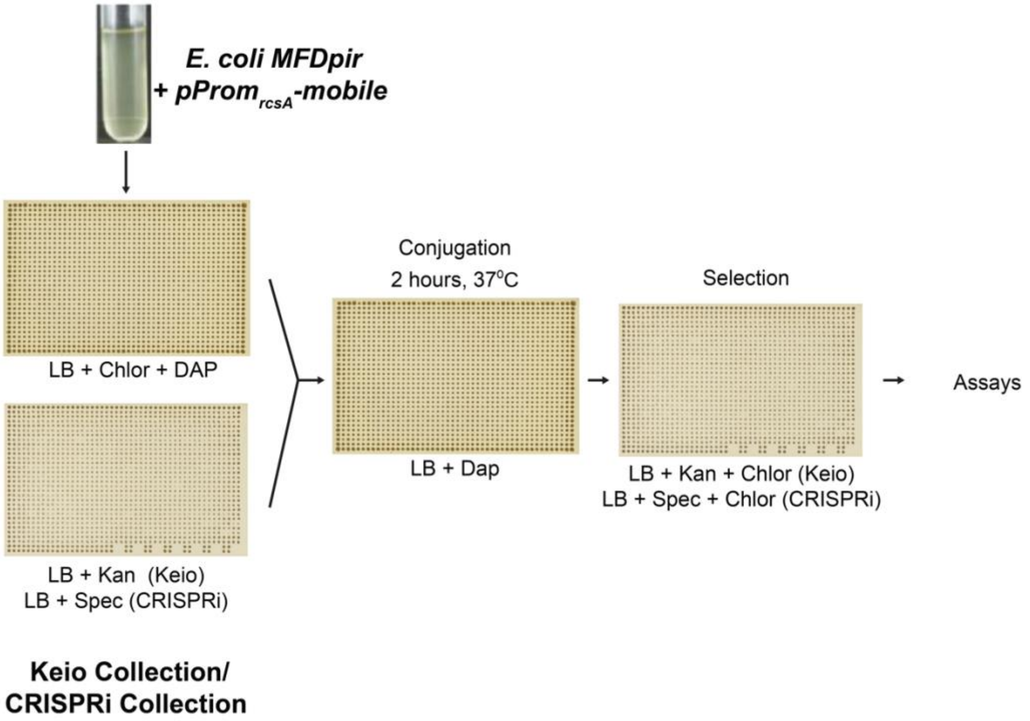

Figure S1. Conjugation workflow for introducing pProm$_{rcsA}$-GFP-mobile into the Keio and CRISPRi collections, related to Figure 2. The donor strain (*E. coli* MFD*pir*) harboring pProm$_{rcsA}$-GFP-mobile and the recipient strains (the Keio or CRISPRi collections) were arrayed at 1,536 colony density with appropriate selection using the Singer ROTOR. Donor and recipient strains were then co-spotted to allow conjugation, then pinned onto LB-agar with appropriate double selection to select for exconjugants.

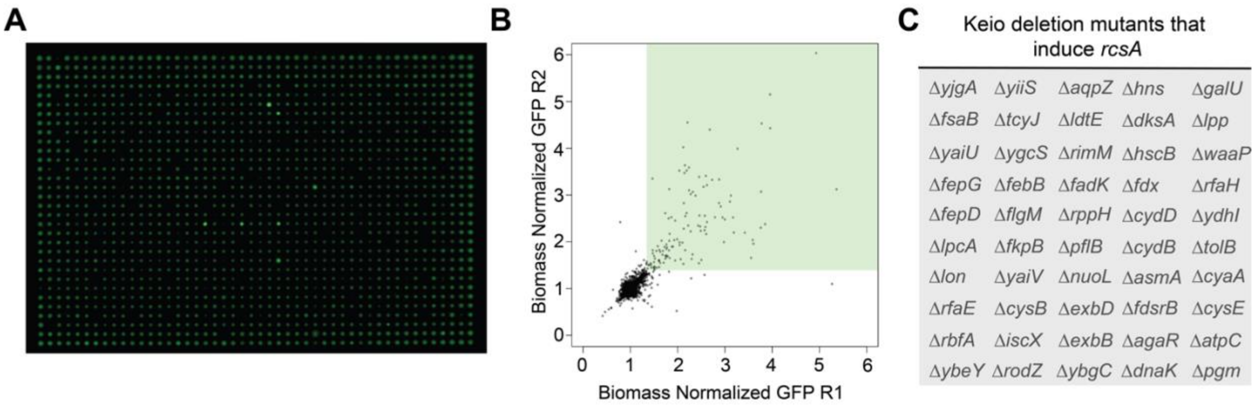

Figure S2. **Genomic screen for pProm$_{rcsA}$-GFP-mobile induction with the Keio collection, related to Figure 2**. (**A**) A representative GFP plate image of 1,536 different deletion mutants harboring the bioreporter grown on MOPS-minimal agar. Colonies of deletion mutants with higher bioreporter induction are clearly visible. Two biological replicates of each mutant were assayed. Colony array was imaged using the BioRad Chemidoc (Cy2 channel) (**B**) Replicate plots of biomass normalized GFP expression. Region in green indicates a three standard deviation increase in GFP expression relative to the mean of the dataset. (**C**) *E. coli* deletion strains in the Keio collection that induce bioreporter expression.

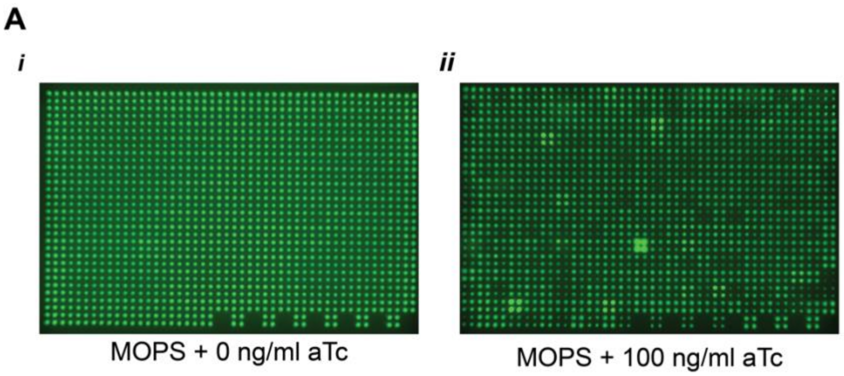

B

CRISPRi constructs that induce *rcsA*

bamA	*lpxC*	*lpxD*	*igaA*	*pgsA*	*racR*
uppS	*der*	*yejM*	*hscA*	*fdx*	*lspA*
prs	*grpE*	*alaS*	*lgt*	*secE*	*nusA*
iraM	*pdxH*	*glmU*	*csrA*	*nusG*	*rho*
eno	*ispG*	*dapA*	*asd*	*iscU*	*rpoC*
rpoA	*rpoD*	*ycaR*	*ptsI*	*infA*	*fbaA*
pyrG	*hemD*	*pgk*	*rplJ*	*rplL*	*rplY*
dapA	*dapE*	*dapD*	*obgE*	*gapA*	*ispA*

Figure S3. **Genomic screen for pProm$_{rcsA}$-GFP-mobile induction with the CRISPRi collection, related to Figure 2**. (**A**) Representative image of the CRISPRi collection harboring pProm$_{rcsA}$-GFP grown at 1,536 colony density on MOPS-minimal agar in the absence (i) and presence (ii) of CRISPRi induction with anhydrotetracycline (aTc). In each 1,536 colony array, four technical replicates of each CRISPRi strain are grown in quadrants. Each array contains multiple empty vector controls throughout the plate. Colony array was imaged using the BioRad Chemidoc (Cy2 channel). (**B**) *E. coli* CRISPRi strains that induce bioreporter expression by at least 25% relative to the empty vector controls.

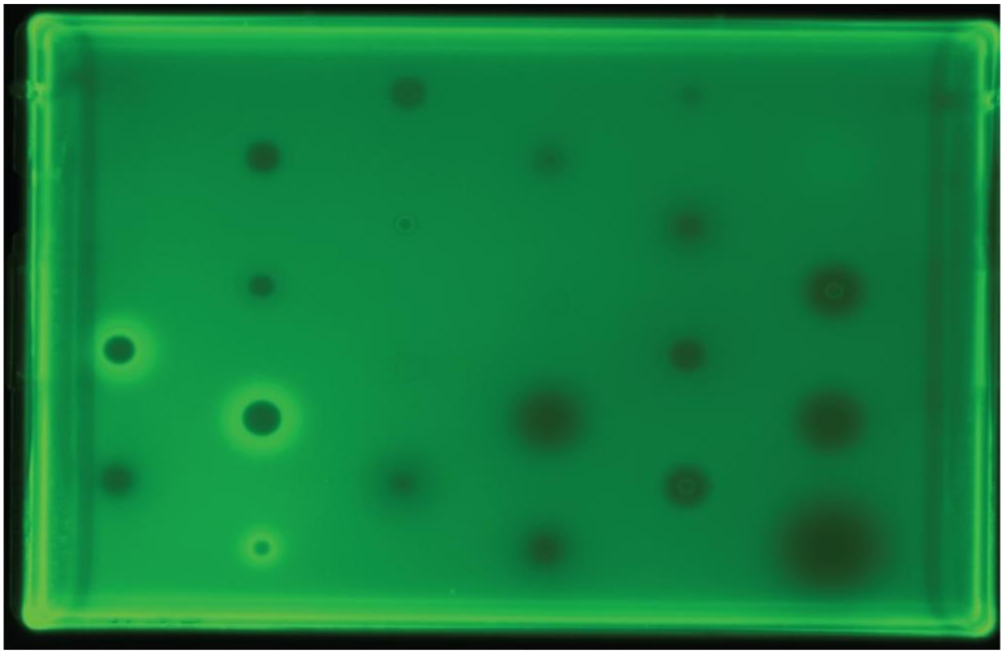

Figure S4. **Representative screening plate from small molecule secondary screen, related to Figure 3**. Compounds from a bioactive small molecule collection were spotted onto MOPS-minimal agar impregnated with *E. coli* Δ*tolC*-pore harboring $pProm_{rcsA}$-GFP-mobile to test for GFP induction. Assay plates are grown overnight, then imaged for GFP production. Molecules that induce the bioreporter are identified qualitatively by visually inspecting images – three hit compounds are seen on representative plate. Colony array was imaged using the BioRad Chemidoc (Cy2 channel).

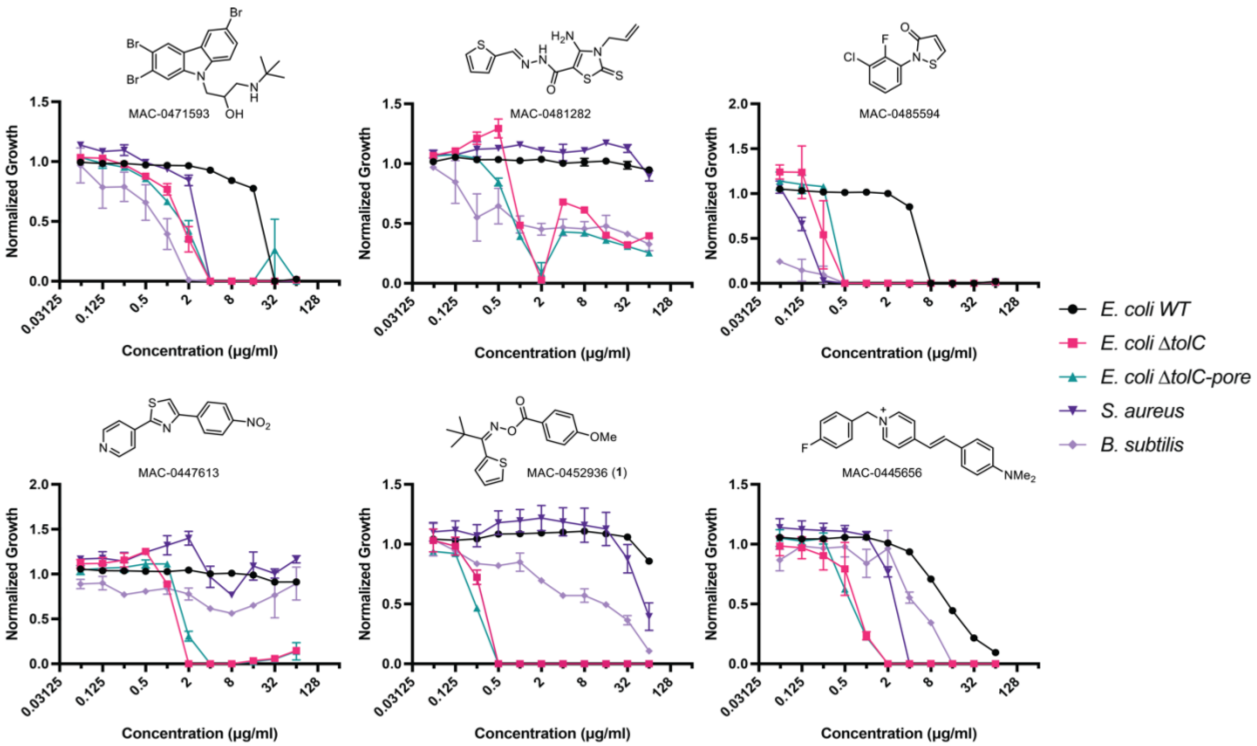

Figure S5. MICs of six prioritized small molecules against wildtype *E. coli, E. coli ΔtolC, E. coli ΔtolC*-pore and the Gram-positive bacteria *S. aureus and B. subtilis*, related to Figure 3. All assays were conducted in biological triplicate, Data are represented as mean +/- SEM..

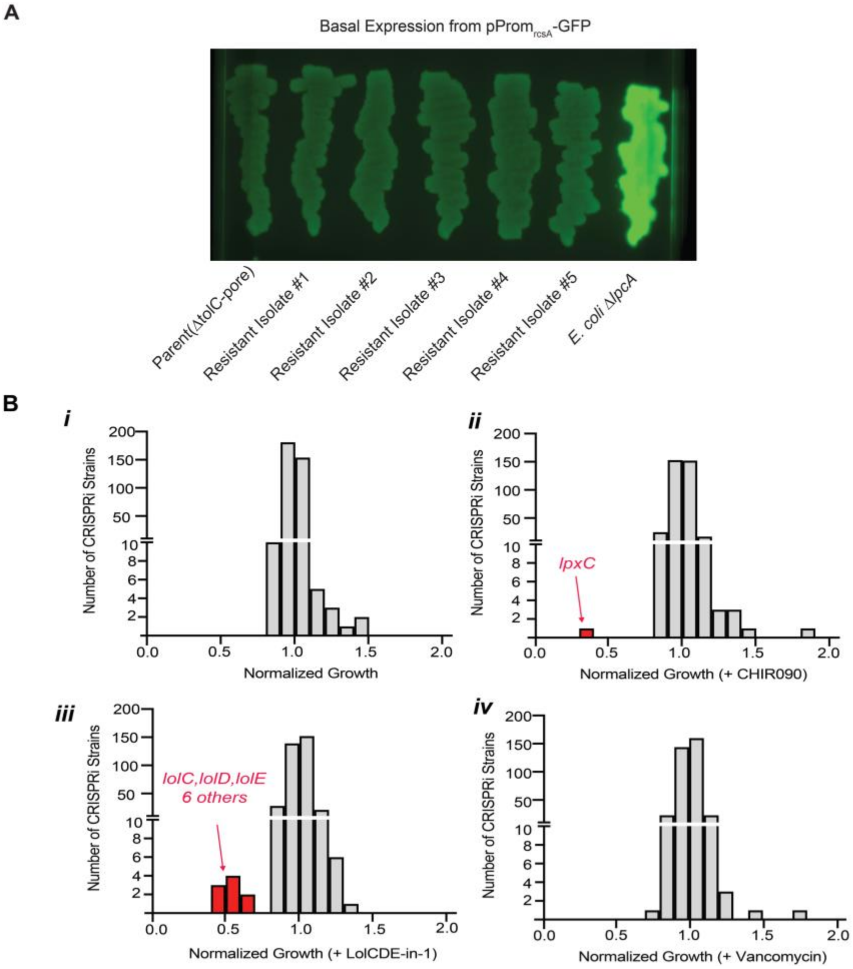

Figure S6. **MAC-0452936 resistance isolates do not exhibit higher Rcs activation and CRISPRi collection screen with other antibiotics, related to Figure 4.** (**A**) pProm$_{rcsA}$—GFP was transformed into the parental isolate, into the five MAC-0452936 spontaneous resistance isolates, and into *E. coli* Δ*lpcA* to serve as a control. Each of the strains were streaked adjacent to each other on an LB agar plate, and GFP florescence was visualized using the BioRad Chemidoc. (**B**) The CRISPRi collection in the *E. coli* Δ*tolc*-pore background was grown on solid LB agar plates (***i***) without drug, or in the presence of sub-inhibitory concentrations of (***ii***) CHIR090 (7 ng/ml), (***iii***) LolCDE-in-1 (6 ng/ml), or (***iv***) Vancomycin (250 ng/ml). Each CRISPRi strain was grown in a colony array in technical quadruplicate. The mean normalized growth of the 4 replicates is plotted for each CRISPRi strain.

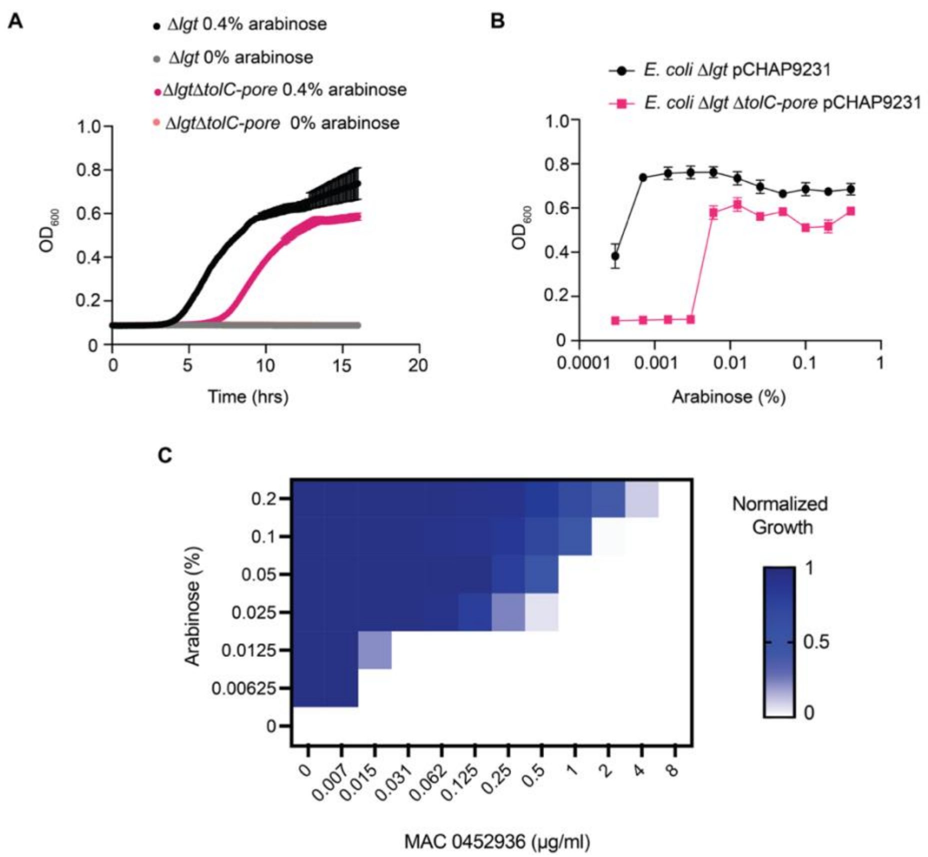

Figure S7. **MAC-0452936 MIC correlates with *in trans* expression of *lgt* from an arabinose inducible vector, related to Figure 4**. (**A**) Growth curve of *E. coli* Δ*lgt* pChap9231 and *E. coli* Δ*lgt*Δ*tolC-pore* pChap9231 with and without arabinose supplementation. Both strains require arabinose for normal growth. Data represents three technical replicates for each growth curves, data are represented as mean +/- SEM. (**B**) Growth of of *E. coli* Δ*lgt* pChap9231 and *E. coli* Δ*lgt*Δ*tolC-pore* pChap9231 at 18 hours with varying arabinose supplementation. *E. coli* Δ*lgt*Δ*tolC-pore* pChap9231 requires higher levels of arabinose for growth. Data are represented as mean +/- SEM. (**C**) MIC of MAC-0452936 against *E. coli* Δ*lgt*Δ*tolC-pore* pChap9231 with different concentrations of arabinose in the assay medium. White boxes indicate regions lacking growth, and blue boxes indicate growth. MIC values for MAC-0452936 increase with increasing concentrations of arabinose, and thereby increasing concentrations of *lgt* expressed. Data are represented as mean of two replicates.

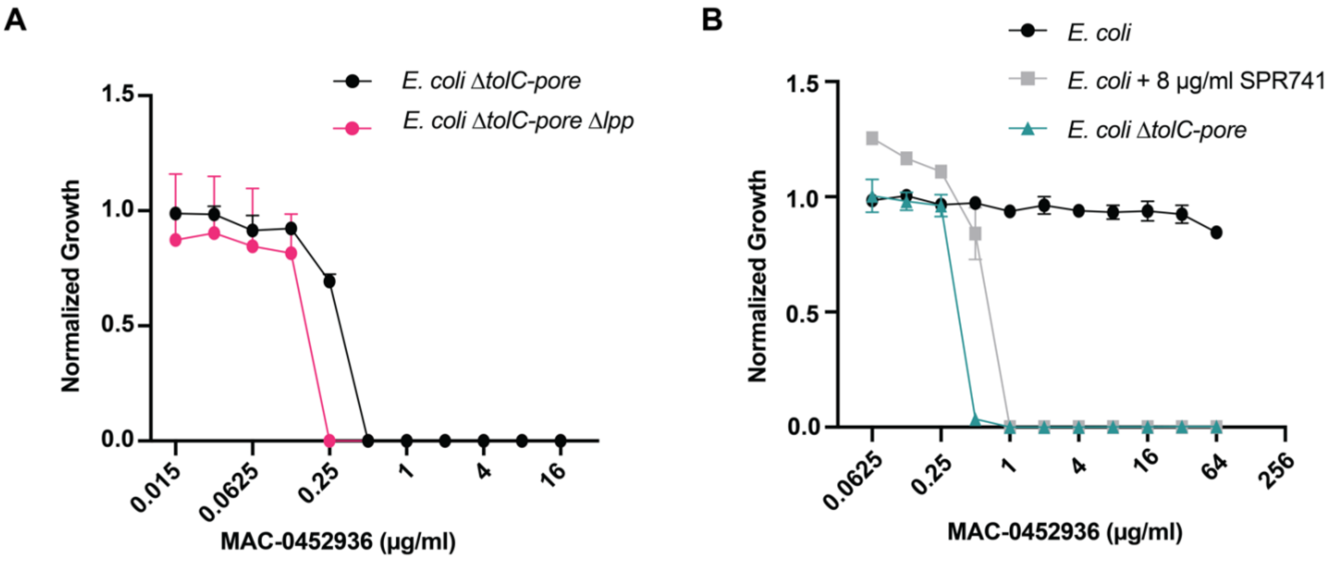

Figure S8. **MAC-0452936 MICs, related to Figure 4.** (**A**) MAC-0452936 MIC against *E. coli* Δ*tolC-pore* and *E. coli* Δ*tolC-pore*Δ*lpp*. An lpp deletion modestly sensitizes *E. coli* to MAC-0452936. (**B**) MAC-0452936 MIC against *E. coli* Δ*tolC*-pore, wildtype *E. coli*, and wildtype *E. coli* in the presence of the antibiotic adjuvant SPR-741. SPR-741 sensitizes wildtype *E. coli* to MAC-0452936. MICs were conducted in technical triplicate, data are represented as mean +/- SEM.

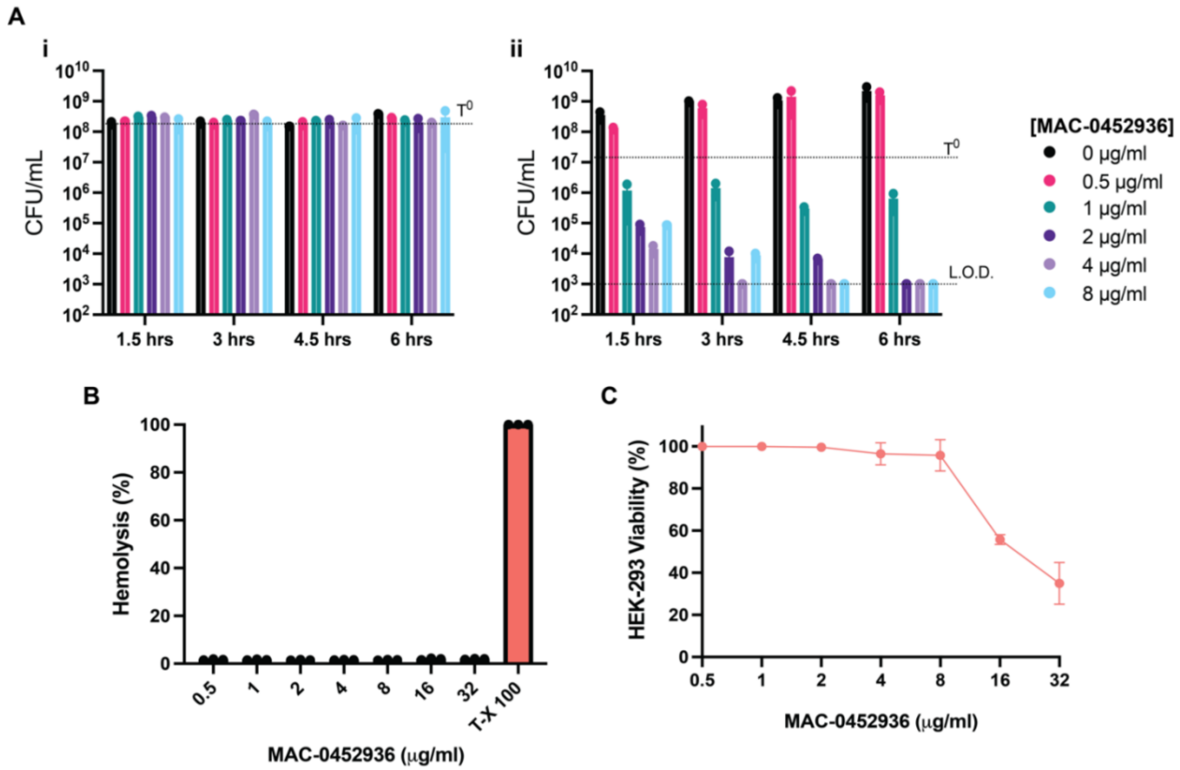

Figure S9. **MAC-0452936 time kill assay and eukaryotic toxicity, related to Figure 4. (A)** Non growing *E. coli* ∆*tolC*-pore in PBS **(i),** or, *E. coli* ∆*tolC*-pore at mid-log phase of growth in LB broth **(ii)** were exposed to increasing concentrations of MAC-0452936 for six hours. Colony forming units (CFUs) from two independent biological replicates were enumerated at different time points to examine kinetics of killing by MAC-0452936. Inoculum at that start of experiment and the limit of detection (L.O.D.) are shown with dotted lines. **(B)** Hemolysis of human red blood cells exposed to different concentrations of MAC-0452936. Triton X-100 was used as a positive control. Data was collected in technical triplicate. Data are represented as mean +/- SEM. **(C)** Viability of human HEK-293 cells exposed to MAC-0452936. Experiment was conducted in technical triplicate, data are represented as mean +/- SEM.

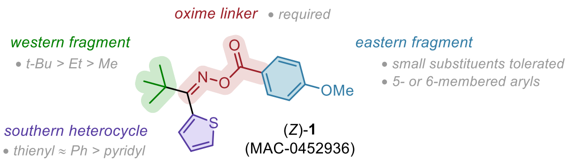

Compound Structure		McMaster cmpd name MLEB	*E. coli* Δ*tolC*-pore[b]	*E. coli* Δ*tolC*	*E. coli* pore	*E. coli* WT SPR741[a] –	*E. coli* WT SPR741[a] +	*E. coli* C0244 SPR741 –	*E. coli* C0244 SPR741 +	*E. coli* CFT073 SPR741 –	*E. coli* CFT073 SPR741 +	*S.* Tm SL1344 SPR741 –	*S.* Tm SL1344 SPR741 +
Eastern Heterocycle Modifications													
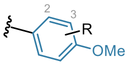	**1**: R = H	22056	0.5	1	>32	>32	0.5	>32	2	>32	2	>32	4
	**2**: R = 2-F	22066	0.5	1	>32	>32	0.5	>32	2	>32	1	>32	4
	**6**: R = 3-NH_2	22062	16	16	>32	>32	8	>32	>32	>32	>32	>32	>32
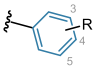	**7**: R = 3,5-$(OMe)_2$	22057	>32	>32	>32	>32	4	>32	>32	>32	>32	>32	>32
	**8**: R = 4-Cl	22061	2	16	>32	>32	2	>32	4	>32	4	>32	>32
	**9**: R = 4-CF_3	23001	>32	>32	>32	>32	4	>32	8	>32	>32	>32	>32
	**10**: R = NHMe	22063	4	4	>32	>32	2	>32	>32	>32	>32	>32	>32
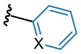	**11**: X = CH	22067	1	1	>32	>32	0.5	>32	2	>32	2	>32	4
	**12**: X = N	22059	2	2	>32	>32	2	>32	8	>32	8	>32	32
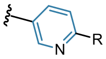	**3**: R = H	22064	1	1	>32	>32	0.5	>32	4	>32	4	>32	8
	**13**: R = OMe	22005	2	4	>32	>32	2	>32	8	>32	8	>32	>32
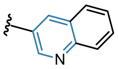	**14**	22058	4	8	>32	>32	2	>32	>32	>32	>32	>32	>32
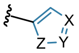	**15**: XYZ = CH=N-O	23002	>32	>32	>32	>32	32	>32	>32	>32	>32	>32	>32
	**4**: XYZ = N=CH-S	22065	2	2	>32	>32	2	>32	8	>32	4	>32	16
	**5**: XYZ = N=CH-NMe	23003	4	4	>32	>32	4	>32	32	>32	8	>32	32
Linker Modifications													
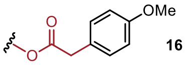	**16**	23034	32	32	>32	>32	4	>32	32	>32	>32	>32	>32
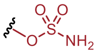	**17** [c]	23004	>32	>32	>32	>32	>32	>32	>32	>32	>32	>32	>32
Western Fragment Modifications													
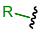	(*E*)-**18**: R = Me	23064	32	32	>32	>32	16	>32	>32	>32	>32	>32	>32
	(*Z*)-**18**: R = Me	23030	>32	>32	>32	>32	16	>32	>32	>32	>32	>32	>32
	**19**: R = Et [d]	23031	>32	>32	>32	>32	8	>32	>32	>32	>32	>32	>32
Southern Heterocycle Modifications													
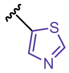	**20**	23035	16	16	>32	>32	16	>32	32	>32	16	>32	>32
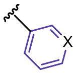	**21**: X = CH	23032	0.5	1	>32	>32	0.5	>32	1	>32	0.5	>32	2
	**22**: X = N	23033	32	32	>32	>32	32	>32	>32	>32	>32	>32	>32

Table S1. MICs (µg/mL) of MAC-0452936 and analogues against a panel of bacterial strains, related to Figure 5. MICs of compounds against strains of *E. coli* and *S.* Tm were evaluated in the absence (–) or presence (+) of outer-membrane permeabilizer SPR741 (8 µg/mL). [b] A hyperpermeable strain of *E. coli* that lacks TolC and expresses a truncated (pore-only) form of FhuA. Oxime stereochemistry was undefined for **17**. [c] Oxime **19** was isolated and assayed as a mixture of stereoisomers (*E*:*Z* 33:67).
