# Supplementary material for: A screen for cell envelope stress uncovers an inhibitor of prolipoprotein diacylglyceryl transferase, Lgt, in Escherichiacoli
Source: iScience. 2024 Sep 5;27(10):110894. doi: 10.1016/j.isci.2024.110894 (PMC11456916; doi:10.1016/j.isci.2024.110894)
Supplement: Data S5. MAC-0452936 and analogs NMR data [file mmc6.pdf]

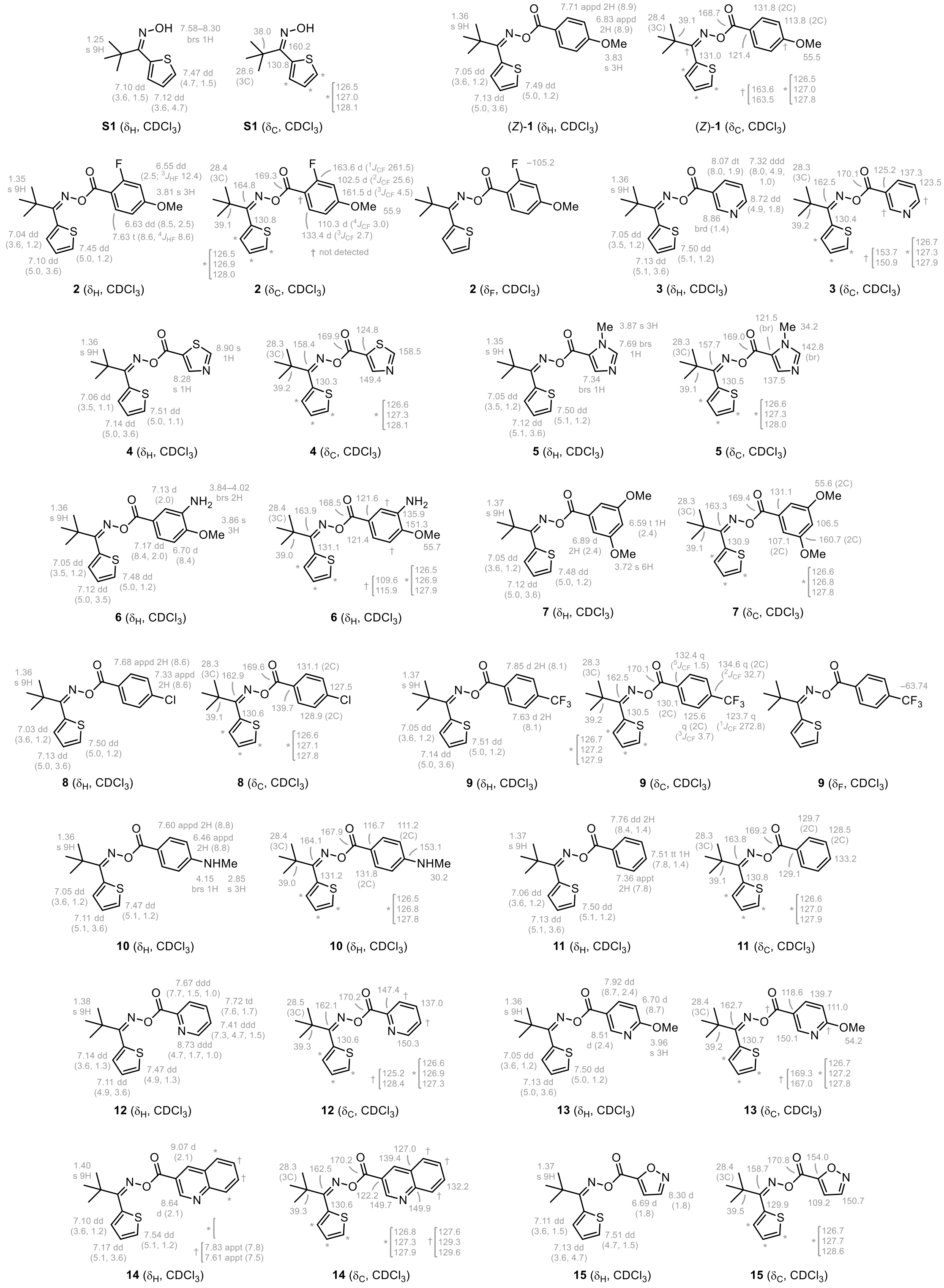

NMR assignments for Lgt inhibitors and synthetic intermediates.

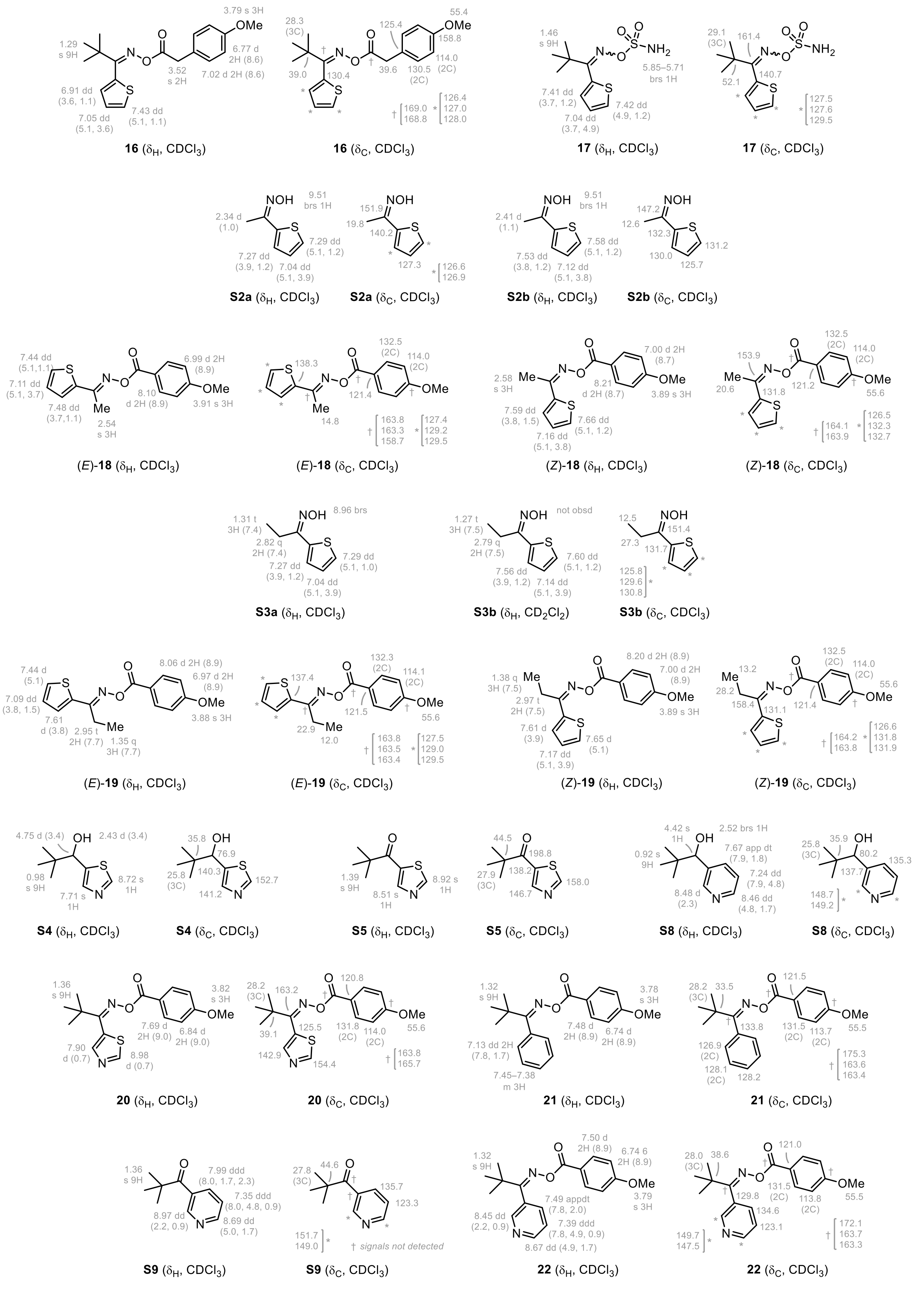

NMR assignments for Lgt inhibitors and synthetic intermediates (Cont.)

^{1}H, ^{13}C, and ^{19}F NMR Spectra

(*Z*)-2-Pivaloylthiophene oxime (**S1**) (^{1}H NMR; 400 MHz; $CDCl_3$)

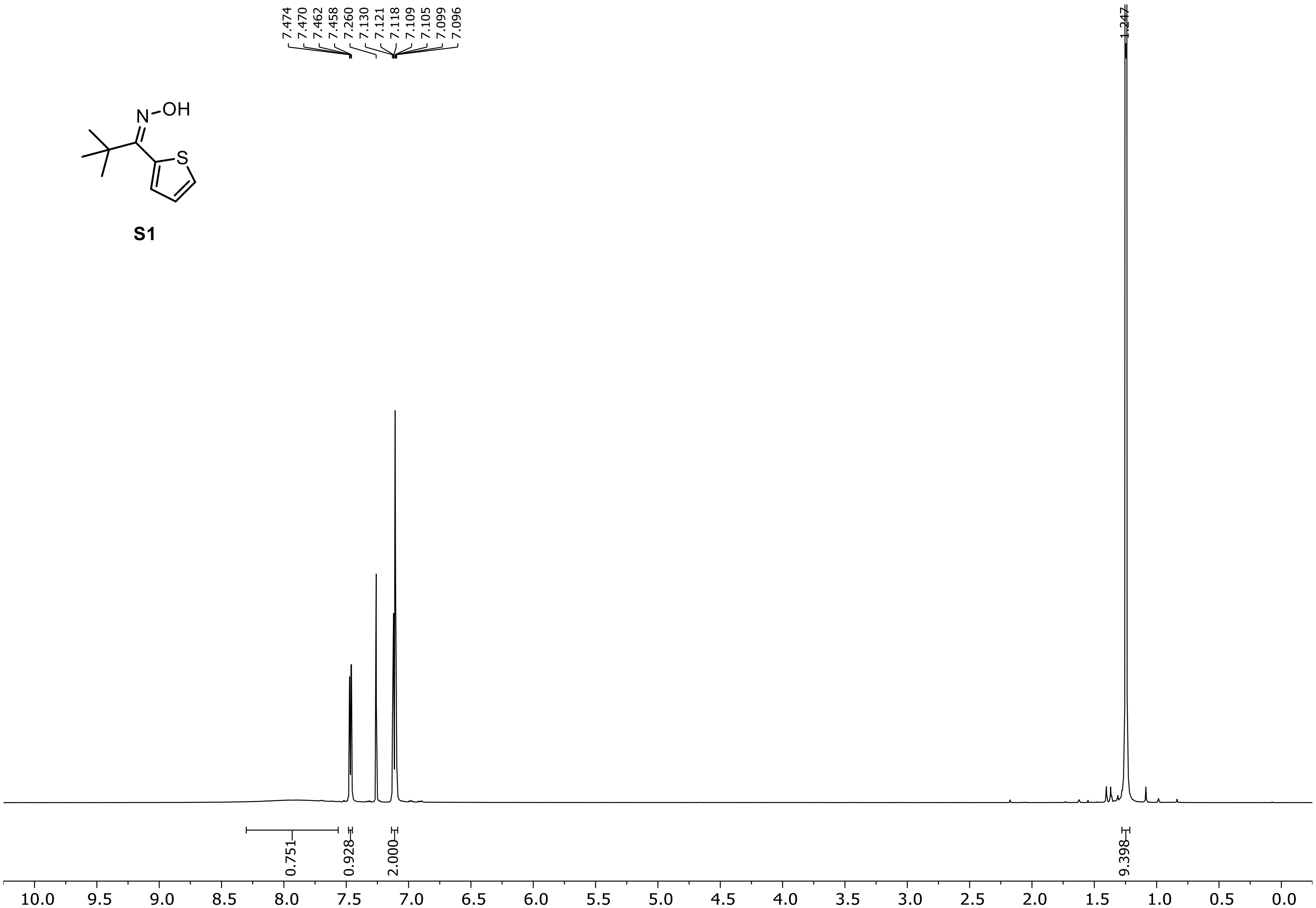

(*Z*)-2-Pivaloylthiophene oxime (**S1**) (^{13}C NMR; 100 MHz; $CDCl_3$)

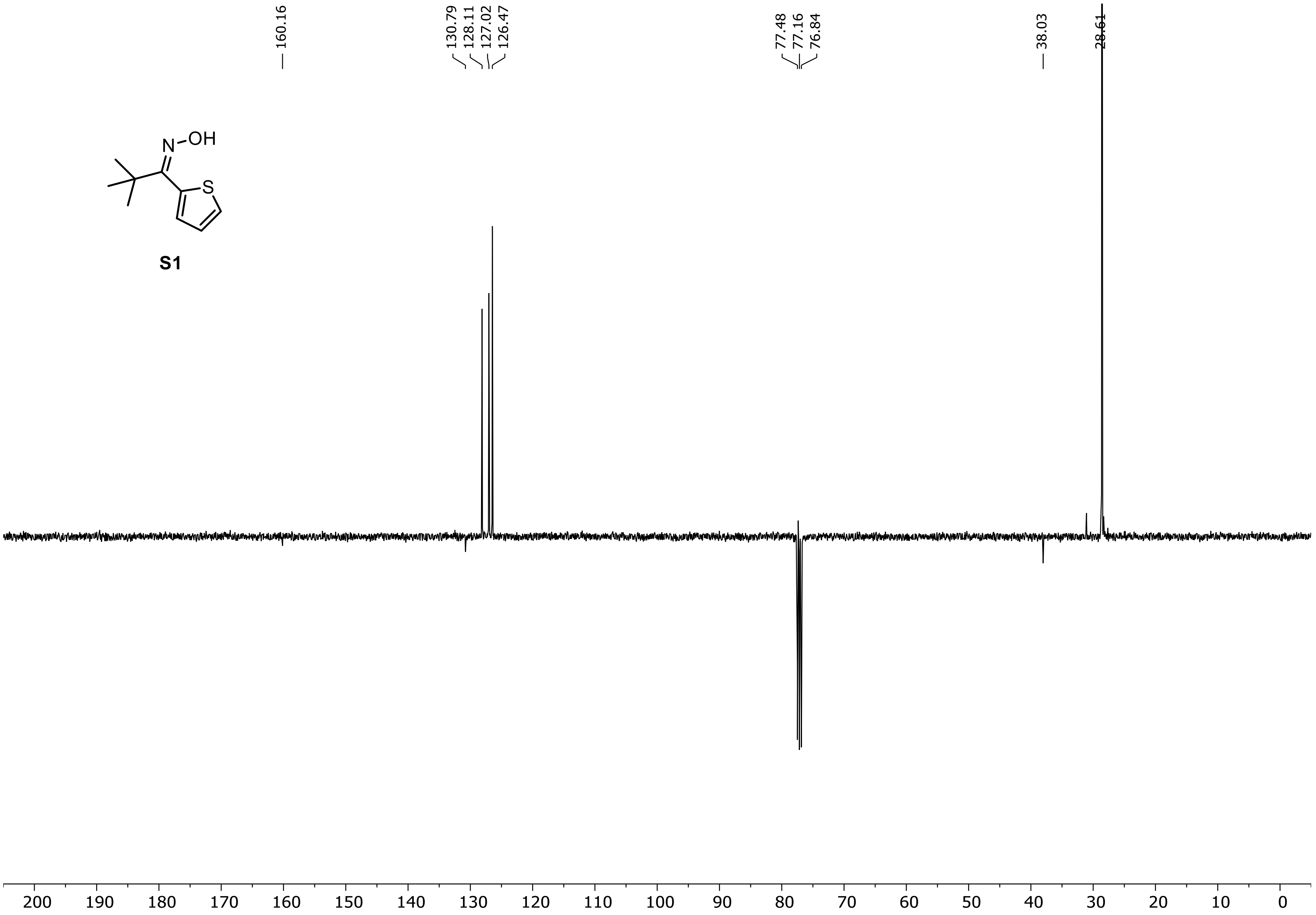

(*Z*)-2-Pivaloylthiophene *O*-(4-methoxybenzoyl) oxime (**1**, MAC-0452936) (^{1}H NMR; 400 MHz; $CDCl_3$)

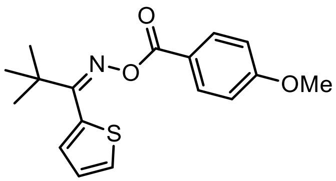

1
(MAC-0452936)

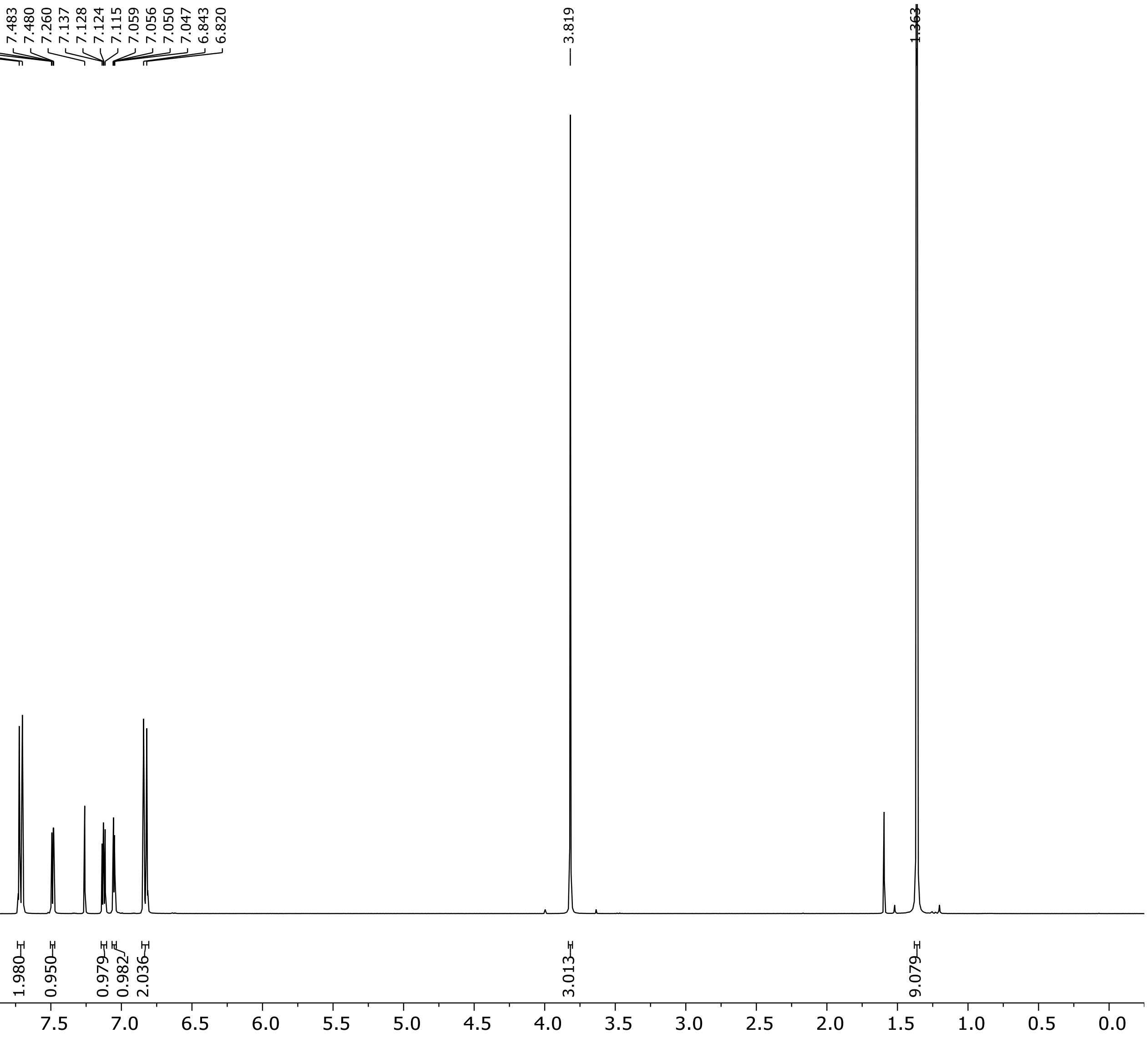

(*Z*)-2-Pivaloylthiophene *O*-(4-methoxybenzoyl) oxime (**1**, MAC-0452936) (^{13}C NMR; 101 MHz; $CDCl_3$)

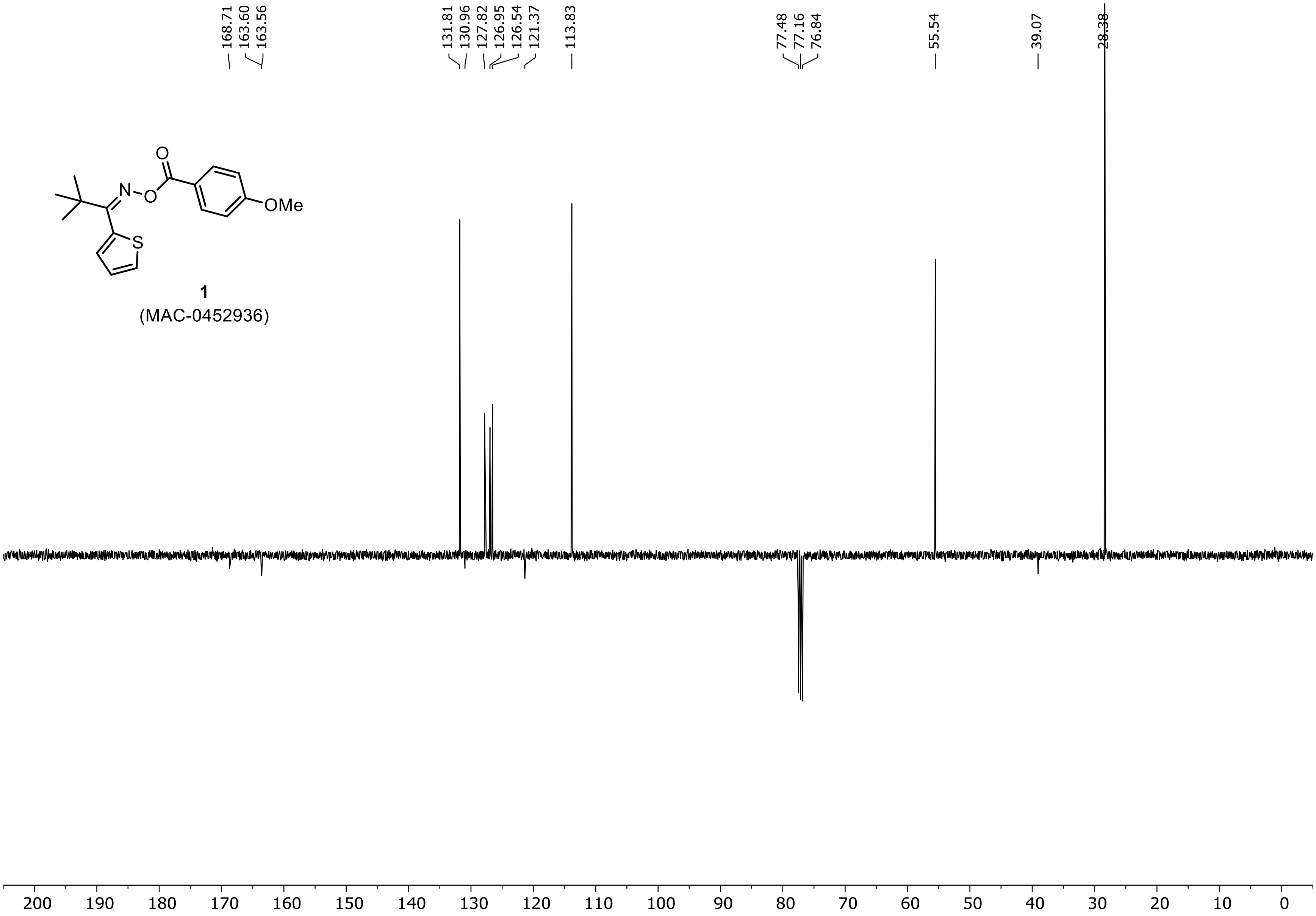

(*Z*)-2-Pivaloylthiophene *O*-(2-fluoro-4-methoxybenzoyl) oxime (**2**) (^{1}H NMR; 400 MHz; $CDCl_3$)

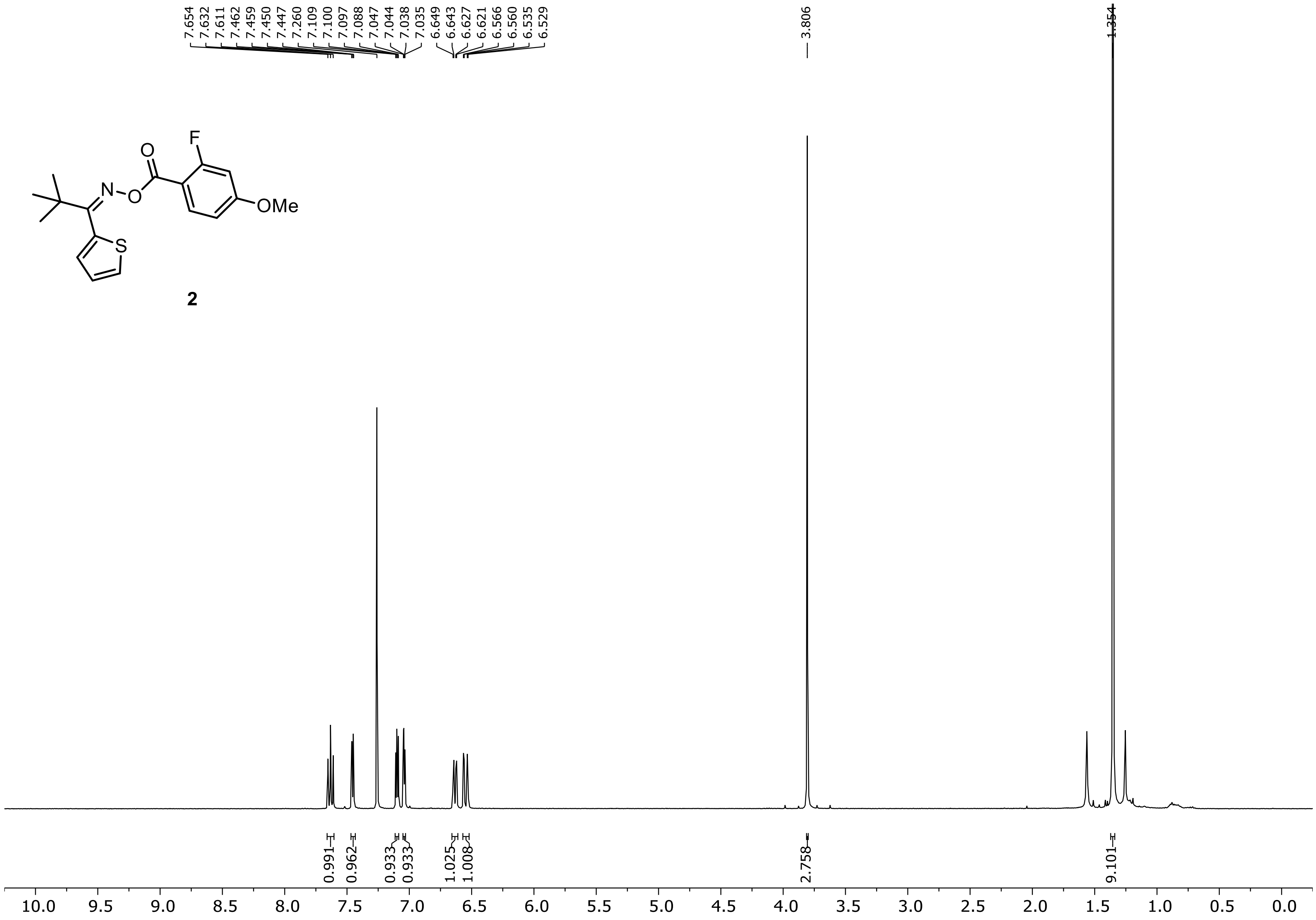

(*Z*)-2-Pivaloylthiophene *O*-(2-fluoro-4-methoxybenzoyl) oxime (**2**) (^{13}C NMR; 101 MHz; $CDCl_3$)

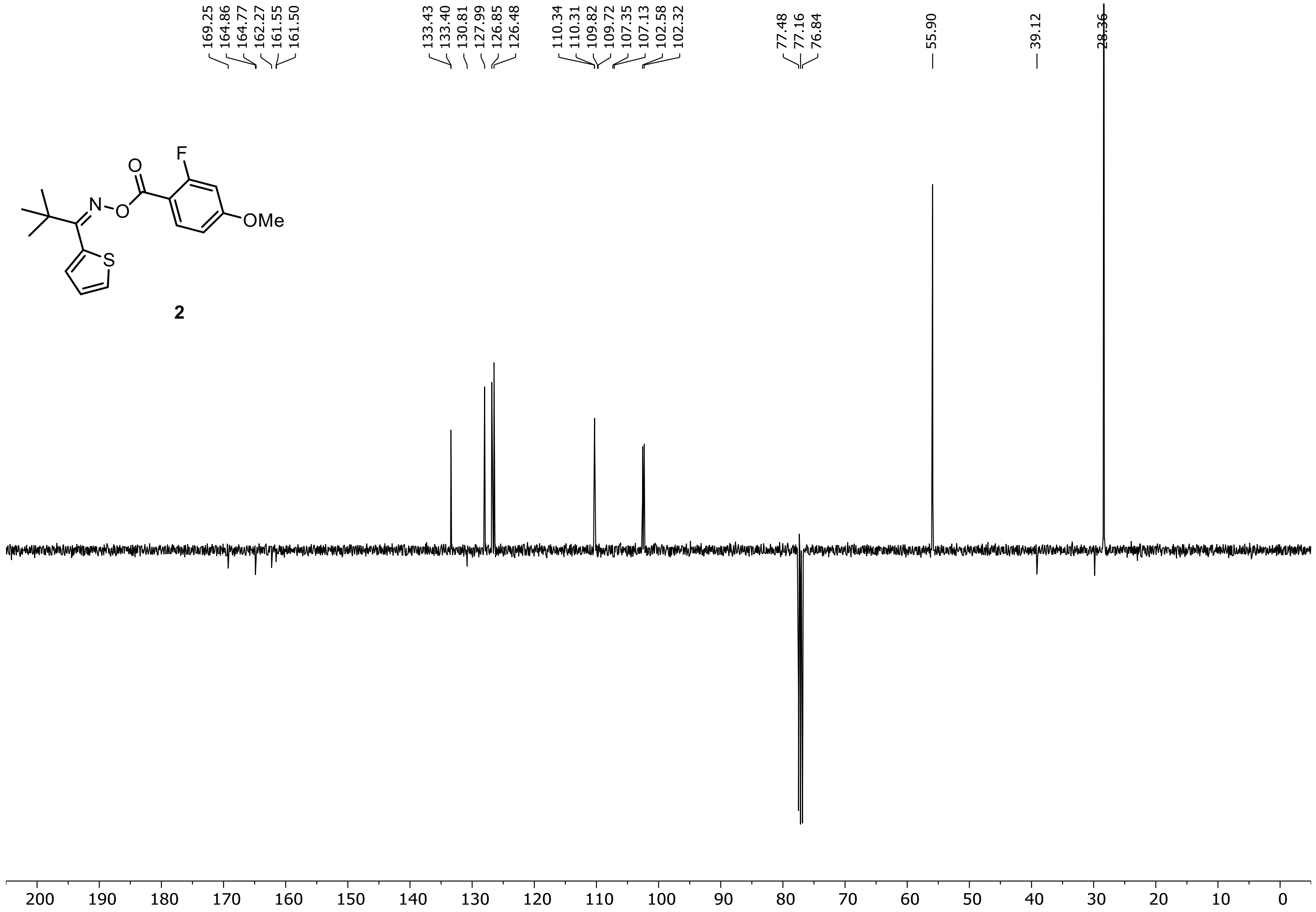

(*Z*)-2-Pivaloylthiophene *O*-(2-fluoro-4-methoxybenzoyl) oxime (**2**) (^{19}F NMR; 377 MHz, $CDCl_3$ + $CFCl_3$)

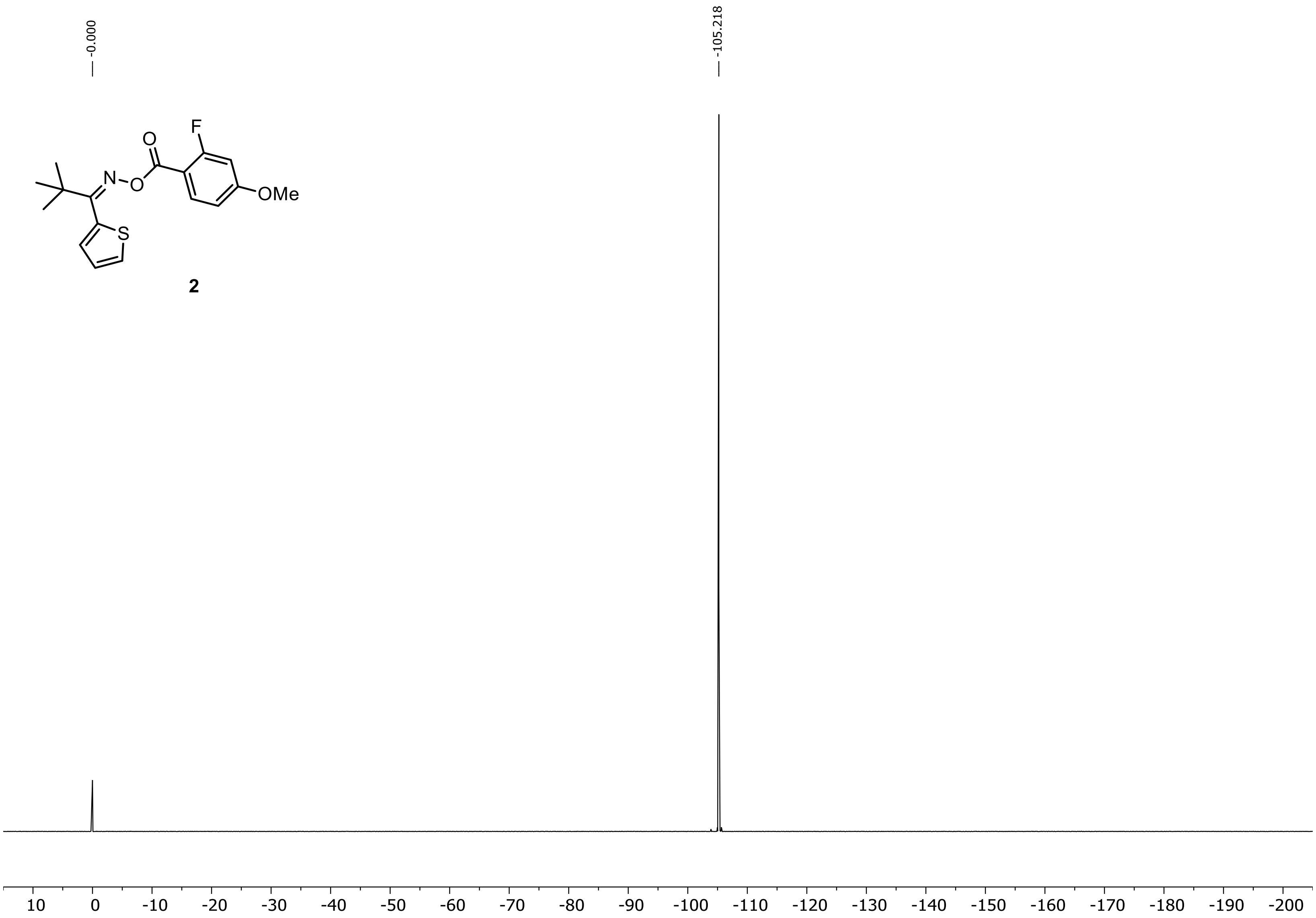

2

(*Z*)-2-Pivaloylthiophene *O*-(pyridine-3-oyl) oxime (**3**) (^{1}H NMR; 400 MHz; $CDCl_3$)

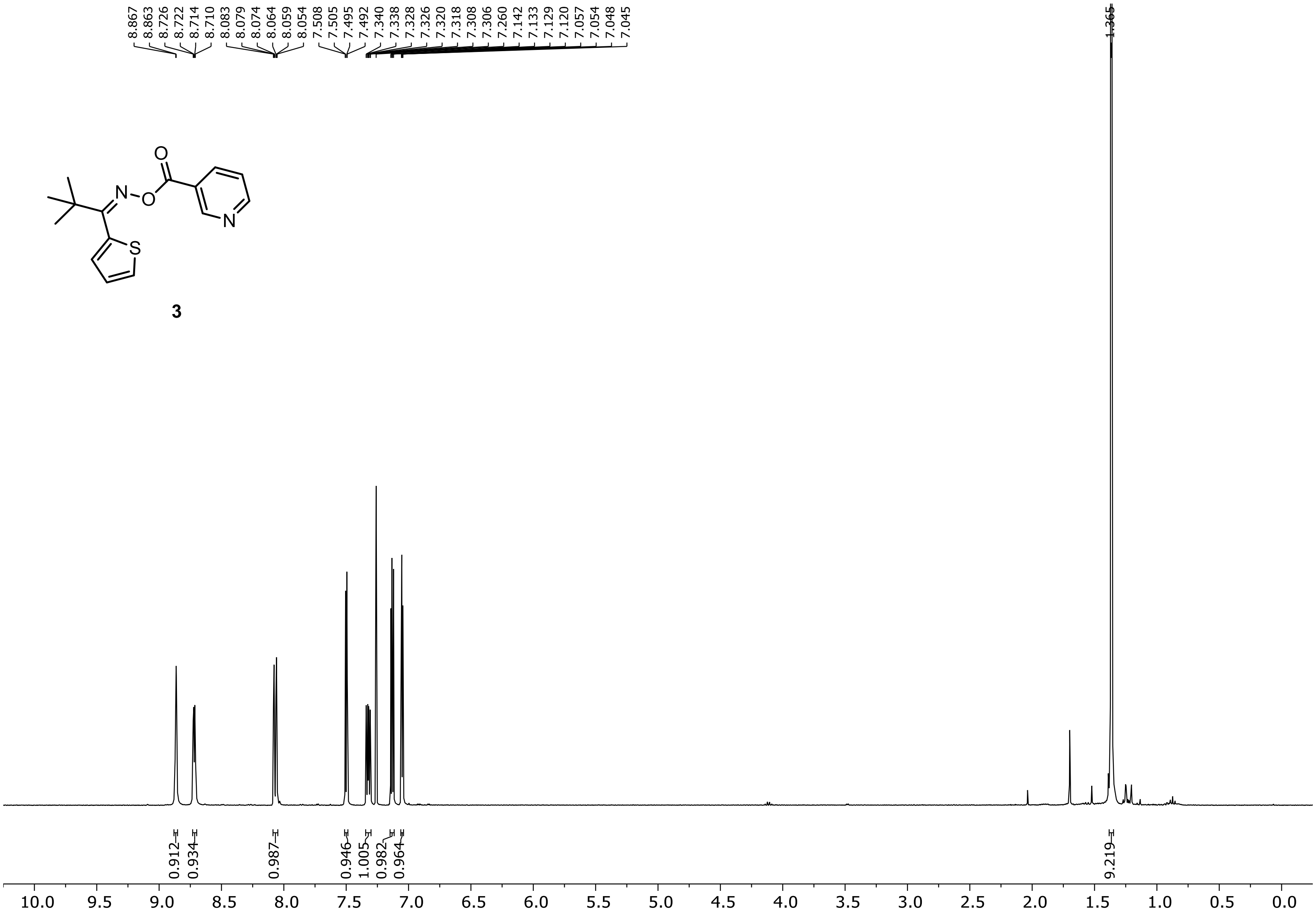

(*Z*)-2-Pivaloylthiophene *O*-(pyridine-3-oyl) oxime (**3**) (^{13}C NMR; 101 MHz; $CDCl_3$)

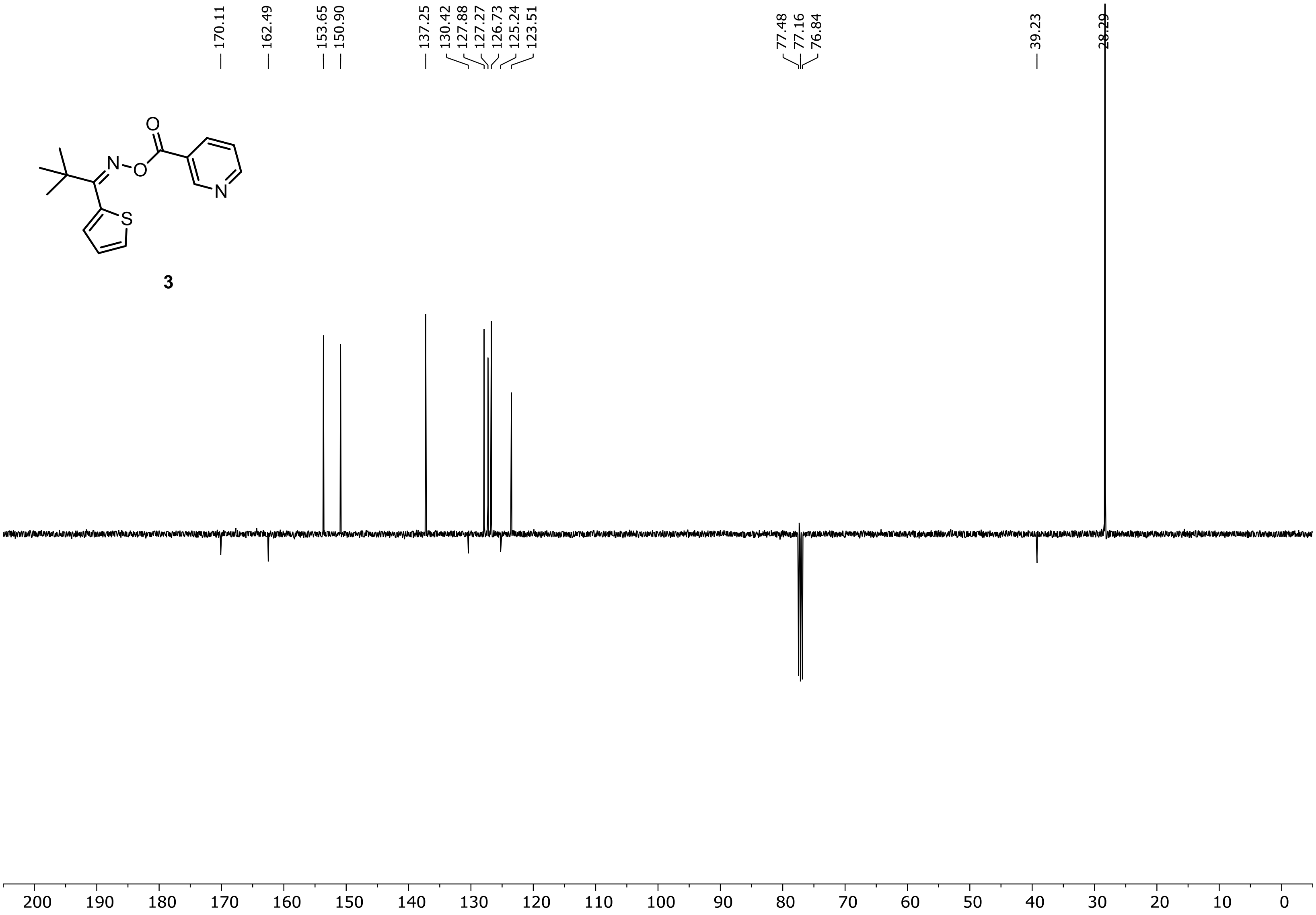

(*Z*)-2-Pivaloylthiophene *O*-(thiazol-5-ylcarbonyl) oxime (**4**) (^{1}H NMR; 400 MHz; $CDCl_3$)

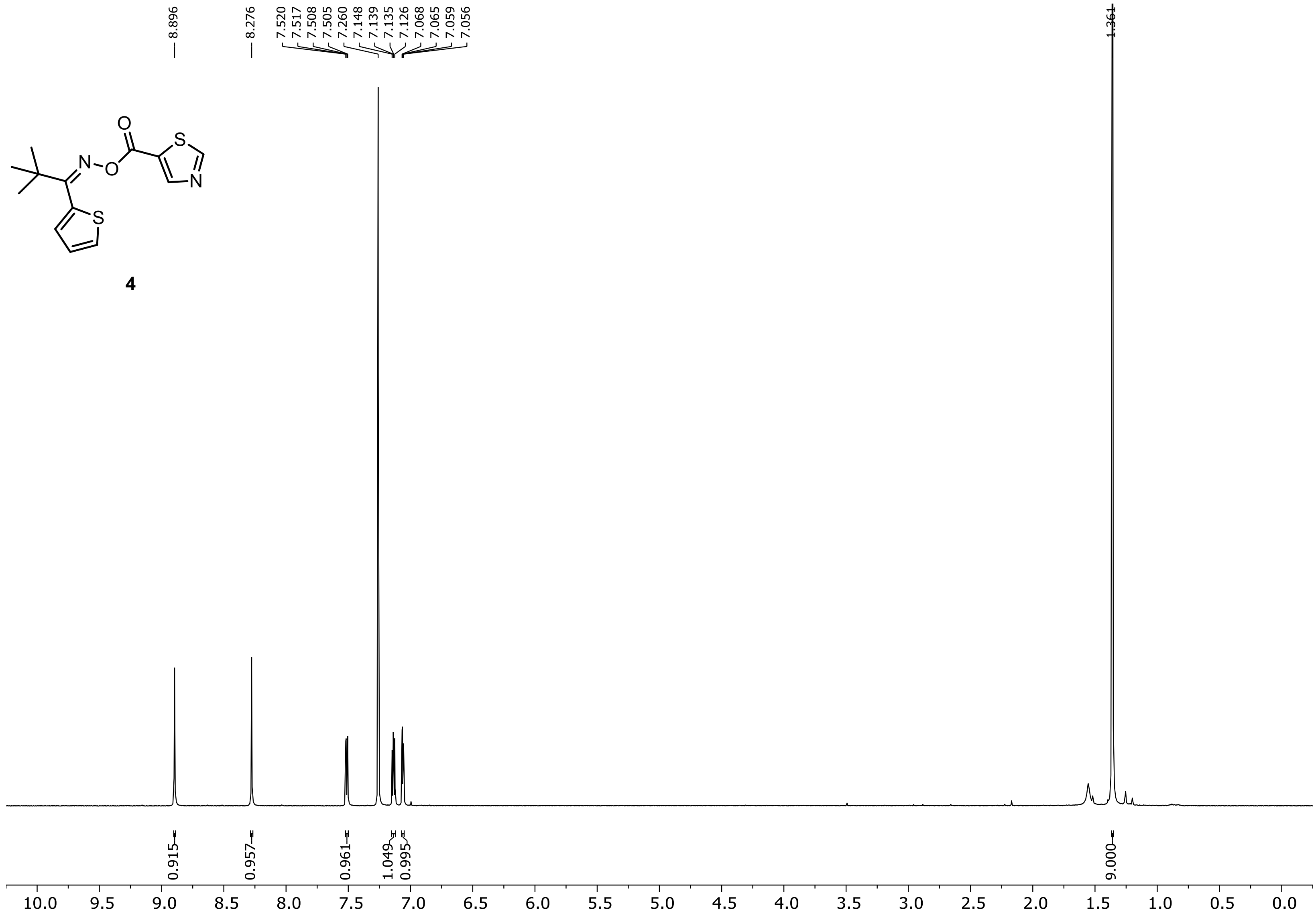

(*Z*)-2-Pivaloylthiophene *O*-(thiazol-5-ylcarbonyl) oxime (**4**) (^{13}C NMR; 101 MHz; $CDCl_3$)

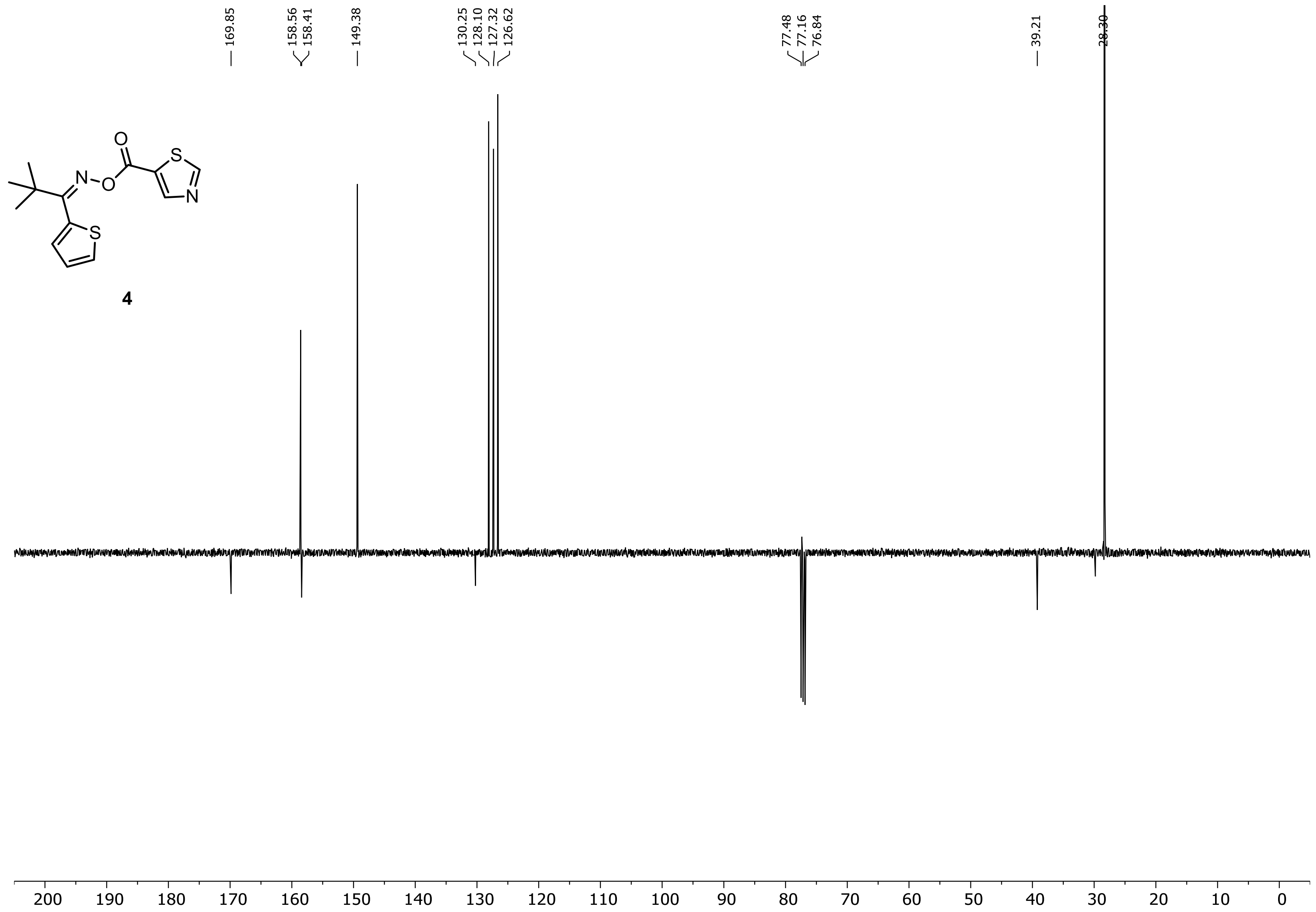

(*Z*)-2-Pivaloylthiophene *O*-(thiazol-5-ylcarbonyl) oxime (**4**) (^{13}C NMR; 101 MHz; $CDCl_3$)

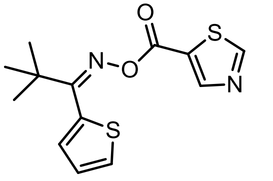

4

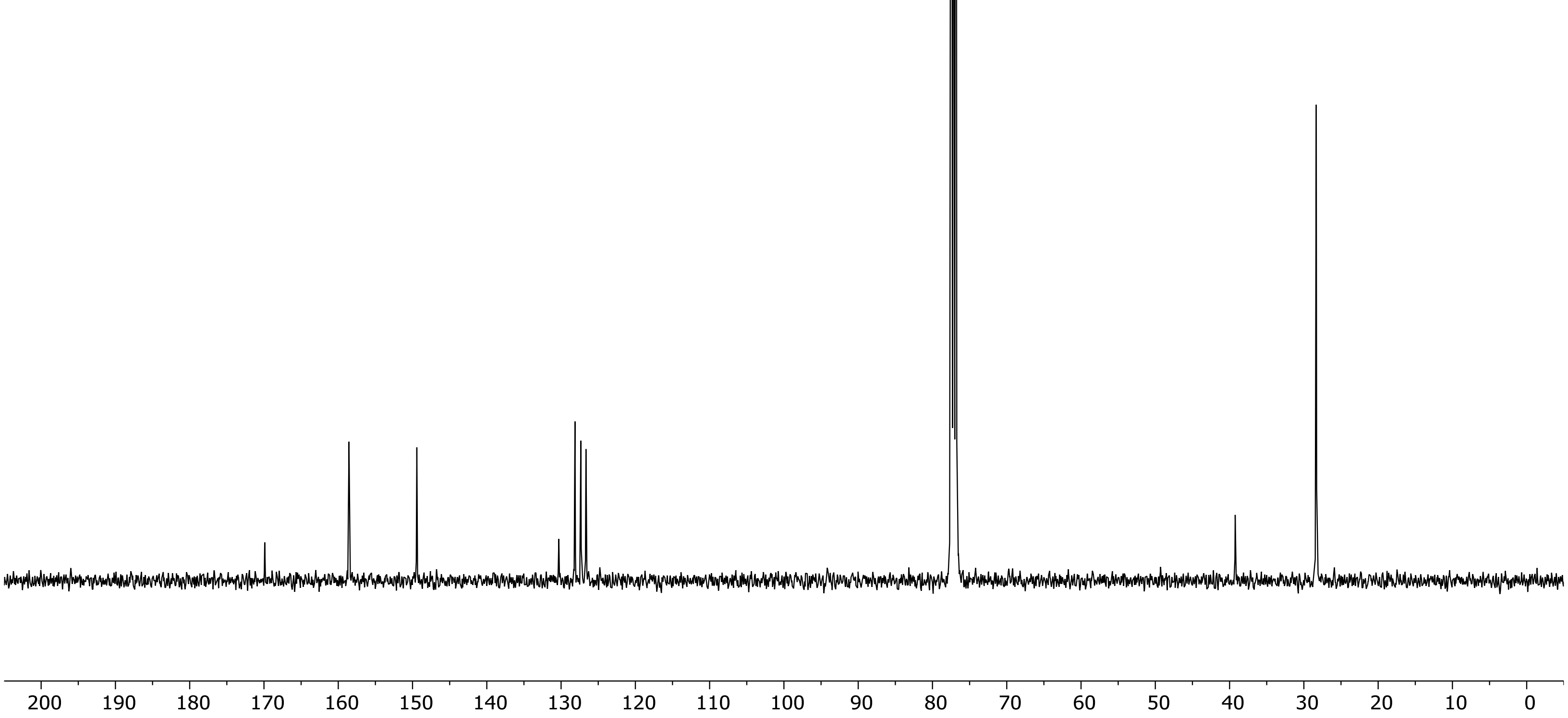

(*Z*)-2-Pivaloylthiophene *O*-(1-methyl-1*H*-imidazol-5-oyl) oxime (**5**) (^{1}H NMR; 400 MHz; $CDCl_3$)

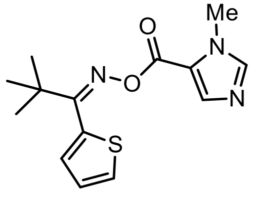

5

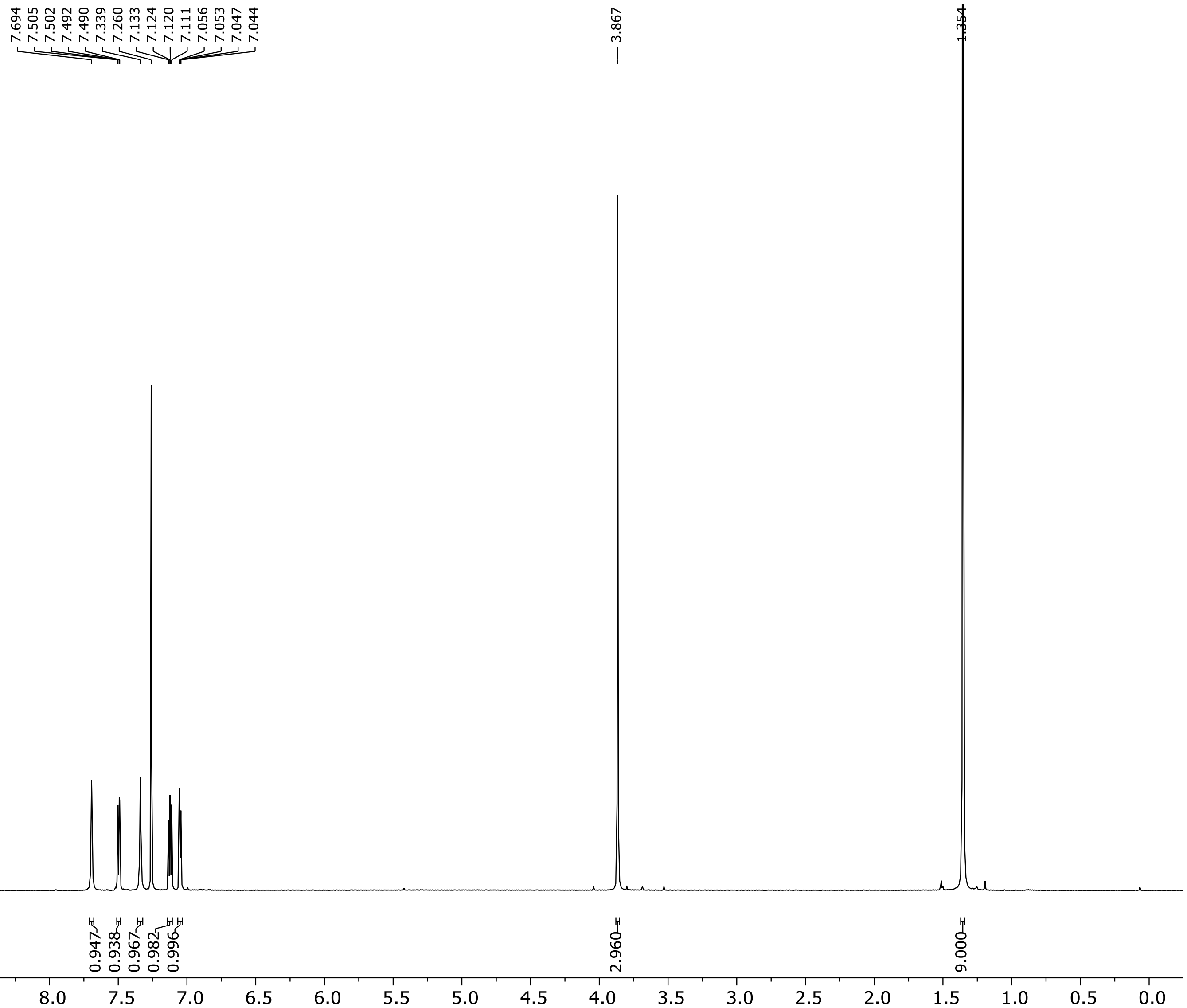

(*Z*)-2-Pivaloylthiophene *O*-(1-methyl-1*H*-imidazol-5-oyl) oxime (**5**) (^{13}C NMR; 101 MHz; $CDCl_3$)

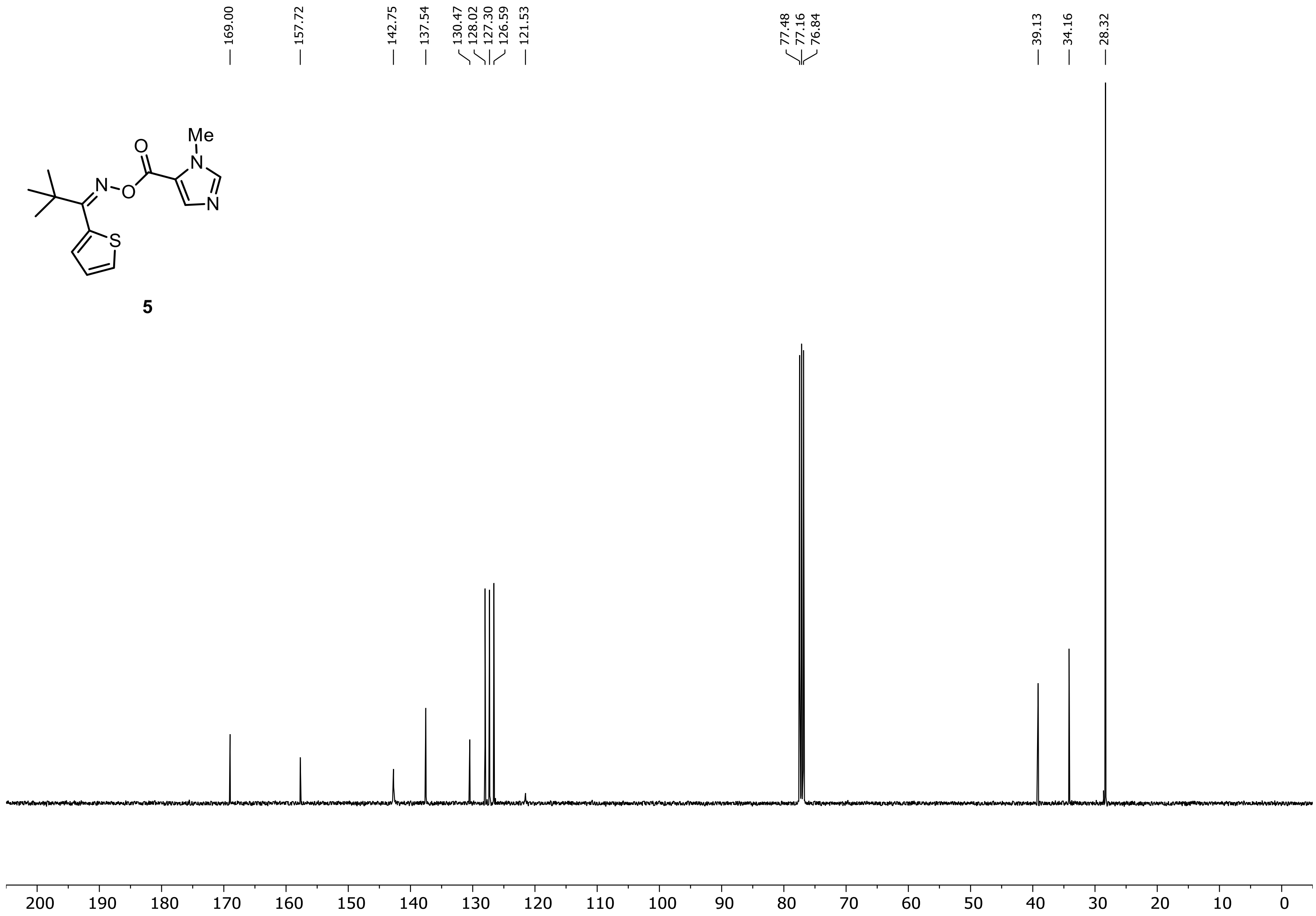

(*Z*)-2-Pivaloylthiophene *O*-(3-amino-4-methoxybenzoyl) oxime (**6**) (^{1}H NMR; 400 MHz; $CDCl_3$)

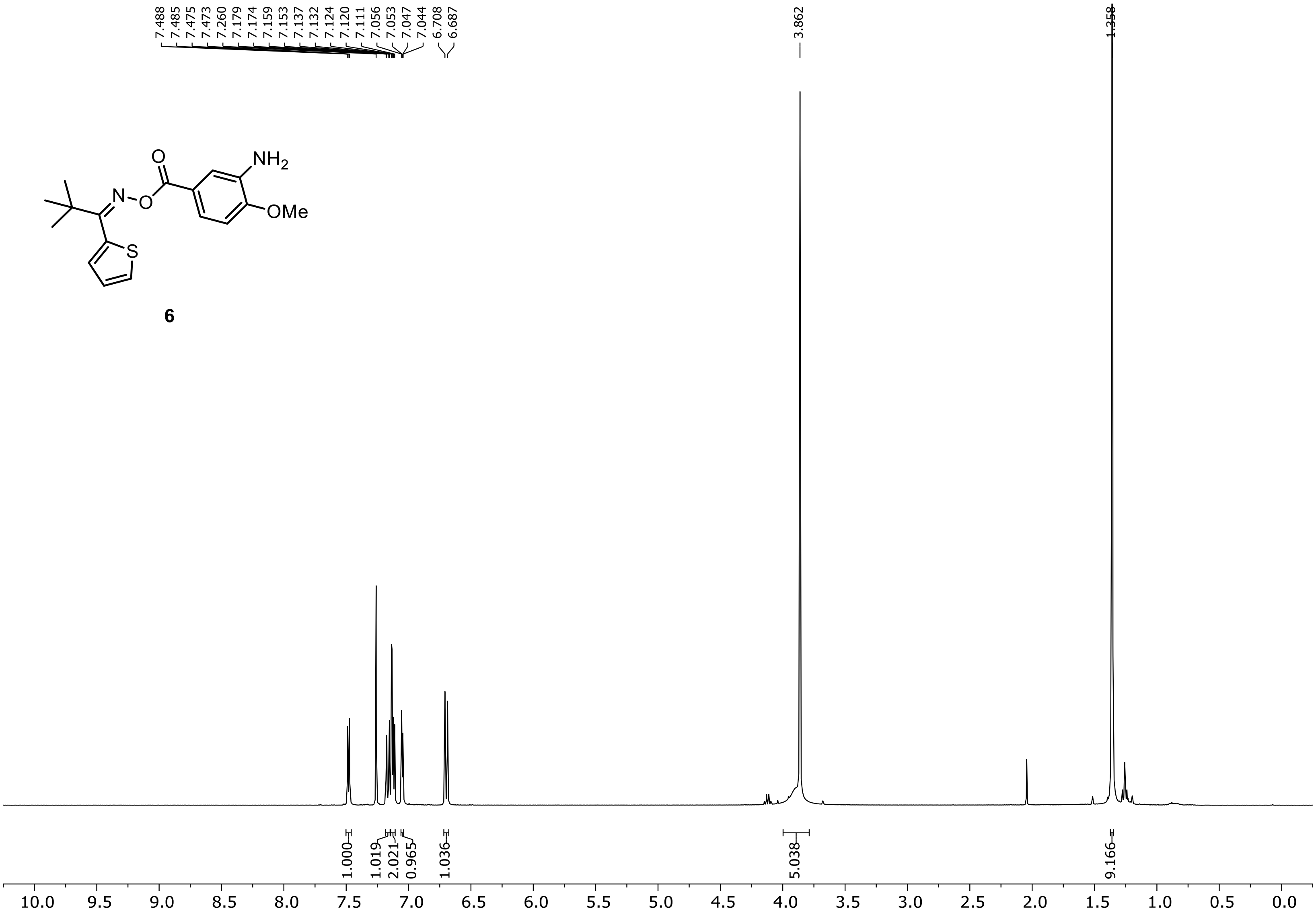

(*Z*)-2-Pivaloylthiophene *O*-(3-amino-4-methoxybenzoyl) oxime (**6**) (^{13}C NMR; 101 MHz; $CDCl_3$)

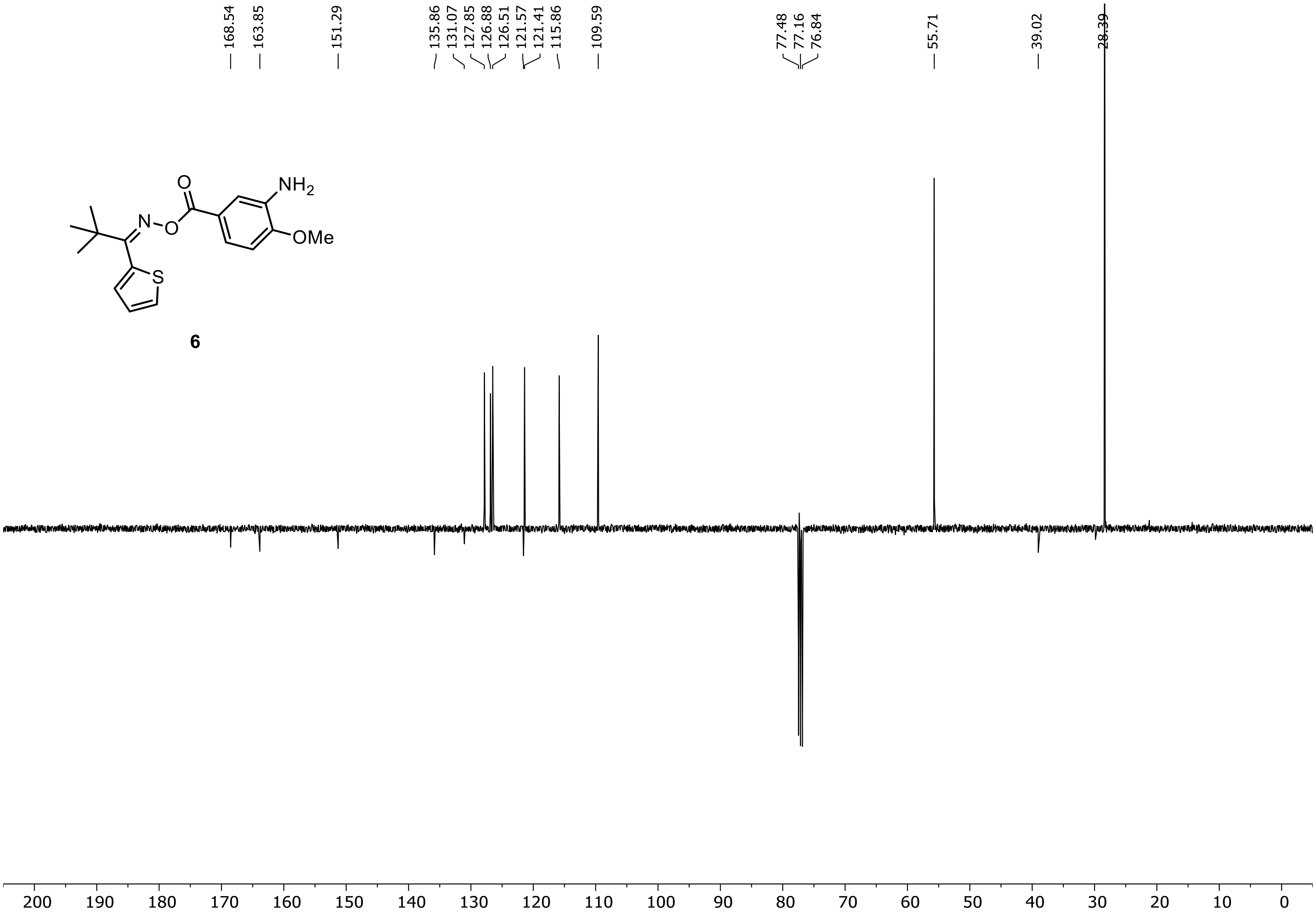

(*Z*)-2-Pivaloylthiophene *O*-(3,5-dimethoxybenzoyl) oxime (**7**) (^{1}H NMR; 400 MHz; $CDCl_3$)

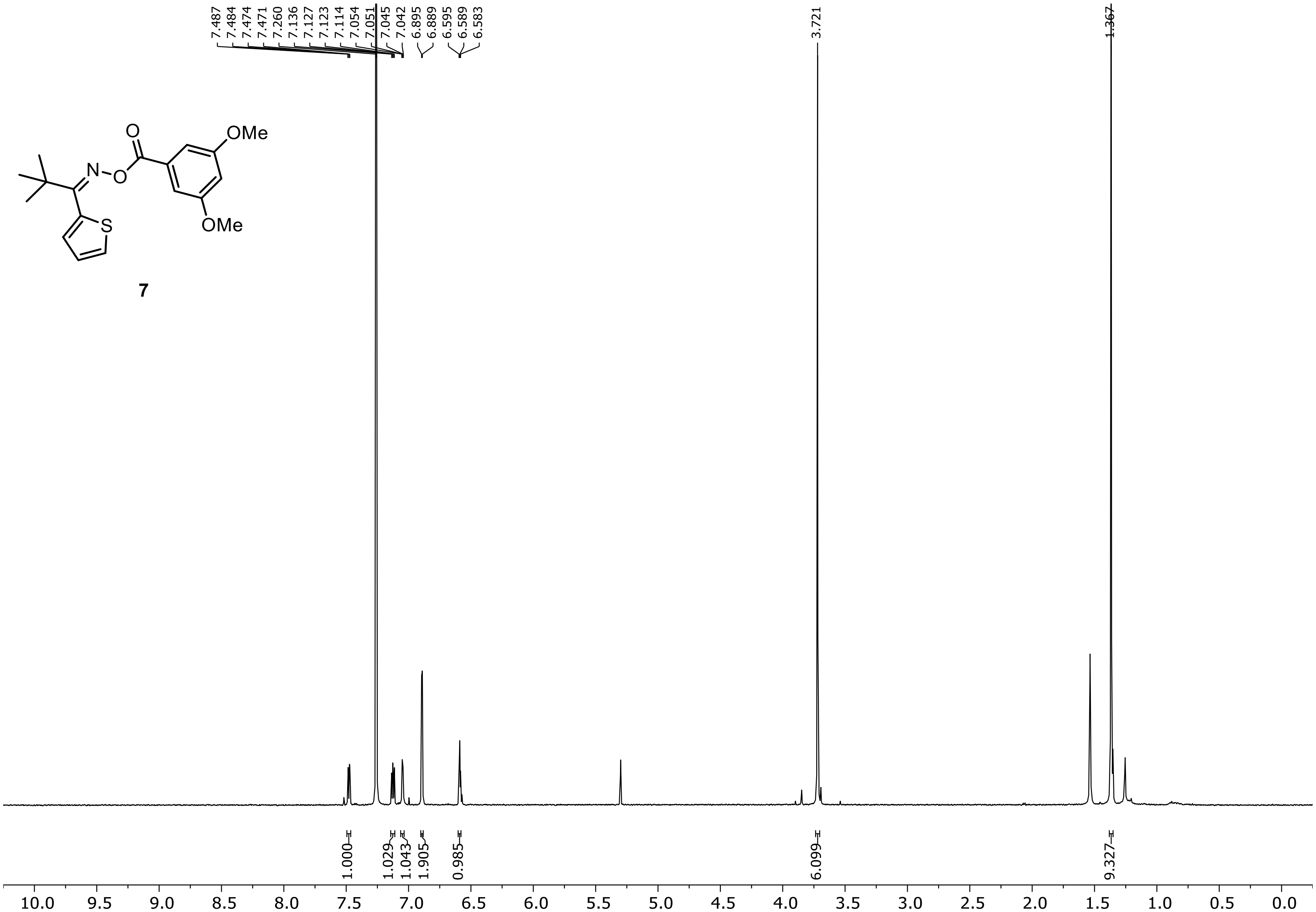

(*Z*)-2-Pivaloylthiophene *O*-(3,5-dimethoxybenzoyl) oxime (**7**) (^{13}C NMR; 101 MHz; $CDCl_3$)

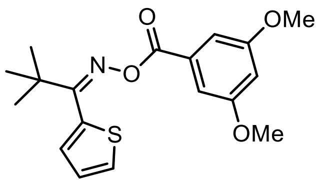

7

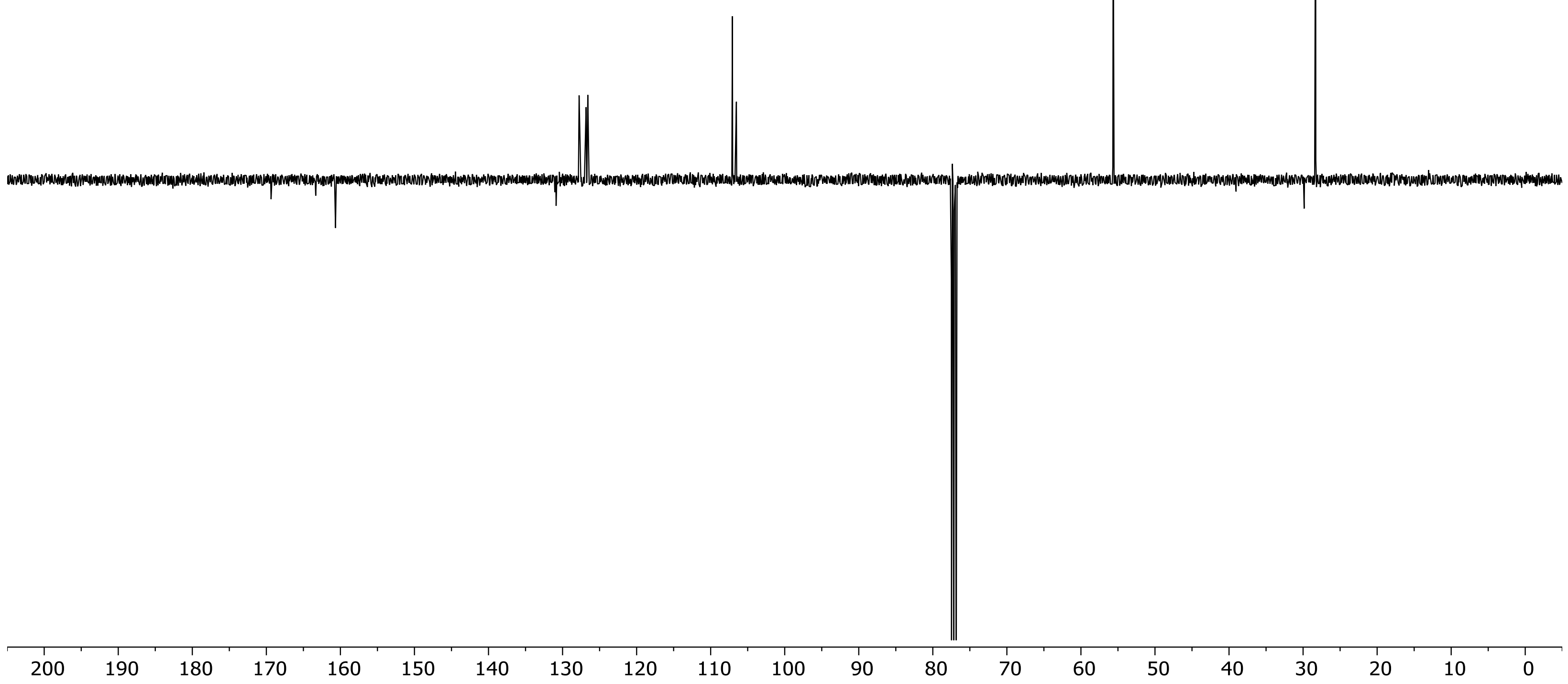

(*Z*)-2-Pivaloylthiophene *O*-(4-chlorobenzoyl) oxime (**8**) (^{1}H NMR; 400 MHz; $CDCl_3$)

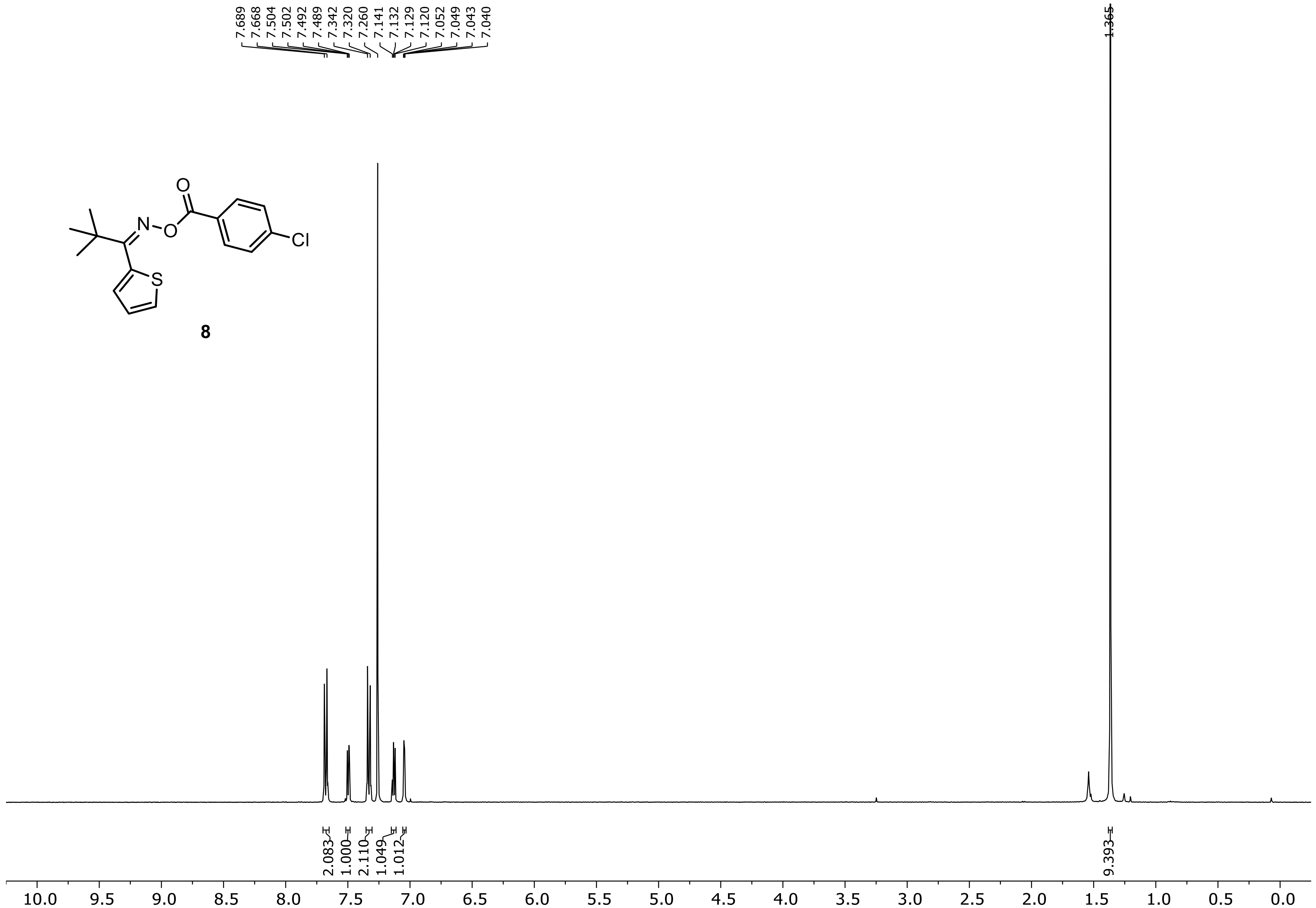

(*Z*)-2-Pivaloylthiophene *O*-(4-chlorobenzoyl) oxime (**8**) (^{13}C NMR; 101 MHz; $CDCl_3$)

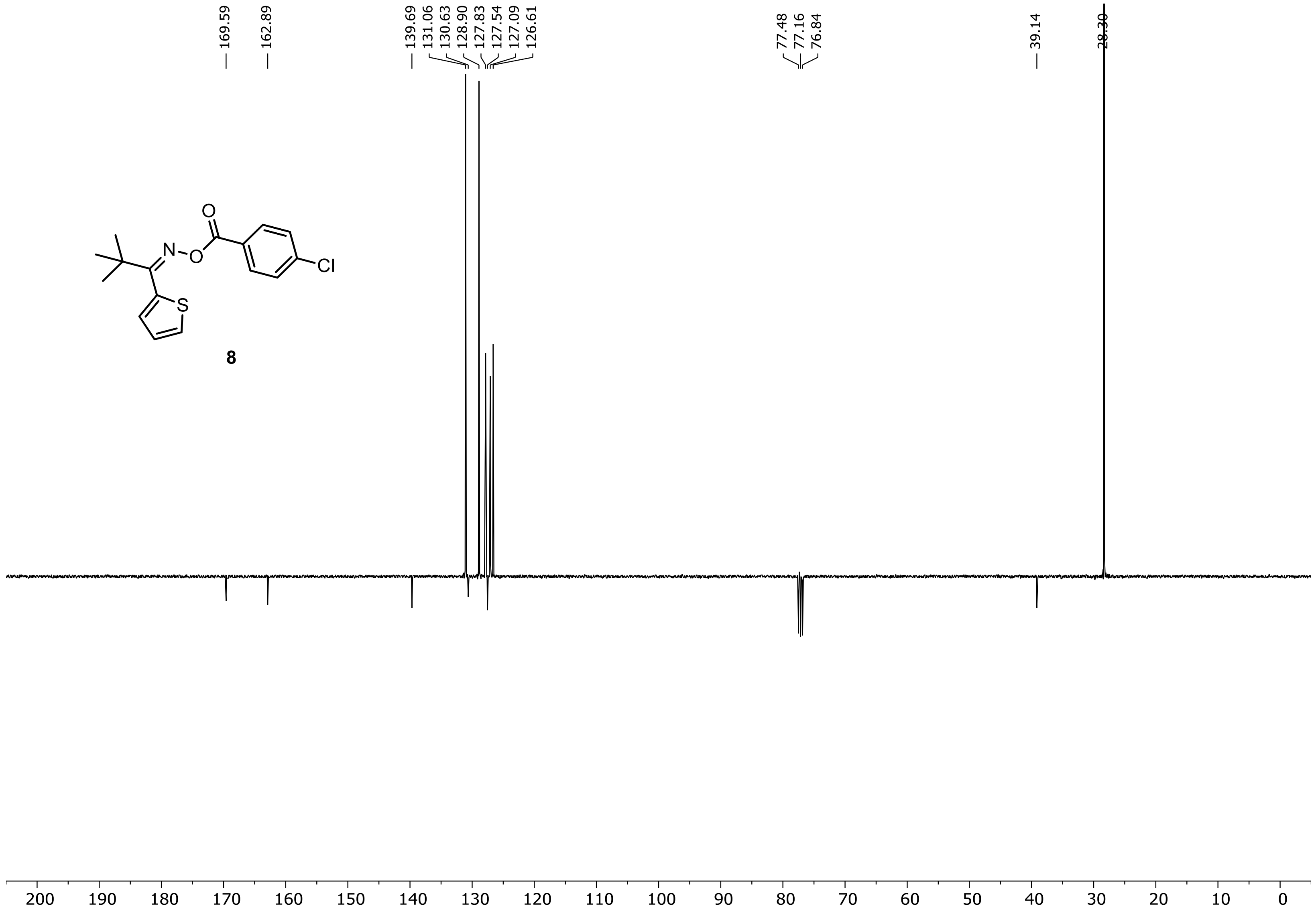

(*Z*)-2-Pivaloylthiophene *O*-(4-trifluoromethylbenzoyl) oxime (**9**) (^{1}H NMR; 400 MHz; $CDCl_3$)

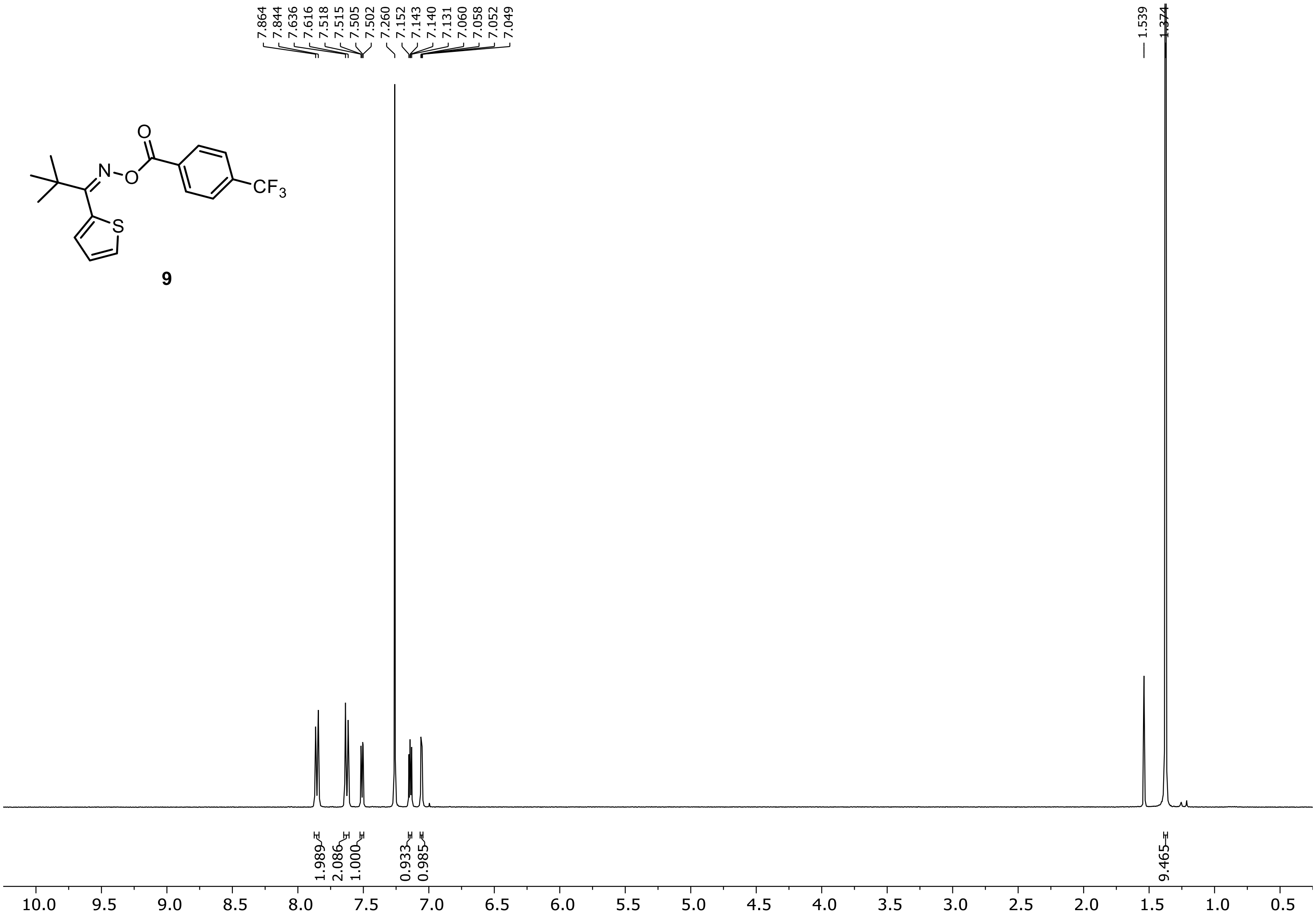

(*Z*)-2-Pivaloylthiophene *O*-(4-trifluoromethylbenzoyl) oxime (**9**) (^{13}C NMR; 101 MHz; $CDCl_3$)

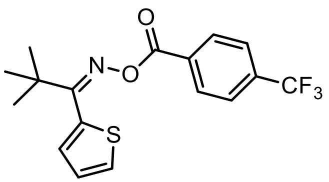

9

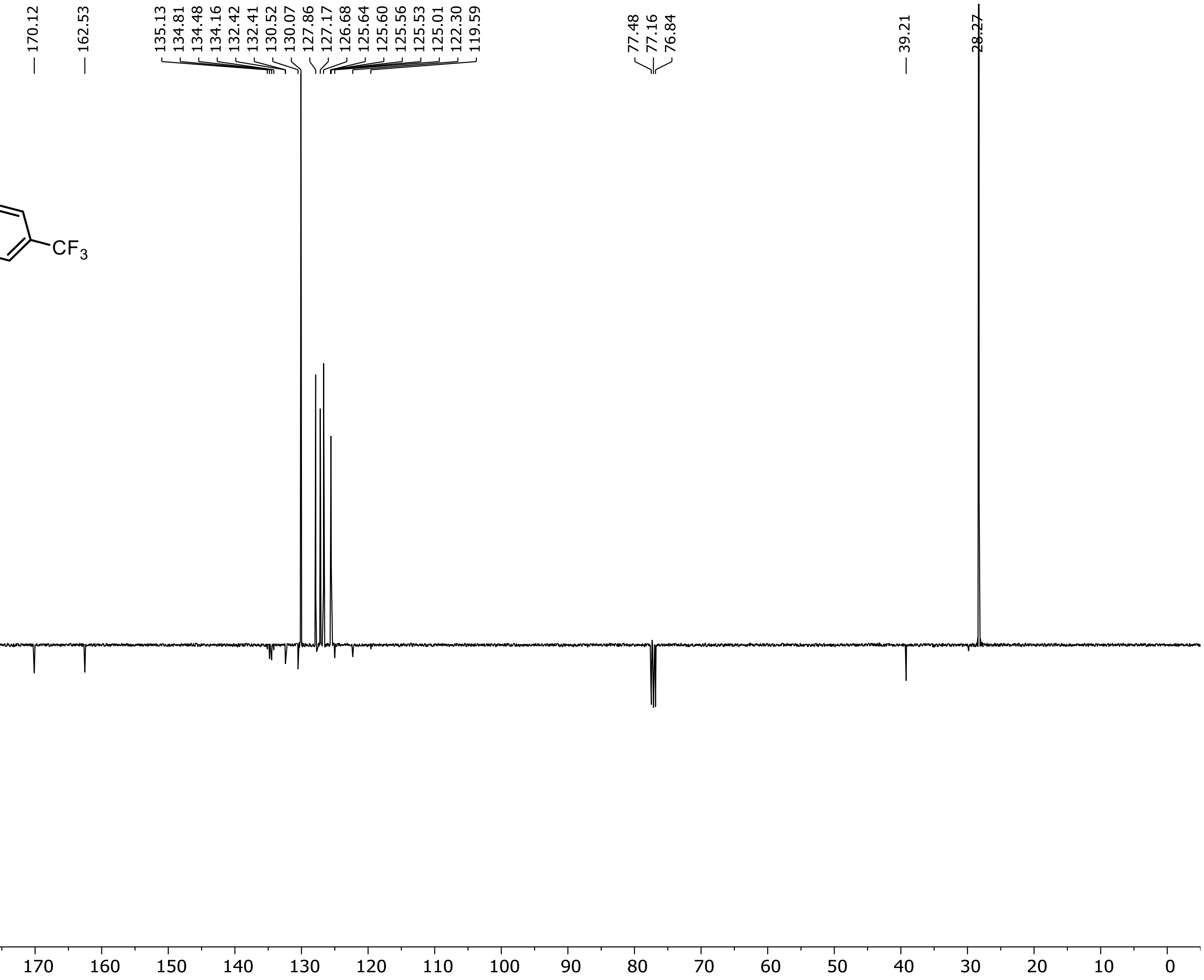

(*Z*)-2-Pivaloylthiophene *O*-(4-trifluoromethylbenzoyl) oxime (**9**) (^{19}F NMR; 377 MHz, $CDCl_3$ + $CFCl_3$)

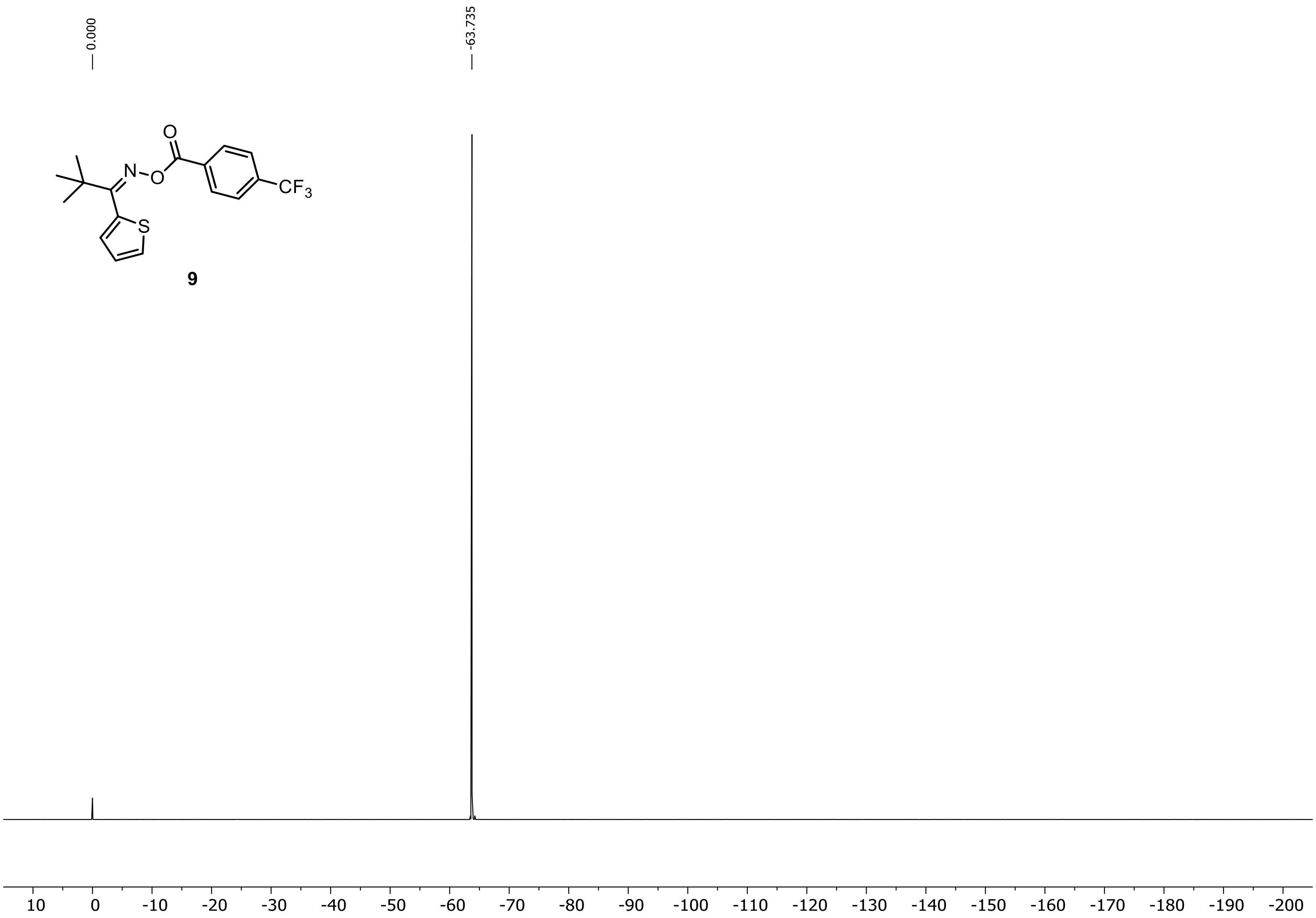

(*Z*)-2-Pivaloylthiophene *O*-(4-methylaminobenzoyl) oxime (**10**) (^{1}H NMR; 400 MHz; $CDCl_3$)

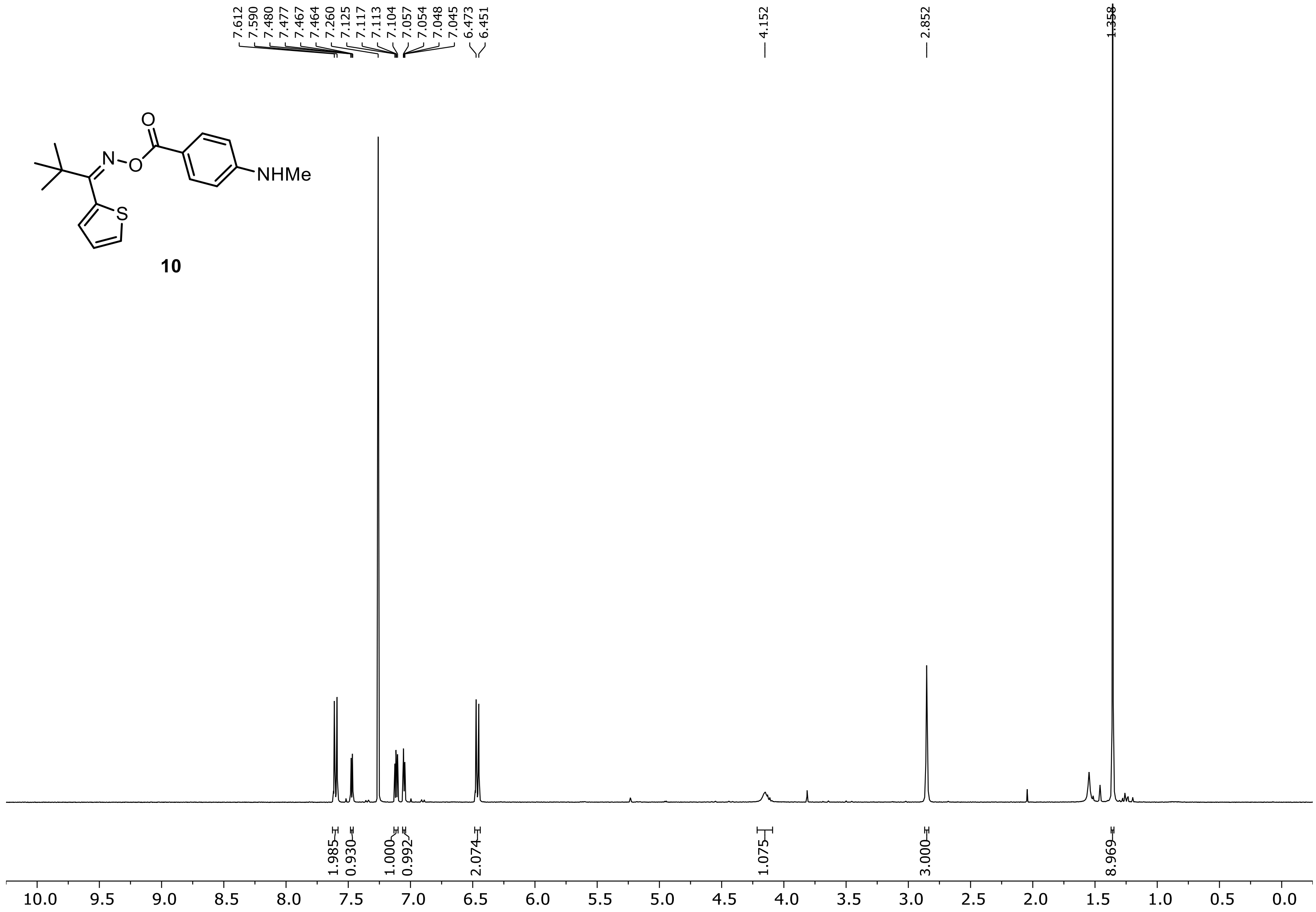

(*Z*)-2-Pivaloylthiophene *O*-(4-methylaminobenzoyl) oxime (**10**) (^{13}C NMR; 101 MHz; $CDCl_3$)

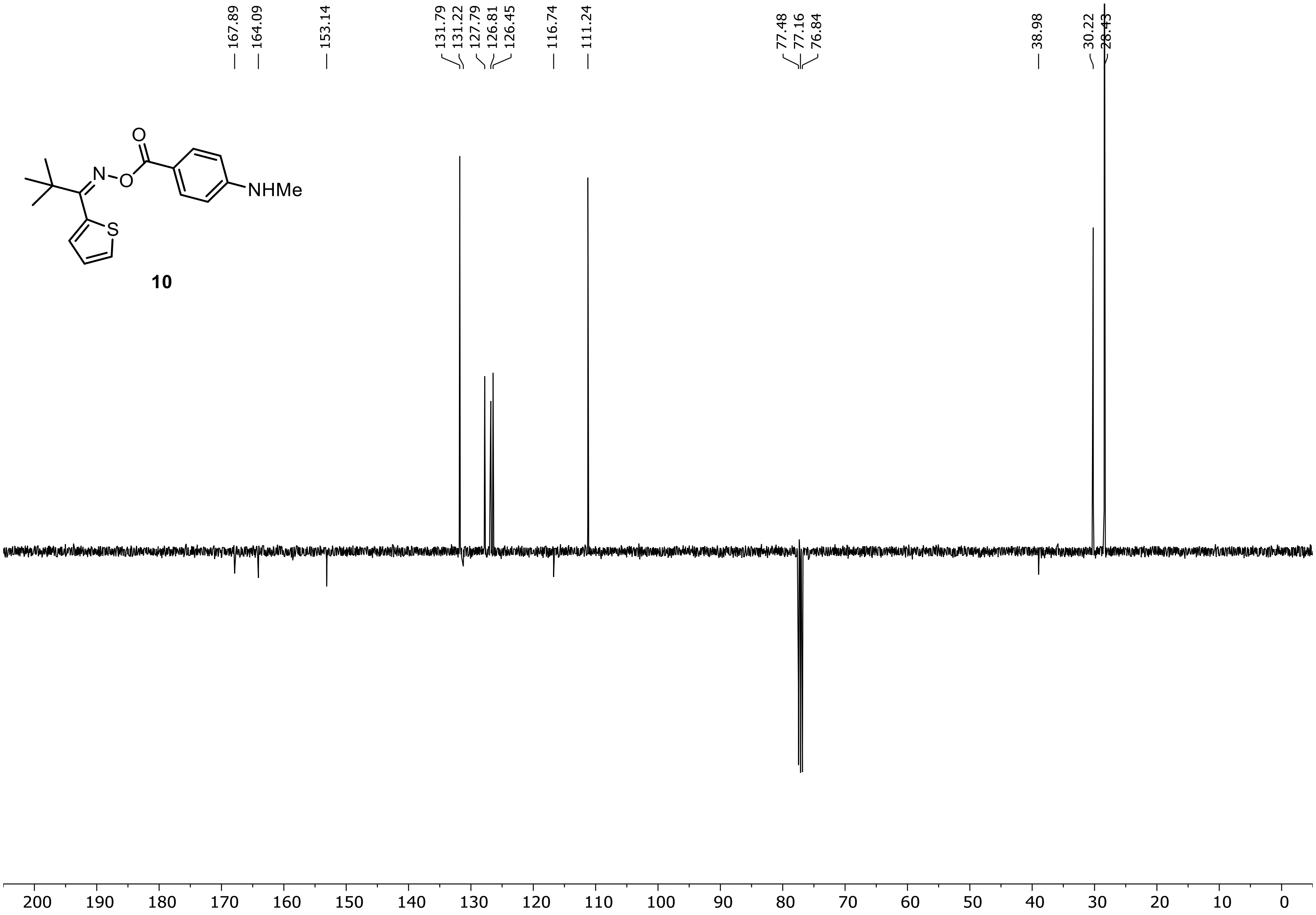

(*Z*)-2-Pivaloylthiophene *O*-benzoyl oxime (**11**) (^{1}H NMR; 400 MHz; $CDCl_3$)

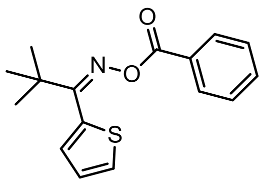

11

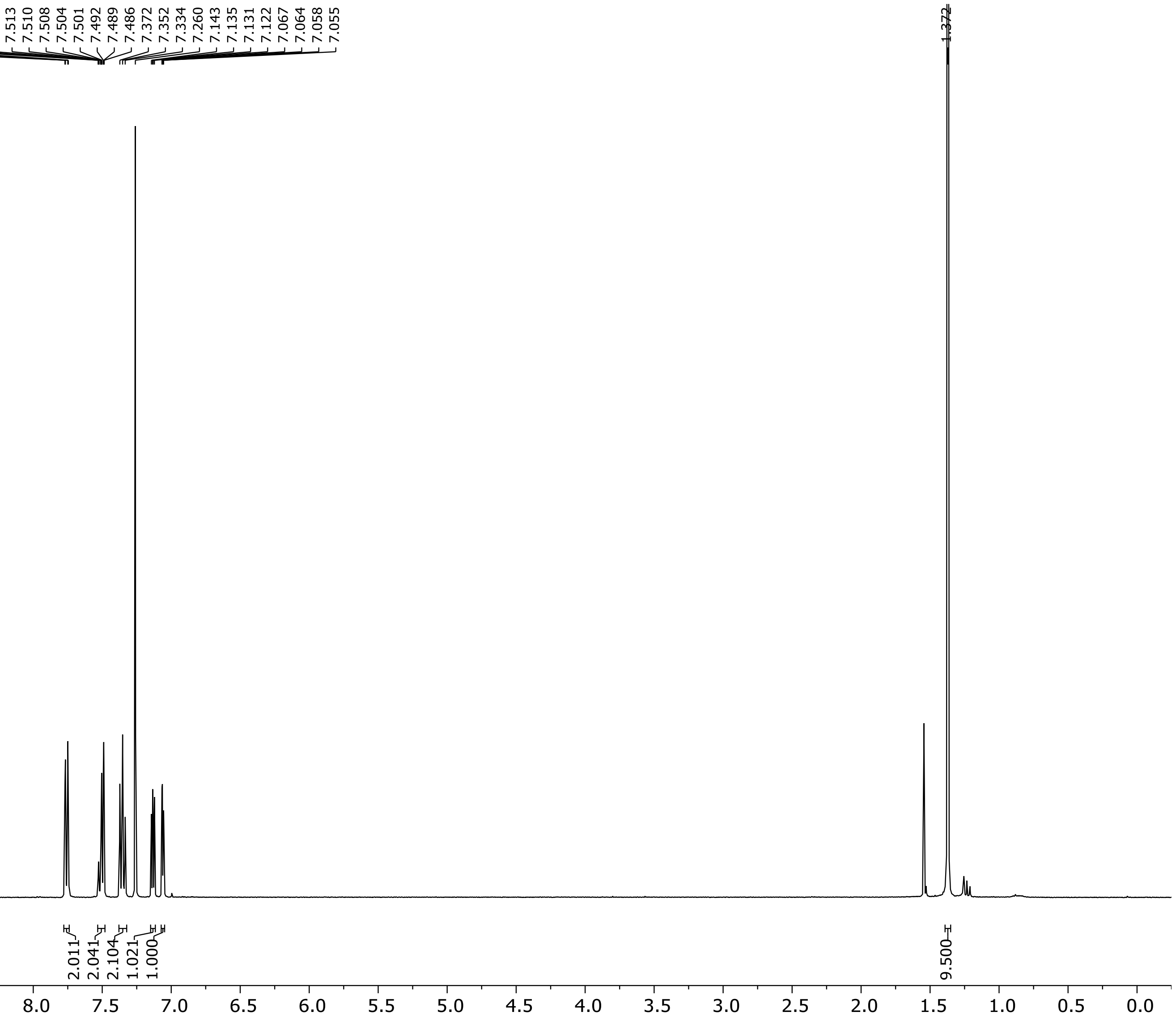

(*Z*)-2-Pivaloylthiophene *O*-benzoyl oxime (**11**) (^{13}C NMR; 101 MHz; $CDCl_3$)

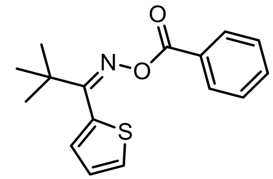

11

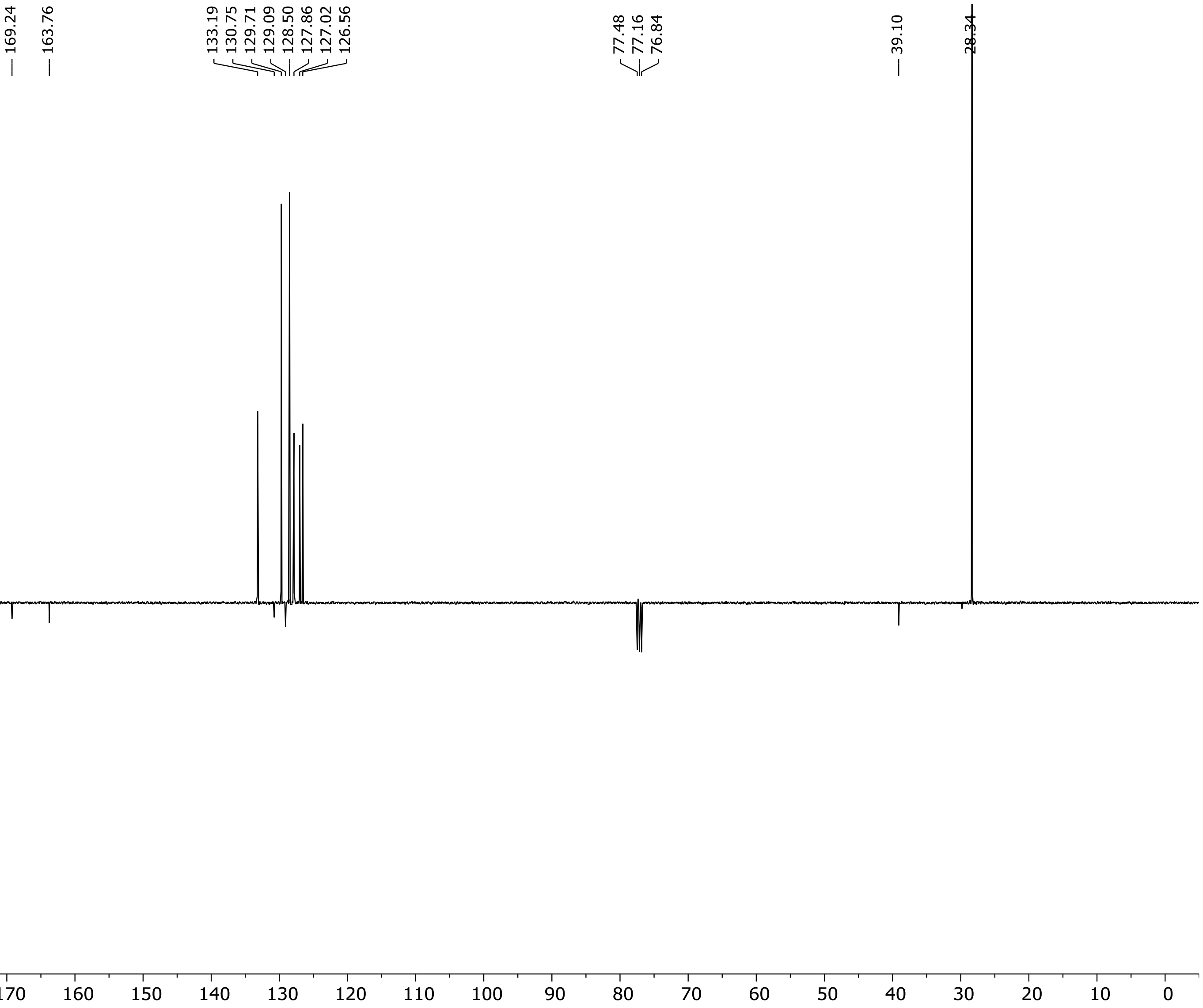

(*Z*)-2-Pivaloylthiophene *O*-picolinoyl oxime (**12**) (^{1}H NMR; 400 MHz; $CDCl_3$)

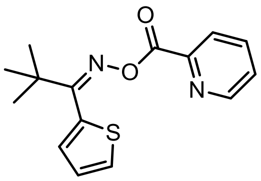

12

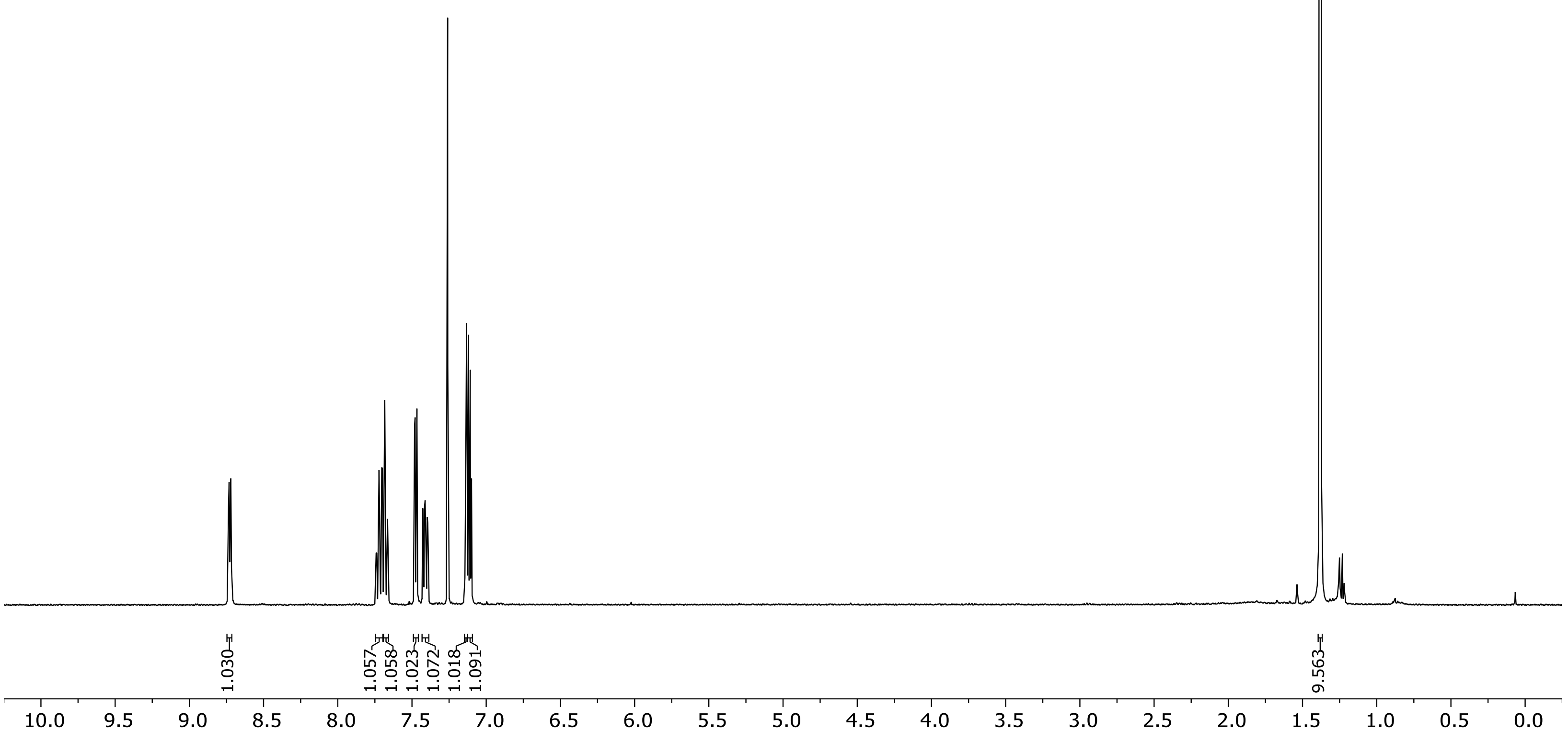

(*Z*)-2-Pivaloylthiophene *O*-picolinoyl oxime (**12**) (^{13}C NMR; 101 MHz; $CDCl_3$)

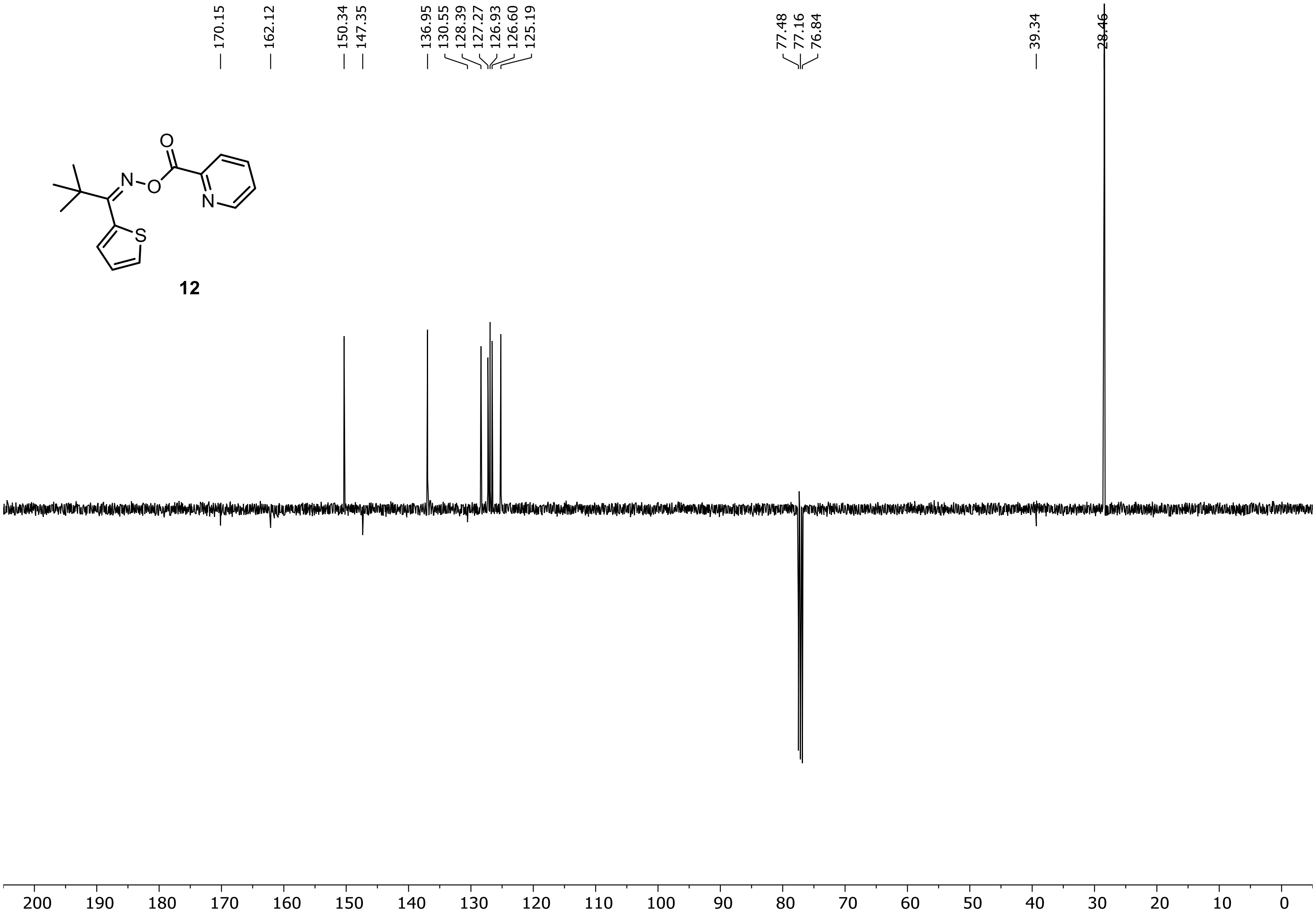

(*Z*)-2-Pivaloylthiophene *O*-(6-methoxynicotinoyl) oxime (**13**) (^{1}H NMR; 400 MHz; $CDCl_3$)

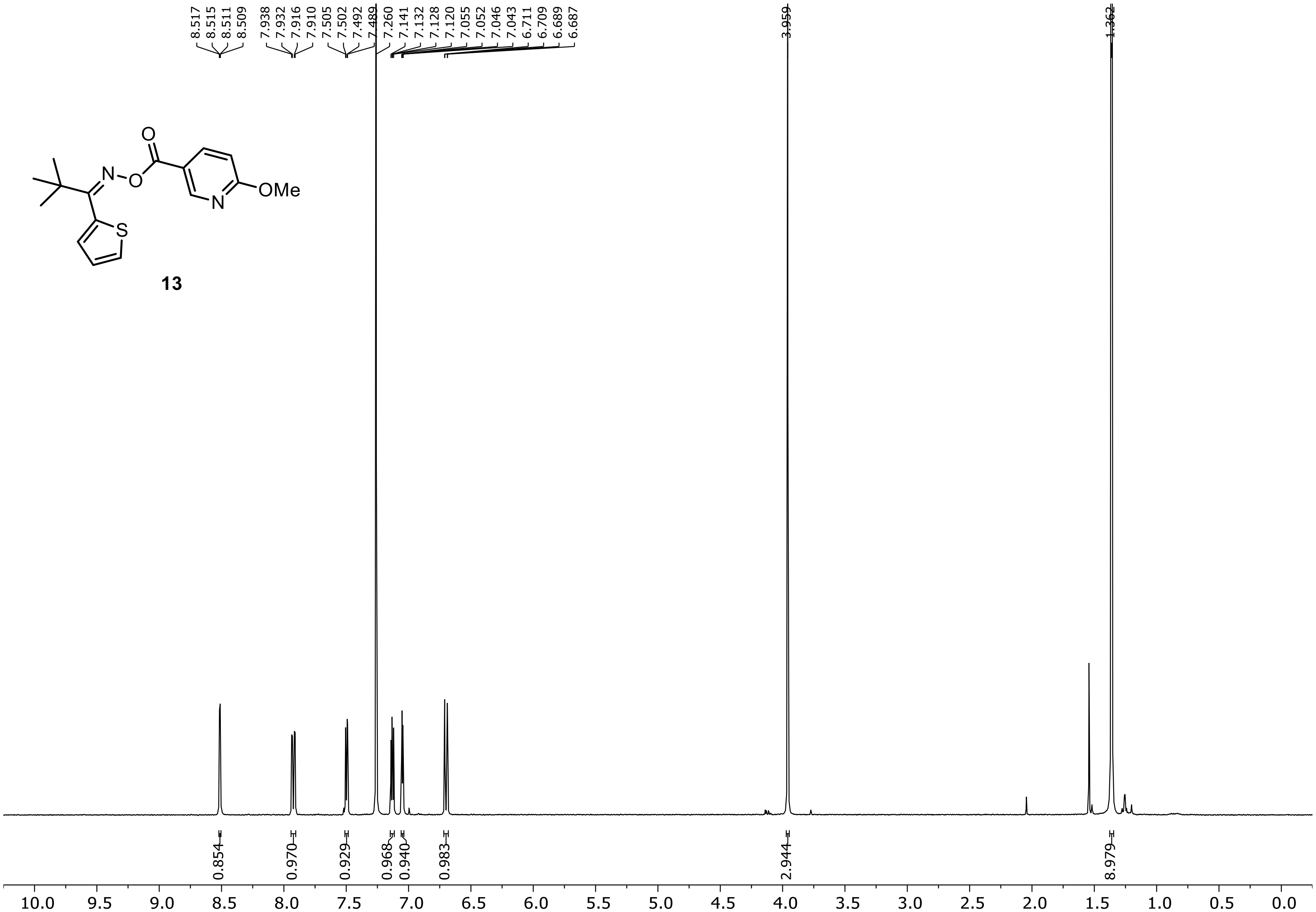

(*Z*)-2-Pivaloylthiophene *O*-(6-methoxynicotinoyl) oxime (**13**) (^{13}C NMR; 101 MHz; $CDCl_3$)

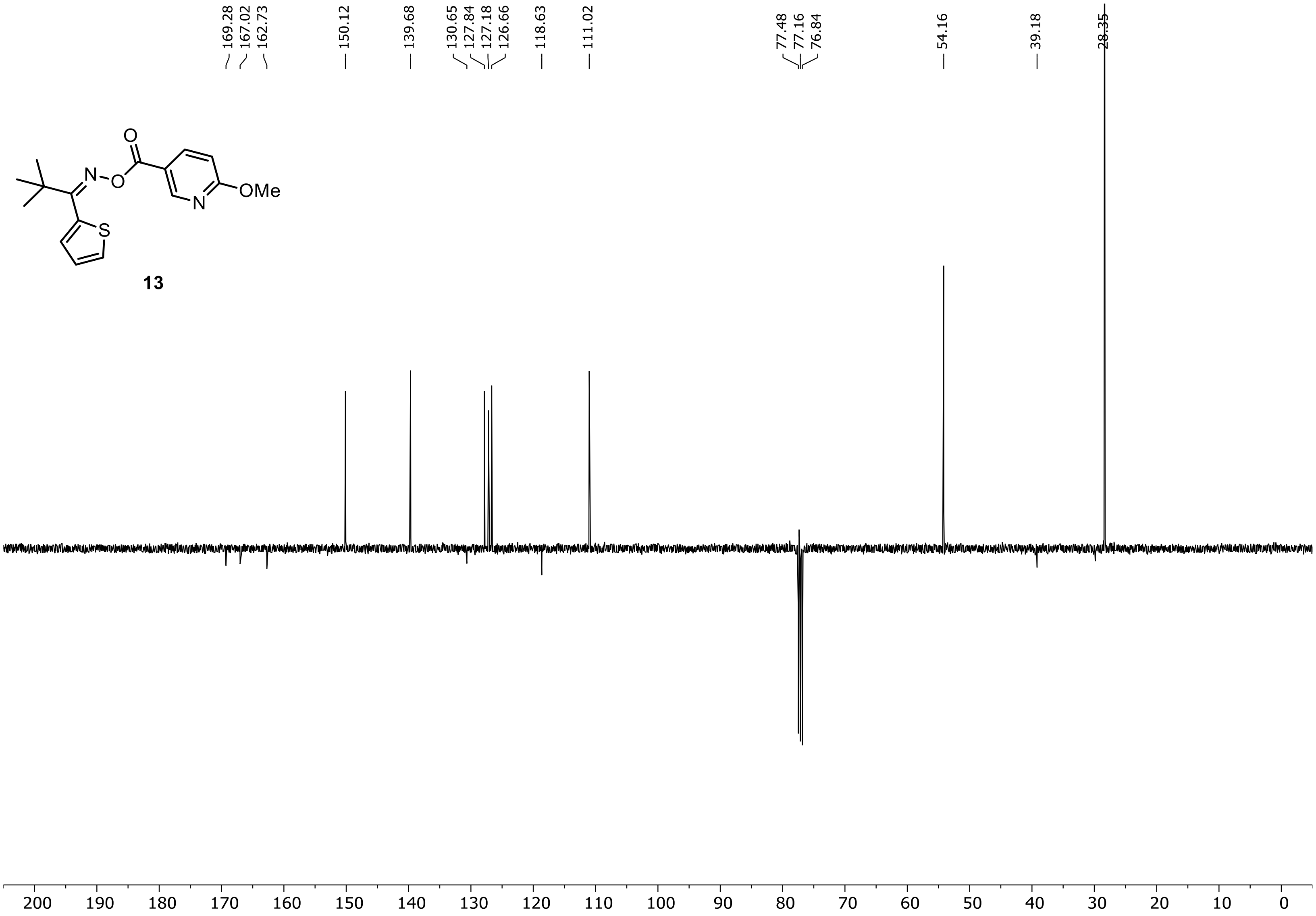

(*Z*)-2-Pivaloylthiophene *O*-(quinolin-3-oyl) oxime (**14**) (^{1}H NMR; 400 MHz; $CDCl_3$)

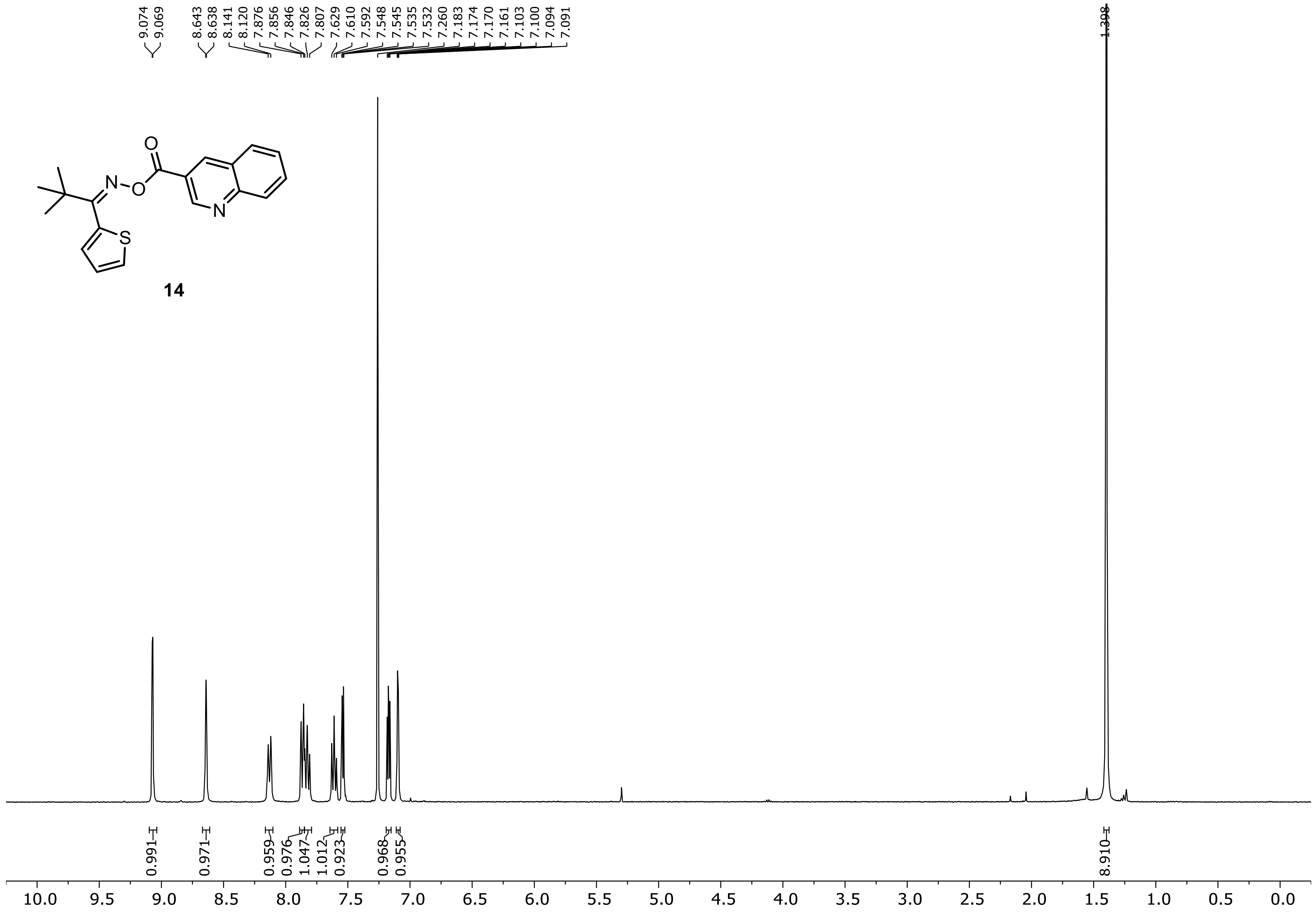

14

(*Z*)-2-Pivaloylthiophene *O*-(quinolin-3-oyl) oxime (**14**) (^{13}C NMR; 101 MHz; $CDCl_3$)

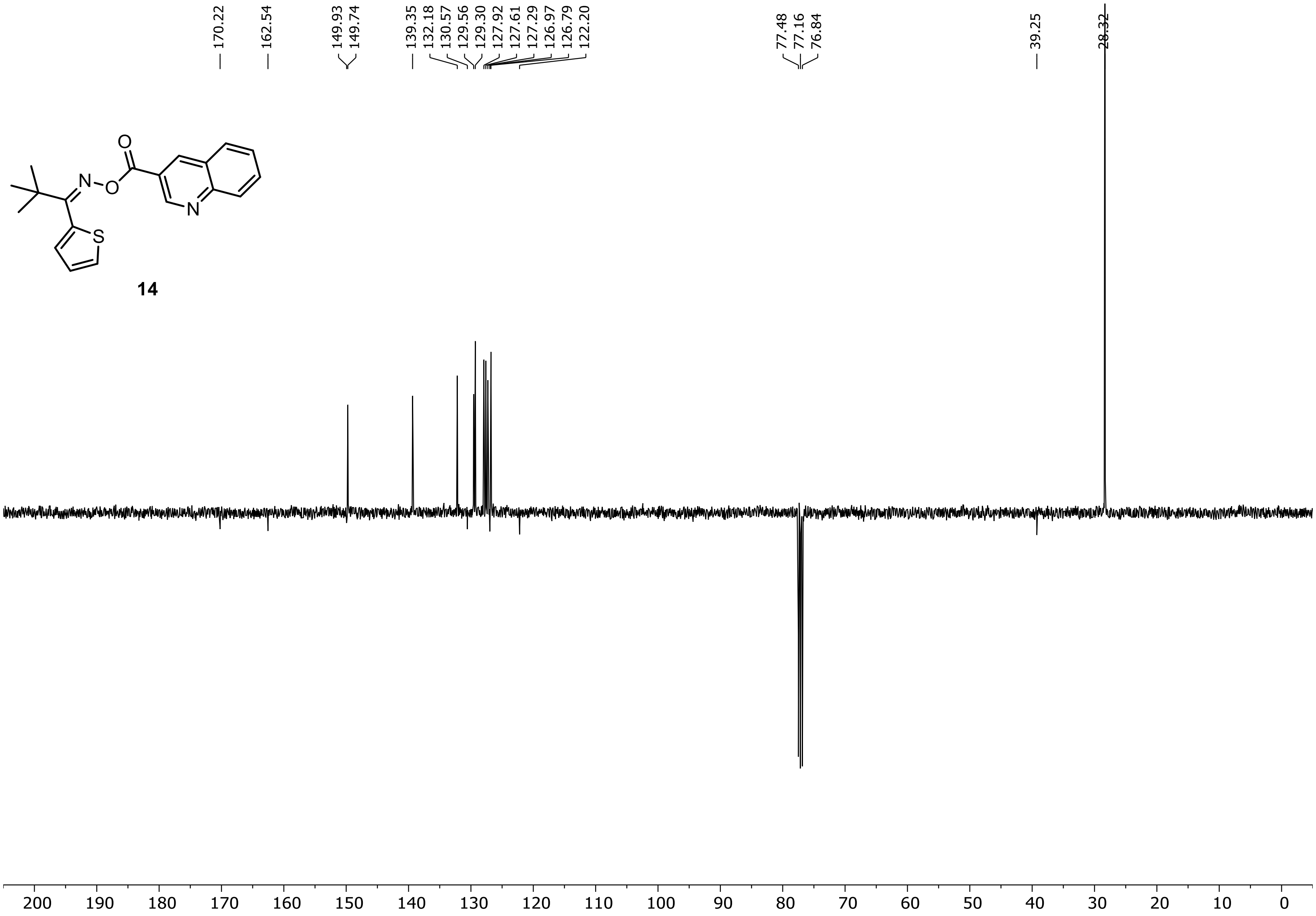

(*Z*)-2-Pivaloylthiophene *O*-(isoxazol-5-ylcarbonyl) oxime (**15**) (^{1}H NMR; 400 MHz; $CDCl_3$)

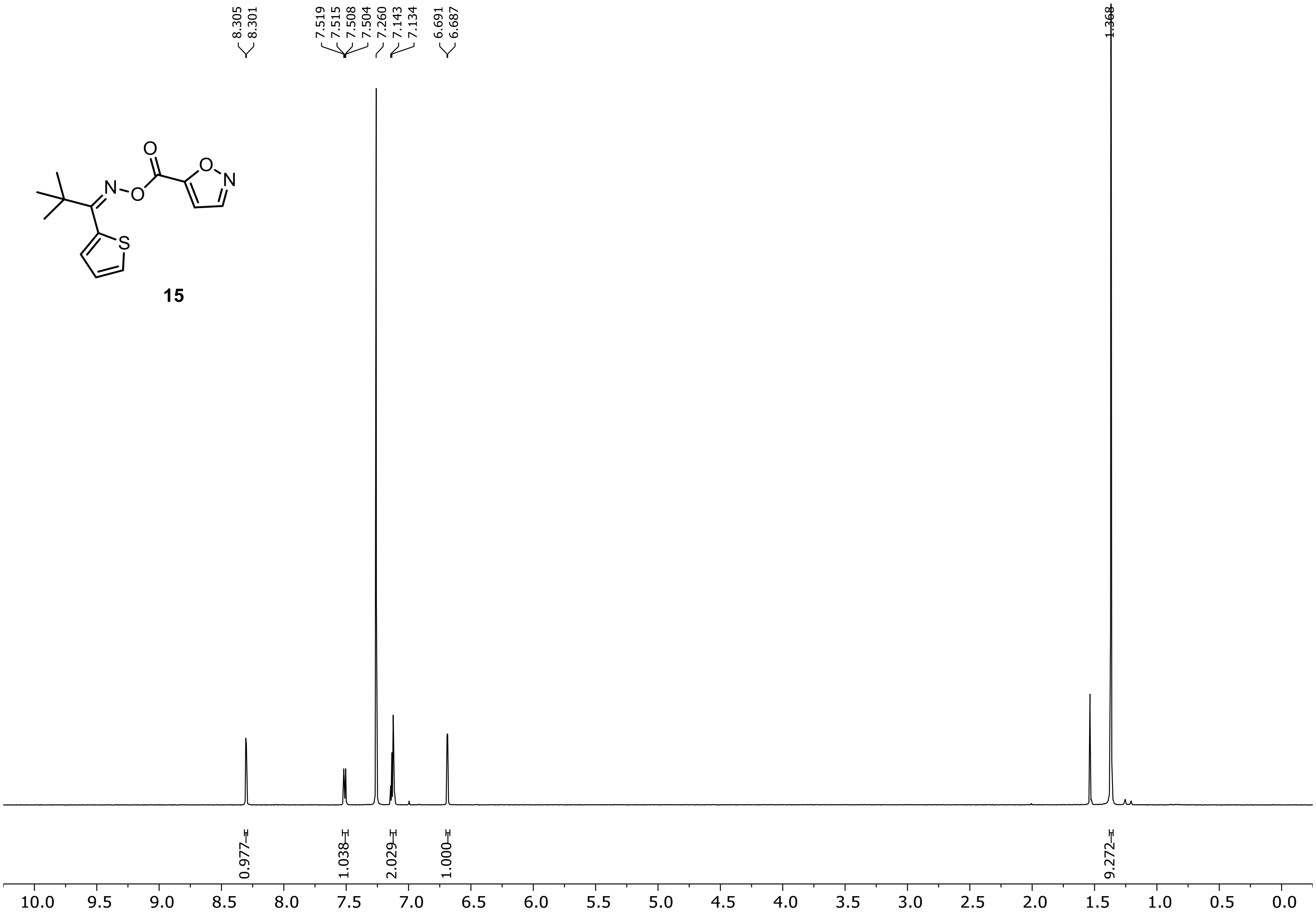

(*Z*)-2-Pivaloylthiophene *O*-(isoxazol-5-ylcarbonyl) oxime (**15**) (^{13}C NMR; 101 MHz; $CDCl_3$)

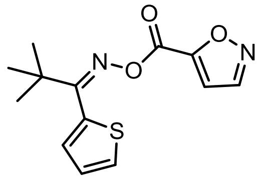

15

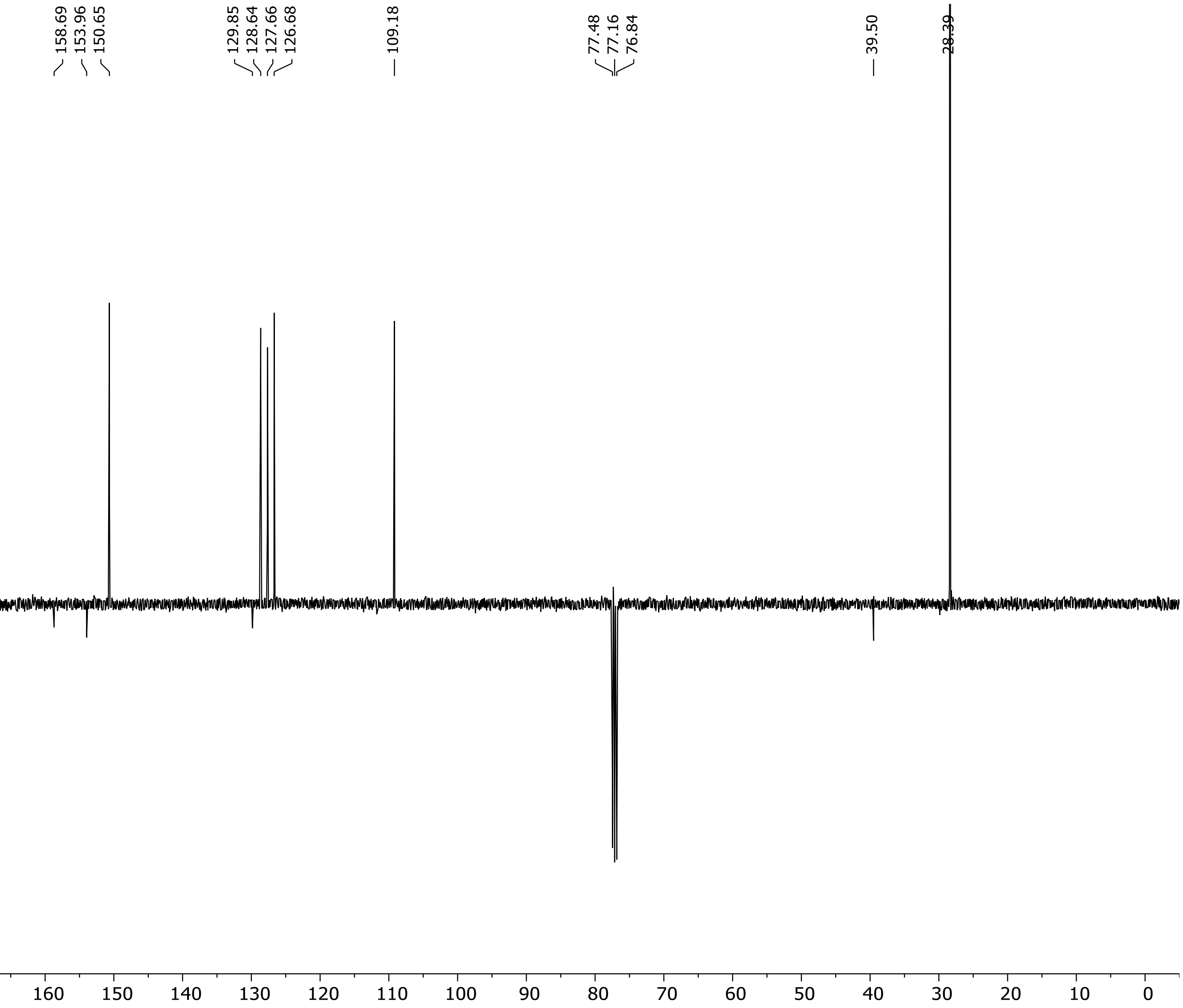

(*Z*)-2-Pivaloylthiophene *O*-(2-(4-methoxyphenyl)acetyl) oxime (**16**) (^{1}H NMR; 400 MHz; $CDCl_3$)

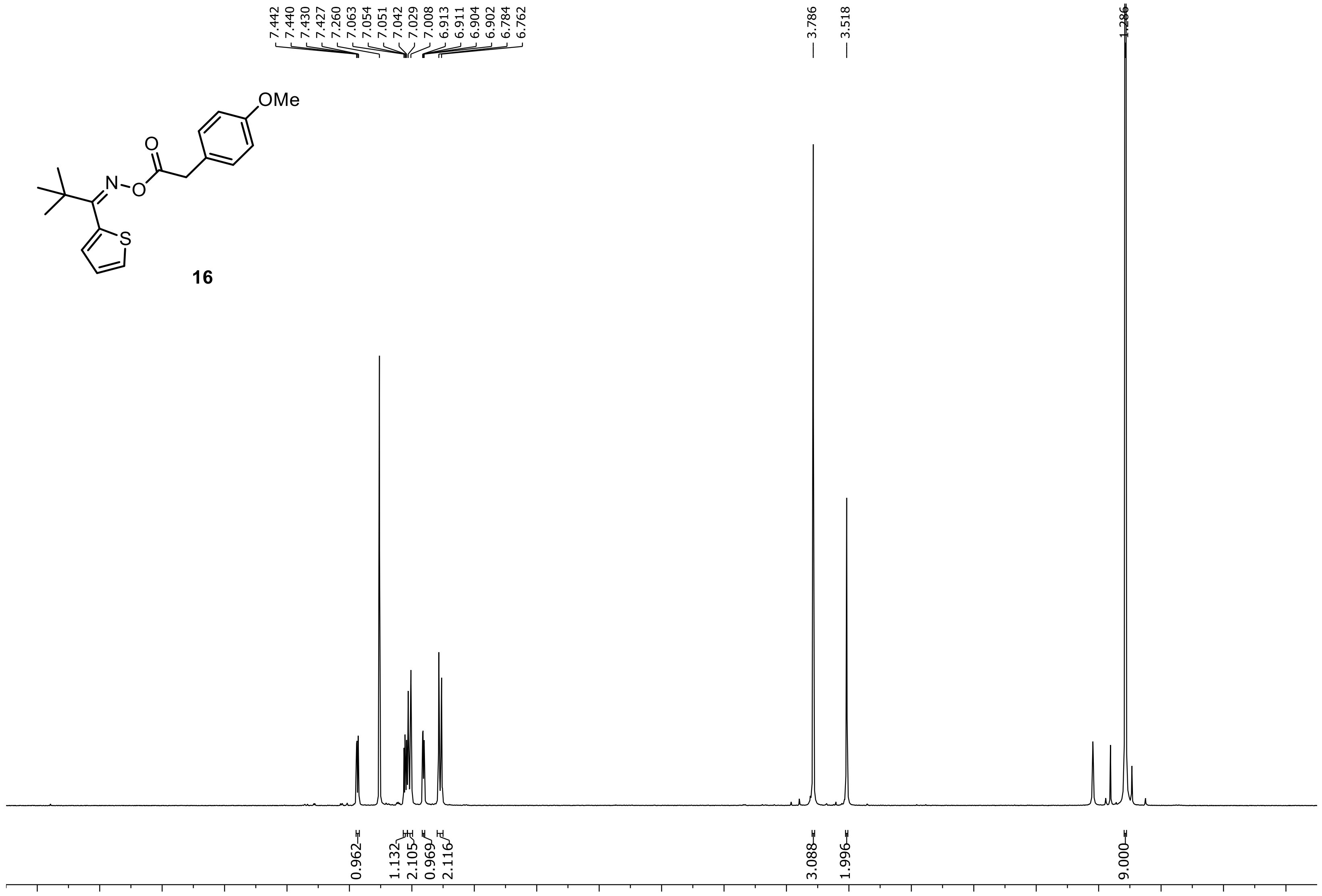

(*Z*)-2-Pivaloylthiophene *O*-(2-(4-methoxyphenyl)acetyl) oxime (**16**) (^{13}C NMR; 101 MHz; $CDCl_3$)

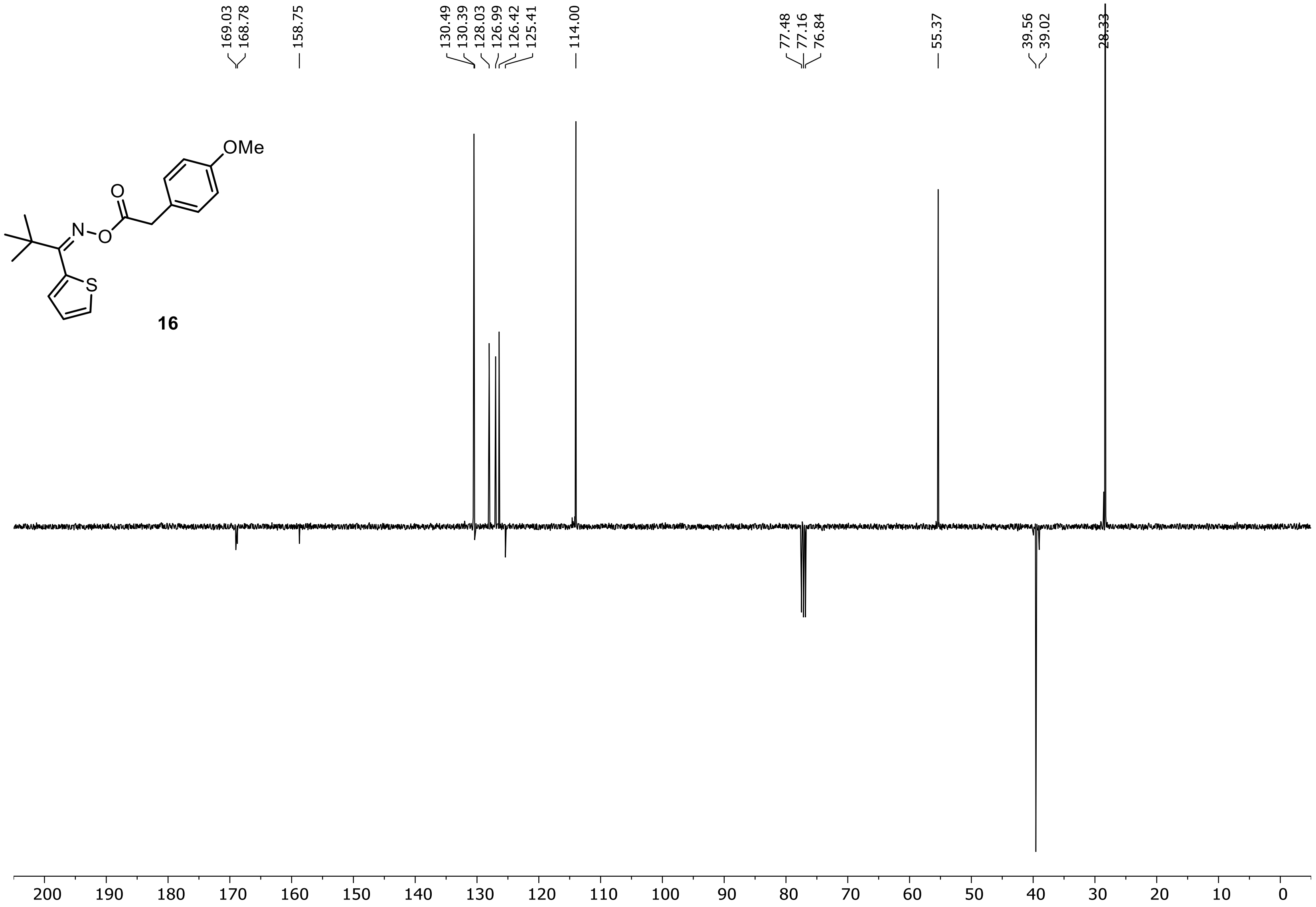

(*Z*)-2-Pivaloylthiophene *O*-sulfamoyl oxime (**17**) (^{1}H NMR; 400 MHz; $CDCl_3$)

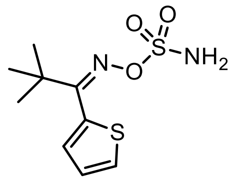

17

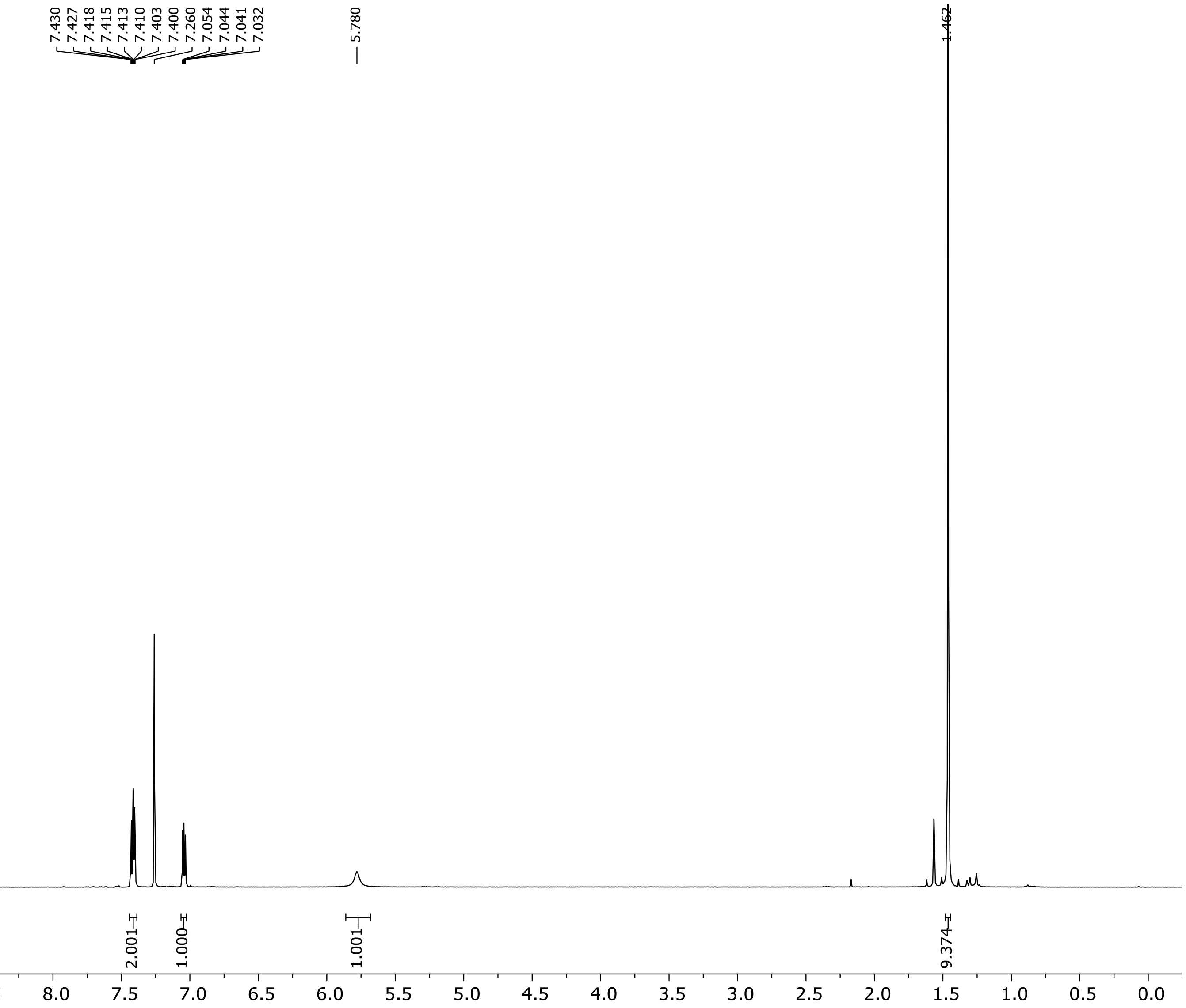

(*Z*)-2-Pivaloylthiophene *O*-sulfamoyl oxime (**17**) (^{13}C NMR; 101 MHz; $CDCl_3$)

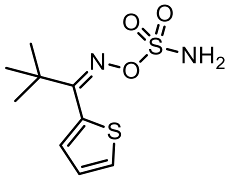

17

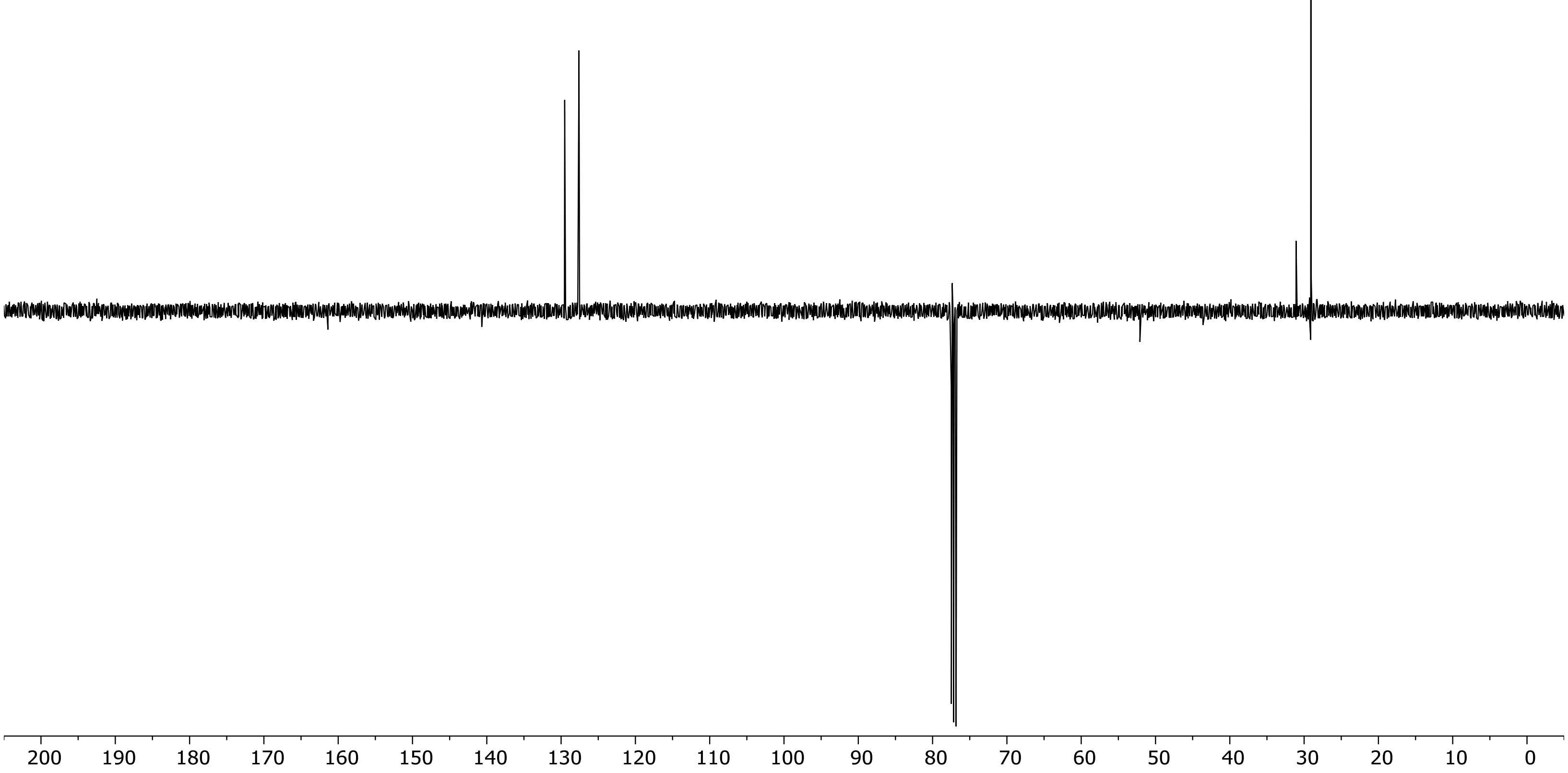

2-Acetylthiophene oxime (**S2a**) (^{1}H NMR; 400 MHz; $CDCl_3$)

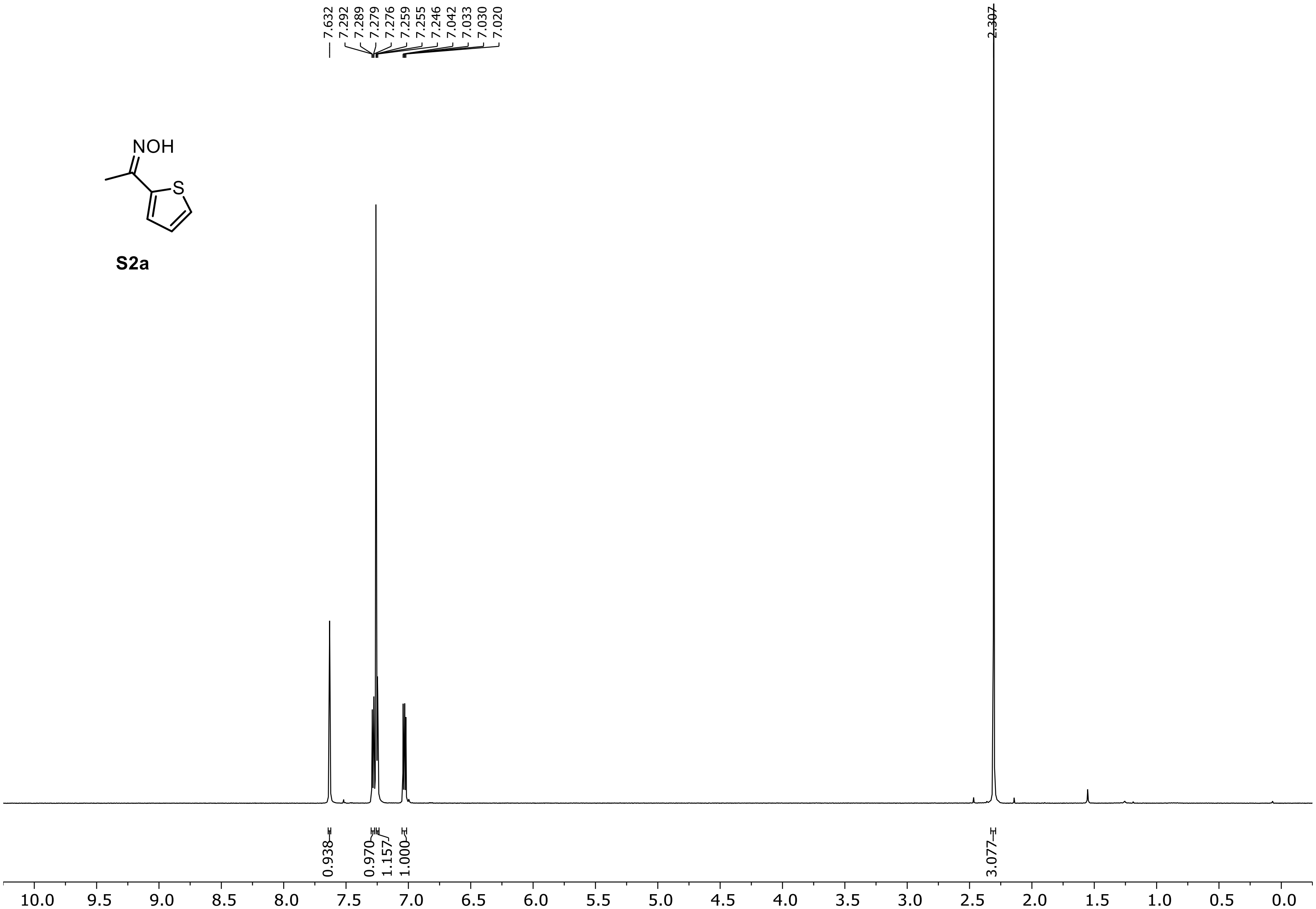

2-Acetylthiophene oxime (**S2a**) (^{13}C NMR; 100 MHz; $CDCl_3$)

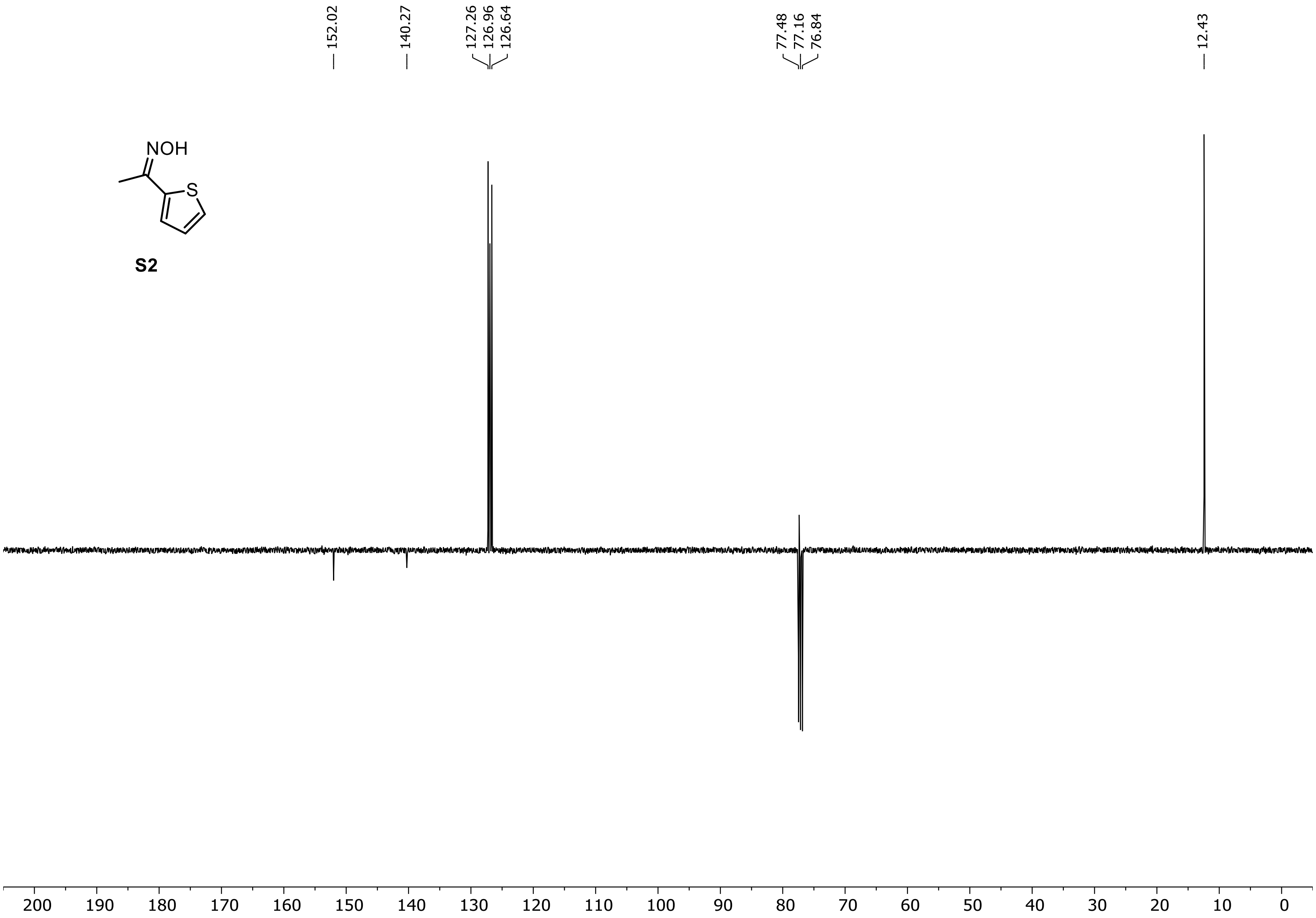

2-Acetylthiophene oxime isomers (**S2a**/**S2b**) (^{1}H NMR; 400 MHz; $CDCl_3$)

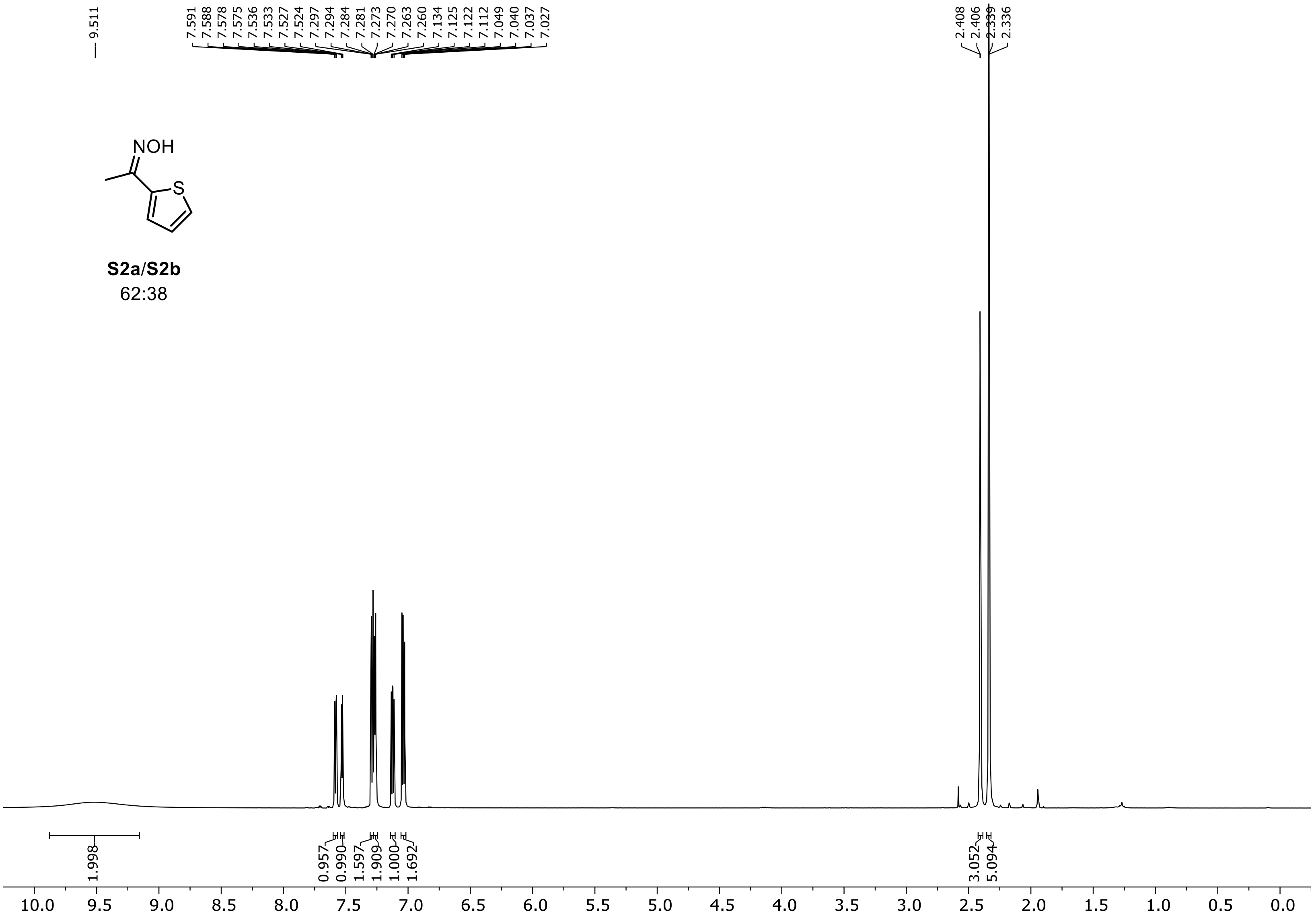

2-Acetylthiophene oxime isomers (**S2a**/**S2b**) (^{13}C NMR; 176 MHz; $CDCl_3$)

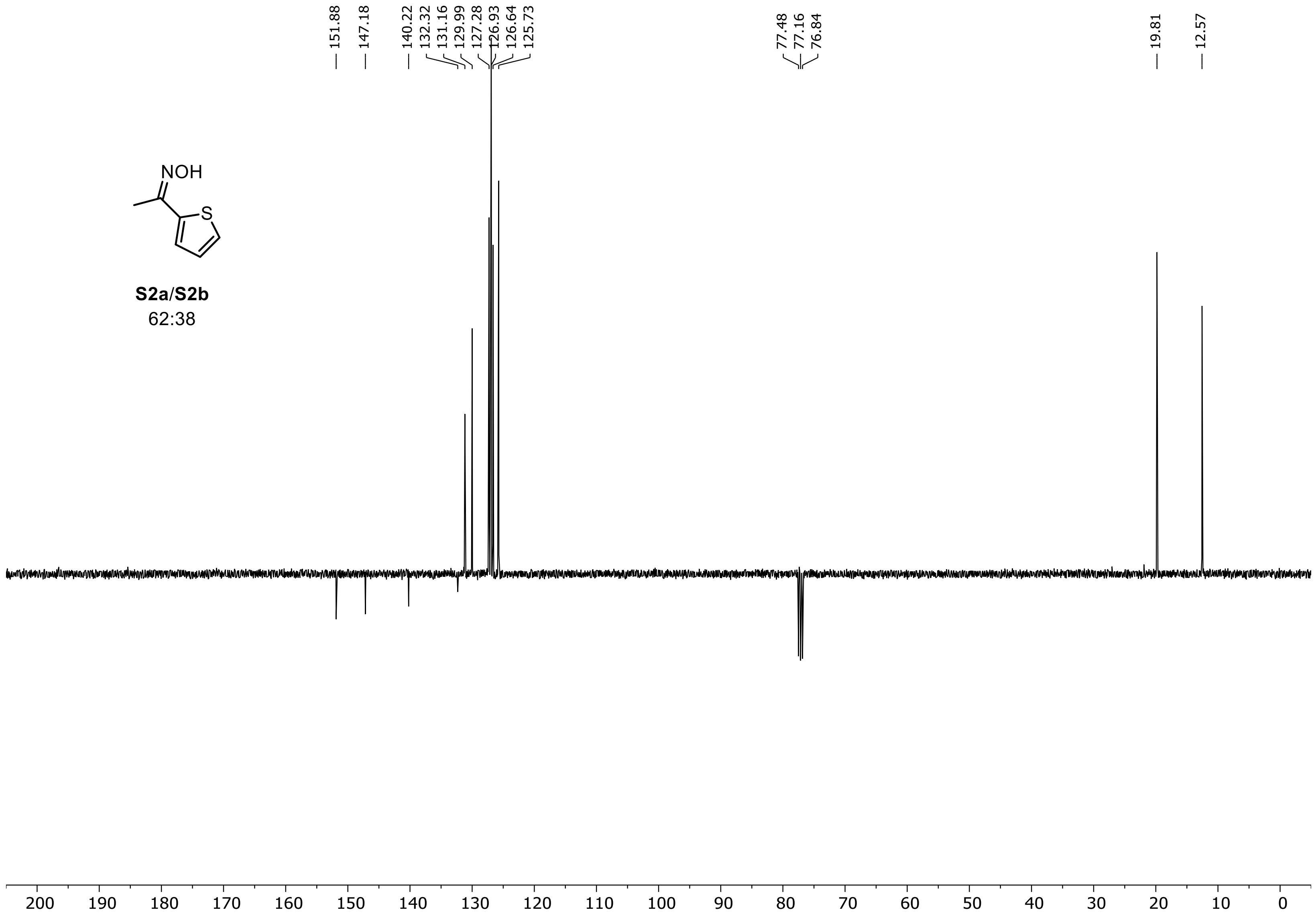

2-Acetylthiophene oxime isomers (**S2a**/**S2b**) (HSQC; $CDCl_3$)

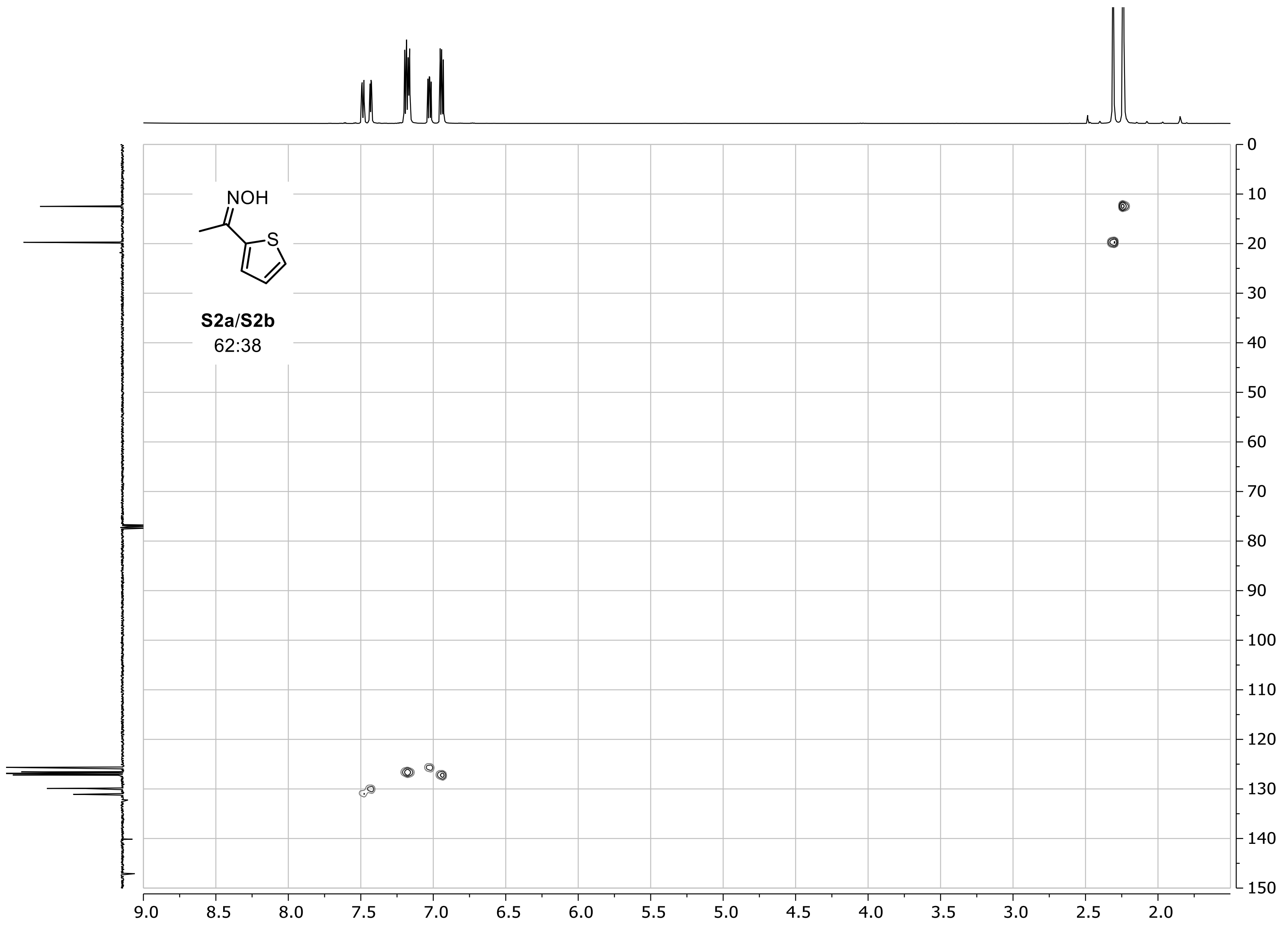

(Z)-2-Acetylthiophene O-(4-methoxybenzoyl) oxime (Z)-**18** (^{1}H NMR; 700 MHz; $CDCl_3$)

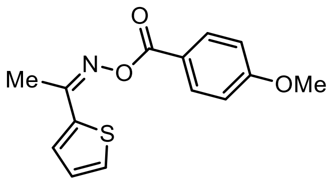

(Z)-**18**

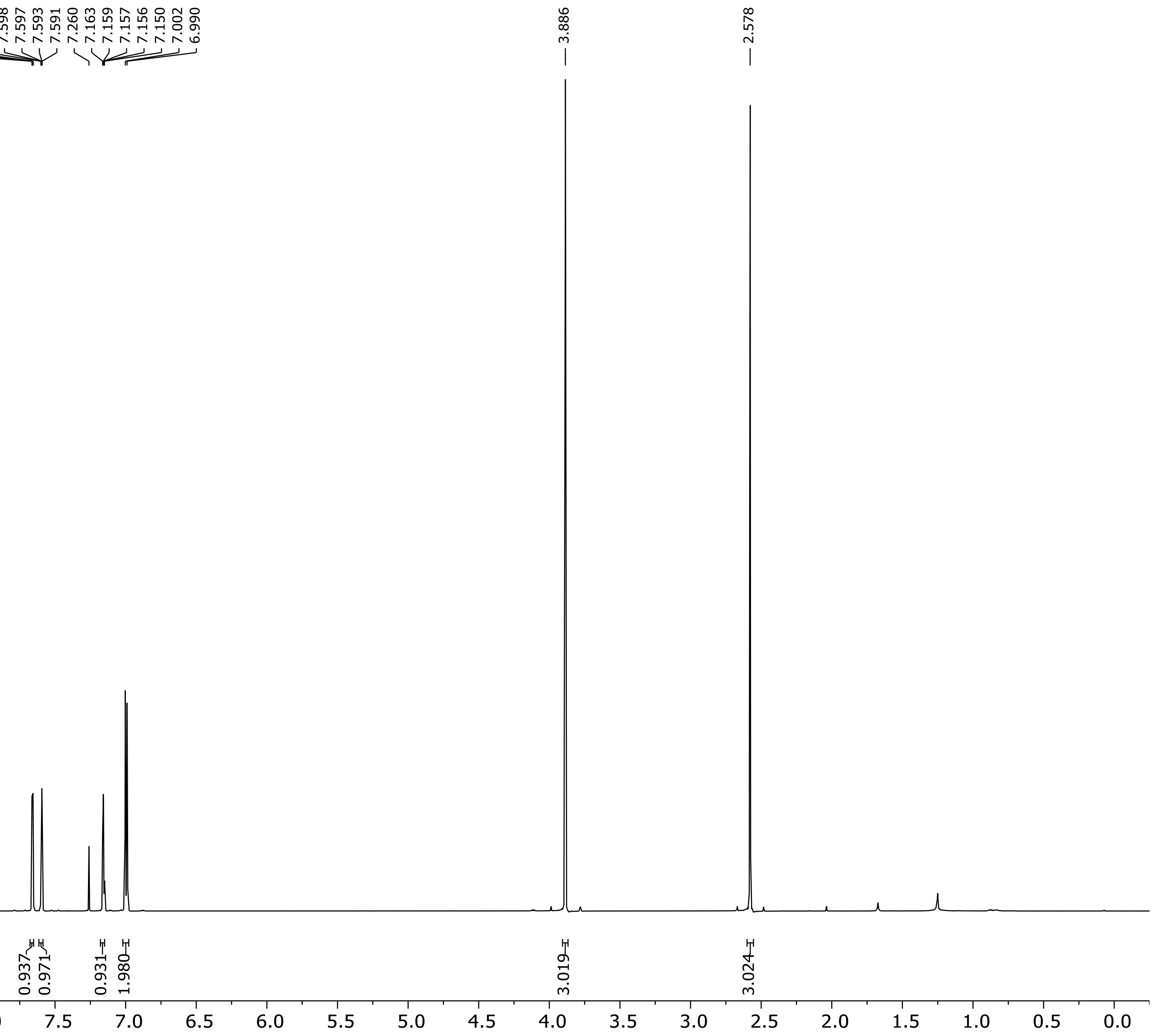

(*Z*)-2-Acetylthiophene *O*-(4-methoxybenzoyl) oxime (*Z*)-**18** (^{13}C NMR; 176 MHz; $CDCl_3$)

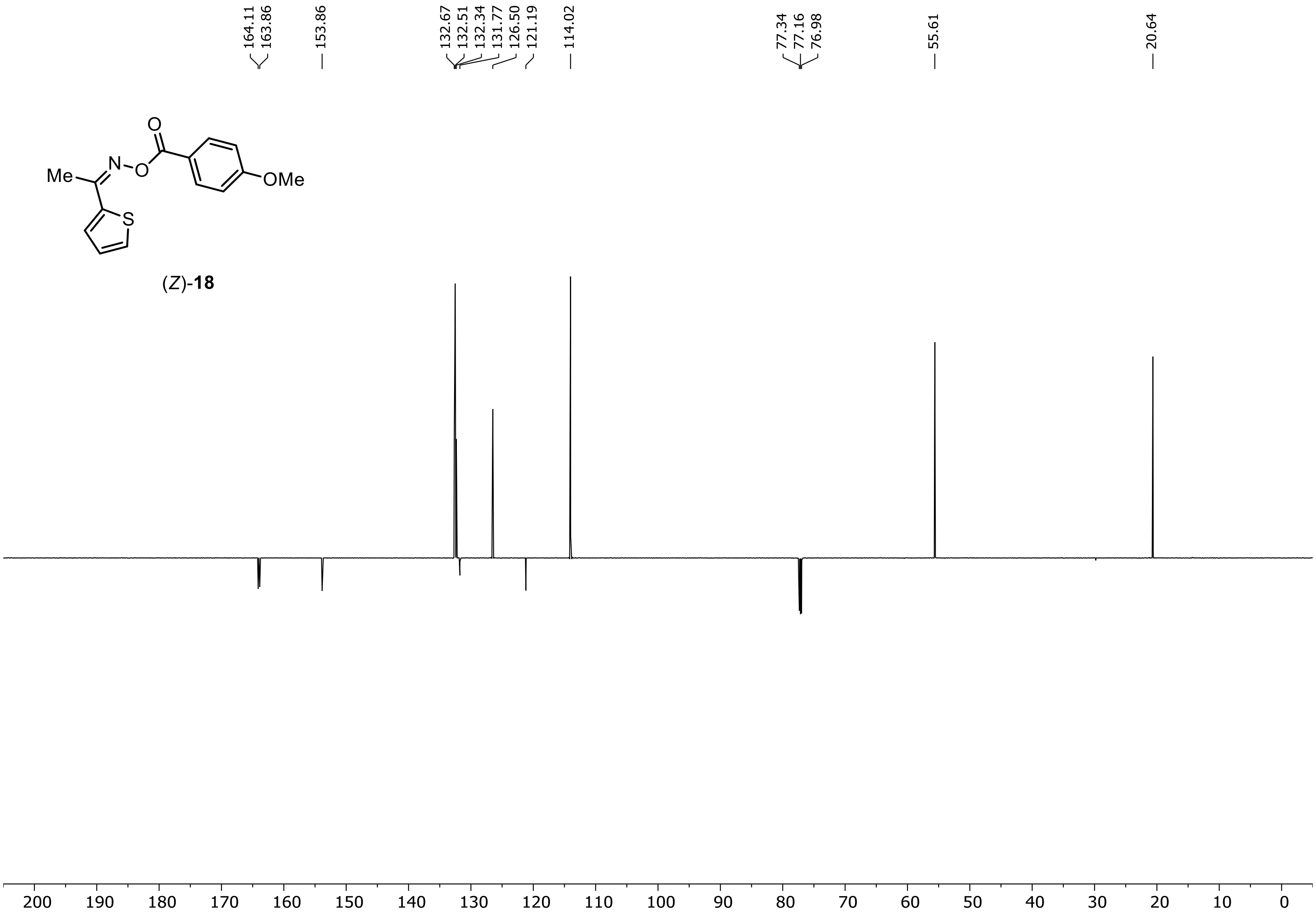

2-(Propan-1-oyl)thiophene oxime (**S3b**) (^{1}H NMR; 400 MHz; CD_2Cl_2)

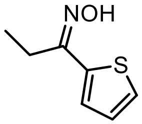

S3b

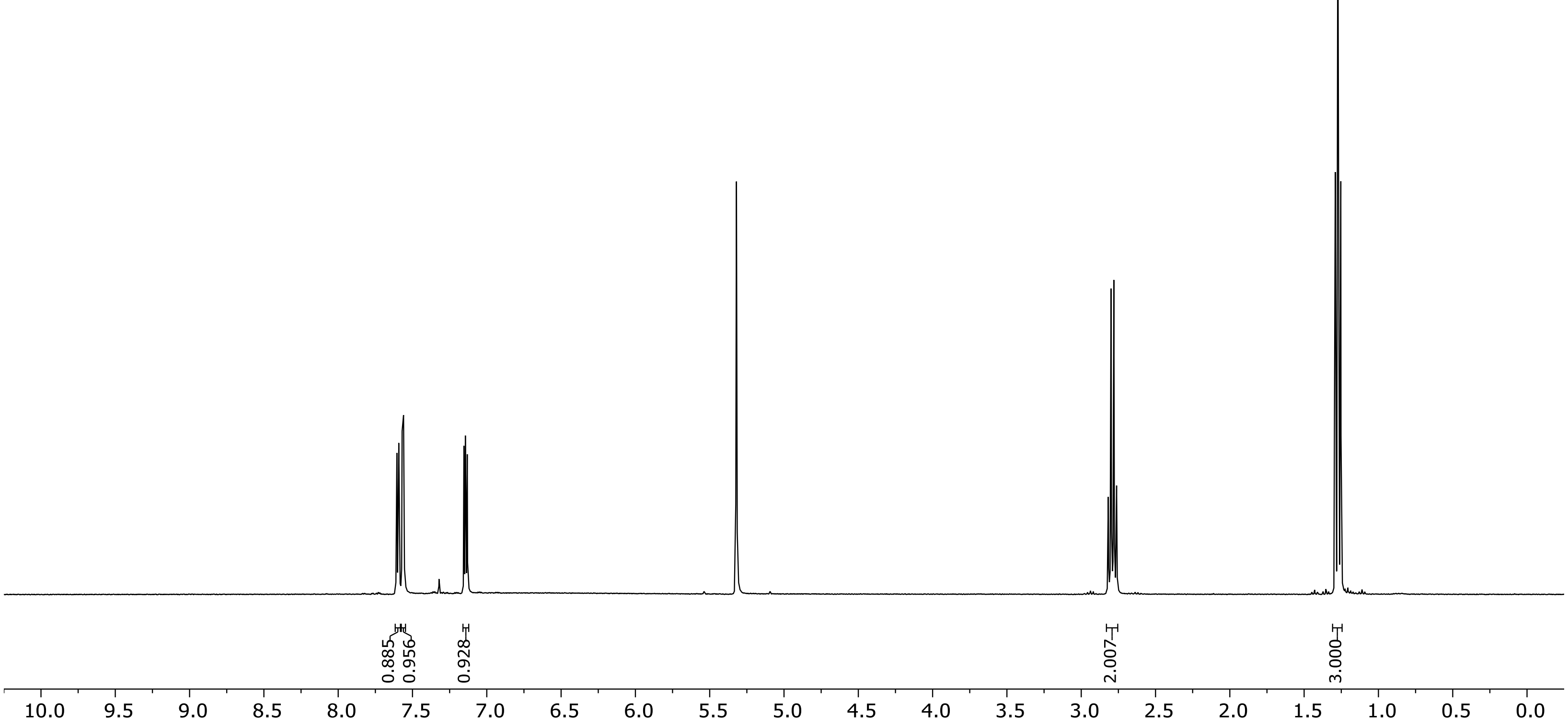

2-(Propan-1-oyl)thiophene oxime (**S3b**) (^{13}C NMR; 101 MHz; $CDCl_3$)

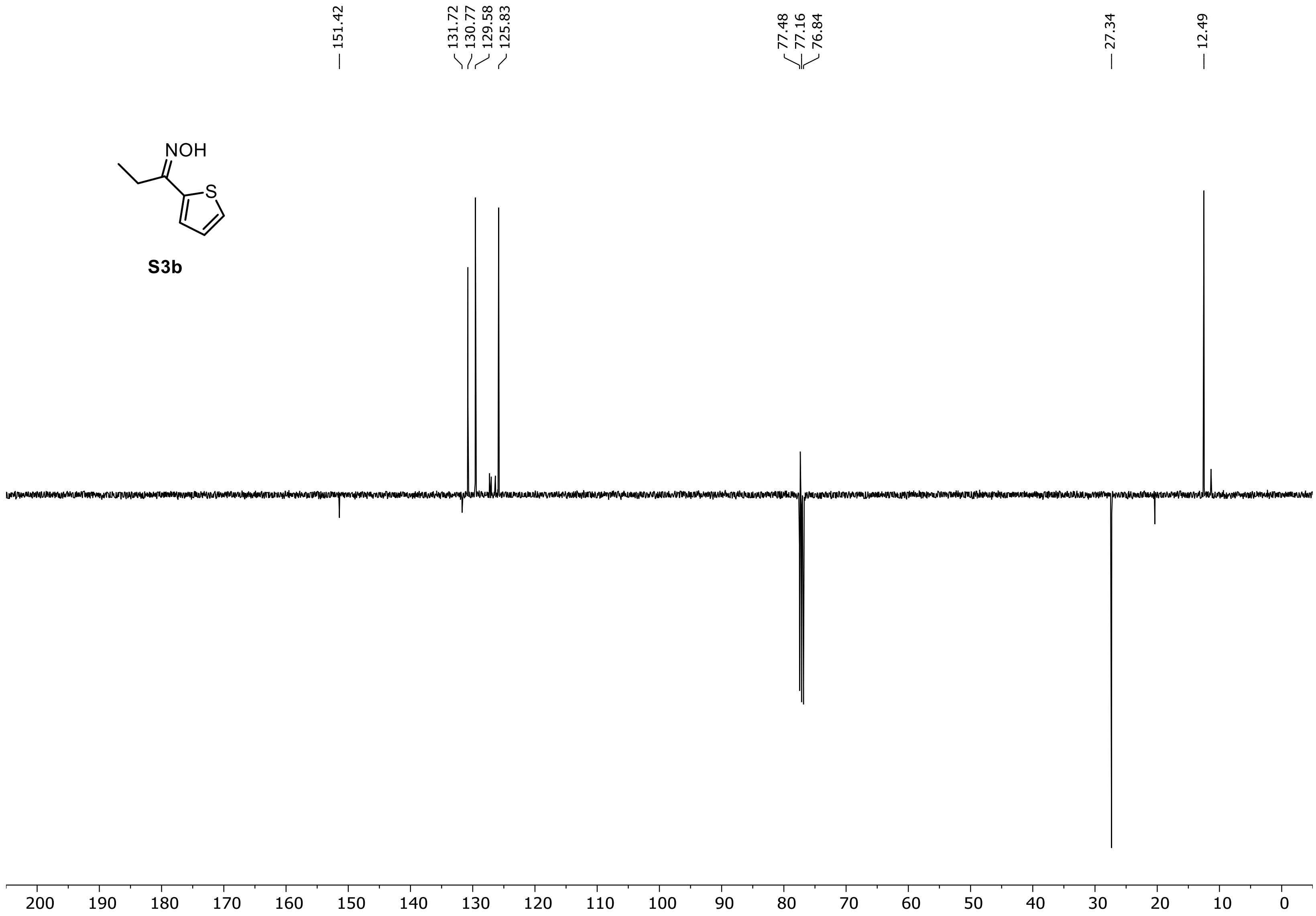

2-(Propan-1-oyl)thiophene *O*-(4-methoxybenzoyl) oxime (**19**) (^{1}H NMR; 700 MHz; $CDCl_3$)

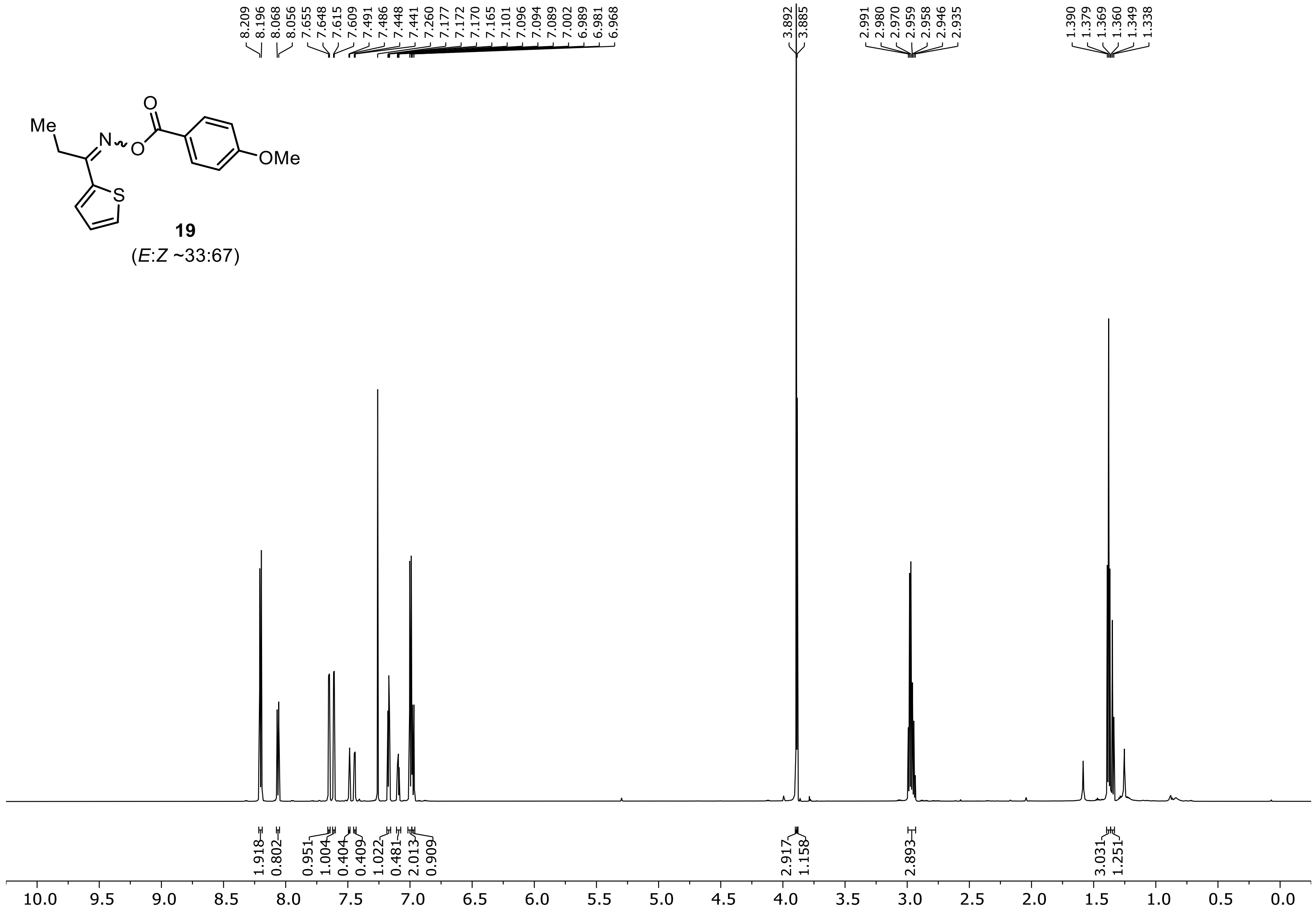

19
(*E*:*Z* ~33:67)

2-(Propan-1-oyl)thiophene *O*-(4-methoxybenzoyl) oxime (**19**) (^{13}C NMR; 176 MHz; CDCl$_3$)

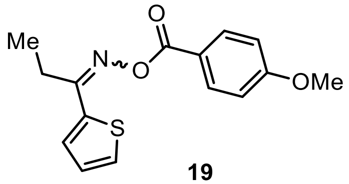

19
(*E*:*Z* ~33:67)

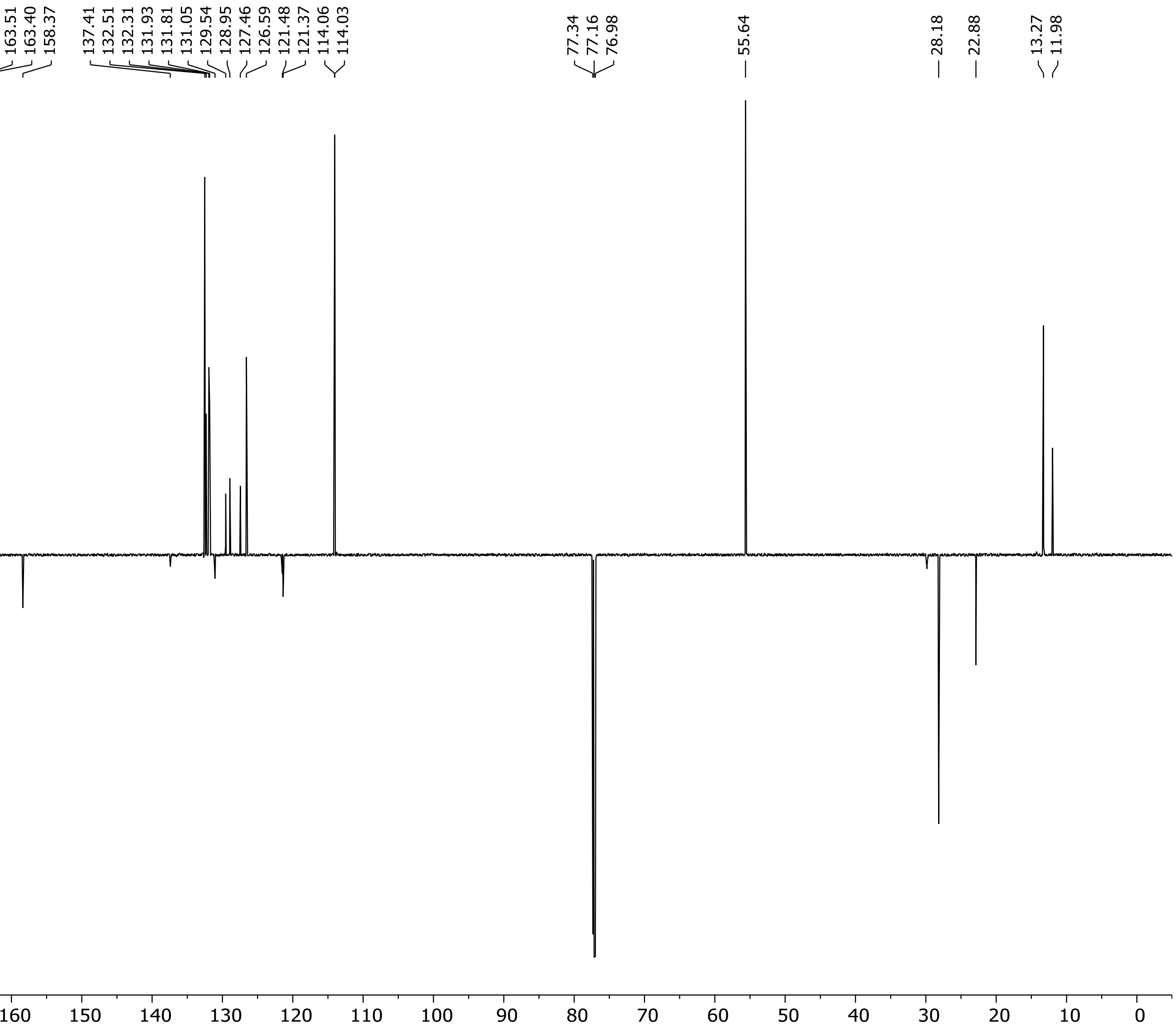

2,2-Dimethyl-1-(thiazol-5-yl)propan-1-ol (**S4**) (^{1}H NMR; 400 MHz; $CDCl_3$)

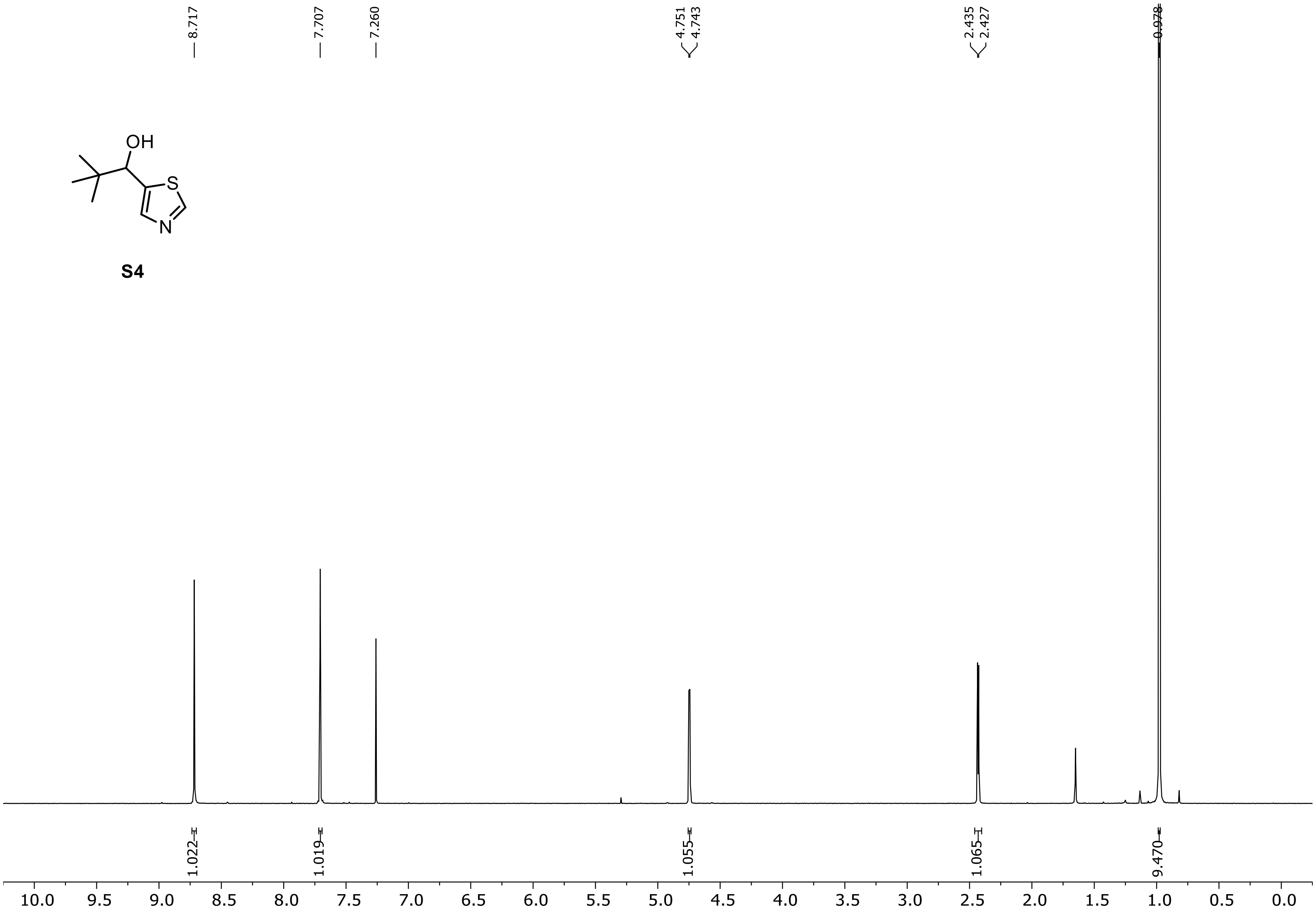

2,2-Dimethyl-1-(thiazol-5-yl)propan-1-ol (**S4**) (^{13}C NMR; 101 MHz; $CDCl_3$)

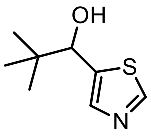

S4

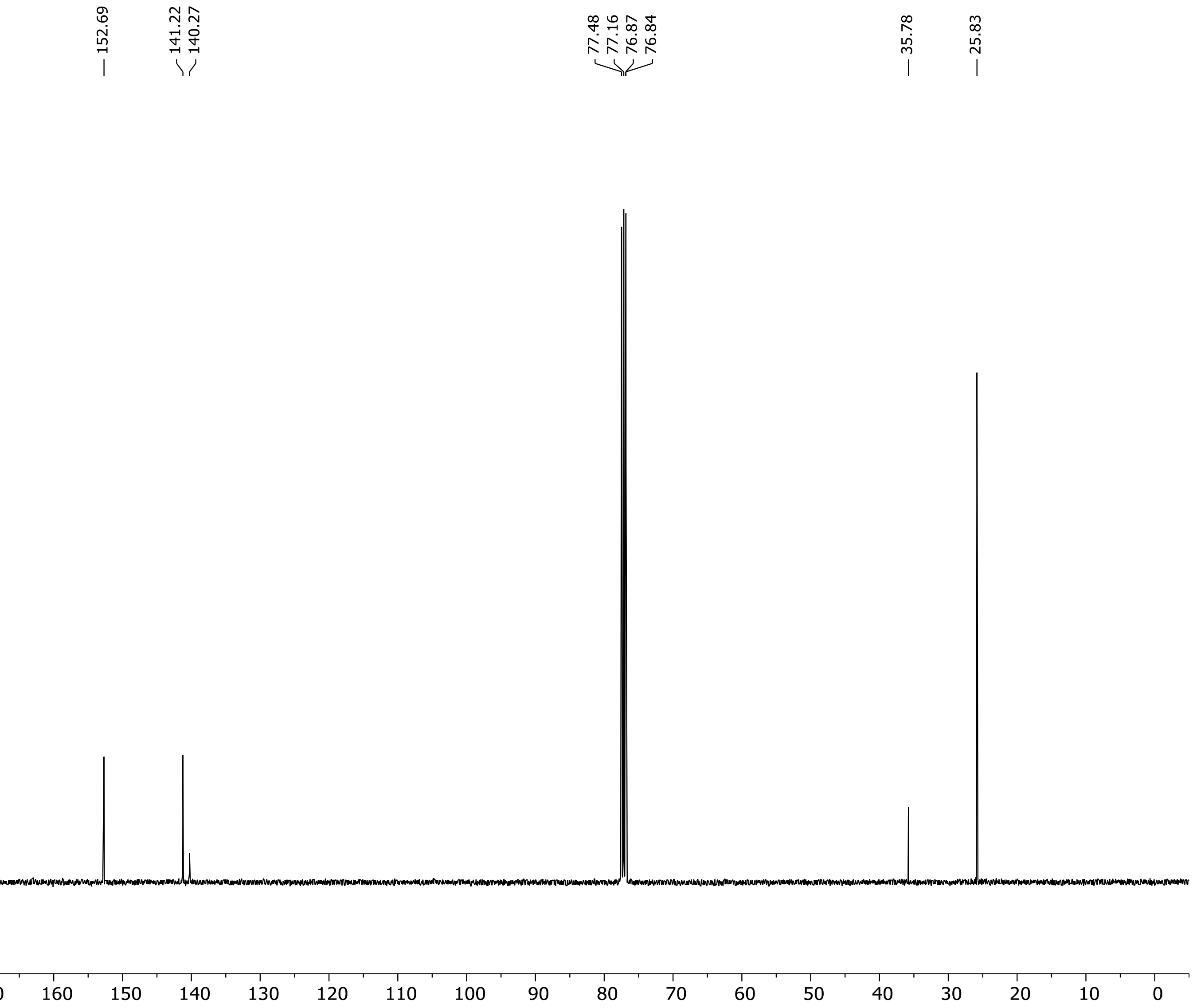

5-Pivaloylthiazole (**S5**) (^{1}H NMR; 400 MHz; $CDCl_3$)

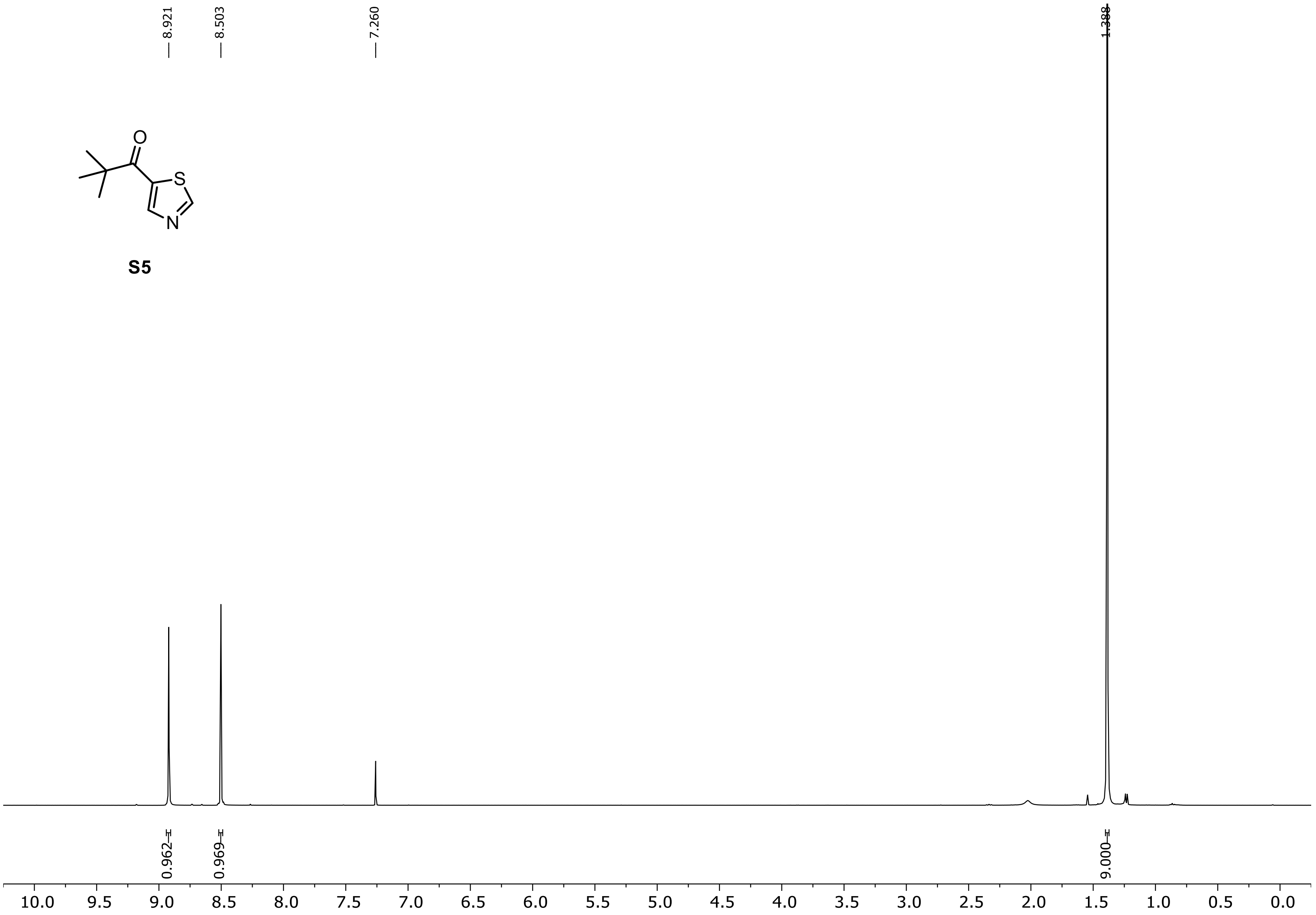

5-Pivaloylthiazole (**S5**) (^{13}C NMR; 101 MHz; $CDCl_3$)

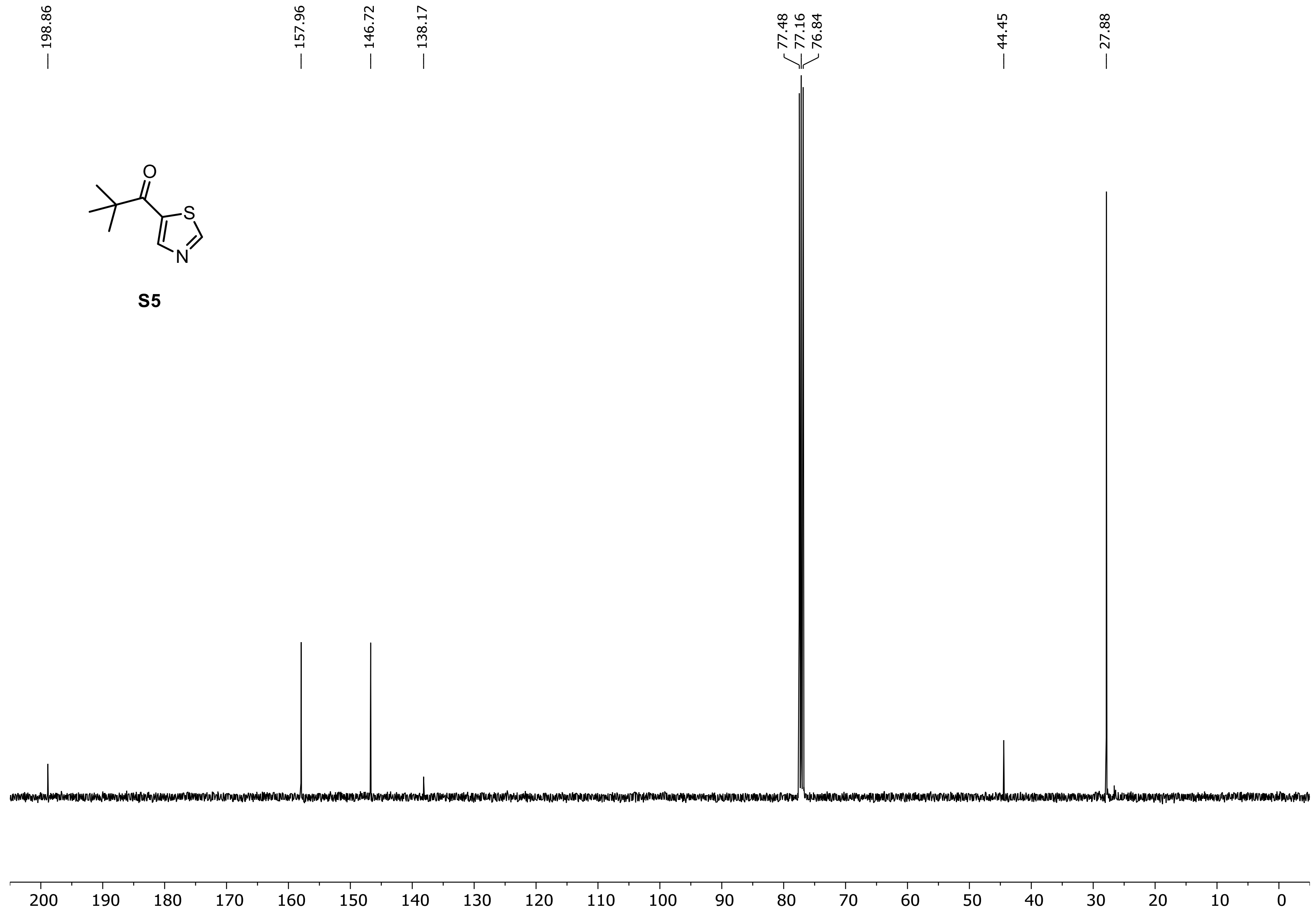

(*Z*)-5-Pivaloylthiazole *O*-(4-methoxybenzoyl) oxime (**20**) (^{1}H NMR; 400 MHz; $CDCl_3$)

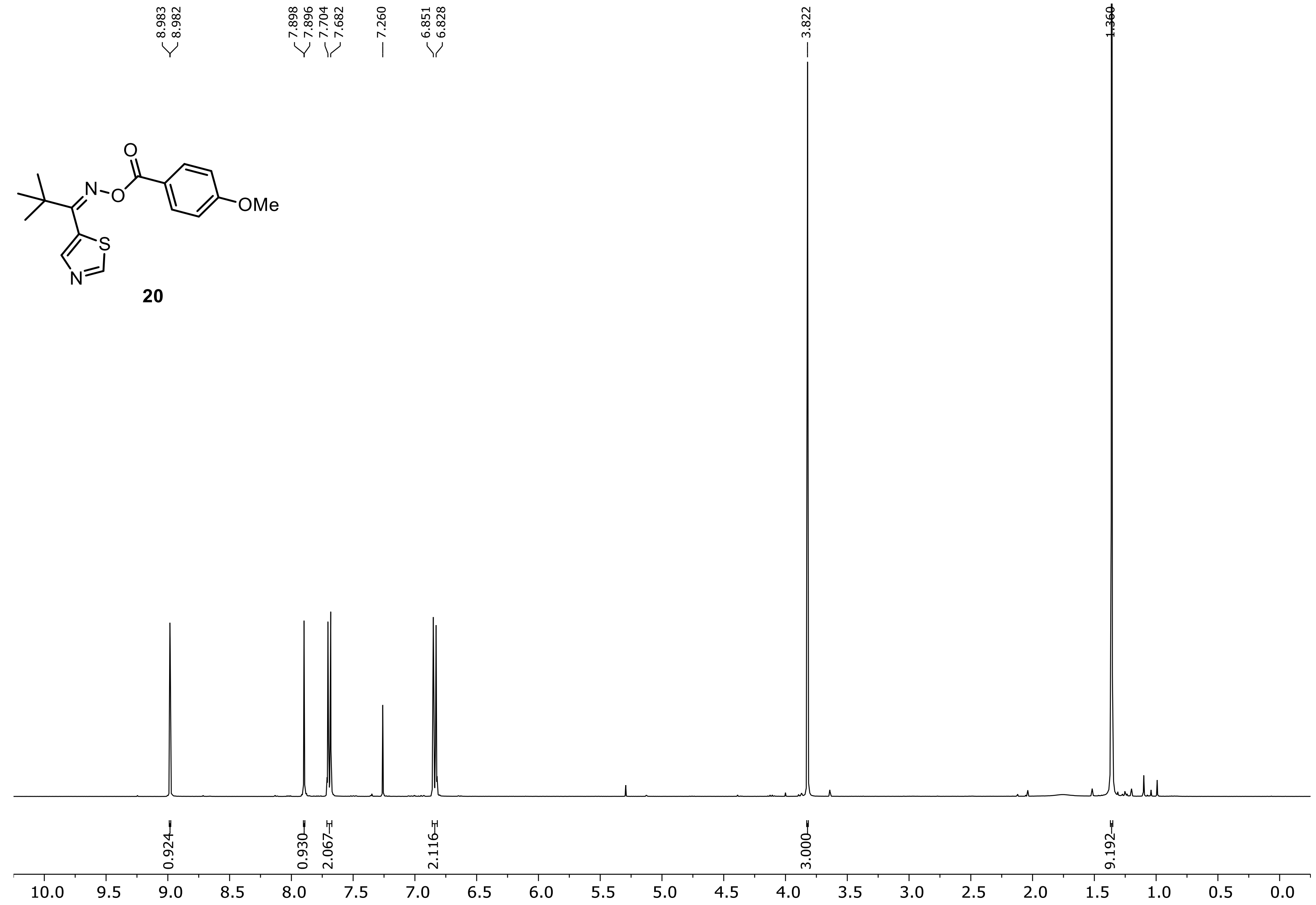

(*Z*)-5-Pivaloylthiazole *O*-(4-methoxybenzoyl) oxime (**20**) (^{13}C NMR; 101 MHz; $CDCl_3$)

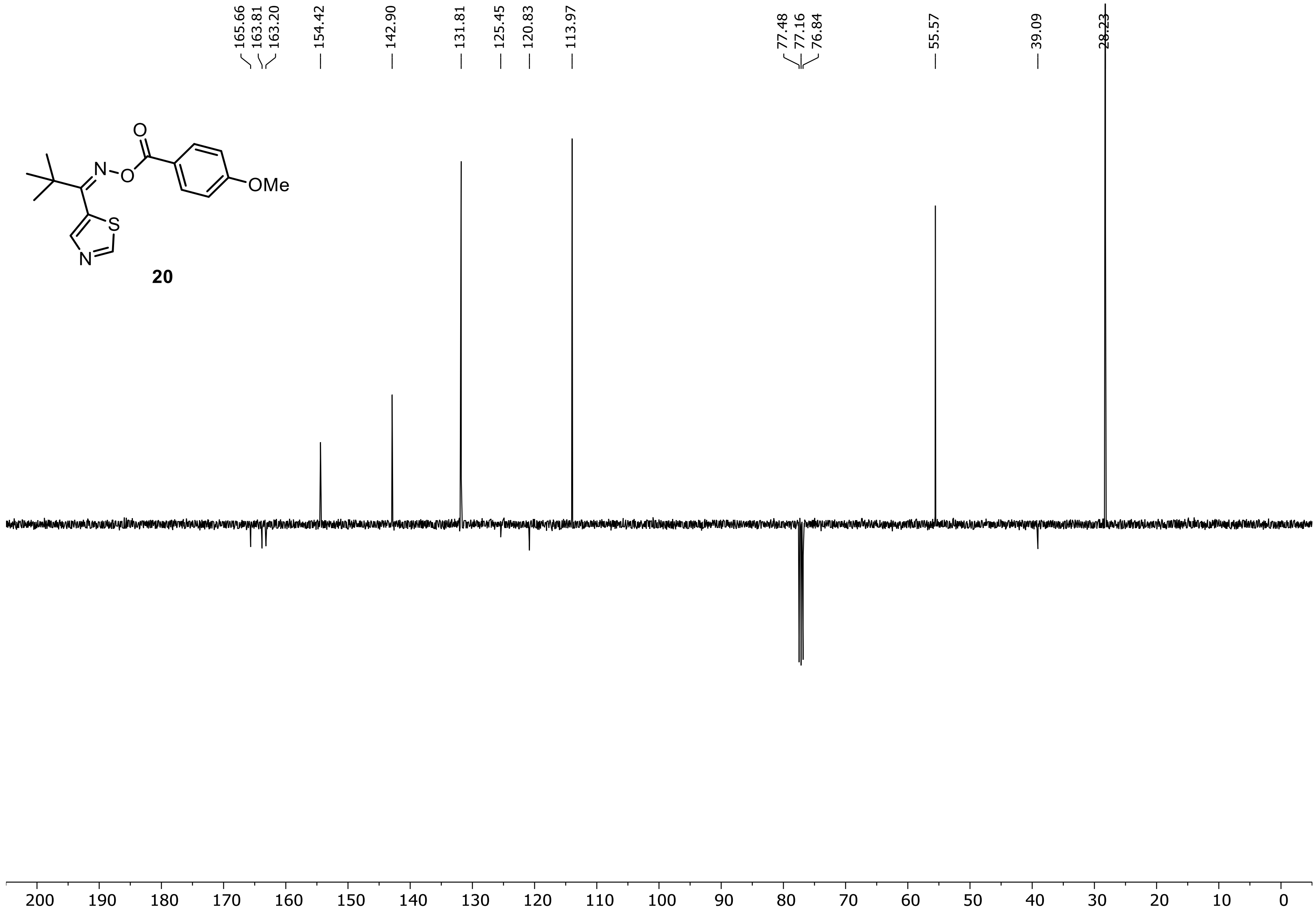

2,2,2-Trimethylacetophenone *O*-(4-methoxybenzoyl) oxime (**21**) (^1H NMR; 400 MHz; $CDCl_3$)

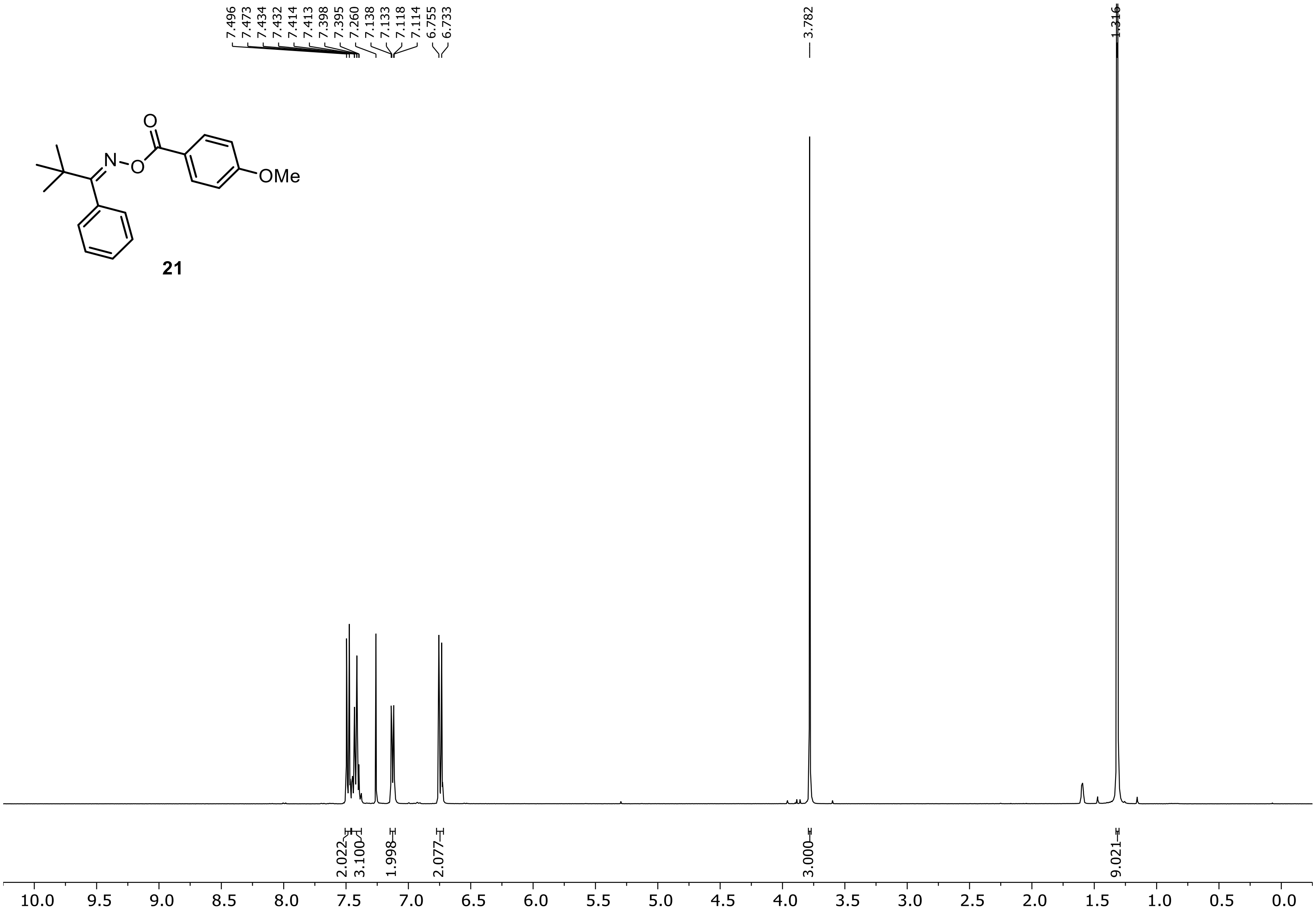

2,2,2-Trimethylacetophenone *O*-(4-methoxybenzoyl) oxime (**21**) (^{13}C NMR; 101 MHz; $CDCl_3$)

21

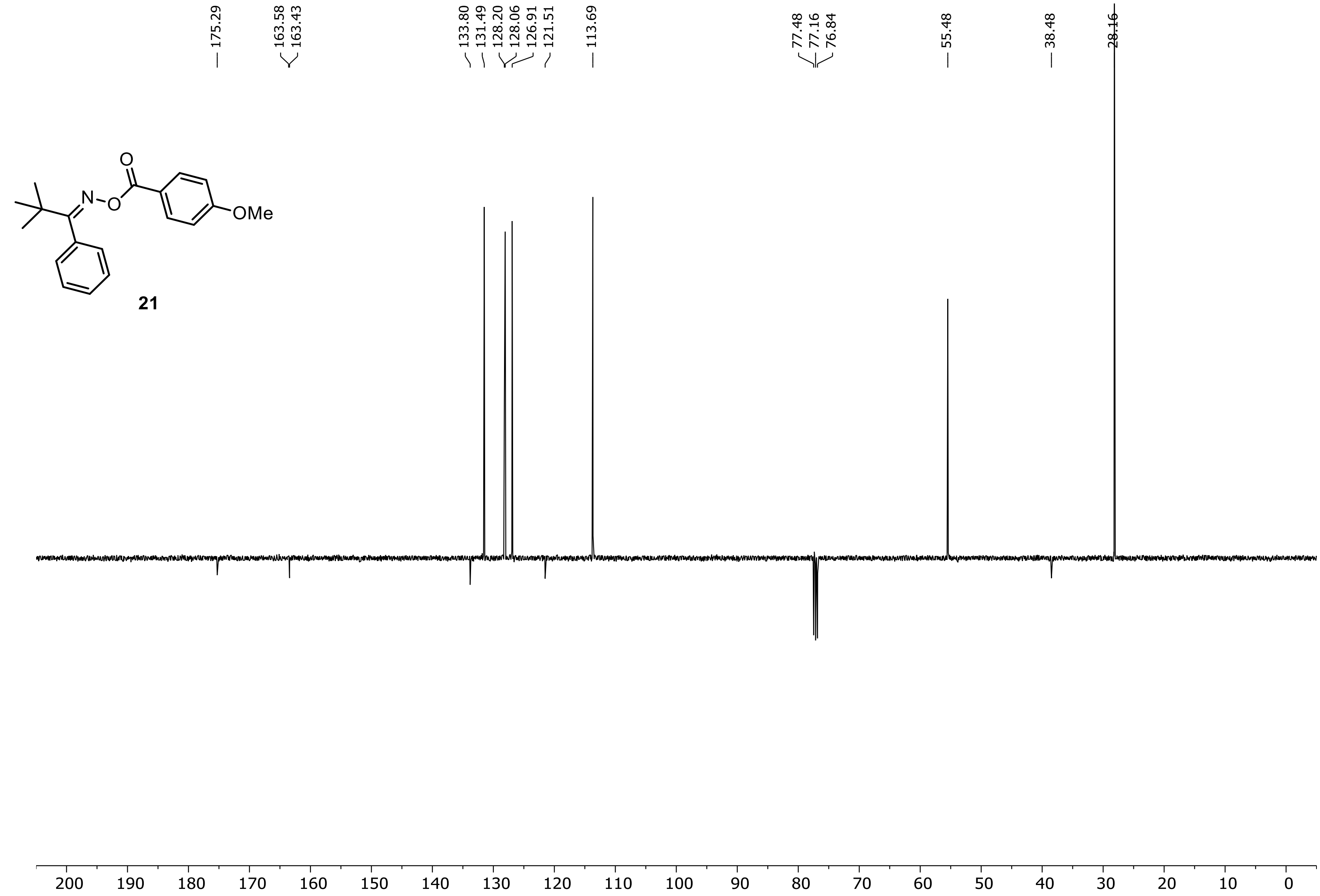

2,2-Dimethyl-1-(pyridin-3-yl)propan-1-ol (**S8**) (^{1}H NMR; 400 MHz; $CDCl_3$)

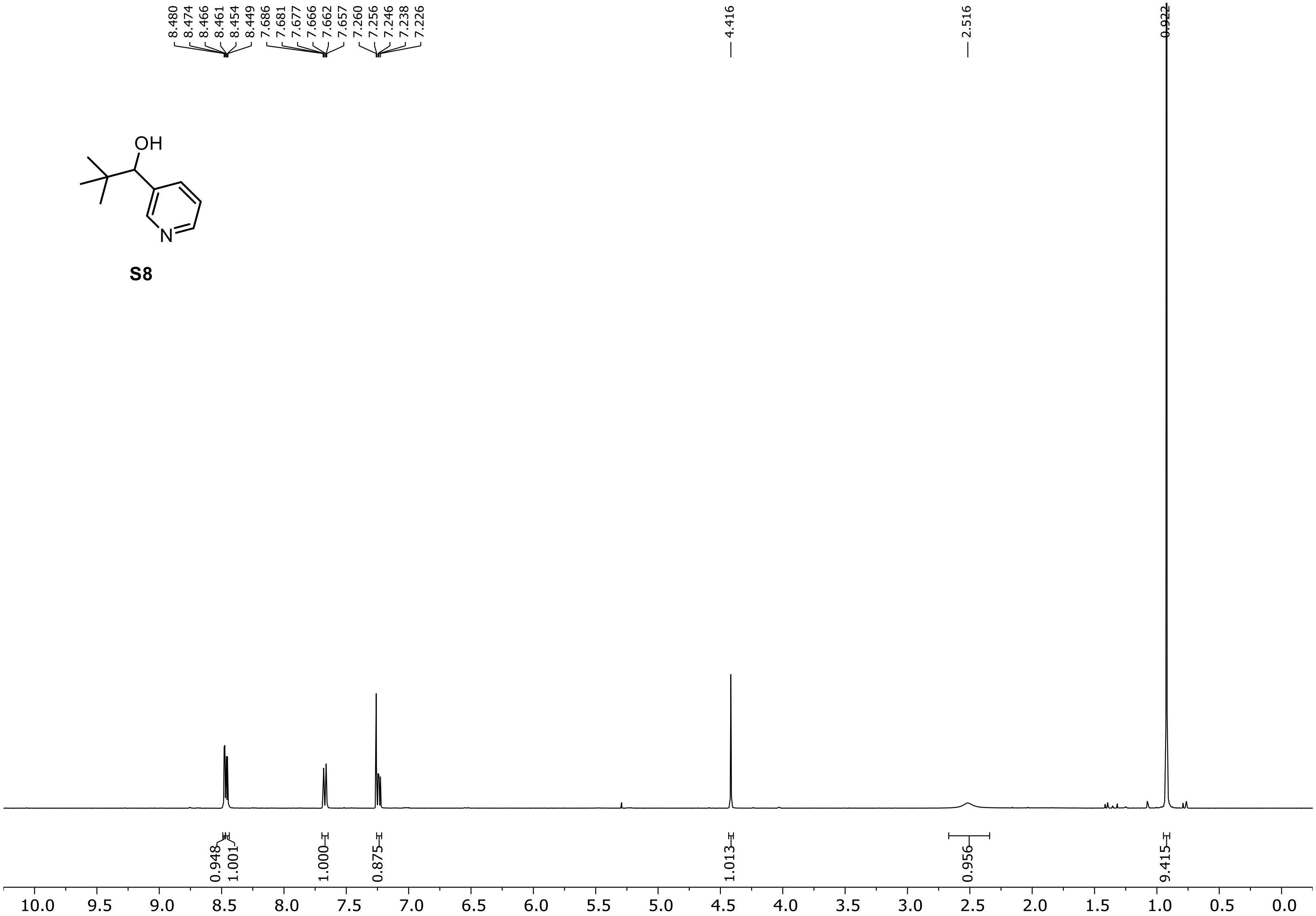

2,2-Dimethyl-1-(pyridin-3-yl)propan-1-ol (**S8**) (^{13}C NMR; 101 MHz; $CDCl_3$)

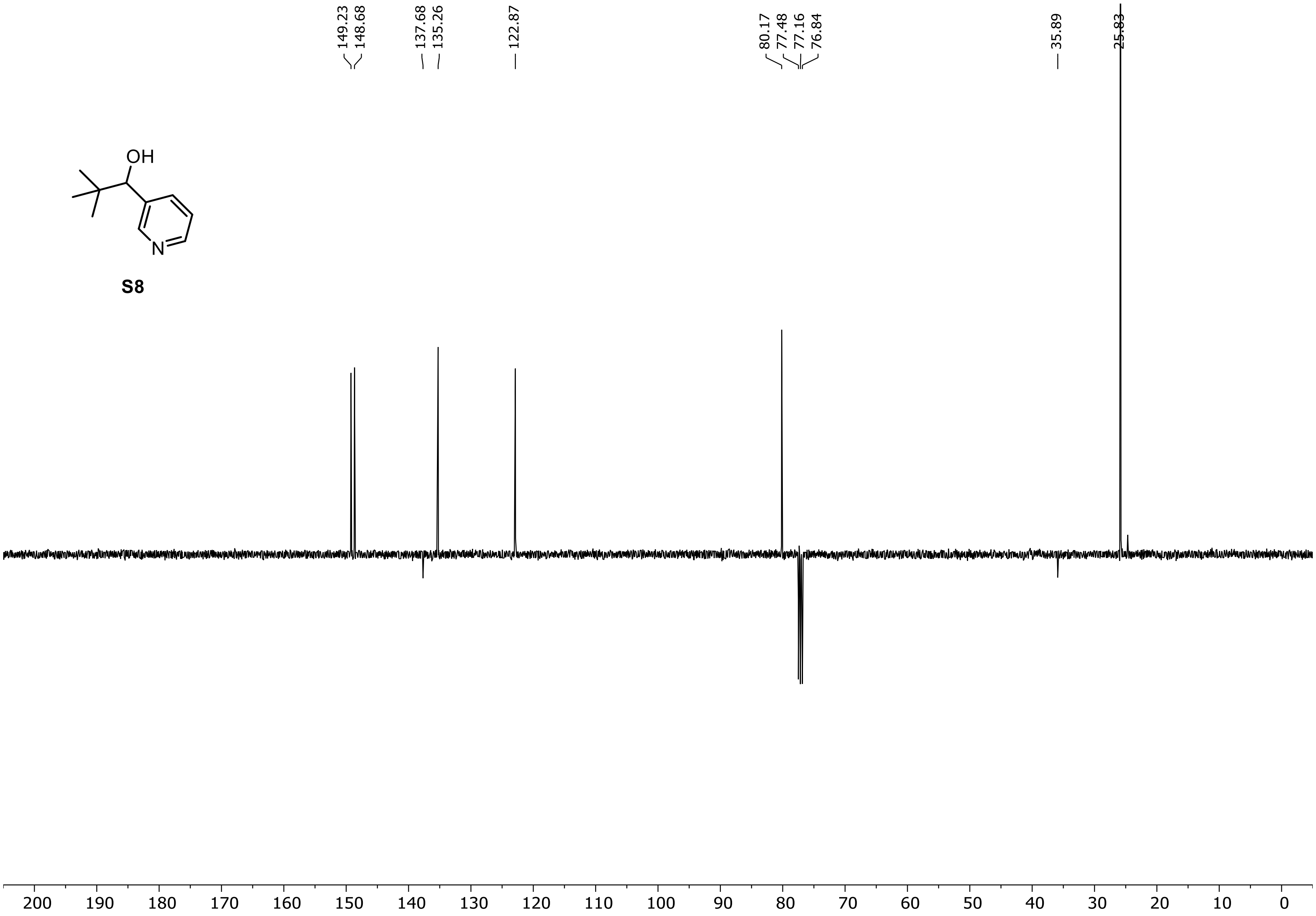

(*Z*)-3-Pivaloylpyridine *O*-(4-methoxybenzoyl) oxime (**22**) (^{1}H NMR; 400 MHz; $CDCl_3$)

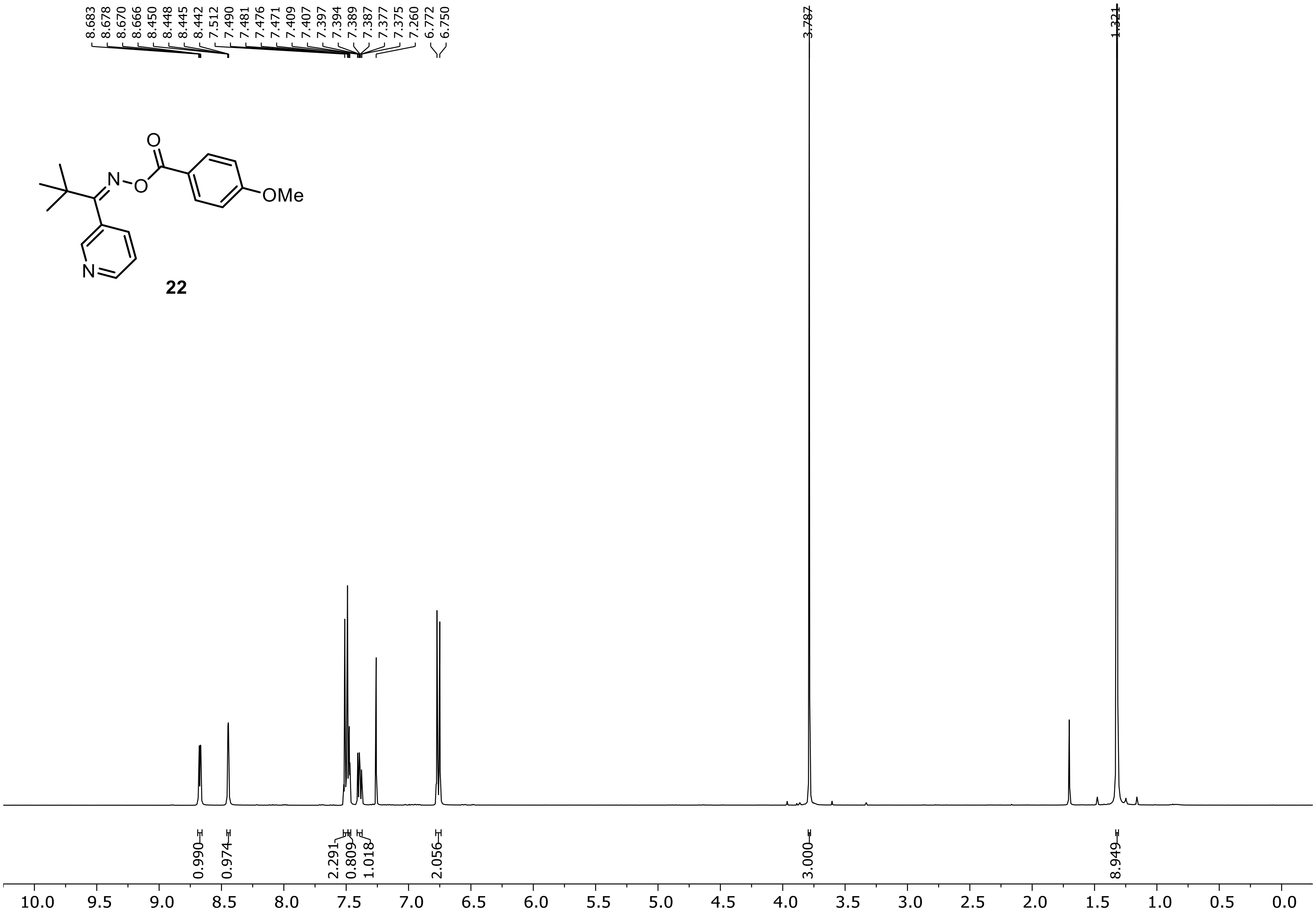

(*Z*)-3-Pivaloylpyridine *O*-(4-methoxybenzoyl) oxime (**22**) (^{13}C NMR; 101 MHz; $CDCl_3$)

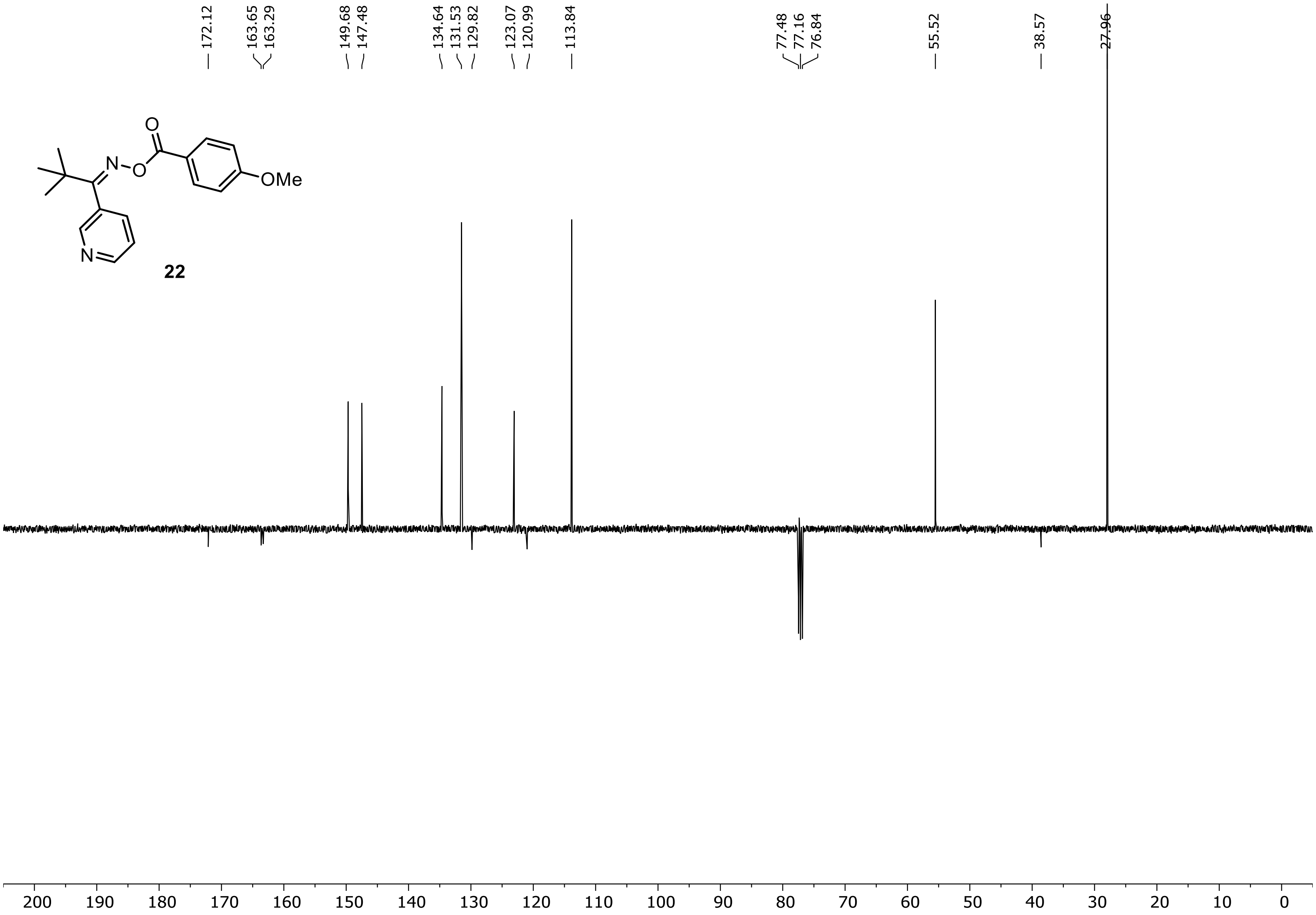

22
