# Supplementary material for: A screen for cell envelope stress uncovers an inhibitor of prolipoprotein diacylglyceryl transferase, Lgt, in Escherichiacoli
Source: iScience. 2024 Sep 5;27(10):110894. doi: 10.1016/j.isci.2024.110894 (PMC11456916; doi:10.1016/j.isci.2024.110894)
Supplement: Data S6. MAC-0452936 and Z-(18) X-ray crystallography [file mmc7.pdf]

X-Ray Crystallographic Data

MAC-0452936

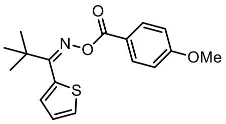

1: MAC-0452936

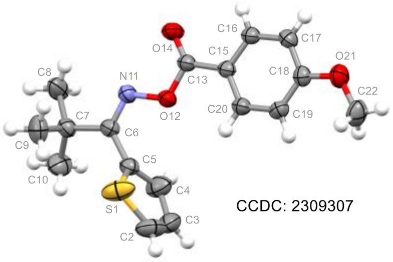

CCDC: 2309307

X-ray structure of MAC-0452936 (**1**).

Crystal Data and Structure Refinement for MAC-0452936.

Identification code	RI-01-113
Empirical formula	$C_{17}H_{19}NO_3S$
Formula weight	317.39
Temperature	296.00 K
Crystal system	monoclinic
Space group	$P2_1/C$
Unit cell dimensions	a = 10.2246(2) Å, b = 110.6532(12) Å, c = 15.9482(14) Å
	α = 90°, β = 100.551(12)°, γ = 90°
Volume	1707.8(3) $Å^3$
Z	4
ρ_{calc} (calculated density)	1.234 g/cm^3
absorption coefficient	1.779 μ/mm^{-1}
F(000)	672.0
Crystal size	0.20 mm × 0.20 mm × 0.03 mm
Radiation	CuKα (λ = 1.54178)
2Θ range for data collection	8.796 to 149.012°
Index ranges	$-12 \leq h \leq 12$, $-13 \leq k \leq 13$, $-19 \leq l \leq 19$
Reflections collected	31020
Independent reflections	3482 [R_{int} = 0.0591, R_{sigma} = 0.0379]
Data/restraints/parameters	3482/16/257
Goodness-of-fit on F^2	1.060
Final R indexes [I≥2σ (I)]	R_1 = 0.0471, wR_2 = 0.1243
Final R indexes [all data]	R_1 = 0.0693, wR_2 = 0.1327
Largest diff. peak/hole / e $Å^{-3}$	0.20/–0.26

Fractional atomic coordinates (× 10^4) and equivalent isotropic displacement parameters (Å^2 × 10^3) for MAC-0452936 (1). U_{eq} is defined as 1/3 of the trace of the orthogonalized U_{IJ} tensor.

Atom	*x*	*y*	*z*	U(eq)
C5	3598.9(15)	1535.7(14)	6061.1(10)	61.9(4)
C6	3198.4(14)	1406.6(13)	6903.7(10)	57.9(3)
C7	3618.5(17)	254.6(15)	7457.7(11)	66.6(4)
N11	2493.2(14)	2211.9(12)	7209.7(9)	66.9(3)
O12	2203.2(12)	3254.1(10)	6635.4(7)	66.4(3)
C13	1495.5(15)	4167.0(14)	6944.8(10)	61.1(4)
O14	1124.4(18)	4095.5(13)	7607.2(9)	99.4(5)
C15	1271.0(14)	5240.4(13)	6349.3(9)	55.1(3)
C16	544.2(16)	6254.4(15)	6569.3(10)	63.3(4)
C17	306.1(18)	7286.5(16)	6051.5(11)	69.6(4)
C18	805.6(16)	7336.0(15)	5302.9(10)	65.6(4)
C19	1517.7(17)	6334.9(17)	5067.3(11)	68.7(4)
C20	1744.2(16)	5289.6(15)	5591.1(10)	64.0(4)
O21	538.2(15)	8415.7(12)	4844.2(9)	88.7(4)
C22	957(3)	8519(3)	4048.0(17)	98.1(7)
S1	5156.6(9)	1943.3(10)	5955.1(5)	111.5(4)
C2	4791(7)	1885(5)	4883(3)	112.2(12)
C3	3547(7)	1560(5)	4616(3)	103.6(11)
C4	2818(14)	1406(14)	5282(7)	102.9(16)
C8	2723(6)	74(6)	8129(4)	109(2)
C9	5063(4)	478(5)	7925(3)	102.0(13)
C10	3560(6)	-921(3)	6894(3)	104.6(13)
S1A	2609(18)	1313(19)	5152(9)	102.9(16)
C2A	3580(30)	1830(30)	4517(16)	103.6(11)
C3A	4730(30)	2130(30)	4997(16)	112.2(12)
C4A	4580(18)	2250(20)	5857(12)	111.5(4)
C8A	3352(10)	413(12)	8348(7)	109(2)
C9A	5072(7)	-40(8)	7478(6)	102.0(13)
C10A	2794(10)	-840(6)	7041(6)	104.6(13)

Anisotropic displacement parameters (Å^2 × 10^3) for MAC-0452936 (1). The anisotropic displacement factor exponent takes the form: $-2\pi^2[h^2a^{*2}U_{11} + 2hka^*b^*U_{12} + \ldots]$.

Atom	U_{11}	U_{22}	U_{33}	U_{23}	U_{13}	U_{12}
C5	67.5(8)	54.9(7)	66.3(9)	-5.2(6)	20.4(6)	4.2(6)
C6	58.1(7)	54.7(7)	62.6(8)	-3.2(6)	15.4(6)	1.8(6)
C7	72.3(9)	57.1(8)	73.1(9)	3.5(7)	20.0(7)	9.3(7)
N11	81.1(8)	58.0(7)	66.5(7)	8.2(6)	26.4(6)	13.8(6)
O12	83.8(7)	56.3(6)	65.1(6)	6.5(4)	29.4(5)	16.7(5)
C13	70.5(8)	56.7(7)	60.7(8)	-0.6(6)	24.0(6)	7.8(6)
O14	151.6(13)	79.7(8)	84.7(8)	21.0(7)	68.4(9)	40.8(8)
C15	58.8(7)	53.3(7)	54.9(7)	-3.3(6)	14.7(5)	2.6(6)
C16	73.6(9)	60.4(8)	59.3(8)	-3.8(6)	21.2(7)	6.9(7)
C17	81.0(10)	58.4(8)	72.1(10)	-1.6(7)	21.1(8)	13.8(7)
C18	68.2(8)	60.3(8)	67.6(9)	6.4(7)	10.8(7)	1.6(7)
C19	76.1(9)	75.4(10)	58.9(8)	5.4(7)	23.7(7)	6.0(8)
C20	70.7(9)	63.0(8)	62.4(8)	-1.3(7)	22.5(7)	9.3(7)
O21	104.5(10)	72.8(7)	91.9(9)	25.6(6)	26.0(7)	14.2(7)
C22	105.2(16)	97.1(16)	93.3(15)	37.1(13)	21.1(12)	-1.4(13)
S1	96.4(6)	152.3(8)	97.3(5)	-36.7(5)	48.2(4)	-49.1(5)
C2	149(3)	104(3)	104(2)	-17.1(18)	78(2)	-15(2)
C3	142(2)	110(3)	62.0(16)	1.9(16)	27.3(14)	23(2)
C4	91(4)	142(2)	76(3)	-16(2)	14(2)	-3(2)
C8	129(5)	97(4)	119(4)	47(3)	69(4)	41(4)
C9	85.4(15)	100(3)	111(3)	25(2)	-7(2)	8(2)
C10	159(5)	56.1(12)	105(2)	-2.0(14)	40(3)	4(2)
S1A	91(4)	142(2)	76(3)	-16(2)	14(2)	-3(2)
C2A	142(2)	110(3)	62.0(16)	1.9(16)	27.3(14)	23(2)
C3A	149(3)	104(3)	104(2)	-17.1(18)	78(2)	-15(2)
C4A	96.4(6)	152.3(8)	97.3(5)	-36.7(5)	48.2(4)	-49.1(5)
C8A	129(5)	97(4)	119(4)	47(3)	69(4)	41(4)
C9A	85.4(15)	100(3)	111(3)	25(2)	-7(2)	8(2)
C10A	159(5)	56.1(12)	105(2)	-2.0(14)	40(3)	4(2)

Oxime (*Z*)-18

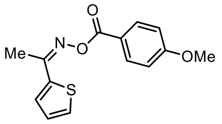

(*Z*)-**18**

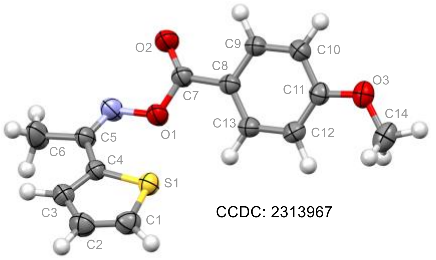

X-ray structure of oxime (*Z*)-**18**.

Crystal Data and Structure Refinement for (*Z*)-18.

Identification code	R1_01_97
Empirical formula	$C_{14}H_{13}NO_3S$
Formula weight	275.31
Temperature	295 K
Crystal system	orthorhombic
Space group	Pccn
Unit cell dimensions	a = 23.5686(17) Å, b = 7.7472(10) Å, c = 14.6932(10) Å
	α = 90°, β = 90°, γ = 90°
Volume	2682.8(4) Å
Z	8
ρ_{calc} (calculated density)	1.363 g/cm^3
absorption coefficient	2.183 μ/mm^{-1}
F(000)	1152.0
Crystal size	0.28 mm × 0.28 mm × 0.08 mm
Radiation	CuKα (λ = 1.54178)
2Θ range for data collection	7.502 to 148.938°
Index ranges	$-29 \leq h \leq 29$, $-9 \leq k \leq 8$, $-18 \leq l \leq 18$
Reflections collected	38504
Independent reflections	2739 [R_{int} = 0.0282, R_{sigma} = 0.0188]
Data/restraints/parameters	2739/0/175
Goodness-of-fit on F^2	1.068
Final R indexes [I≥2σ (I)]	R_1 = 0.0373, wR_2 = 0.1040
Final R indexes [all data]	R_1 = 0.0378, wR_2 = 0.1047
Largest diff. peak/hole / e Å^{-3}	0.27/–0.22

Fractional atomic coordinates (× 10^4) and equivalent isotropic displacement parameters (Å^2 × 10^3) for (*Z*)-18. U_{eq} is defined as 1/3 of the trace of the orthogonalized U_{IJ} tensor.

Atom	*x*	*y*	*z*	U(eq)
S1	7636.8(2)	4919.9(4)	2909.2(2)	49.04(16)
O1	6925.9(4)	5589.4(15)	1514.2(6)	55.2(3)
O2	6203.6(5)	4870.1(16)	582.7(8)	70.4(3)
O3	4887.7(4)	6511.4(13)	4237.8(6)	57.7(3)
N1	7288.3(5)	5338(2)	735.1(8)	60.9(3)
C1	8187.3(6)	4204(2)	3551.4(9)	56.9(3)
C2	8624.7(6)	3647(2)	3033.8(10)	61.6(4)
C3	8522.9(6)	3831(2)	2100.0(9)	55.8(3)
C4	7997.2(5)	4517.5(17)	1909.6(9)	45.1(3)
C5	7784.7(6)	4869.3(19)	997.7(10)	52.5(3)
C6	8197.6(7)	4673(3)	219.9(12)	82.0(6)
C7	6369.1(5)	5363.3(18)	1309.6(9)	47.5(3)
C8	6005.8(5)	5737.2(16)	2104.4(8)	43.0(3)
C9	5454.1(6)	5074.5(17)	2097.9(9)	49.1(3)
C10	5098.3(6)	5325.2(19)	2823.7(10)	51.6(3)
C11	5280.2(5)	6294.6(15)	3566.9(8)	45.1(3)
C12	5824.0(6)	6972.9(16)	3582.1(8)	48.8(3)
C13	6184.1(5)	6677.8(17)	2853.0(8)	48.2(3)
C14	5052.8(7)	7426(2)	5034.7(9)	67.0(4)

Anisotropic displacement parameters (Å^2 × 10^3) for (*Z*)-18. The anisotropic displacement factor exponent takes the form: $-2\pi^2[h^2a^{*2}U_{11} + 2hka^*b^*U_{12} + \ldots]$.

Atom	U_{11}	U_{22}	U_{33}	U_{23}	U_{13}	U_{12}
S1	47.7(2)	61.3(3)	38.1(2)	-0.29(11)	4.80(11)	-2.23(12)
O1	41.0(5)	85.6(7)	39.0(5)	-4.6(4)	0.9(3)	-2.7(4)
O2	51.8(6)	113.3(10)	46.1(6)	-16.1(5)	-2.1(4)	-8.5(5)
O3	59.2(5)	62.0(6)	52.1(5)	-3.4(4)	11.9(4)	4.8(4)
N1	48.3(6)	97.5(10)	36.9(6)	-0.4(6)	4.5(5)	-5.6(6)
C1	61.1(8)	64.8(8)	44.9(7)	4.7(6)	-6.5(6)	-6.8(7)
C2	52.7(8)	71.8(9)	60.4(8)	0.8(7)	-9.6(6)	2.1(7)
C3	44.5(6)	67.8(9)	55.2(8)	-10.5(6)	1.3(5)	-0.1(6)
C4	41.1(6)	53.2(6)	40.9(6)	-5.7(5)	3.4(5)	-8.1(5)
C5	44.2(7)	73.3(9)	40.0(7)	-5.9(5)	4.3(5)	-10.8(5)
C6	55.5(8)	147.0(18)	43.4(8)	-4.9(9)	10.3(7)	-5.1(10)
C7	42.6(6)	58.3(7)	41.7(7)	1.0(5)	-3.5(5)	-1.7(5)
C8	41.0(6)	46.2(7)	41.8(6)	0.6(5)	-2.9(4)	0.4(5)
C9	43.2(7)	57.4(8)	46.7(7)	-7.7(5)	-4.3(5)	-4.0(5)
C10	41.1(6)	59.9(8)	53.8(8)	-4.9(6)	-0.5(5)	-4.0(5)
C11	48.3(6)	42.4(6)	44.5(6)	2.7(5)	2.9(5)	7.4(5)
C12	54.2(7)	47.0(6)	45.2(6)	-5.4(5)	-4.2(5)	-0.9(5)
C13	44.0(6)	50.4(7)	50.2(7)	-3.1(5)	-3.8(5)	-5.7(5)
C14	87.6(10)	61.5(9)	52.0(9)	-6.3(6)	13.0(7)	8.1(8)
